# Enzyme-like polyene cyclizations catalyzed by dynamic, self-assembled, supramolecular fluoro alcohol-amine clusters

**DOI:** 10.1038/s41467-023-36157-0

**Published:** 2023-02-13

**Authors:** Andreas M. Arnold, Philipp Dullinger, Aniruddha Biswas, Christian Jandl, Dominik Horinek, Tanja Gulder

**Affiliations:** 1 Biomimetic Catalysis, Department of Chemistry, Technical University Munich, Garching, Germany; 2 Chair of Organic Chemistry, Faculty of Chemistry and Mineralogy, Leipzig University, Leipzig, Germany; 3 Institute of Physical and Theoretical Chemistry, University of Regensburg, Regensburg, Germany; 4 Catalysis Research Center, Technical University Munich, Garching, Germany

**Keywords:** Homogeneous catalysis, Natural product synthesis, Supramolecular chemistry

## Abstract

Terpene cyclases catalyze one of the most powerful transformations with respect to efficiency and selectivity in natural product (bio)synthesis. In such polyene cyclizations, structurally highly complex carbon scaffolds are built by the controlled ring closure of linear polyenes. Thereby, multiple *C*,*C* bonds and stereocenters are simultaneously created with high precision. Structural pre-organization of the substrate carbon chain inside the active center of the enzyme is responsible for the product- and stereoselectivity of this cyclization. Here, we show that in-situ formed fluorinated-alcohol-amine supramolecular clusters serve as artificial cyclases by triggering enzyme-like reactivity and selectivity by controlling substrate conformation in solution. Because of the dynamic nature of these supramolecular assemblies, a broad range of terpenes can be produced diastereoselectively. Mechanistic studies reveal a finely balanced interplay of fluorinated solvent, catalyst, and substrate as key to establishing nature’s concept of a shape-selective polyene cyclization in organic synthesis.

## Abstract (web summary)

Terpene cyclases enable the synthesis of (poly)cyclic carbon frameworks via ring closure of linear polyenes. Here, the authors report in-situ formed fluorinated-alcohol-amine supramolecular clusters that mimic terpene cyclases for shape-controlled polyene cyclizations.

## Introduction

**Fig. 1:**
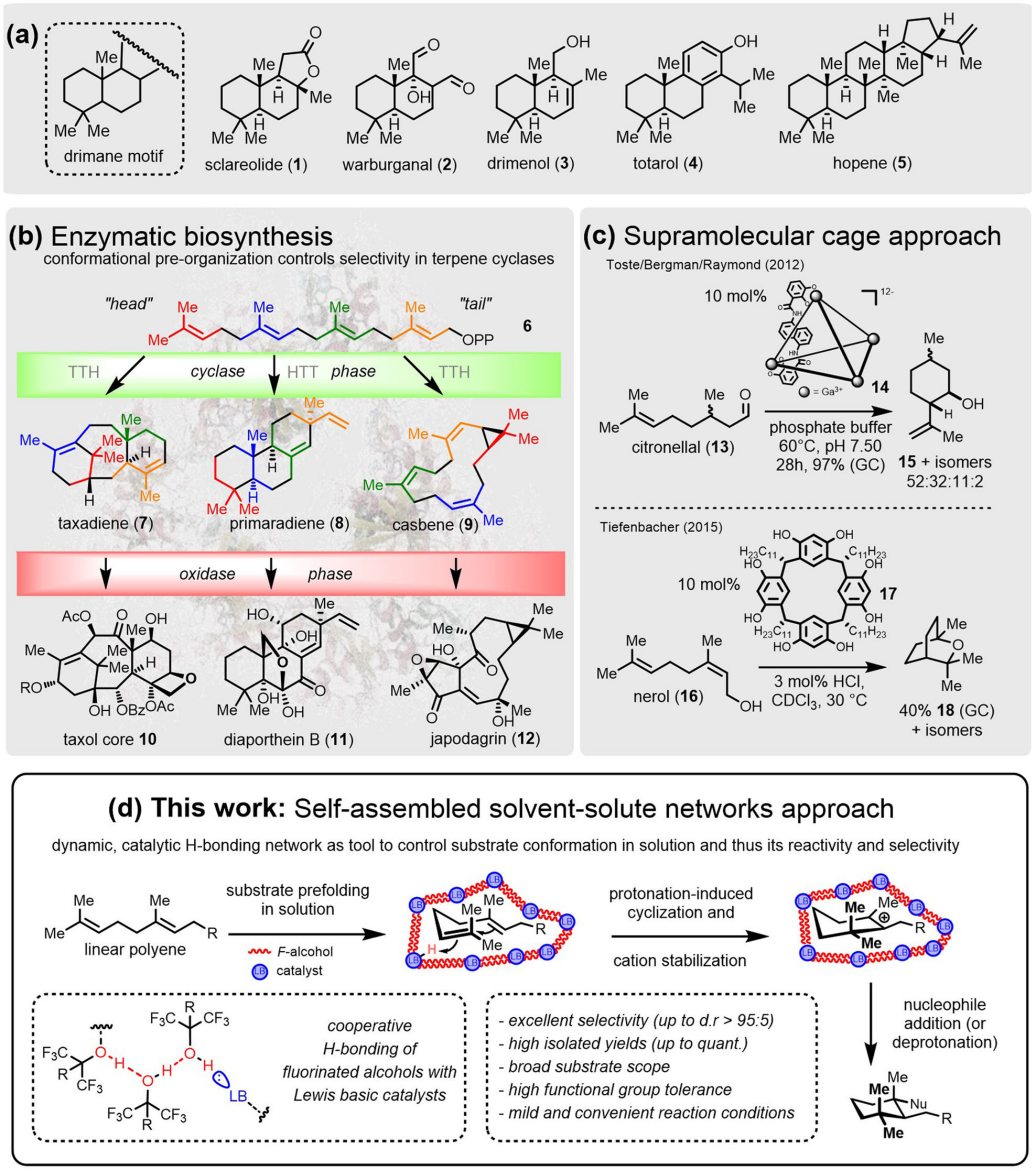
Examples of terpene natural products, their biosynthesis, and conceptual approaches for biomimetic polyene cyclizations. **a** Examples of versatile cyclic terpenes **1**–**5**. **b** Enzymatic terpene synthesis: the cyclase phase gives rise to structurally diverse hydrocarbon skeletons that are further elaborated during the oxidase phase. **c** Supramolecular approaches mimicking enzymatic confinement by employing macromolecular cages for selective cyclizations^44,45^. **d** Concept for macromolecular solvent clusters facilitating effective cyclizations through pre-arrangement of the apolar polyene chain in the dipolar solvent environment.

Terpenes are the largest class of natural products with hundreds of structures reported each year, totaling over 80,000 examples as of 2019^1^. Many terpenes exhibit interesting biological activities, e.g., to protect the producing organism from predators and/or pathogens and/or to enable intra- and inter-species chemical communication^2,3^. By utilizing a single enzyme class, the terpene cyclases, nature generates a remarkable number of structurally most diverse and often highly complex (poly)cyclic carbon frameworks. Even more impressive, this is accomplished from simple, achiral linear C_5_-isoprene derived precursors in a single step, as, for example, in the biosynthesis of the cyclic drimane terpenes (Fig. 1, top). Examples of these include the perfume ingredient and synthetic building block sclareolide (**1**)^4–7^, the antibacterials warburganal (**2**)^8^ and totarol (**4**)^9^, the antifungal drimenol (**3**)^10^ and the polycyclic anti-tumoral triterpene hopene (**5**, Fig. 1, top)^11^. Chemists have been fascinated for many decades by these remarkably complex, biosynthetic cyclization cascades. Mimicking the natural cyclization phase^12,13^ has been a major theme in biomimetic synthesis, even dating back to Eschenmoser’s and Stork’s seminal work on terpene assembly^14,15^. Since then, multiple publications have appeared offering access to a plethora of sophisticated structural terpene motifs, many of them arising from a protonation-assisted cyclization. However, when following the natural pathway, linear polyenes have to be treated with strong organic or inorganic Brønsted or Lewis acids such as trifluoroacetic acid^16^, fluorosulfonic acid^17–20^, SnCl_4_^21–25^, BF_3_·OEt_2_^26–28^, RuCl_3_^29–31^, and In(III) salts^30,32^, mostly under cryogenic conditions, to allow for productive ring closure. These synthetic methodologies suffer from several general drawbacks, including moderate diastereoselectivities and low functional group tolerance that overall lead to a narrow substrate scope^33–35^. Enantioselective polyene cyclizations have likewise been explored, with all known examples employing either Lewis or Brønsted super acids equipped with bulky, BINOL-derived ligands. These methods are not generally applicable as each catalytic system needs to be tailored for a specific and small set of substrates to effectively convey stereoinduction during cyclization^36^.

The harsh and strongly acidic conditions needed to conduct terpene cyclizations are in clear contrast to biosynthetic terpene formation, which nature performs with high precision under mild conditions at a physiological pH. Structural pre-organization of the substrate carbon chain within the enzyme’s active site is key to success. Depending on how the carbon chain of the polyprenol is folded inside the confined, hydrophobic pocket of the cyclase (Fig. 1b), various head-to-tail (HTT) or tail-to-head (TTH) cyclized hydrocarbons are delivered from identical precursors, utilizing the same reaction pathway in a shape-selective transformation. For example, geranylgeraniolpyrophosphate (**6**) is the sole precursor for the taxadiene **7**, that upon further enzymatic oxidations and tailoring give rise to taxol (**10**)^37^, to primaradiene (**8**, → diaporthein B, **11**)^38^ and to casbene (**9**, → japodagrin, **12**)^39^. Such a predictable control of molecular conformation in solution thereby steering the selectivity of the reaction^14^ has been a long-standing problem for organic chemists and is the ultimate motivation for the field of polyene cyclizations.

In the late 1960s, Breslow had already identified supramolecular frameworks exhibiting precise host-guest interactions on a molecular level as potential catalysts with enzyme-like properties and thus coined the term “artificial enzymes”^40^. Since then, multiple successful examples have been reported of creating molecular containers in the form of nanoscale cavities, pores, pockets, or channels to accelerate reaction performance and alter or enhance selectivity^41–43^. Only recently has this biomimetic concept entered the field of *C*,*C*-cyclizations. Self-assembled, polyanionic gallium catecholamide cages **14** can increase the chemoselectivity of the Prins cyclization of citronellal (**13**) to isopulegol (**15**) and its diastereomers by forming a hydrophobic pocket, thereby excluding water from the ring-closing step (Fig. 1c)^44^. The only terpene cyclization conducted in a host cavity so far was reported by Tiefenbacher and colleagues. The self-assembled, resorcinol-based hexameric capsule **17** efficiently promoted cyclization of linear precursors, such as nerol (**16**), to cyclic monoterpenes **18** with the key step being the cisoid-carbocation formation^45,46^. These examples constituted landmark conceptual discoveries. However, they still only convert a small set of compounds, because their predefined and rigid structures prevent them from reaching the broad applicability that small-molecule catalysis offers. Thus, for selective reactions, a confined space is necessary. While the first approaches have been made in this direction, methods to achieve this challenge in solution are vastly underexploited to date.

Inspired by these supramolecular approaches, in this work we aim to exploit and extend the concept of artificial enzyme catalysis in polyene cyclizations. The goal is to create a more flexible, building-block-oriented procedure. Emulating the mechanism as well as the conformation-determining properties of terpene cyclases, we create dynamic but structurally defined macromolecular assemblies in-situ. This is achieved by forming discrete, catalytically active Lewis acid-Lewis base complexes with a defined supramolecular structure assembled from fluorinated alcohols, such as 1,1,1,3,3,3-hexafluoroisopropanol (HFIP)^47–49^ or perfluoro-*tert*-butanol (PFTB), and catalytic amounts of ammonium or pyridinium salts. These are subsequently employed in a plethora of protonation-induced polyene cyclizations. We thereby succeed in transforming a broad variety of structurally most diverse alkenes into the corresponding ring-closed products with a huge functional group tolerance and high efficiency under complete stereocontrol. This catalytic method only requires cheap, off-the-shelf components to achieve conversion under mild and practical reaction conditions. Finally, we have a closer look at the mutual catalyst-solvent and substrate-solvent interplay. NMR investigations reveal structural folding of the substrate and the formation of 3D-self-assembled structures in solution, the latter being corroborated by molecular dynamics (MD) simulations.

## Results and discussions

### Reaction design and optimization

**Fig. 2:**
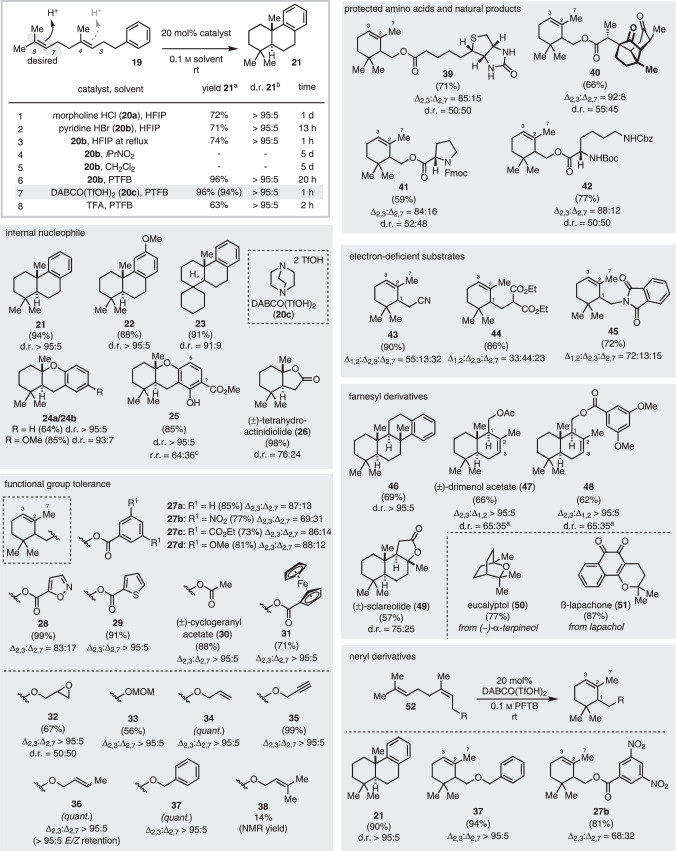
Optimization and scope of the proton-induced cyclization. Optimized reaction conditions: Substrate (1.0 eq) and DABCO(TfOH)_2_ (**20c**, 20 mol%) were dissolved in 0.1 m PFTB at rt under air and stirred until completion. All yields refer to isolated yields. ^*a*^Determined by GC-FID from the crude reaction mixture using an internal standard. Isolated yields are in parentheses. ^*b*^The diastereomeric ratio (d. r.) was determined by GC-FID or GC-MS. d.r. at *C*1. ^c^r. r. refers to *C*7:*C*5 CO_2_Me. HFIP  1,1,1,3,3,3-hexafluoroisopropanol, PTFB perfluoro-*tert*-butanol.

The starting point for our investigations was the halogenation-induced polyene cyclization we recently developed that provided unprecedented access to a manifold of iodinated, brominated, and even chlorinated carbocycles with excellent yields (up to 90%) and diastereoselectivities (up to >95:5)^50,51^. The fluorinated alcohol HFIP, together with a tailored Lewis basic electrophilic halogenation reagent, were decisive for a successful conversion. *N*-halo morpholines showed optimal performance in these halogenation-assisted polyene cyclizations. However, by design this approach always delivers halogenated products and is thus not suitable for the direct synthesis of non-halogenated terpene backbones. For the protonation-induced cation-π cyclization targeted within the current work, we thus began our studies by employing catalytic amounts of morpholinium salts with varying anions (20 mol% catalyst, Fig. 2 and Supplementary Information (SI), Fig. S1). Homogeranyl benzene (**19**) served as model substrate. Compound **19** possesses two *C,C-*double bonds of similar electrophilicity, of which the *C*7,*C*8-alkene must be chemoselectively addressed to achieve a selective transformation. The *trans*-decalin product **21** was obtained with excellent diastereoselectivity (>95:5), but reaction times (5h–1d) and chemical yields (8–72%) strongly depended on the anion of the morpholine salt (see SI). Morpholine hydrochloride (**20a**) gave the best results (72% yield after 1d, see entry 1, Fig. 2). Further extensive optimization of the reaction conditions including, i.a., the proton donor, solvent, and temperature (see SI for further information), identified HFIP, together with 20 mol% of the weakly acidic pyridinium hydrobromide (**20b**), as the ideal combination to furnish the desired tricyclic product **21** in 71% yield after 13 h at rt (Fig. 2, entry 2). Increasing the reaction temperature to reflux did significantly speed up conversion but also led to decomposition. Diverse side products were likewise obtained under all the above-mentioned conditions, mainly arising from protonation of the internal C_3_,C_4_ alkene, which were difficult to separate from the desired **21**. Importantly, reactions conducted in non-fluorinated solvents under these conditions did not result in any conversion at all (see entries 4 and 5, Fig. 2 and Supplementary Table 3, SI).

Only by switching the solvent to perfluoro-*tert*-butanol (PFTB) complete cyclization of **19** was observed, giving **21** as the only product (94% isolated yield) with perfect diastereoselectivity (d.r. >95:5). To the best of our knowledge, PFTB has only been used in mechanistic studies until now and has not been investigated as a solvent in organic chemistry^52–55^. While PFTB is already a weak acid (pKa = 5.4), conversion of **19** without a catalyst afforded **21** only in trace amounts after 13 h (see SI). After 7 days less then 50% conversion of **19** was detectable by GC-MS. It is noteworthy that the purity of the fluorinated alcohols needs to be checked carefully. Purification prior to use, as stated in the SI, is necessary as acidic contaminants from the production process can interfere with the catalytic transformation. Further investigations of the catalyst showed that DABCO ditriflate (**20c**) was even superior to pyridine HBr (**20b**), as it furnished the desired product **21** in excellent selectivity (d.r. >95:5) and again almost quantitative yield (96%; 94% isolated yield) in less than one hour (cf. entry 7, Fig. 2 and SI). Hence, employing DABCO(TfOH)_2_ (**20c**) in PFTB in the transformation offers the opportunity to significantly expand the substrate scope of polyene cyclizations as it likewise addresses electron-poor and thus slowly reacting substrates in a reasonable time (*vide infra*). When exchanging **20c** with strong organic or inorganic acids, many undesired products were produced, reducing the overall yield of **21**, e.g., to 63% for TFA (see entry 8, Fig. 2, Supplementary Table 2 and Supplementary Fig. 1, SI).

### Synthetic scope

To assess the synthetic utility of our cyclization cascade, we subjected a wide range of structurally and electronically diverse substrates to our reaction conditions (Fig. 2). Ring closure proceeded smoothly with *p*-methoxy substituted arene **22** (88% yield) or cyclohexyl derivative **23 (**91% yield), both giving the cyclic material with excellent diastereoselection (d.r. = 91:9 to > 95:5). Geranylphenols afforded products **24**–**25** in very good yields (64−85%) with diastereoselectivities of up to >95:5. Switching the terminating nucleophile to a carboxylic acid furnished the natural product (±)-tetrahydroactinidiolide (**26**) in 98% isolated yield. Next, we set out to test the functional group tolerance of our method. Different geranyl benzoates and heterocycles containing esters were smoothly converted into the monocyclic products **27**–**29** (73–99%). Because of the absence of suitable nucleophiles, the trimethyl cyclohexene derivatives were isolated as the major or even only products (69:31 to >95:5 r.r). Acetates and ferrocene esters were also suitable, selectively giving the *endo*-elimination products **30** and **31**. The mild reaction conditions even allowed the conversion of chemically sensitive substrates, such as epoxides, which formed **32** in 67% yield. Notably, the acid labile MOM protecting group in **33** was also tolerated, which is exceptional for acid-catalyzed reactions. Protonation occurred selectively at the prenyl portion, as allyl, propargyl, benzyl, and crotyl moieties were left untouched and gave products **34**–**37** in quantitative isolated yields and fully retained *E/Z* ratios. Only if two prenyl units were present, the reaction became sluggish, furnishing **38** only in 14% yield. In general, all tested geranyl ethers afforded the corresponding cyclohexenyl products **32**–**37** with >95:5 regioselectivity, independent of the workup (see Supplementary Table 4, SI for further information). This suggests that the deprotonation step trapping the transient carbocation occurs immediately after the cyclization step (cf. Fig. 1, bottom).

More complex starting materials, such as compounds bearing biotin, santonic acid, or amino acids equipped with Fmoc, Cbz, and even the Boc protecting groups, were successfully converted to the monocyclic products **39**–**42**. Electron deficient substrates leading to products **43**–**45** were likewise produced in up to 90% isolated yield. The regioisomeric ratio corresponded to that previously observed for halocyclizations^50,56^. The tetracyclic product **46** and the antimicrobial and anticancer natural product drimenol acetate (**47**), together with its corresponding dimethoxy ester **48**, were accessible from farnesol derivatives with complete diastereoselectivity, while homofarnesic acid provided the fragrance sclareolide (**49**) in 57% yield. The bicyclic monoterpene eucalyptol (**50**) and the DNA topoisomerase I inhibitor ß-lapachone (**51**) were smoothly obtained from α-terpineol and lapachol, respectively. Nerol-derived compounds **52** were likewise cyclized with almost unchanged regioselectivity and yield (**21**, **27b**, and **37**). Interestingly, when homoneryl benzene was used, the more stable *trans*-decalin product **21** was solely obtained with perfect diastereoselectivity. Kinetic studies for the conversions of **19** and **52a** showed that the neryl derivative **52a** was converted at a higher rate. In both cases, a stepwise process was obvious from the GC-MS data, suggesting equilibration of the alkene confirmation for **52a** before ring closure (see Supplementary Figs. 7–10, SI).

### Mechanistic studies

To investigate the reaction mechanism of this approach to polyene cyclizations, we subjected pyridinium deuterobromide (*d*-**20b**, 20 and 120 mol% loading) to the reaction mixture. In both cases, deuterium incorporation, at all sp^2^-carbon atoms that are susceptible to protonation and subsequent re-elimination, was detected in the final products *d*-**21**. This is consistent with the stepwise process seen in the kinetic investigations (cf. SI). From these results, we hypothesize:

1. that the active proton fluctuates in the H-bonding network of PFTB in a Grotthuß-like mechanism with a rapid exchange of the acidic protons in solution.
2. that a rapid and dynamic proton exchange between solvent and the catalyst occurs.

The determined kinetic isotope effect (KIE) value of 2.8 ± 0.1 (see SI) obtained by comparing the consumption rate of **19** in PFTB-OH and PFTB-OD further corroborated this assumption, as it revealed the importance of the PTFB-Lewis base H-bonding network in the rate-determining step^57^.

**Fig. 3:**
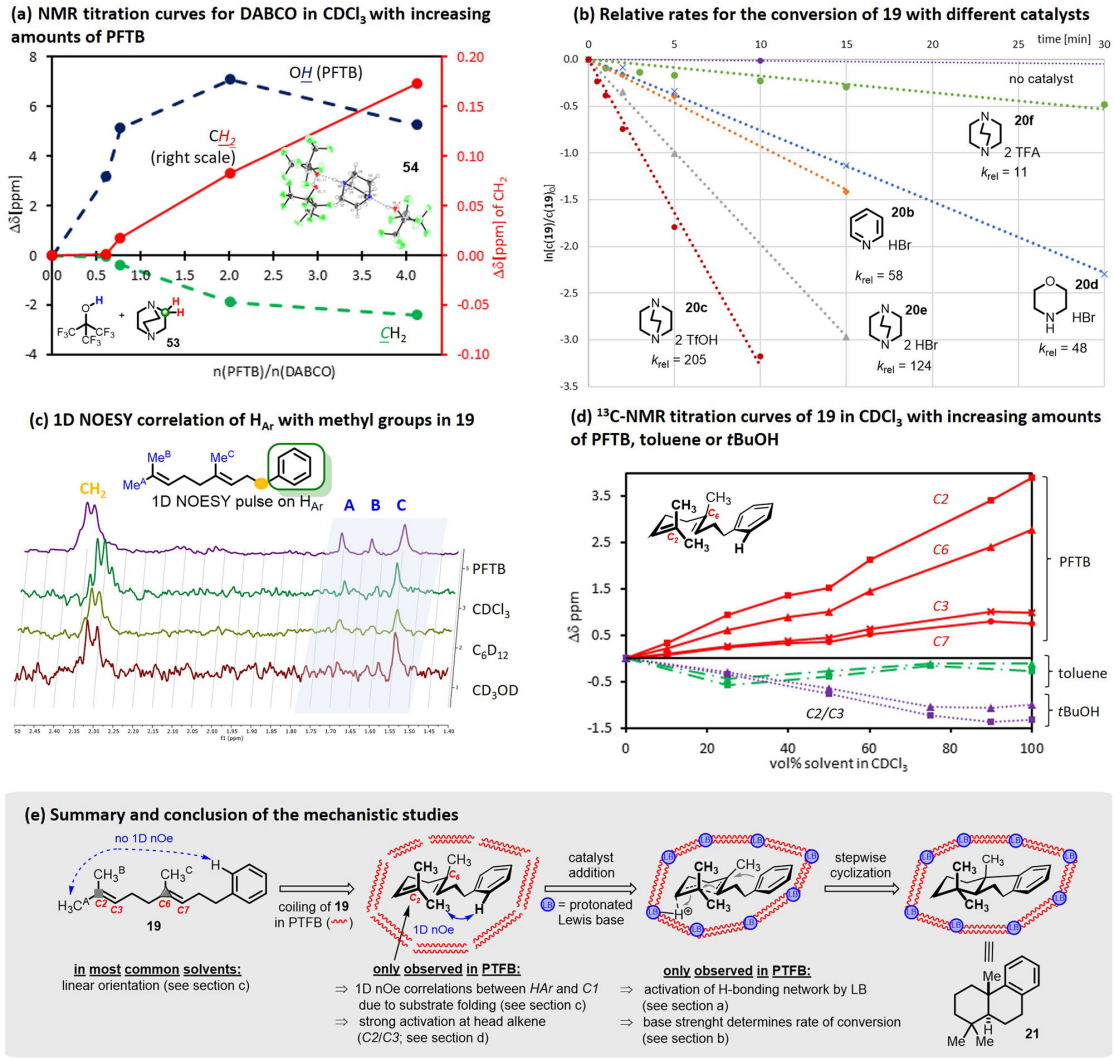
Mechanistic Investigations. **a** NMR titration curve for DABCO (**53**) in CDCl_3_ with increasing amounts of PFTB shows the interaction of the alcohol with the Lewis base. Insert: Crystal structure of DABCO(PFTB)_3_ salt (**54**). **b** Relative rates for conversion of **19** with different catalysts. For more experimental details see the SI. **c** 1D-NOESY correlation of aromatic protons with the methyl groups in **19**. Spectrum was recorded by selective pulsing on H_Ar_ (7.25 ppm as band center) using a 0.1 m solution of **19** in the respective solvent. **d** ^13^C-NMR titration curves of **19** in CDCl_3_ with increasing amounts of PFTB, toluene, or *t*BuOH show that the strongest activation is observed in PFTB at *C2* over *C6*, explaining the selective protonation of the terminal olefin. **e** Mechanistic summary of proton-induced polyene cyclization.

The role of the Lewis base in our system was examined by titrating DABCO **53** with increasing amounts of PFTB, which resulted in a significant downfield shift of the alcohol proton from 3.6 ppm to 10.7 ppm. This indicates strong H-bonding interactions between the Lewis base and PFTB (Fig. 3a and SI). The change of ^1^H-chemical shifts of the methylene groups in DABCO was less distinct upon the addition of PTFB, yet visible in the ^13^C-NMR spectra. This suggests that strong H-bonding interactions indeed take place between the Lewis base and the solvent, as previously observed for *N*-bromo morpholine and HFIP^50^. Further increasing the amount of PFTB to >4 equivalents resulted in precipitation of a PFTB-DABCO complex **54** (X-ray structure Fig. 3a, insert and SI). Similar to reported HFIP-amine salts^50,58^, two PTFB molecules share one proton with the ammonium cation nearby within this complex. In the case of DABCO, however, the pK_a_ of the second amine is 3.0 (vs. 5.4 for PFTB) and thus attracts another molecule of PFTB through strong H-bonding interactions. This results in a final stoichiometry of DABCO/PFTB of 25:75.

We then investigated the structure-reactivity relationship of the catalyst regarding both the cation and the anion (Fig. 3b). While without catalyst no significant conversion of **19** was visible, an increase in reaction rate was observed by applying the protonated *N*-compounds morpholine, pyridine, and DABCO in the form of their hydrobromide salts. The *k*_*rel*_ increased with decreasing pKa value. Changing the anion further accelerated the transformation. Applying DABCO bistriflate (**20c)** showed an almost doubled reaction rate (*k*_*rel*_ = 2.05 × 10^2^) when compared to its hydrobromide derivative (*k*_*rel*_ = 1.24 ×10^2^) and an even 20 fold increased rate constant relative to that of the DABCO TFA salt (**20f**, *k*_*rel*_ = 1.1 × 10^1^). Gutmann-Becket^59,60^ experiments revealed that besides the Brønsted acidity the catalyst’s Lewis acidity, and thus the strength of the mutual interaction of amine salts and F-alcohol within the H-bonding network, plays an important role in the cyclization event (see SI). In general, the acceptor number (AN) of fluorinated alcohols does not correlate with their pKa value but rather with their ability to coordinate Lewis basic molecules. Upon addition of our catalysts **20** to PTFB the Lewis acidity further increased (AN (PTFB) = 53.8 vs. AN (PTFB + **20b**) = 54.1 vs. AN (PTFB + **20c**) = 59.8) surpassing even the Lewis acidity of SnCl_4_ (AN = 59.0) if PTFB is activated by DABCO ditriflate **20c**.

Next, we turned our attention to the influence of the extended H-bonding network on reaction selectivity. Within the scope of this work, we have shown that fluorinated alcohols, particularly PFTB play an important role in the selective cyclization of mono- and sesquiterpenes. As hypothesized earlier by Denmark^61^ and our group^50^, fluorinated alcohols are capable of controlling the conformation of apolar linear materials by forming cyclic, linear, or helical supramolecular clusters in solution^62–66^. These domains force the substrates to prearrange in their thermodynamically favored chair-like conformation. This shape-induced control enables the transformation to proceed with excellent yields and selectivities. To test this hypothesis, we recorded 1D-^1^H-NOESY spectra of model substrate **19** in various solvents by selectively resonating **19** at the aromatic protons (Fig. 3c and SI). Only in PFTB did the protons of the terminal C1 Me-groups show significant NOE contacts to aromatic H-atoms, which can only be explained by the folding of **19** and thus reduction of the surface area of this lipophilic polyene substrate. Subjecting **19** to ^13^C-NMR titration with increasing amounts of PFTB, toluene, and *tert*-butanol in CDCl_3_ revealed that only in PFTB did the sp^2^-carbons significantly shift downfield, doing so by up to 3.5 ppm for *C*2 (Fig. 3d and SI). This observation was due to fluorine-π interactions stemming from the hydrophobic CF_3_ groups clustering around the apolar starting material^62^ and thus activating the more exposed head alkene (*C2/C3*) the most. This finding nicely explains the superior selectivity of terminal vs. internal protonation in **19** in PFTB. In other solvents, no such activation was observed, thus resulting in the production of significant amounts of apolar side products.

Altogether, these investigations clearly reveal that the strong H-bonding network formed by non-covalent interactions of catalyst **20c** and PTFB plays a decisive role in controlling the solution phase conformation and thus steers the outcome of the reaction. The microstructured solution forces the linear polyene **19** into a distinct chair,chair-like conformation simultaneously activating selectively the terminal alkene moiety and stabilizing cationic intermediates and transition states. These aspects of structural pre-organization with their impact on reactivity and selectivity in chemical transformations are key to explaining the biogenic isoprene rule formulated by Eschenmoser^14^ and Stork^15^.

### Molecular dynamics (MD) studies

**Fig. 4:**
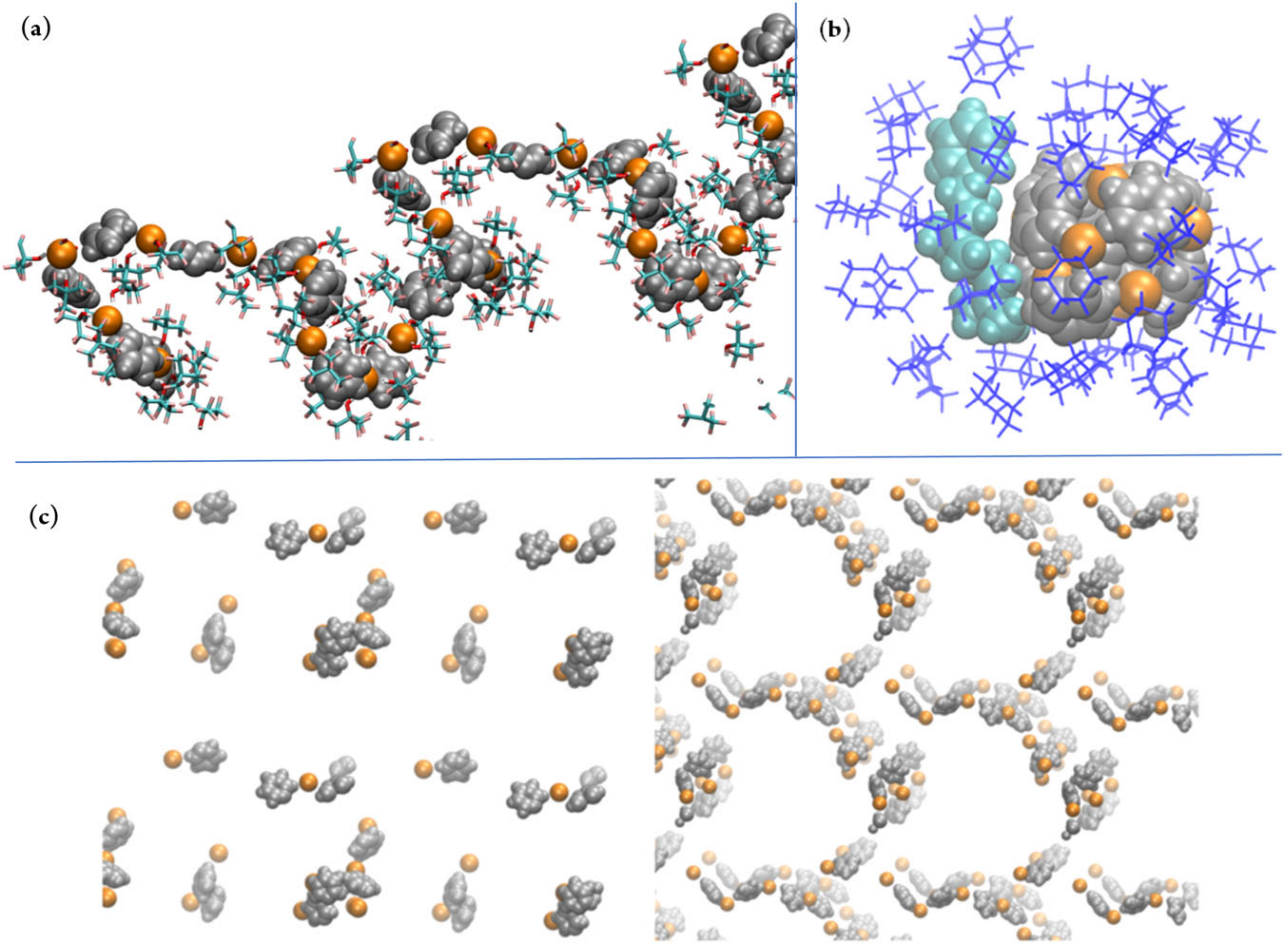
Solvent dependent change of ionic aggregation observed in MD simulations. Formation of supramolecular aggregates in **a** PFTB (first layer as sticks) and **b** cyclohexane (blue sticks). In **a** the polar hydroxyl groups of the immediate PFTB molecules (stick) are facing the pyridinium bromide aggregates and CF_3_ groups the bulk. **c** Aggregates observed in MeOH are less well defined (left side), or well defined (right side). The brightness of the atoms reflects their distance to the observer in the direction perpendicular to the focal plane; VdW representation is used for cations (silver), anions (orange), and **19** (turquoise).

The postulated role of the solvent was further explored by extensive MD studies (simulation details are given in the SI). Similar to the structuring induced by HFIP^62^, a clear structuring of the polar and apolar structural elements by the polar F-alcohol was observed. The most prominent result was the formation of supramolecular, linear ionic aggregates of the catalyst (pyridinium hydrobromide (**20b**), Fig. 4a and DABCO bistriflate (**20c**) see SI) in PFTB. This was facilitated by the polar hydroxy groups of PFTB, which preferentially face the charged ions and the apolar CF_3_ groups pointing towards the bulk solution, thereby creating distinct and structured catalytic domains in the solution by a different hydrogen bonding network compared to the bulk. This gives rise to a percolating global microstructure because the linear aggregates of pyridinium bromide **20b** are not limited to a local inhomogeneity as in cyclohexane (Fig. 4b). The latter causes a depletion of ions in the bulk and allows contact with the ions only at the ion-cyclohexane interface. In PTFB, however, a new preferential orientation of hydrogen bonds arises through the inclusion of **20b**, which is not present in pure PTFB. This prearrangement could potentially support proton transfer along the aggregates and follow a Grotthus-like diffusion, as similarly observed in the cyclase II enzyme *ent*-copy diphosphate synthase^67^. The non-point-like pyridinium cation is likewise important, as such structuring could not be observed in control simulations where **20b** was substituted by NaBr.

Aggregates like those occurring in PTFB were likewise observed in control simulations using other polar, organic solvents such as MeOH (Fig. 4c), but were overall less well-defined. The question of whether the solvent itself facilitates prearrangement in the coiled state of **19** was tackled by comparing potentials of mean force (PMFs) obtained from umbrella sampling^68^ and the weighted histogram analysis method^69^ for the bond-forming carbon atoms of **19**. For all observed solvents, the folded form of **19** was less favorable. However, with the addition of the ions, the coiled conformation of **19** was stabilized in PTFB as opposed to destabilized in cyclohexane (see SI). As the reaction can only happen from the coiled form, the preaggregation gives an additional favorable contribution to the reaction.

### Conclusions

By pre-organizing substrate conformation, nature controls and directs the outcome of its chemical reactions with high efficiency. Terpene cyclizations are impressive examples of such shape-selective transformations, providing a manifold of different and structurally complex carbon frameworks depending on how the carbon chain gets folded inside the enzymatic pocket. Controlling substrate conformation in solution, as terpene cyclases do, and thus steering the selectivity of a certain transformation predictably is yet an unmatched challenge for organic chemists. With our studies, we introduced a broadly applicable method for the head-to-tail proton cyclization of structurally different linear polyenes to the organic synthetic toolbox. This was accomplished by mimicking the decisive enzymatic features of (a) adjusting the reagent and/or substrate’s reactivity, (b) stabilizing transition-states and intermediates, and (c) controlling substrate conformation within our in-situ formed, self-assembled, cooperative supramolecular network consisting of PTFB and catalytic amounts of ammonium or pyridinium salts. These dynamic PTFB-amine-supramolecular clusters show all decisive properties and are perfectly suited to be employed in carbocation cascade reactions, and thus, in the total synthesis of terpenes and their artificial analogs. Our approach is very mild, tolerating many functional groups including acid-labile functionalities. The procedure is operationally simple and robust, does not require inert or cryogenic conditions, and needs only affordable, commercially available catalysts and solvents. It provides polycyclic products with generally high to excellent yields and simultaneously assembles multiple stereogenic centers. The excellent diastereoselectivity (up to d.r > 95:5) is caused by the controlled conformational pre-folding of the substrate chain inside the supramolecular assemblies. Due to the size-adaptive nature of our supramolecular networks, the reaction offers a broad substrate scope. Applying this methodology, we have efficiently synthesized a broad range of terpene-type structural scaffolds, including the natural products tetrahydroactinidiolide (**26**), cyclogeranylacetate (**30**), drimenol acetate (**47**), sclareolide (**49**), eucalyptol (**50**), and β-lapachone (**51**). Through our mechanistic investigations, important insights into the underlying principles of H-bonding networks and their influence on reactivity and selectivity are now available. These principles will help to further advance the field of fluorous alcohol-triggered reactions from pure serendipity to a more rational reaction design. In addition, our studies impressively revealed that PTFB, which had not yet found its way into organic synthesis, can indeed serve as a versatile solvent, pushing distinct transformations even in a superior fashion to its more popular relative HFIP. PFTB constitutes an excellent reaction medium for proton-induced polyene cyclization. However, at the very least, it will also be effective for any transformation needing cationic species to be stabilized, in a dipolar medium, and mildly acidic conditions. We hope that PFTB will rise from a laboratory curiosity to a valuable solvent like HFIP.

## Methods

### General procedure for the cyclization of linear polyenes

The corresponding linear polyene substrate was dissolved in 0.1 m PFTB at rt in a 4 mL screw cap vial with a magnetic stir bar. In total 20 mol% of DABCO(TfOH)_2_ (**20c**) was added and the mixture was stirred until completion (determined by GC or TLC). Then, 2 mL CHCl_3_ was added to terminate the reaction and the solvent was removed under reduced pressure. The crude mixture was then directly purified by column chromatography on silica gel to obtain the corresponding product.

## Supplementary information

Supplementary Information

Description of Additional Supplementary Files

Supplementary Data 1

Supplementary Data 2

Supplementary Data 3

## Data Availability

The authors declare that the data supporting the findings of this study are available within the paper and its supplementary information files. The NMR data of compounds 21–51 can be found in the Supplementary Data 1. Supplementary Data 2 contains the structural data for compound **54** including its cartesian coordinates. In addition, the data for the X-ray crystal analysis for compound **54** generated in this study have been deposited under accession number CCDC 2205029 at The Cambridge Crystallographic Data Centre. Supplementary Data 3 contains the parameters for the simulations conducted in this study.
