# Supplementary material for: Enzyme-like polyene cyclizations catalyzed by dynamic, self-assembled, supramolecular fluoro alcohol-amine clusters
Source: Nat Commun. 2023 Feb 13;14:813. doi: 10.1038/s41467-023-36157-0 (PMC9925744; doi:10.1038/s41467-023-36157-0)
Supplement: Supplementary file 1 — Supplementary Information [file 41467_2023_36157_MOESM1_ESM.pdf]

Enzyme-Like Polyene Cyclizations Catalyzed by Dynamic, Self-Assembled, Supramolecular Fluoro Alcohol-Amine Clusters

Andreas M. Arnold,[1,2] Philipp Dullinger,[3] Aniruddha Biswas,[2] Christian Jandl,[4] Dominik Horinek[3] and Tanja Gulder[1,2,4*]

[1]Biomimetic Catalysis, Department of Chemistry, Technical University Munich, Lichtenbergstraße 4, 85747 Garching, Germany
[2]Chair of Organic Chemistry, Faculty of Chemistry and Mineralogy, Leipzig University, Johannisallee 29, 04103 Leipzig, Germany[3]Institute of Physical and Theoretical Chemistry, University of Regensburg, 93040 Regensburg, Germany
[4]Catalysis Research Center, Technical University Munich, Ernst-Otto-Fischer-Straße 1, 85747 Garching, Germany

# – SUPPLEMENTARY INFORMATION –

1. General Information 1

2. Preparation of DABCO(TfOH)$_2$ (20c) and DABCO(TFA)$_2$ (20f) 2

3. Optimization of Reaction Conditions 3

4. Mechanistic Studies 6

4.1 Preparation of PFTB-OD and Pyridine-DBr (*d*-**20b**) 7

4.2 Reaction of Homogeranyl Benzene (**19**) using Catalyst *d*-**20b** in Deuterated PTFB 7

4.3. Kinetic Isotope Effect (KIE) Determination 8

4.4. Determination of the Reaction Rate depending on the Catalysts 9

4.5. Time-Dependent Conversion of Homogeranyl Benzene (**19**) and Homoneryl Benzene (**52a**) 10

4.6. 1D-1H-NOESY Investigations on the Folding of Homogeranylbenzene (**19**) in Different Solvents 12

4.7. Comparison of the Lewis Acidities of *t*BuOH, TFE, HFIP, PTFB, PTFB + **20b**, and PTFB + **20c** using the Gutmann-Beckett Method 14

4.8. NMR Investigations on Non-Covalent Interactions of Homogeranyl Benzene (**19**) and Different Solvents 15

4.9 Transformation of Homogeranyl Benzene (**19**) using Trifilic Acid (TfOH) as Catalyst 16

4.10 Transformation of Homogeranyl Benzene (**19**) performed in the presence of 2,6-di-*tert*-butyl pyridine 16

5. General Procedure for the Cyclization of Linear Polyenes 17

6. Physical and Spectroscopic Data of Compounds 21 – 51 17

7. X-ray Crystal Analysis of DABCO-PFTB 54 (CCDC 2205029) 35

7.1 Crystal Data 35

7.2 Data Collection 35

7.3 Refinement 36

8. Simulation Details 38

9. Supplementary References 41

1. General Information

All solvents used in reactions were p.A. grade. Solvents for chromatography were technical grade and distilled before use. Anhydrous dichloromethane, diethyl ether, and THF were obtained from a MBraun MB-SPS 800 solvent purification system. Other dry solvents were obtained from Fluka and Acros in the highest purity available and used without further purification. 1,1,1,3,3,3-hexafluoroisopropanol (HFIP) and nonafluro-*tert*-butanol (PFTB) was purchased from Fluorochem with a purity >99%. Reagents were purchased at the highest commercial quality and used without further purification. Yields refer to chromatographically and spectroscopically (^{1}H NMR) homogeneous material unless otherwise stated. Reactions were monitored by thin layer chromatography (TLC) carried out on Merck silica gel aluminium plates with Fan -254 indicator using UV light as the visualizing agent (UV), basic potassium permanganate solution ($KMnO_4$), ceric ammonium molybdate (CAM), and heat as developing agents, or by GC-FID or GC-MS where applicable. Silica gel Merck 60 (particle size 40 – 60 µm) was used for flash column chromatography. Solvent mixtures are understood as volume/volume. NMR spectra were recorded on Bruker AV300, Bruker AV400, Bruker AV500, or Bruker AV500-cryo spectrometers. The spectra were calibrated using residual undeuterated solvent as an internal reference ($CDCl_3$ @ 7.26 ppm, $CDCl_3$ @ 77.16 ppm ^{13}C NMR). The following abbreviations (or combinations thereof) were used to explain the multiplicities: s = singlet, d = doublet, dd = doublet of doublets, t = triplet, dt = doublet of triplets, q = quartet, quint = quintet (with 1:2:3:2:1 intensity), hept = heptet, m = multiplet, br = broad. In addition, the following abbreviations were used: EtOAc = ethyl acetate, MeCN = acetonitrile, DCM = dichloromethane, DCE = 1,2-dichloroethane, TFE = 1,1,1-trifluoroethanol, HFIP = 1,1,1,3,3,3-hexafluoro-2-propanol, PFTB = nonafluoro-*tert*-butanol, TLC = thin layer chromatography, rt = room temperature, sat = saturated. Melting points were measured on a Büchi 510 and are not calibrated. IR spectra were recorded on a JASCO FT-IR-4100 (ATR) and are reported in terms of frequency of absorption (cm^{-1}). Mass spectra were conducted on a Finnigan MAT SSQ 7000 (MS-EI, 70 eV; CI, 100 eV), or a Thermo Scientific LTQ-FT ultra and ThermoFisher Scientific LTQ Orbitrap XL spectrometer (ESI HRMS). Diastereomeric ratios (d.r.) were determined using GC-FID or GC-MS. The following method was used for GC separation: 60 °C 3 min, 15 °C/min → 250 °C, 250 °C 5 min.

2. Preparation of DABCO(TfOH)$_2$ (20c) and DABCO(TFA)$_2$ (20f)

2.00 g DABCO (**53**, 17.8 mmol, 1.0 eq) were dissolved in 40 mL abs. CH_2Cl_2. At 0 °C, 3.31 mL trifluoromethanesulfonic acid (5.61 mL, 37.4 mmol, 2.1 eq) was added dropwise. The mixture was allowed to stir for 30 min at rt and then the volatiles were removed under reduced pressure. The crude product was twice triturated with abs. isopropanol and dried under a high vacuum to give 6.06 g **20c** (14.7 mmol, 83%) as a colorless solid.

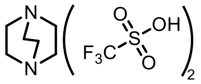

20c

m.p. 320 °C (dec., *i*PrOH); **^{1}H NMR** (400 MHz, DMSO-*d*$_6$) δ 3.54 (s, 12H) ppm; **^{13}C NMR** (101 MHz, DMSO-*d*$_6$) δ 120.7 (q, *J* = 322.2 Hz, 2C), 42.95 (6C); **^{19}F NMR** (377 MHz, DMSO-*d*$_6$) δ -77.75 ppm; **IR** (KBr) $\tilde{\nu}_{max}$ = 3450, 1266, 1178, 1038, 648, 521 cm^{-1}.

500 mg DABCO (**53**, 4.45 mmol, 1.0 eq) were dissolved in 15 mL dry CH_2Cl_2. At 0 °C, and 825 µL trifluoromethanesulfonic acid (1.40 g, 9.35 mmol, 2.1 eq) was added dropwise. The mixture was allowed to stir for 30 min at rt and then the volatiles were removed under reduced pressure. The crude product was twice triturated with abs. isopropanol and dried under a high vacuum to give 1.34 g DABCO(TFA)$_2$ (**20f**, 14.7 mmol, 88%) as colorless solid.

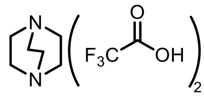

20f

m.p. 235 °C (*i*PrOH); **^{1}H NMR** (300 MHz, acetone-*d*$_6$) δ 9.18 (brs, 2H), 3.56 (s, 12H) ppm; **^{19}F NMR** (282 MHz, acetone-*d*$_6$) δ -76.06 ppm; **IR** (KBr) $\tilde{\nu}_{max}$ = 3666, 1681, 1437, 1199, 1141, 722 cm^{-1}.

3. Optimization of Reaction Conditions

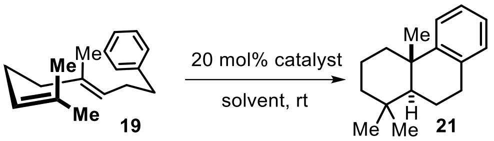

22.8 mg Homogeranyl benzene (**19**, 100 µmol, 1.0 eq) and 20 mol% catalyst (20.0 µmol) were mixed in 1.00 mL solvent and the reaction was monitored via GC-FID.

Supplementary Table 1. Proton-induced cyclization of **19**.

entry	solvent	catalyst	time	conversion	yield 21[a]	d.r.[a]
1	HFIP	-	1 d	> 95%	61%	> 95:5
2	HFIP[b]	-	7 d	< 5%	-	-
3	HFIP[b]	mor-HCl (**20a**)	1 d	> 95%	72%	> 95:5
4	HFIP[b]	mor-HBr	5 h	> 95%	69%	> 95:5
5	HFIP[b]	mor-TosOH	1 d	12%	8%	-
6	HFIP[b]	mor-H_2SO_4	1 d	> 95%	65%	> 95:5
7	HFIP[b]	mor-AcOH	20 d	< 1%	-	-
8	HFIP[b]	py-HCl	1 d	> 95%	66%	> 95:5
9	HFIP[b]	py-HBr (**20b**)	13 h	> 95%	71%	> 95:5
10	HFIP[b]	py-HI	15 h	> 95%	63%	> 95:5
11	HFIP[b]	py-TfOH	3 h	> 95%	63%	> 95:5
12	HFIP[b]	py-3-SO_3H	1 h	> 95%	61%	> 95:5
13	HFIP[b]	py-HPF_6	12 h	> 95%	57%	> 95:5
14	HFIP[b]	py-$HClO_4$	1 h	> 95%	67%	> 95:5
15	HFIP[b]	py-SO_3	1 h	> 95%	64%	> 95:5

[a]Determined by GC-FID from the crude reaction mixture using an internal standard. [b]HFIP was distilled from a 3 Å/4 Å molecular sieve and 250 mg KOH/100 mL HFIP. mor = morpholine, py = pyridine, TosOH = *p*-toluolsulphonic acid, AcOH = acetic acid, TfOH = triflic acid

Supplementary Table 2. Screening of organic and inorganic acids.

entry	solvent	catalyst	time	conversion	yield 21[a]	d.r.[a]
1	HFIP[b]	TosOH·H_2O	15 min	> 95%	62%	> 95:5
2	HFIP[b]	H_2SO_4	15 min	> 95%	59%	> 95:5
3	HFIP[b]	TFA	15 min	> 95%	56%	> 95:5
4	HFIP[b]	$CF_3SO_2NH_2$	1 d	93%	59%	> 95:5
5	HFIP[b]	C_6F_5OH	14 d	> 95%	61%	> 95:5
6	PFTB	AcOH	2 d	> 95%	71%	> 95:5
7	PFTB*	H_2SO_4	30 min	> 95%	84%	> 95:5
8	PFTB	TFA	2 h	> 95%	63%	> 95:5

[a]Determined by GC-FID from the crude reaction mixture using an internal standard. [b]HFIP was distilled from 3 Å/4 Å molecular sieve and 250 mg KOH/100 mL HFIP. *ca. 15% of substrate **19** was converted by protonation of the internal alkene moiety leading to regioisomeric carbon cycles that could not be removed from **21**. TosOH = *p*-toluolsulphonic acid, AcOH = acetic acid, TfOH = triflic acid, TFA = trifluoro acetic acid.

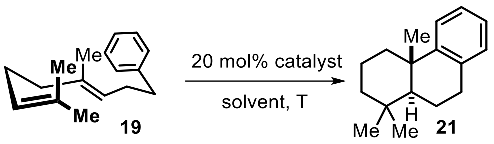

22.8 mg Homogeranyl benzene (**19**, 100 µmol, 1.0 eq) and 20 mol% catalyst (20.0 µmol) were mixed in 1.00 mL solvent and the reaction was monitored via GC-FID.

Supplementary Table 3. Screening of different solvents for the cyclization of **19**.

entry	solvent	catalyst	time	conversion	yield 21[a]	d.r.[a]
1	HFIP	-	1 d	> 95%	61%	> 95:5
2	HFIP[b]	-	7 d	< 5%	-	-
3	HFIP[b]	py-HBr (**20b**)	13 h	> 95%	71%	> 95:5
4	HFIP (65 °C)[b]	py-HBr (**20b**)	1 h	> 95%	74%	> 95:5
5	Et_2O	py-HBr (**20b**)	5 d	< 1%	-	-
6	*i*$PrNO_2$	py-HBr (**20b**)	5 d	< 1%	-	-
7	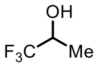	py-HBr (**20b**)	5 d	44%	28%	-
8	CH_2Cl_2	py-HBr (**20b**)	5 d	< 1%	-	-
9	*i*PrOH	py-HBr (**20b**)	5 d	< 1%	-	-
10	TFE	py-HBr (**20b**)	5 d	75%	5%	-
11	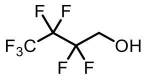	py-HBr (**20b**)	5 d	2%	-	-
12	PFTB	py-HBr (**20b**)	24 h	> 99%	96%	> 95:5
13	PFTB/HFIP (1:9)	py-HBr (**20b**)	2 h	> 99%	71%	> 95:5
14	PFTB	py-HBr (**20b**)	20 h	> 99%	96%	> 95:5
15	PFTB	DABCO$(TfOH)_2$ (**20c**)	1 h	> 99%	96% (94%)	> 95:5

[a]Determined by GC-FID from the crude reaction mixture using an internal standard. Isolated yields are in parentheses. [b]HFIP was distilled from a 3 Å/4 Å molecular sieve and 250 mg KOH/100 mL HFIP. py = pyridine, DABCO = 1,4-diazabicyclo[2.2.2]octane, TfOH = triflic acid

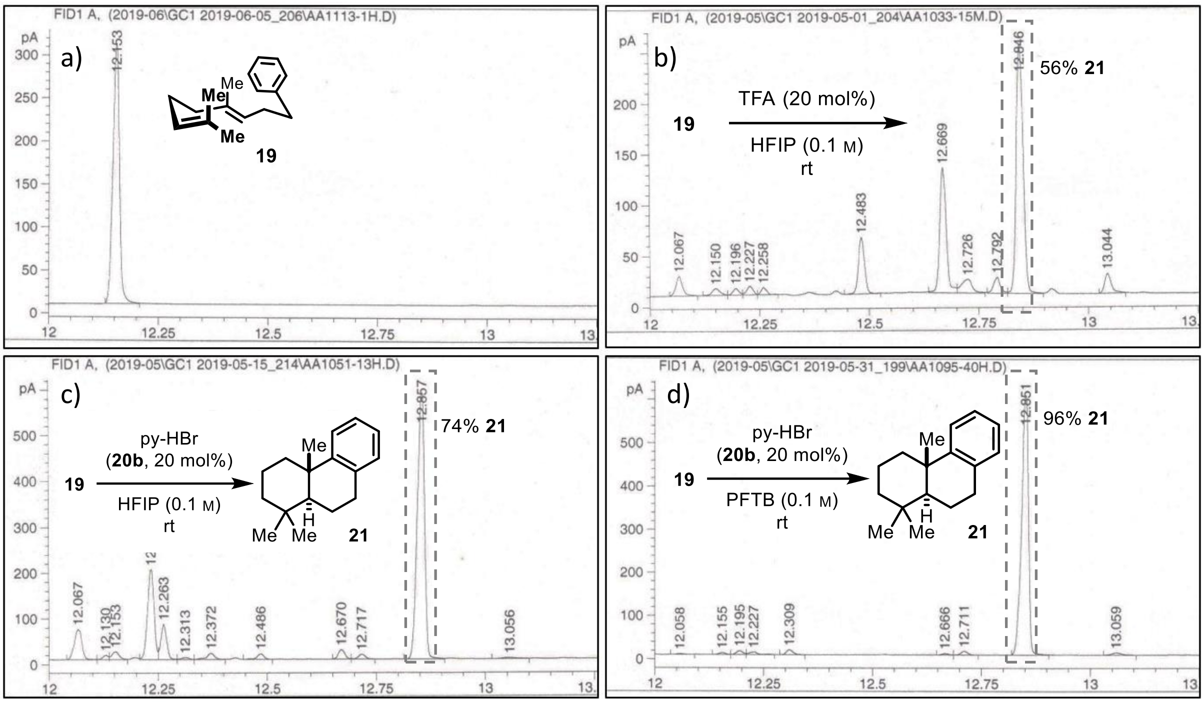

Supplementary Figure 1. GC traces of a) **19**, and conversion of **19** using b) TFA in HFIP (Table S2, entry 3); c) **20b** in HFIP (Table S3, entry 3); d) **20b** in PTFB (optimized conditions; Table S3, entry 15).

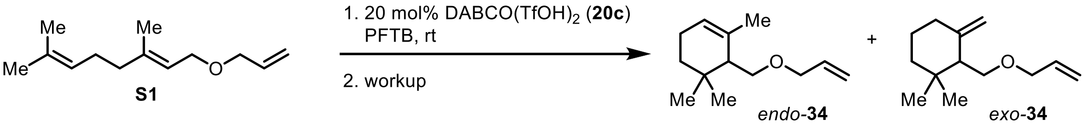

19.5 mg allyl geraniol allyl ether (**S1**, 100 µmol, 1.0 eq) and 8.20 mg DABCO(TfOH)$_2$ (**20c**, 20.0 µmol, 20 mol%) were stirred in 1.00 mL PFTB at rt. After completion, the reaction mixture was evenly distributed into 5 vials and treated as described below. The crude mixtures were then diluted with 10 mL CH_2Cl_2, filtered (entries 2-3 and 5), partitioned between water (10 mL) and CH_2Cl_2 (15 mL), and extracted twice with CH_2Cl_2. The combined organic layers were washed with brine, dried over $MgSO_4$, filtered and the solvent was removed under reduced pressure. The ratio of *endo:exo* **34** was determined by GC-FID

Supplementary Table 4. Different workup procedures and their effect on the regioselectivity of the deprotonation.

entry	workup	*endo*-34 : *exo*-34
1	standard conditions (chapter 5.2)	> 99:1
2	addition of the crude mixture to 2.00 g solid KOH pellets	> 99:1
3	addition of the crude mixture to basic aluminium oxide	> 99:1
4	addition of the crude mixture to 2.00 mL neat NEt_3	> 99:1
5	addition of the crude mixture to 2.00 g solid *t*BuOK	> 99:1

4. Mechanistic Studies

4.1 Preparation of PFTB-OD and Pyridine-DBr (*d*-20b)

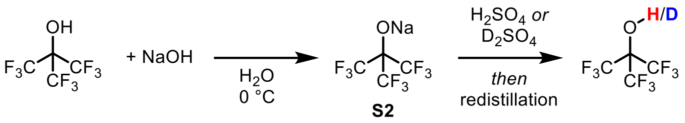

11.2 g PFTB (47.6 mmol, 1.25 eq) was carefully added to 15.0 mL of an aq. solution of 1.52 g NaOH (38.1 mmol, 1.0 eq) at 0 °C. Upon complete dissolution, the volatiles were removed under high vacuum, 500 µL PFTB was added, and the mixture stirred for 1 minute followed by removing the volatiles again under high vacuum. 9.31 g (24.0 mmol, 63%) PFTB sodium salt **S2** was obtained as a colorless powder.

2.50 g PFTB sodium salt (**S2**, 9.69 mmol, 1.0 eq) was slowly added to 1.50 mL of either concentrated H_2SO_4 or D_2SO_4 (1.86 g, 29.1 mmol, 3.0 eq) at rt. Reduced pressure was applied via a neoprene tubing leading to a –78 °C cooled flask collecting the volatile solvent. Redistillation from rt to –78 °C afforded 1.2 mL of PFTB-OH and 1.1 mL PFTB-OD, respectively. These solvents were used in the experiments determining the kinetic isotope effect (KIE, see chapter 4.2, SI).

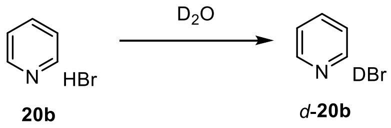

500 mg Pyridinium bromide (**20b**, 313 µmol) was dissolved in 2.00 mL of D_2O, stirred for 1 minute and the solvent was removed under reduced pressure upon gentle heating. This procedure was repeated 5 times to afford 502 mg pyridine deuterobromide (*d*-**20b**, 312 µmol, 99%) as a colorless solid.

IR (neat) $\tilde{\nu}_{max}$ = 2135, 1477, 1304, 1188, 1151, 1053,989, 925, 799, 756, 684 cm^{-1}. The spectroscopic data are in accordance with those reported in the literature. [1]

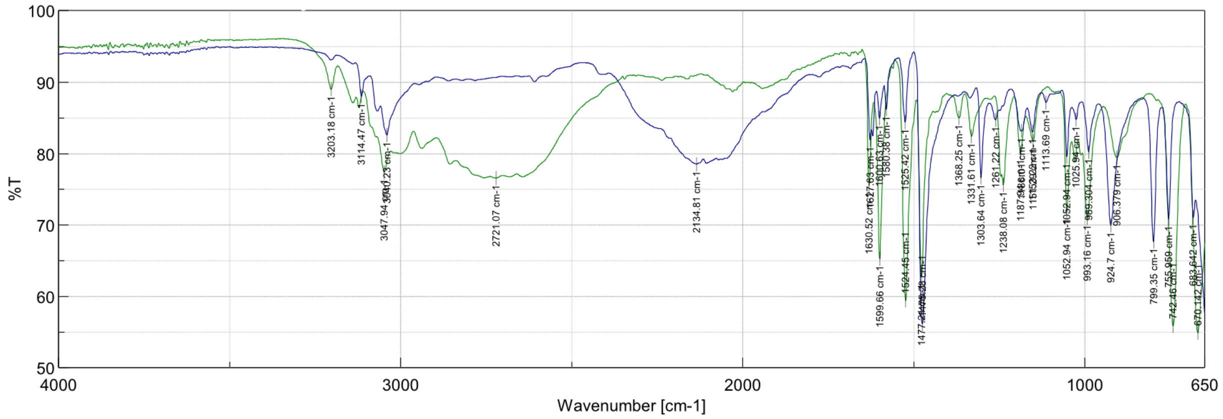

Supplementary Figure 2. Overlaid IR spectra (neat) of **20b** (green) and *d*-**20b** (blue).

4.2 Reaction of Homogeranyl Benzene 19 using Catalyst *d*-20b in Deuterated PTFB

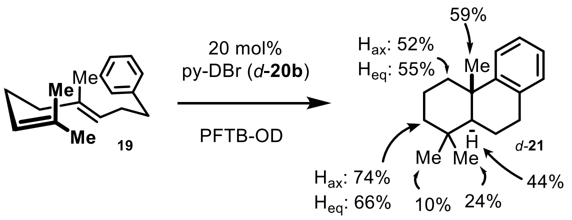

22.8 mg Homogeranyl benzene (**19**, 100 µmol, 1.0 eq) and 3.20 mg pyridine deuterobromide (*d*-**20b**, 20.0 µmol, 20 mol%) were mixed in 1.00 mL PFTB-OD and stirred at rt. The conversion from **19** to *d*-**21** was monitored by GC-MS. Percentages in *d*-**21** refer to the degree of deuteration. All unlabeled sites show <5% D incorporation.

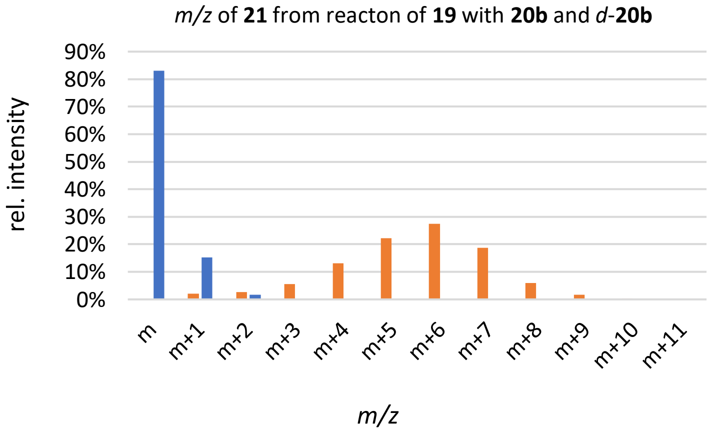

Supplementary Figure 3. Mass distribution of **21** (blue) obtained from converting **19** using unlabelled **20b** in PFTB and *d*-**21** (red) obtained from converting **19** using deuterated *d*-**20b** in PFTB-OD (m refers to the mass of the undeuterated product **21** (m/z = 228)).

4.3. Kinetic Isotope Effect (KIE) Determination

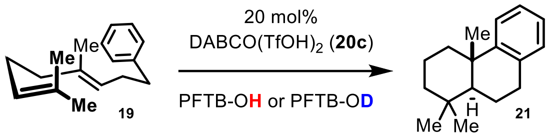

22.8 mg Homogeranyl benzene (**19**, 100 µmol, 1.0 eq) and 8.20 mg DABCO(TfOH)$_2$ (**20c**, 20.0 µmol, 20 mol%) were mixed in 1.0 mL PFTB-OH and PFTB-OD prepared above, respectively, and the reaction monitored via GC-FID.

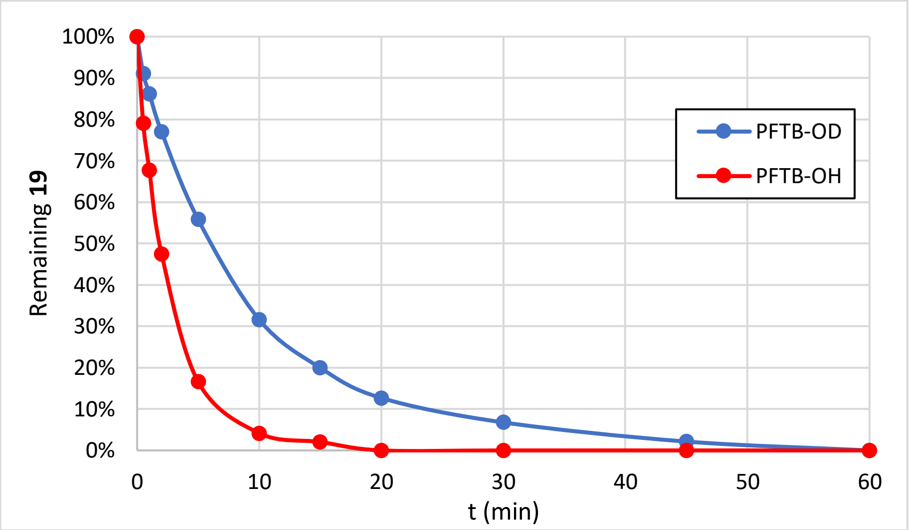

Supplementary Figure 4. Consumption of starting material **19** in PFTB-OH (**red**) or PFTB-OD (**blue**) in the presence of 20 mol% **20c**.

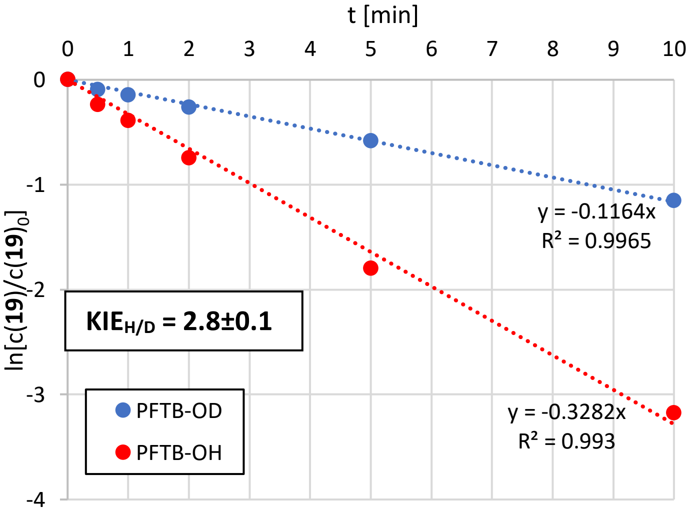

Supplementary Figure 5. Slopes for the consumption of **19** within the first 10 min. The $KIE_{H/D}$ value of 2.8±0.1 for PFTB-OH (red) and PFTB-OD (blue) was determined.

4.4. Determination of the Reaction Rate depending on the Catalysts

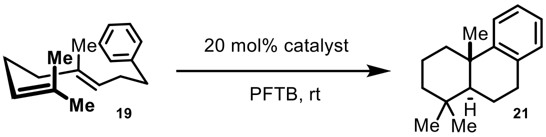

22.8 mg Homogeranyl benzene (**19**, 100 µmol, 1.0 eq) and 20 mol% catalyst (20.0 µmol) were mixed in 1.0 mL PFTB, and the reaction was monitored via GC-FID.

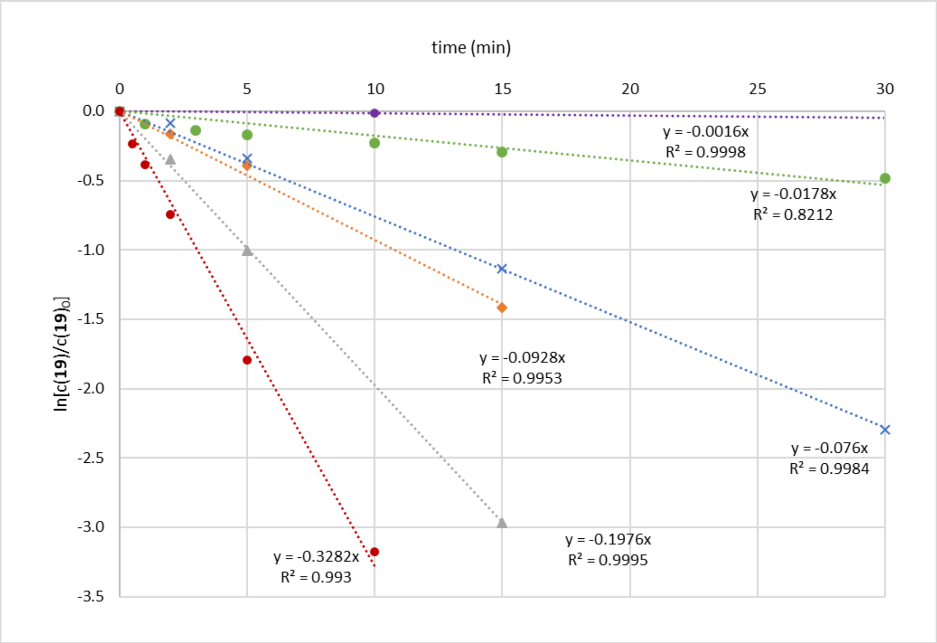

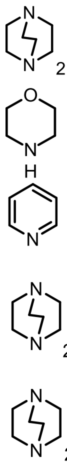

catalyst		k_{rel}
none	●	1
**20f** 2 TFA	●	11
**20d** HBr	×	48
**20b** HBr	◆	58
**20e** 2 HBr	▲	124
**20c** 2 TfOH	●	205

Supplementary Figure 6. Slopes for the consumption of **19** (k_{rel}) over time using the different amine and pyridinium salts **20b**-**20f**.

4.5. Time-Dependent Conversion of Homogeranyl Benzene (19) and Homoneryl Benzene (52a)

45.6 mg Homogeranyl benzene (**19**, 200 µmol, 1.0 eq) and 6.40 mg py-HBr (**20b**, 40.0 µmol, 20 mol%) were mixed in 2.0 mL PFTB and the reaction was monitored via GC-FID.

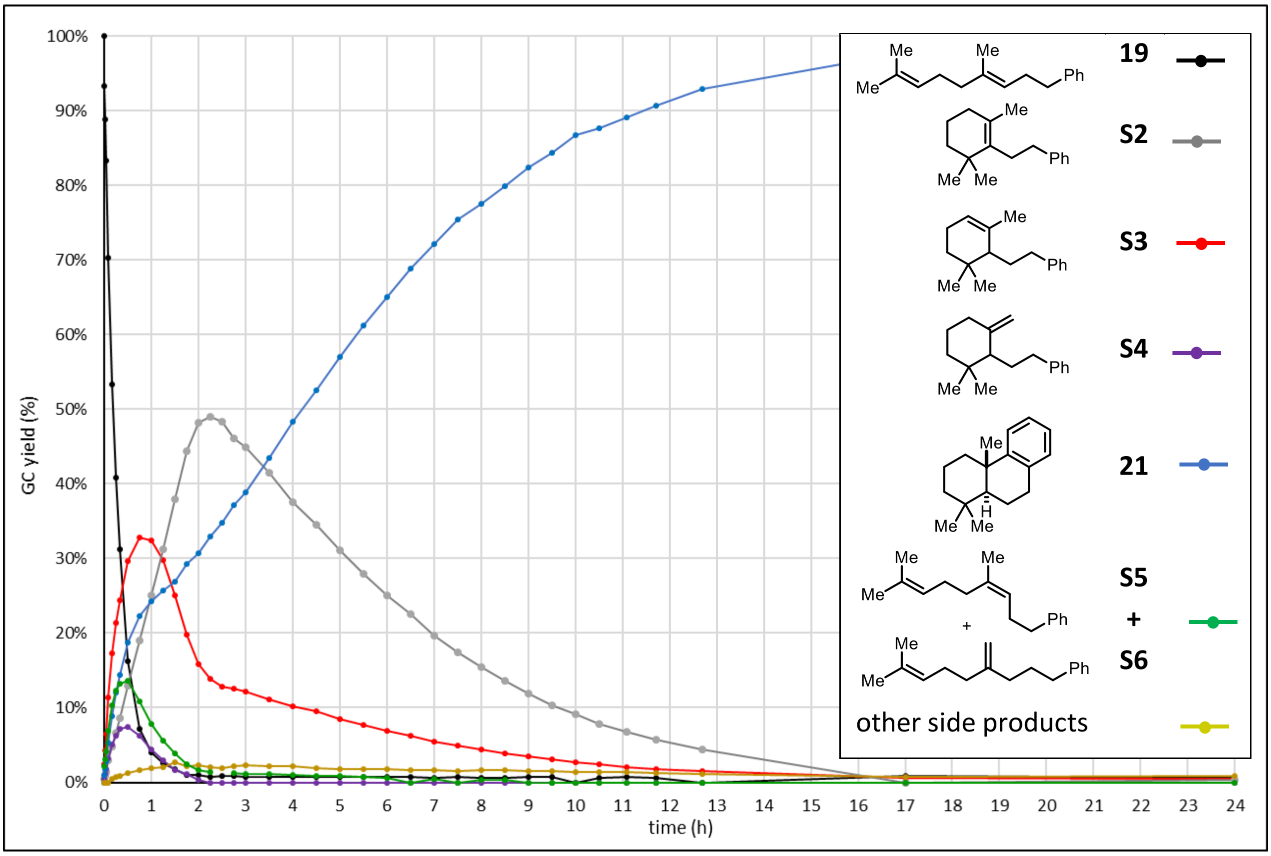

Supplementary Figure 7. Reaction kinetics for the conversion of **19** to **21** using py-HBr (**20b**) in PTFB.

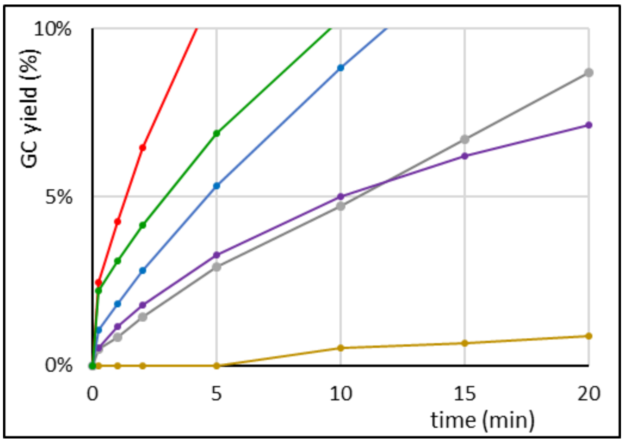

Supplementary Figure 8. Enlarged graphs of the first 20 minutes. The completely cyclized product **21** (blue line) is formed within seconds directly from homogeranyl benzene (**19**).

45.6 mg Homoneryl benzene (**52a**, 200 µmol, 1.0 eq) and 6.40 mg py-HBr (**20b**, 40.0 µmol, 20 mol%) were mixed in 2.0 mL PFTB and the reaction was monitored via GC-FID.

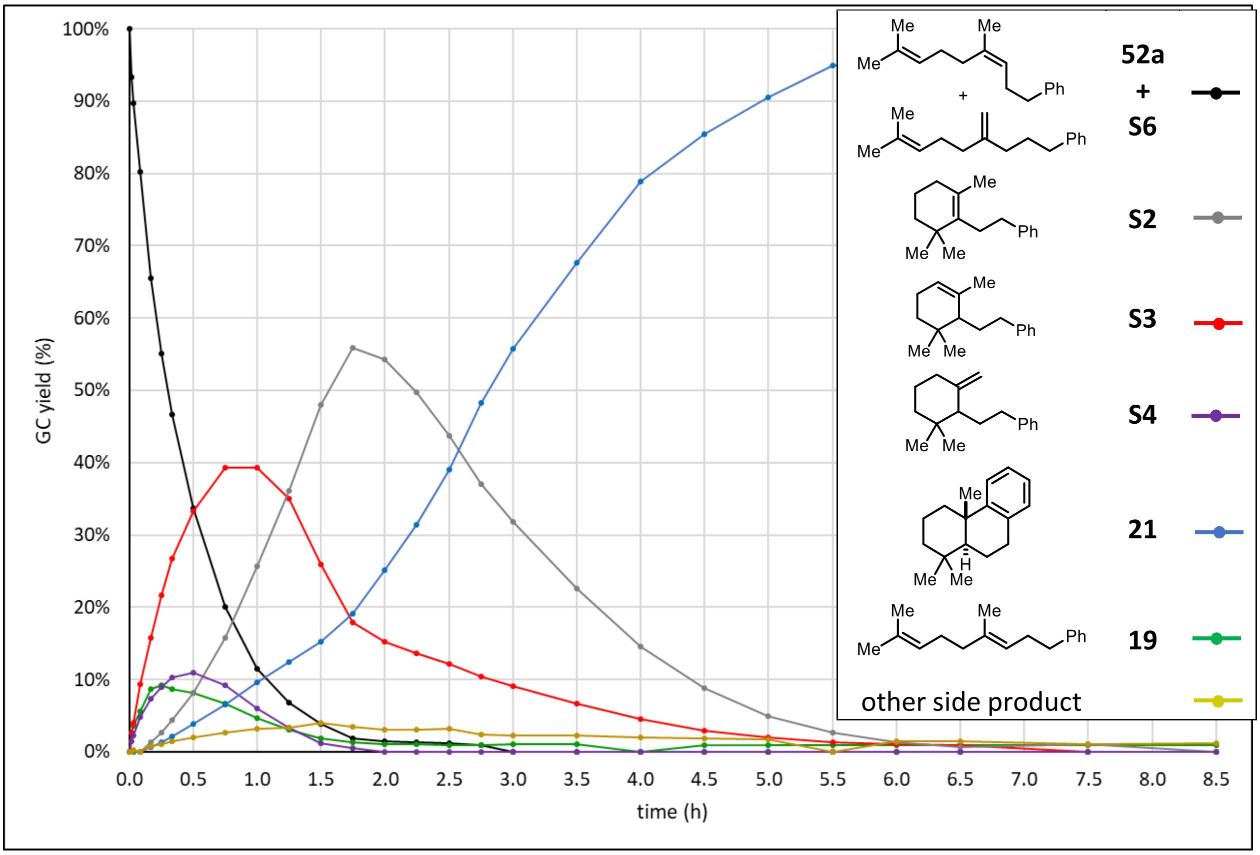

Supplementary Figure 9. Reaction kinetics for the conversion of homoneryl benzene (**52a**) to **21** using py-HBr (**20b**) in PTFB.

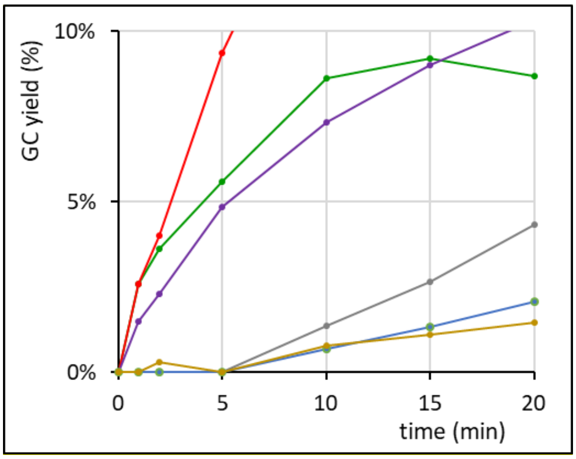

Supplementary Figure 10. Enlarged graphs of the first 20 minutes. Homoneryl benzene (**52a**, black) is converted to geranyl benzene **19** (green) followed by cyclization to the *trans*-decalin product **21** (blue).

4.6. 1D-^{1}H-NOESY Investigations on the Folding of Homogeranylbenzene (**19**) in Different Solvents

12.5 mg homogeranyl benzene (**19**, 55.0 µmol) was dissolved in 0.55 mL (0.1 M) of the respective solvent and directly subjected to 1D-^{1}H-NOESY measurements. The aromatic protons of **19** were selectively excited with 100 ± 10 Hz. On the NMR timescale, interactions of the aromatic Hs with Me_a and Me_b in **19** were only observed in PFTB or $CDCl_3$:PFTB (1:1).[2]

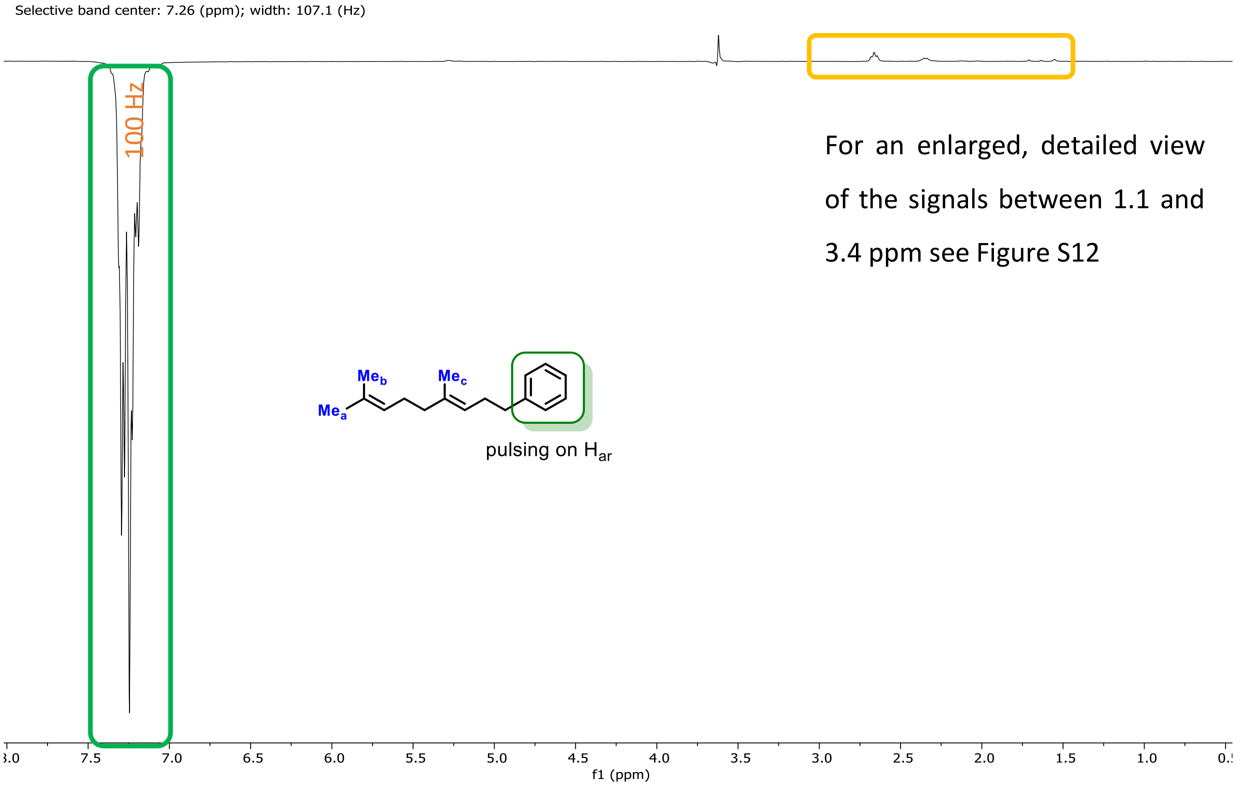

Supplementary Figure 11. 1D-^{1}H-NOESY with **19** in PFTB. Positive signals show interactions of the protons through space.

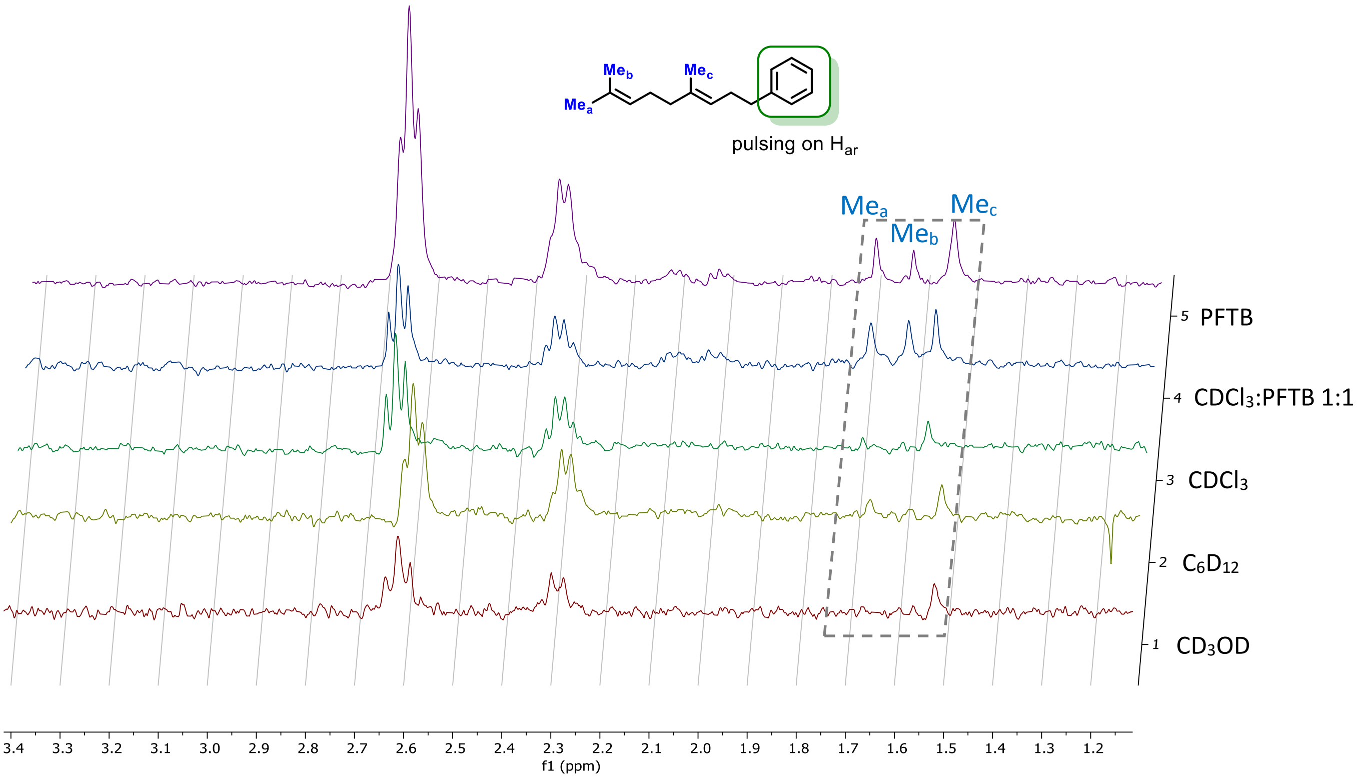

Supplementary Figure 12. Enlarged excerpts (1.1 – 3.4 ppm) from the 1D-^{1}H-NOESY spectra of 0.1 M **19** in 1) CD_3OD (red), 2) C_6D_{12} (yellow), 3) $CDCl_3$ (green), 4) $CDCl_3$/PFTB 1:1 (blue), 5) PFTB (purple).

Significant interactions of Me$_a$ and Me$_b$ and the aryl protons in **19** are only observed in samples containing PFTB. This hints at a more rigid, coiled conformation of **19** triggered by the fluorinated alcohol solvent.

4.7. Comparison of the Lewis Acidities of *t*BuOH, TFE, HFIP, PTFB, PTFB + **20b**, and PTFB + **20c** using the Gutmann-Beckett Method

Gutmann-Beckett experiments were conducted according to the procedure reported in the literature. [3] 7.40 mg Et_3PO (**S7**, 55.0 µmol) were dissolved in 0.55 mL (0.1 M) of the corresponding solvent, and, where applicable, 11.0 µmol salt was added to the solution. A ^{31}P-NMR spectrum of each sample was immediately recorded. Shifts are in relation to $(MeO)_3PO$ (2.0 M in $CDCl_3$, δ = 2.2 ppm; internal reference). [4]

The acceptor number (AN) was calculated according to equation 1 with the AN of Et_3PO (**S7**) in *n*-hexane set to zero and the AN of a 1:1 complex of Et_3PO (**S7**) and $SbCl_5$ in DCE to 100. [5]

$$\mathrm{AN} = (\delta_{\mathrm{sample}} - 41.0) \times \left(\frac{100}{86.14 - 41.0}\right) \tag{1}$$

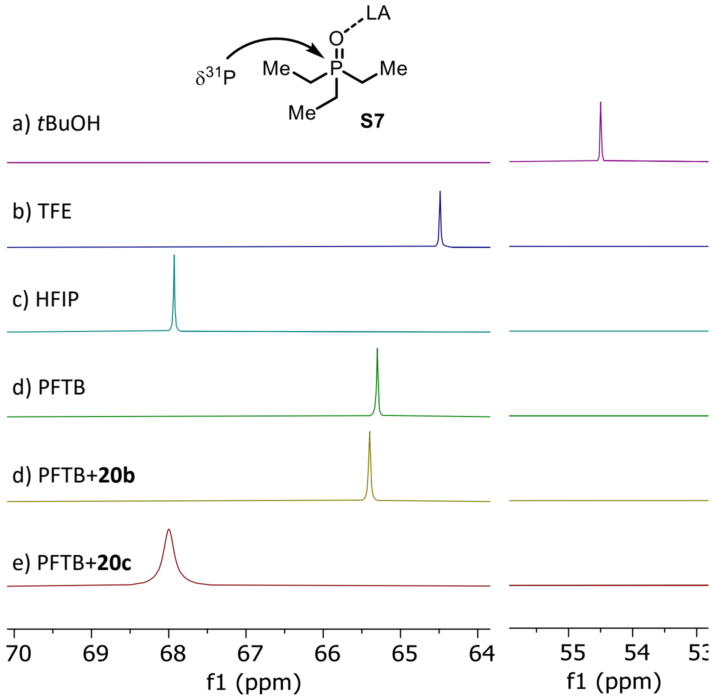

solvent/LA	δ (^{31}P, ppm)	AN
n-hexane[3a]	41.0	0.0
CH_2Cl_2[3a]	50.2	20.4
***t*BuOH**	**54.5**	**29.9**
EtOH[3a]	58.7	37.1
**TFE**	**64.5**	**52.1**
AcOH[3a]	64.9	52.9
**PFTB**	**65.3**	**53.8**
**PFTB+20b**	**65.4**	**54.1**
H_2O[3a]	65.7	54.8
$SnCl_4$[5]	-	59.0
**HFIP**	**67.9**	**59.6**
**PFTB+20c**	**68.0**	**59.8**
$TiCl_4$[5]	-	70.0
$SbCl_5$ (DCE)[3a]	86.1	100.0
TFA[3a]	88.5	105.3
TfOH[3a]	99.3	129.1

Supplementary Figure 13. ^{31}P-NMR spectra of 0.1M Et_3PO in different solvents and solvent-solute (0.02M) mixtures (left) and AN values of different Lewis acidics (right). The higher the AN the stronger the Lewis acidity.

The addition of *N*-containing salts to the Lewis acidic *F*-alcohol PTFB shifted the ^{31}P-NMR signal of **S7** downfield due to the non-covalent interactions of the Lewis basic **S7** and the Lewis acidic *F*-alcohol-salt

containing environment. The addition of DABCO(TfOH)$_2$ (**20c**) to PTFB resulted in a more significant downfield shift (ca. 3 ppm) of the ^{31}P-NMR signal and thus to an increase in the Lewis acidity of the fluorinated environment. In addition, the signal was broadened, hinting at a dynamic interaction of the PTFB-**20c** mixture and **S7**.

4.8. NMR Investigations on Non-Covalent Interactions of Homogeranyl Benzene (19) and Different Solvents

12.6 mg **19** (55.0 µmol) were dissolved in 550 µL (0.1 M) of the corresponding solvent or solvent:$CDCl_3$ mixture. A ^{13}C-NMR spectrum of each sample was recorded.

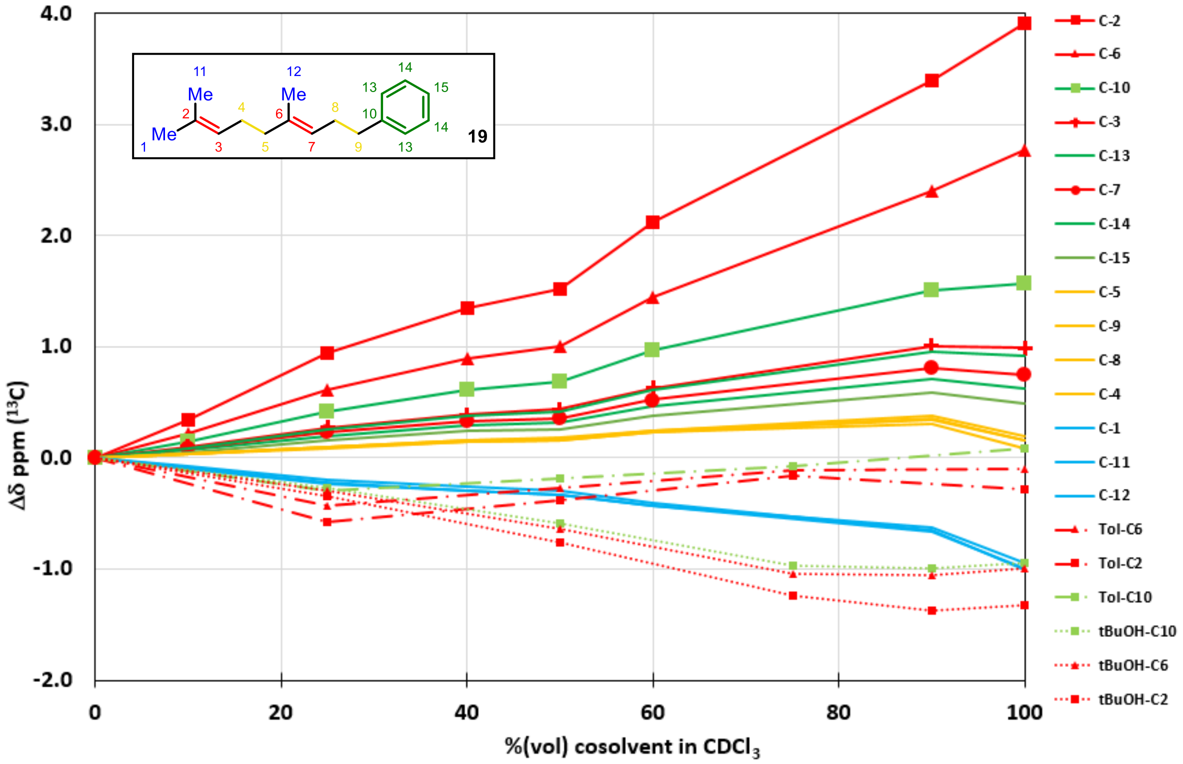

Supplementary Figure 14. ^{13}C-NMR titration curves of homogeranyl benzene (**19**) in different solvents. In the case of the experiments conducted in *t*BuOH/$CDCl_3$ (dotted lines) or toluene/$CDCl_3$ (dashed lines) only the curves for C2, C6 and C10 are shown for clarity.

4.9 Transformation of Homogeranyl Benzene (19) using Trifilic Acid (TfOH) as Catalyst

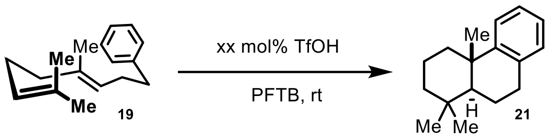

22.8 mg Homogeranyl benzene (**19**, 100 µmol, 1.0 eq) and 20 mol% catalyst (20.0 µmol) were mixed in 1.0 mL PFTB, and the reaction was monitored via GC-FID.

Supplementary Table 5. Cyclization of **19** using different loadings of TfOH catalyst.

entry	TfOH [mol%]	time	conversion	yield 21[a]	d.r.[a]
1	0.1[b]	24 h	46%	31%	> 95:5
2	1.0	8 h	> 99%	68%	> 95:5
3	5.0	4 h	> 99%	71%	> 95:5

[a]Determined by GC-FID from the crude reaction mixture using an internal standard. [b]no further conversion was observed after 24h. HFIP was distilled from a 3 Å/4 Å molecular sieve and 250 mg KOH/100 mL HFIP.

4.10 Transformation of Homogeranyl Benzene (19) performed in the presence of 2,6-di-*tert*-butylpyridine

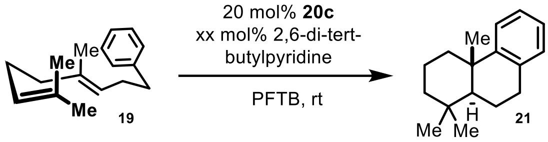

22.8 mg Homogeranyl benzene (**19**, 100 µmol, 1.0 eq) and 20 mol% catalyst (20.0 µmol) were mixed in a solution of varying amount of 2,6-di-tert-butylpyridine in 1.0 mL PFTB, and the reaction was monitored via GC-FID.

Supplementary Table 6. Cyclization of **19** with 2,6-di-tert-butylpyridine as additive.

entry	2,6-di-tert-butylpyridine [mol%]	time	conversion	yield 21[a]	d.r.[a]
1	5.0	1.5 h	> 99%	95%	> 95:5
2	10	12 h	> 99%	93%	> 95:5
3	20	24 h	-	n.d.	n.d.
4	50	24 h	-	n.d.	n.d.
5	100	24 h	-	n.d.	n.d.

[a]Determined by GC-FID from the crude reaction mixture using an internal standard. HFIP was distilled from a 3 Å/4 Å molecular sieve and 250 mg KOH/100 mL HFIP.

5. General Procedure for the Cyclization of Linear Polyenes

The corresponding linear polyene substrate was dissolved in 0.1 M PFTB at rt in a 4 mL screw cap vial with a magnetic stir bar. 20 mol% of DABCO(TfOH)$_2$ (**20c**) was added and the mixture was stirred until completion (determined by GC or TLC). Then, 2 mL $CHCl_3$ was added to terminate the reaction and the solvent was removed under reduced pressure. The crude mixture was then directly purified by column chromatography on silica gel to obtain the corresponding product.

6. Physical and Spectroscopic Data of Compounds 21 – 51

1,1,4a-Trimethyl-1,2,3,4,4a,9,10,10a-octahydrophenanthrene (**21**), prepared from homogeranyl benzene (**19**, 22.8 mg, 100 µmol); colorless oil (21.4 mg, 93.8 µmol, 94%, d.r. > 95:5); prepared from homoneryl benzene (**52a**, 22.8 mg, 100 µmol); colorless oil (20.5 mg, 89.9 µmol, 90%, d.r. > 95:5).

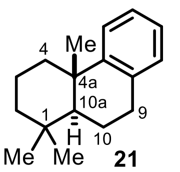

TLC: R_f = 0.60 (silica gel, *n*-hexane) [CAM]; **^{1}H NMR** (400 MHz, $CDCl_3$) δ 7.29 – 7.26 (m, 1H), 7.16 – 7.02 (m, 3H), 3.00 – 2.81 (m, 2H), 2.30 (bd, *J* = 12.9, Hz, 1H), 1.93 – 1.86 (m, 1H), 1.80 – 1.68 (m, 2H), 1.66 – 1.60 (m, 1H), 1.52 – 1.47 (m, 1H), 1.46 – 1.32 (m, 2H), 1.25 (dd, *J* = 13.5, 4.2 Hz, 1H), 1.20 (s, 3H), 0.97 (s, 3H), 0.95 (s, 3H) ppm; **^{13}C NMR** (101 MHz, $CDCl_3$) δ 150.3, 135.4, 129.1, 125.7, 125.3, 124.5, 50.44, 41.84, 38.95, 37.95, 33.62, 33.45, 30.54, 25.01, 21.79, 19.45, 19.17 ppm.

The spectroscopic data are in accordance with those reported in the literature. [6]

6-Methoxy-1,1,4a-trimethyl-1,2,3,4,4a,9,10,10a-octahydrophenanthrene (**22**), prepared from (*E*)-1-(4,8-dimethylnona-3,7-dien-1-yl)-4-methoxybenzene (34.2 mg, 132 µmol); colorless oil (30.0 mg, 116 µmol, 88%, d.r. > 95:5).

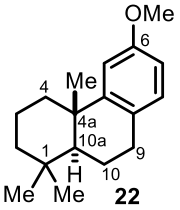

TLC: R_f = 0.43 (silica gel, *n*-hexane/EtOAc; 95:5) [CAM]; **^{1}H NMR** (300 MHz, $CDCl_3$) δ 6.96 (d, *J* = 8.4, 1H), 6.82 (d, *J* = 2.8 Hz, 1H), 6.67 (dd, *J* = 8.4, 2.7 Hz, 1H), 3.78 (s, 3H), 2.94 – 2.84 (m, 1H), 2.85 – 2.75 (m, 1H), 2.30 – 2.19 (m, 1H), 1.93 – 1.82 (m, 1H), 1.81 – 1.71 (m, 2H), 1.71 – 1.58 (m, 1H), 1.53 – 1.40 (m, 2H), 1.33 (dd, *J* = 12.3, 2.4 Hz, 1H), 1.28 – 1.21 (m, 1H), 1.20 (d, *J* = 0.8 Hz, 3H), 0.96 (s, 3H), 0.94 (s, 3H) ppm; **^{13}C NMR** (75 MHz, $CDCl_3$) δ 157.8, 151.6, 130.0, 127.6, 110.9, 110.3, 55.40, 50.47, 41.83, 38.99, 38.17, 33.64, 33.46, 29.71, 24.89, 21.81, 19.47, 19.30 ppm.

The NMR spectra contain ca. 9% of 4,4’dimethoxybibenzyl, a site product generated during starting material synthesis and which could not be separated at either of the following steps. The yield of **22** was corrected accordingly.

The spectroscopic data are in accordance with those reported in the literature. [7]

4a'-Methyl-3',4',4a',9',10',10a'-hexahydro-2'H-spiro[cyclohexane-1,1'-phenanthrene] (**23**), prepared from (*E*)-(7-cyclohexylidene-4-methylhept-3-en-1-yl)benzene (35.2 mg, 131 µmol); colorless oil (32.0 mg, 119 µmol, 91%, d.r. = 91:9).

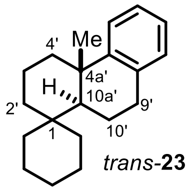

trans-**23**

TLC: R_f = 0.78 (silica gel, *n*-hexane) [CAM]; *trans isomer:* **^{1}H NMR** (300 MHz, $CDCl_3$) δ 7.30 – 7.23 (m, 1H), 7.17 – 7.08 (m, 1H), 7.08 – 6.99 (m, 2H), 3.01 – 2.75 (m, 2H), 2.29 (td, *J* = 12.0, 11.4, 5.3 Hz, 2H), 2.03 – 1.94 (m, 1H), 1.85 – 1.73 (m, 2H), 1.72 – 1.59 (m, 4H), 1.50 (m, 4H), 1.33 – 1.24 (m, 2H), 1.21 (s, 3H), 0.98 – 0.73 (m, 4H) ppm; **^{13}C NMR** (76 MHz, $CDCl_3$) δ 150.7, 135.4, 129.1, 125.7, 125.3, 124.5, 51.42, 39.41, 39.20, 38.27, 36.45, 33.62, 31.15, 28.12, 26.72, 26.24, 21.96, 21.92, 18.75, 18.70 ppm; **IR** (KBr) $\tilde{\nu}_{max}$ = 2935, 1683, 1449, 1217, 1044, 759 cm^{-1}; **HRMS** (EI) calcd. for $C_{20}H_{28}$ $[M]^+$ 268.2184, found 268.2194.

Characteristic signals for the *cis isomer*: **^{1}H NMR** (300 MHz, $CDCl_3$) δ 6.88 (t, *J* = 4.5 Hz, 1H), 2.69 (dt, *J* = 7.0, 3.1Hz, 1H), 2.56 (t, *J* = 7.5 Hz, 1H), 1.12 (s, 3H) ppm; **^{13}C NMR** (76 MHz, $CDCl_3$) δ 128.5, 128.4, 45.13, 36.29, 35.46, 31.74, 27.25, 22.80, 13.32 ppm.

1,1,4a-Trimethyl-2,3,4,4a,9,9a-hexahydro-1*H*-xanthene (**24a**), prepared from (*E*)-2-(3,7-dimethylocta-2,6-dien-1-yl)phenol (39.1 mg, 170 µmol); colorless solid (25.0 mg, 109 µmol, 64%, d.r. > 95:5).

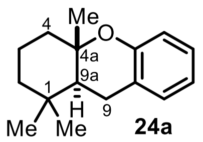

24a

TLC: R_f = 0.19 (silica gel, *n*-hexane) [CAM]; **^{1}H NMR** (500 MHz, $CDCl_3$) δ 7.11 – 7.03 (m, 2H), 6.82 (t, *J* = 7.3 Hz, 1H), 6.76 (d, *J* = 8.0 Hz, 1H), 2.71 (dd, *J* = 16.3, 5.0 Hz, 1H), 2.61 (dd, *J* = 16.4, 13.1 Hz, 1H), 2.00 – 1.94 (m, 1H), 1.71 (dd, *J* = 13.2, 5.1 Hz, 1H), 1.69 – 1.63 (m, 1H), 1.61 (d, *J* = 4.2 Hz, 1H), 1.55 (dt, *J* = 11.7, 3.0 Hz, 1H), 1.52 – 1.46 (m, 1H), 1.37 – 1.29 (m, 1H), 1.22 (s, 3H), 1.01 (s, 3H), 0.91 (s, 3H) ppm; **^{13}C NMR** (75 MHz, $CDCl_3$) δ 153.4, 129.8, 127.3, 122.8, 119.8, 117.2, 77.36, 48.25, 41.68, 40.17, 33.54, 32.25, 23.41, 20.83, 20.00, 19.95 ppm.

The spectroscopic data are in accordance with those reported in the literature. [8]

7-Methoxy-1,1,4a-trimethyl-2,3,4,4a,9,9a-hexahydro-1*H*-xanthene (**24b**), prepared from (*E*)-2-(3,7-dimethylocta-2,6-dien-1-yl)-4-methoxyphenol (28.1 mg, 108 µmol); colorless oil (24.0 mg, 92.2 µmol, 85%, d.r. = 93:7).

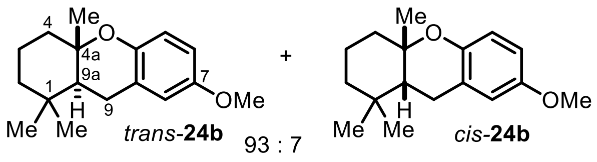

TLC: R_f = 0.61 (silica gel, *n*-hexane/EtOAc; 90:10) [CAM]; *trans isomer*: **^{1}H NMR** (300 MHz, $CDCl_3$) δ 6.71 – 6.64 (m, 2H), 6.64 – 6.61 (m, 1H), 3.75 (s, 3H), 2.69 (dd, *J* = 16.5, 5.5 Hz, 1H), 2.58 (dd, *J* = 16.5, 12.7 Hz, 1H), 1.99 – 1.91 (m, 1H), 1.70 (dd, *J* = 12.6, 5.5 Hz, 1H), 1.64 – 1.55 (m, 2H), 1.54 – 1.45 (m, 1H), 1.41 – 1.26 (m, 2H), 1.20 (s, 3H), 1.00 (s, 3H), 0.90 (s, 3H) ppm; **^{13}C NMR** (75 MHz, $CDCl_3$) δ 153.1, 147.4, 123.3, 117.6, 114.4, 113.3, 77.00, 55.84, 48.27, 41.68, 40.13, 33.49, 32.23, 23.78, 20.78, 19.94, 19.84 ppm.

Characteristic signals for the cis isomer: **^{1}H NMR** (300 MHz, $CDCl_3$) δ 3.01 (dd, *J* = 17.6, 8.1 Hz, 1H), 1.18 (s, 3H), 0.96 (s, 3H), 0.67 (s, 3H) ppm.

The spectroscopic data are in accordance with those reported in the literature. [8]

Methyl-8-hydroxy-1,1,4a-trimethyl-2,3,4,4a,9,9a-hexahydro-1*H*-xanthene-7-carboxylate (**25**), prepared from methyl (*E*)-3-(3,7-dimethylocta-2,6-dien-1-yl)-2,4-dihydroxybenzoate (23.6 mg, 77.5 µmol); colorless oil (20.1 mg, 66.0 µmol, 85%, d.r. > 95:5, 64:36 mixture of inseparable regioisomers).

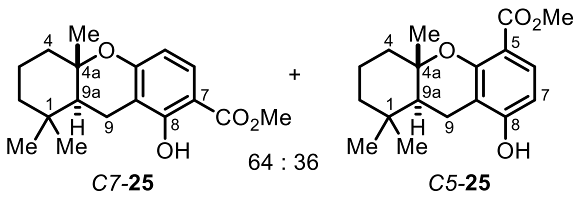

TLC: R_f = 0.35-0.42 (silica gel, *n*-hexane/EtOAc; 95:5) [CAM].

C7-isomer: **^{1}H NMR** (400 MHz, $CDCl_3$) δ 11.28 (s, 1H), 7.60 – 7.53 (m, 1H), 6.31 (d, *J* = 8.8 Hz, 1H), 3.90 (s, 3H), 2.81 (dd, *J* = 16.9, 4.8 Hz, 1H), 2.32 (ddd, *J* = 16.9, 13.3, 0.9 Hz, 1H), 2.01 – 1.94 (m, 1H), 1.75 – 1.54 (m, 4H), 1.52 – 1.39 (m, 2H), 1.38 – 1.27 (m, 2H), 1.22 (s, 3H), 1.04 (s, 3H), 0.94 (s, 3H) ppm.

C5-isomer: **^{1}H NMR** (400 MHz, $CDCl_3$) δ 10.60 (s, 1H), 7.62 – 7.52 (m, 1H), 6.31 (d, *J* = 8.8 Hz, 1H), 3.89 (s, 3H), 2.73 – 2.60 (m, 1H), 2.52 (ddd, *J* = 15.9, 13.3, 1.3 Hz, 1H), 2.01 – 1.94 (m, 1H), 1.71 – 1.55 (m, 4H), 1.53 – 1.39 (m, 2H), 1.34 – 1.29 (m, 2H), 1.22 (s, 3H), 0.99 (s, 3H), 0.89 (s, 3H) ppm.

Mixture of *C7*-**25** and *C5*-**25**: **^{13}C NMR** (101 MHz, $CDCl_3$) δ 171.0, 170.4, 161.4, 161.1, 160.0, 159.4, 131.2, 128.3, 124.0, 114.7, 110.4, 109.1, 105.1, 104.2, 104.0, 78.70, 78.48, 51.86, 51.84, 48.09, 47.39, 41.49, 41.34, 39.78, 39.74, 33.56, 33.48, 32.11, 32.02, 22.42, 20.68, 20.62, 20.06, 19.76, 19.72, 17.56 ppm; **IR**

(KBr) $\tilde{\nu}_{max}$ = 2951, 2866, 1667, 1492, 1439, 1346, 1264, 1164, 987, 758 cm^{-1}; **HRMS** (ESI+) calcd. for $C_{18}H_{25}O_4^+$ $[M+H]^+$ 305.1747, found 305.1726.

(±)-Tetrahydroactinidiolide (**26**), prepared from (*E*)-4,8-dimethylnona-3,7-dienoic acid (32.3 mg, 177 µmol); colorless wax (31.6 mg, 174 µmol, 98%; d.r. = 76:24) separable mixture of diastereomers

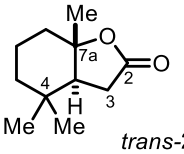

trans-**26**

trans-isomer: 24.0 mg, 132 µmol, 74%; **TLC:** R_f = 0.45 (silica gel, *n*-hexane/EtOAc; 50:50) [CAM]; **^{1}H NMR** (300 MHz, $CDCl_3$) δ 2.42 (dd, *J* = 16.3, 14.6 Hz, 1H), 2.29 (ddd, *J* = 16.3, 6.8, 0.6 Hz, 1H), 2.03 – 1.94 (m, 2H), 1.83 – 1.72 (m, 1H), 1.69 – 1.49 (m, 3H), 1.34 (d, *J* = 0.7 Hz, 3H), 1.32 – 1.21 (m, 1H), 0.96 (s, 3H), 0.93 (s, 3H) ppm; **^{13}C NMR** (75 MHz, $CDCl_3$) δ 176.6, 86.63, 55.73, 40.24, 37.34, 32.86, 32.40, 29.35, 20.86, 20.61, 19.95 ppm.

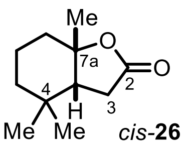

cis-**26**

cis-isomer: 7.6 mg, 41.7 µmol, 24%; **TLC:** R_f = 0.33 (silica gel, *n*-hexane/EtOAc; 50:50) [CAM]; **^{1}H NMR** (500 MHz, $CDCl_3$) δ 2.51 (dd, *J* = 17.3, 12.7 Hz, 1H), 2.43 (dd, *J* = 17.4, 8.2 Hz, 1H), 2.06 (dd, *J* = 12.8, 8.2 Hz, 1H), 1.89 – 1.83 (m, 1H), 1.64 – 1.59 (m, 2H), 1.53 (s, 3H), 1.51 – 1.35 (m, 2H), 1.33 – 1.27 (m, 1H), 1.05 (s, 3H), 0.91 (s, 3H) ppm; **^{13}C NMR** (126 MHz, $CDCl_3$) δ 175.9, 86.25, 52.09, 34.85, 33.70, 33.41, 32.34, 30.28, 28.55, 27.08, 19.10 ppm.

The spectroscopic data for the *trans*[9] and *cis*[10] isomer of **26** are in accordance with those reported in the literature.

(2,6,6-Trimethylcyclohex-2-en-1-yl)methyl benzoate (**27a**), prepared from geraniol benzoate (32.3 mg, 125 µmol); colorless oil (27.4 mg, 106 µmol, 85%; 87:13 mixture of inseparable regioisomers).

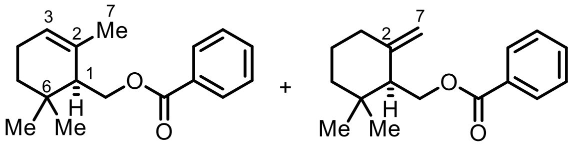

$\Delta_{2,3}$-**27a** 87 : 13 $\Delta_{2,7}$-**27a**

TLC: R_f = 0.50 (silica gel, *n*-hexane/EtOAc; 95:5) [$KMnO_4$]; $\Delta_{2,3}$-**27a**: **^{1}H NMR** (300 MHz, $CDCl_3$) δ 8.05 – 8.00 (m, 2H), 7.58 – 7.52 (m, 1H), 7.48 – 7.40 (m, 2H), 5.50 (s, 1H), 4.38 (d, *J* = 4.6 Hz, 2H), 2.06 – 1.99 (m, 2H), 1.95 (q, *J* = 3.9, 1H), 1.80 (d, *J* = 1.9 Hz, 3H), 1.64 – 1.49 (m, 1H), 1.26 (dtd, *J* = 13.2, 5.0, 1.2 Hz, 1H), 1.03 (s, 3H), 0.97 (s, 3H) ppm; **^{13}C NMR** (75 MHz, $CDCl_3$) δ 166.7, 132.9, 132.3, 130.7, 129.7, 128.5, 123.5, 64.69, 49.16, 32.32, 31.95, 27.57, 27.40, 23.11, 23.06 ppm;

Characteristic signals for $\Delta_{2,7}$-**27a**: **^{1}H NMR** (300 MHz, $CDCl_3$) δ 4.85 (s, 1H), 4.71 (s, 1H), 4.54 (dd, *J* = 11.1, 4.9 Hz, 1H), 4.11 (d, *J* = 2.4 Hz, 1H), 2.33 (dd, *J* = 9.4, 4.9 Hz, 1H), 0.95 (s, 3H); **^{13}C NMR** (75 MHz, $CDCl_3$) δ 132. 9, 129.7, 128.4, 110.2, 63.51, 52.47, 37.91, 34.51, 33.47, 28.90, 25.50, 24.00 ppm;

Mixture of $\Delta_{2,3}$-**27a** and $\Delta_{2,7}$-**27a**: **IR** (film) $\tilde{\nu}_{max}$ = 1718, 1451, 1275, 1114, 712 cm^{-1}; **HRMS** (EI) calcd. for $C_{17}H_{22}O_2^+$ [M]$^+$ 258.1620, found 258.1629.

(2,6,6-Trimethylcyclohex-2-en-1-yl)methyl 3',5'-dinitrobenzoate (**27b**), prepared from (*E*)-3,7-dimethylocta-2,6-dien-1-yl 3',5'-dinitrobenzoate (38.3 mg, 110 µmol); yellow oil (29.5 mg, 84.6 µmol, 77% as a 69:31 mixture of inseperable regioisomers) or prepared from (*Z*)-3,7-dimethylocta-2,6-dien-1-yl 3',5'-dinitrobenzoate (50.3 mg, 144 µmol); yellow oil (40.6 mg, 117 µmol, 81%; 68:32 mixture of inseparable regioisomers).

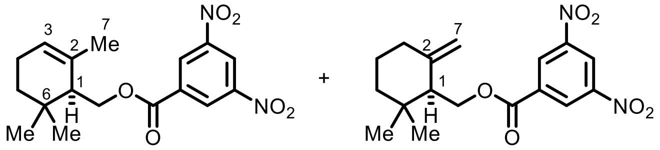

$\Delta_{2,3}$-**27b** 69 : 31 $\Delta_{2,7}$-**27b**

TLC: *R*$_f$ = 0.84 (silica gel, *n*-hexane/EtOAc; 80:20) [UV]; $\Delta_{2,3}$-**27b**: **^{1}H NMR** (500 MHz, CDCl$_3$) δ 9.23 (t, *J* = 2.1 Hz, 1H), 9.13 (d, *J* = 2.1 Hz, 2H), 5.56 (s, 1H), 4.53 (dd, *J* = 11.7, 5.7 Hz, 1H), 4.48 (dd, *J* = 11.7, 3.1 Hz, 1H), 2.09 – 2.05 (m, 2H), 1.97 (br s, 1H), 1.79 (q, *J* = 1.9 Hz, 3H), 1.60 – 1.49 (m, 1H), 1.31 (dt, *J* = 13.8, 4.6 Hz, 1H), 1.04 (s, 3H), 0.98 (s, 3H) ppm; **^{13}C NMR** (126 MHz, CDCl$_3$) δ 162.6, 148.8, 134.3, 131.3, 129.5, 124.2, 122.4, 66.35, 49.02, 32.09, 31.98, 27.71, 27.38, 23.08, 22.97 ppm.

Characteristic signals of $\Delta_{2,7}$-**27b**: **^{1}H NMR** (500 MHz, CDCl$_3$) δ 9.21 (t, *J* = 2.2 Hz, 1H), 9.12 (d, *J* = 2.2 Hz, 2H), 4.87 (s, 1H), 4.69 (s, 1H), 4.61 (d, *J* = 0.9 Hz, 1H), 4.60 (s, 1H), 2.39 (t, *J* = 7.6 Hz, 1H), 2.25 – 2.18 (m, 1H), 2.13 (dt, *J* = 13.5, 5.3 Hz, 1H), 1.64 – 1.59 (m, 2H), 1.45 – 1.34 (m, 2H), 1.04 (s, 3H), 0.97 (s, 3H) ppm; **^{13}C NMR** (126 MHz, CDCl$_3$) δ 162.6, 148.8, 146.8, 134.3, 129.6, 122.4, 110.8, 65.17, 52.46, 37.33, 34.49, 32.86, 28.70, 25.93, 23.34 ppm.

Mixture of $\Delta_{2,3}$-**27b** and $\Delta_{2,7}$-**27b**: **IR** (neat) $\tilde{\nu}_{max}$ = 2959, 2924, 1733, 1629, 1548, 1458, 1344, 1261, 1165, 1075, 800 cm^{-1}; **HRMS** (EI) calcd. for $C_{17}H_{20}N_2O_6^+$ [M]$^+$ 348.1316, found 348.1318.

1',′3-Diethyl 5-((2,6,6-trimethylcyclohex-2-en-1-yl)methyl) benzene-1',3',5'-tricarboxylate (**27c**), prepared from (*E*)-1-(3,7-dimethylocta-2,6-dien-1-yl) 3',5'-diethyl benzene-1',3',5'-tricarboxylate (28.1 mg, 69.9 µmol); colorless oil (20.4 mg, 50.7 µmol, 73%; 86:14 mixture of inseparable regioisomers).

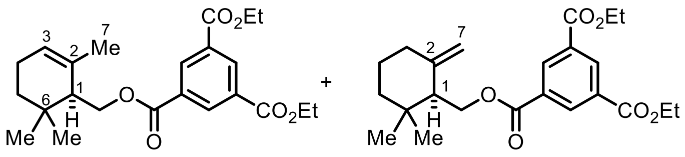

$\Delta_{2,3}$-**27c** 86:14 $\Delta_{2,7}$-**27c**

TLC: *R*$_f$ = 0.24 (silica gel, *n*-hexane/EtOAc; 95:5) [CAM]; $\Delta_{2,3}$-**27c**: **^{1}H NMR** (300 MHz, CDCl$_3$) δ 8.89 – 8.81 (m, 1H), 8.84 – 8.81 (m, 2H), 5.53 (s, 1H), 4.47 – 4.38 (m, 6H), 2.07 –

2.01 (m, 2H), 1.95 (t, J = 4.0 Hz, 1H), 1.79 (q, J = 1.6 Hz, 3H), 1.65 – 1.54 (m, 1H), 1.43 (t, J = 7.2, Hz, 6H), 1.31 – 1.24 (m, 1H), 1.04 (s, 3H), 0.97 (s, 3H) ppm; **^{13}C NMR** (75 MHz, $CDCl_3$) δ 165.2, 165.2, 134.61, 134.57, 131.9, 131.7, 131.6, 123.8, 65.20, 61.82, 49.12, 32.22, 31.98, 27.59, 27.54, 23.11, 23.05, 14.43 ppm.

Characteristic signals of $\Delta_{2,7}$-**27c**: **^{1}H NMR** (300 MHz, $CDCl_3$) δ 8.89 – 8.81 (m, 1H), 8.84 – 8.81 (m, 2H), 4.86 (s, 1H), 4.71 (s, 1H), 4.54 – 4.49 (m, 2H) 2.37 (dd, J = 8.1, 6.5 Hz, 1H), 2.28 – 2.14 (m, 1H), 1.04 (s, 3H), 0.96 (s, 3H) ppm.

Mixture of $\Delta_{2,3}$-**27c** and $\Delta_{2,7}$-**27c**: **IR** (KBr) $\tilde{\nu}_{max}$ = 2964, 1728, 1241, 1026, 741 cm^{-1}; **HRMS** (ESI$^+$) calcd. for $C_{23}H_{30}NaO_6^+$ $[M+Na]^+$ 425.1935, found 425.1903.

(2,6,6-Trimethylcyclohex-2-en-1-yl)methyl 3′,5′-dimethoxybenzoate (**27d**), prepared from (*E*)-3,7-dimethylocta-2,6-dien-1-yl 3′,5′-dimethoxybenzoate (41.0 mg, 129 µmol); colorless oil (33.2 mg, 104 µmol, 81%; 88:12 mixture of inseparable regioisomers).

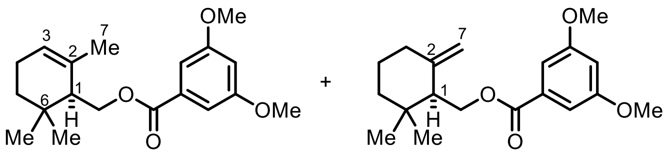

$\Delta_{2,3}$-**27d** 88:12 $\Delta_{2,7}$-**27d**

TLC: R_f = 0.36 (silica gel, n-n-hexane/EtOAc; 95:5) [CAM]; $\Delta_{2,3}$-**27d**: **^{1}H NMR** (300 MHz, $CDCl_3$) δ 7.18 (d, J = 2.4 Hz, 2H), 6.64 (t, J = 2.4 Hz, 1H), 5.50 (s, 1H), 4.36 (d, J = 4.6 Hz, 2H), 3.82 (s, 6H), 2.06 – 1.98 (m, 2H), 1.92 (t, J = 4.7 Hz, 1H), 1.79 (q, J = 1.4 Hz, 3H), 1.57 (dt, J = 16.2, 6.5 Hz, 1H), 1.30 – 1.20 (m, 1H), 1.03 (s, 3H), 0.96 (s, 3H) ppm; **^{13}C NMR** (75 MHz, $CDCl_3$) δ 166.4, 160.8, 132.6, 132.2, 123.5, 107.3, 105.7, 64.79, 55.64, 49.14, 32.27, 31.94, 27.55, 27.46, 23.09 ppm.

Characteristic signals for $\Delta_{2,7}$-**27d**: **^{1}H NMR** (300 MHz, $CDCl_3$) δ 7.17 (d, J = 2.4 Hz, 2H), 6.63 (t, J = 2.4 Hz, 1H), 4.85 (br s, 1H), 4.70 (dt, J = 1.9, 0.9 Hz, 1H), 4.51 (dd, J = 11.1, 5.1 Hz, 1H), 4.42 (dd, J = 11.0, 9.4 Hz, 1H), 3.81 (s, 6H), 2.32 (dd, J = 9.6, 5.3 Hz, 1H), 2.27 – 2.07 (m, 2H), 1.40 – 1.30 (m, 2H), 1.03 (s, 3H), 0.94 (s, 3H) ppm; **^{13}C NMR** (75 MHz, $CDCl_3$) δ 160.7, 147.3, 110.2, 107.3, 105.7, 63.68, 52.49, 37.74, 34.46, 33.31, 28.84, 25.68, 23.50 ppm; further signals could not be identified due to overlapped with the signals of the major isomer.

Mixture of $\Delta_{2,3}$-**27d** and $\Delta_{2,7}$-**27d**: **IR** (KBr) $\tilde{\nu}_{max}$ = 1718, 1598, 1463, 1302, 1206, 1157, 1050, 767 cm^{-1}; **HRMS** (ESI+) calcd. for $C_{19}H_{26}NaO_4^+$ $[M+Na]^+$ 341.1723, found 341.1723.

(2,6,6-Trimethylcyclohex-2-en-1-yl)methyl isoxazole-5-carboxylate (**28**), prepared from geranyl isoxazole-5-carboxylate (39.9 mg, 160 µmol); colorless oil (39.7 mg, 159 µmol, 99%; 83:17 mixture of inseperable regioisomers).

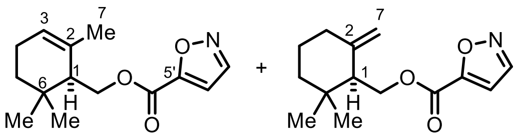

$\Delta_{2,3}$-**28** 83 : 17 $\Delta_{2,7}$-**28**

TLC: R_f = 0.35 (silica gel, *n*-hexane/EtOAc; 95:5); $\Delta_{2,3}$-**28**: **^{1}H NMR** (300 MHz, $CDCl_3$) δ 8.36 (d, *J* = 1.8 Hz, 1H), 6.91 (d, *J* = 1.8 Hz, 1H), 5.53 – 5.46 (m, 1H), 4.47 (dd, *J* = 11.6, 5.8 Hz, 1H), 4.36 (dd, *J* = 11.7, 3.5 Hz, 1H), 2.03 – 1.96 (m, 2H), 1.93 – 1.88 (m, 1H), 1.77 (q, *J* = 1.7 Hz, 3H), 1.58 – 1.49 (m, 1H), 1.30 – 1.20 (m, 1H), 1.00 (s, 3H), 0.94 (s, 3H) ppm; **^{13}C NMR** (75 MHz, $CDCl_3$) δ 160.3, 156.7, 150.7, 131.5, 124.1, 108.7, 65.88, 48.96, 31.99, 31.89, 27.39, 27.37, 23.05, 22.97 ppm.

Characteristic signals of $\Delta_{2,7}$-**28**: **^{1}H NMR** (300 MHz, $CDCl_3$) δ 8.34 (d, *J* = 1.8 Hz, 1H), 6.89 (d, *J* = 1.8 Hz, 1H), 4.82 (d, *J* = 1.6 Hz, 1H), 4.64 (br s, 1H), 4.54 (d, *J* = 3.3 Hz, 1H), 4.52 (d, *J* = 0.7 Hz, 1H), 2.31 (dd, *J* = 8.6, 5.9 Hz, 1H), 1.01 (s, 3H), 0.92 (s, 3H); **^{13}C NMR** (75 MHz, $CDCl_3$) δ 160.2, 158.3, 150.9, 146.5, 110.6, 109.7, 64.78, 52.28, 37.69, 34.47, 33.20, 28.76, 25.54, 23.36 ppm; further signals could not be identified due to overlapped with the signals of the major isomer.

Mixture of $\Delta_{2,3}$-**28** and $\Delta_{2,7}$-**28**: **IR** (neat) $\tilde{\nu}_{max}$ = 2962, 1736, 1280, 1204, 1143, 769 cm^{-1}; **HRMS** (ESI+) calcd. for $C_{14}H_{19}NNaO_3^+$ $[M+Na]^+$ 272.1257, found 272.1255.

(2,6,6-Trimethylcyclohex-2-en-1-yl)methyl thiophene-2'-carboxylate (**29**), prepared from geranyl thiophene-2-carboxylate (34.6 mg, 131 µmol); slightly yellow oil (31.6 mg, 120 µmol, 91%; single regioisomer).

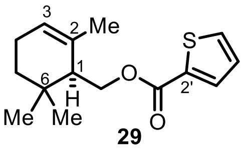

29

TLC: R_f = 0.60 (silica gel, *n*-hexane/EtOAc; 95:5); **^{1}H NMR** (400 MHz, $CDCl_3$) δ 7.78 (dd, *J* = 3.7, 1.3 Hz, 1H), 7.54 (dd, *J* = 5.0, 1.3 Hz, 1H), 7.09 (dd, *J* = 5.0, 3.7 Hz, 1H), 5.49 (br, 1H), 4.37 (dd, *J* = 11.6, 5.6 Hz, 1H), 4.32 (dd, *J* = 11.6, 3.6 Hz, 1H), 2.01 (br, 2H), 1.90 (t, *J* = 4.5 Hz, 1H), 1.78 (q, *J* = 1.9 Hz, 3H), 1.70 – 1.50 (m, 2H), 1.29 – 1.17 (m, 1H), 1.02 (s, 3H), 0.95 (s, 3H) ppm; **^{13}C NMR** (101 MHz, $CDCl_3$) δ 162.3, 134.4, 133.3, 132.3, 132.1, 127.9, 123.6, 64.81, 49.11, 32.22, 31.91, 27.55, 27.44, 23.12, 23.06 ppm; **IR** (neat) $\tilde{\nu}_{max}$ = 2963, 1710, 1419, 1258, 1096, 751, 718 cm^{-1}; **HRMS** (ESI+) calcd. for $C_{15}H_{20}NaO_2S^+$ $[M+Na]^+$ 287.1076, found 287.1072.

(±)-Cyclogeranylacetat (**30**), prepared from geranyl acetate (18.9 mg, 96.3 µmol); clear oil (16.6 mg, 84.6 µmol, 88%; single regioisomer).

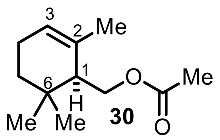

TLC: R_f = 0.51 (silica gel, *n*-hexane/EtOAc; 95:5) [CAM]; **^{1}H NMR** (300 MHz, $CDCl_3$) δ 5.44 (br, 1H), 4.16 (dd, *J* = 11.7, 5.8 Hz, 1H), 4.05 (dd, *J* = 11.7, 3.7 Hz, 1H), 2.02 (s, 3H), 1.99 – 1.93 (m, 2H), 1.83 – 1.74 (m, 1H), 1.72 (q, *J* = 1.9 Hz, 3H), 1.46 (dt, *J* = 13.3, 8.1 Hz, 1H), 1.23 – 1.17 (m, 1H), 0.93 (s, 3H), 0.90 (s, 3H) ppm; **^{13}C NMR** (75 MHz, $CDCl_3$) δ 171.3, 132.4, 123.3, 64.44, 48.90, 32.14, 31.87, 27.46, 27.13, 23.08, 23.01, 21.26 ppm.

The spectroscopic data are in accordance with those reported in the literature. [11]

(2,6,6-Trimethylcyclohex-2-en-1-yl)methyl ferrocene-1'-carboxylate (**31**), prepared from geranyl ferrocene-1'-carboxylate (25.1 mg, 68.5 µmol); deep orange oil (17.8 mg, 48.6 µmol, 71%; single regioisomer).

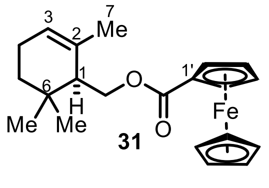

TLC: R_f = 0.53 (silica gel, *n*-hexane/EtOAc, 90:10); **^{1}H NMR** (400 MHz, $CDCl_3$) δ 5.52 (s, 1H), 4.77 (dt, *J* = 3.4, 1.8 Hz, 2H), 4.38 (t, *J* = 2.0 Hz, 2H), 4.31 (dd, *J* = 11.7, 5.4 Hz, 1H), 4.23 (dd, *J* = 11.7, 3.3 Hz, 1H), 4.19 (s, 5H), 2.08 – 2.03 (m, 2H), 1.86 (s, 1H), 1.80 (q, *J* = 1.9 Hz, 3H), 1.63 (dt, *J* = 13.3, 8.3 Hz, 1H), 1.27 (dt, *J* = 12.5, 4.3 Hz, 1H), 1.06 (s, 3H), 0.97 (s, 3H) ppm; **^{13}C NMR (**101 MHz, $CDCl_3$) δ 171.9, 132.3, 123.4, 71.77, 71.29, 71.27, 70.24, 70.18, 69.82, 63.84, 49.22, 32.50, 31.96, 27.73, 27.55, 23.15, 23.11 ppm; **IR** (neat) $\tilde{\nu}_{max}$ = 2958, 1711, 1460, 1380, 1273, 1135, 821 cm^{-1}; **Anal.** calcd. for $C_{21}H_{26}FeO_2$: C: 68.9, H: 7.16, Fe: 15.3; found: C: 68.9, H: 7.44, Fe: 15.6.

2-(((2,6,6-Trimethylcyclohex-2-en-1-yl)methoxy)methyl)oxirane (**32**), prepared from 2-geranyl methyloxirane ether (29.9 mg, 142 µmol); colorless oil (20.0 mg, 95.1 µmol, 67%; d.r = 50:50; single regioisomer).

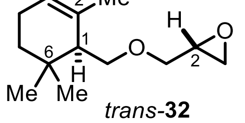

trans-**32**

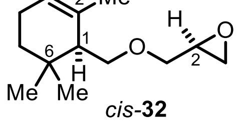

cis-**32**

TLC: R_f = 0.29 (silica gel, *n*-hexane/EtOAc, 90:10); mixture of diastereoisomers: **^{1}H NMR** (400 MHz, $CDCl_3$) δ 5.42 (tt, *J* = 3.5, 1.5 Hz, 2H), 3.68 (dd, *J* = 11.6, 3.1 Hz, 1H), 3.67 (dd, *J* = 11.6, 3.1 Hz, 1H), 3.57 (dd, *J* = 10.0, 5.5 Hz, 1H), 3.53 – 3.46 (m, 2H), 3.42 (dd, *J* = 10.0, 3.4 Hz, 1H), 3.37 (dd, *J* = 11.6, 3.0, 1H), 3.36 (dd, *J* = 11.6, 3.2 Hz, 1H), 3.17 – 3.11 (m, 2H), 2.78 (ddd, *J* = 5.1, 4.1, 1.7 Hz, 2H), 2.60 (ddd, *J* = 5.2, 4.3, 2.7 Hz, 2H), 1.96 (br, 4H), 1.74 (q, *J* = 1.9 Hz, 6H), 1.47 (dtd, *J* = 13.2, 8.1, 2.3 Hz, 2H), 1.18 (dt, *J* = 13.1, 4.6 Hz, 2H), 1.02 – 0.96 (m, 2H), 0.95 (d, *J* = 1.1 Hz, 6H), 0.90 (s, 6H) ppm; **^{13}C NMR** (101 MHz, $CDCl_3$) δ 133.5, 122.6, 71.98, 71.93, 71.68, 71.46, 51.05, 50.07, 44.51, 44.45, 32.54, 31.87, 27.61, 27.20, 27.14, 23.26, 23.12 ppm; some signals of the two

diastereomers overlap; **IR** (KBr) $\tilde{\nu}_{max}$ = 2923, 1716, 1455, 1109, 759 cm^{-1}; **HRMS** (EI) calcd. for $C_{13}H_{22}O_2^+$ $[M]^+$ 210.1614, found 210.1633.

1'-((Methoxymethoxy)methyl)-2,6,6-trimethylcyclohex-2-ene (**33**), prepared from geranyl methoxymethyl ether (18.9 mg, 95.3 µmol); colorless oil (10.6 mg, 53.5 µmol, 56%, single regioisomer).

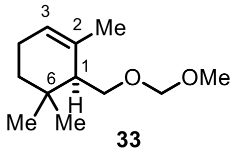

33

TLC: R_f = 0.53 (silica gel, *n*-hexane/EtOAc; 95:5); **^{1}H NMR** (500 MHz, $CDCl_3$) δ 5.44 (s, 1H), 4.61 (d, *J* = 6.6 Hz, 1H), 4.58 (d, *J* = 6.6 Hz, 1H), 3.59 (dd, *J* = 10.2, 5.2 Hz, 1H), 3.53 (dd, *J* = 10.3, 3.4 Hz, 1H), 3.37 (s, 3H), 1.97 (br, 2H), 1.74 (q, *J* = 1.9 Hz, 3H), 1.73 – 1.68 (m, 1H), 1.56 – 1.45 (m, 1H), 1.18 (dtd, *J* = 13.3, 5.0, 1.2 Hz, 1H), 0.97 (s, 3H), 0.91 (s, 3H) ppm; **^{13}C NMR** (126 MHz, $CDCl_3$) δ 133.4, 122.6, 96.74, 68.08, 55.53, 49.96, 32.59, 31.89, 27.70, 27.20, 23.16, 23.10 ppm; **IR** (KBr) $\tilde{\nu}_{max}$ = 2927, 1715, 1457, 1378, 1037, 756 cm^{-1}; **HRMS** (EI) calcd. for $C_{12}H_{22}O_2^+$ $[M]^+$ 198.1614, found 198.1626.

1-((Allyloxy)methyl)-2,6,6-trimethylcyclohex-2-ene (**34**), prepared from geranyl allyl ether (17.5 mg, 90.1 µmol); colorless oil (17.5 mg, 90.1 µmol, quant., single regioisomer).

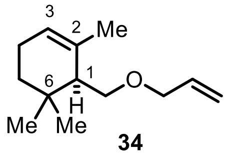

34

TLC: R_f = 0.58 (silica gel, *n*-hexane); **^{1}H NMR** (500 MHz, $CDCl_3$) δ 5.91 (ddt, *J* = 17.2, 10.8, 5.5 Hz, 1H), 5.42 (dt, *J* = 3.9, 2.2 Hz, 1H), 5.26 (dq, *J* = 17.2, 1.8 Hz, 1H), 5.16 (dq, *J* = 10.4, 1.5 Hz, 1H), 3.94 (dd, *J* = 5.5, 1.5 Hz, 2H), 3.47 (dd, *J* = 9.9, 5.5 Hz, 1H), 3.38 (dd, *J* = 10.0, 3.6 Hz, 1H), 1.96 (tdt, *J* = 7.0, 3.8, 2.0 Hz, 2H), 1.74 (d, *J* = 2.0 Hz, 3H), 1.70 – 1.62 (m, 1H), 1.49 (dt, *J* = 13.1, 8.1 Hz, 1H), 1.17 (dt, *J* = 13.2, 4.7 Hz, 1H), 0.95 (s, 3H), 0.90 (s, 3H) ppm; **^{13}C NMR** (75 MHz, $CDCl_3$) δ 135.3, 133.8, 122.4, 116.7, 71.94, 70.79, 50.10, 32.56, 31.89, 27.63, 27.18, 23.29, 23.14 ppm; **IR** (neat) $\tilde{\nu}_{max}$ = 2959, 2925, 1453, 1383, 1102, 921 cm^{-1}; **HRMS** (EI) calcd. for $C_{13}H_{22}O^+$ $[M]^+$ 194.1665, found 194.1664.

2,6,6-Trimethyl-1-((prop-2'-yn-1'-yloxy)methyl)cyclohex-2-ene (**35**), prepared from geranyl propargyl ether (20.5 mg, 107 µmol); colorless oil (20.3 mg, 106 µmol, 99%, 94:6 mixture of inseperable regioisomers).

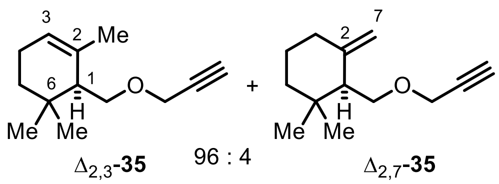

$\Delta_{2,3}$-**35** 96 : 4 $\Delta_{2,7}$-**35**

TLC: R_f = 0.63 (silica gel, *n*-hexane/EtOAc; 95:5); $\Delta_{2,3}$-**35**: **^{1}H NMR** (400 MHz, $CDCl_3$) δ 5.44 (s, 1H), 4.11 (d, *J* = 2.4 Hz, 2H), 3.58 (dd, *J* = 9.8, 5.3 Hz, 1H), 3.50 (dd, *J* = 9.8, 3.2 Hz, 1H), 2.40 (t, *J* = 2.4 Hz, 1H), 1.97 (br, 2H), 1.74 (q, *J* = 1.9 Hz, 3H), 1.54 – 1.44 (m, 2H), 1.18 (dtd, *J* = 13.0, 5.0, 1.2 Hz, 1H), 0.96 (s, 3H), 0.90 (s, 3H) ppm; **^{13}C NMR** (75 MHz, $CDCl_3$) δ 133.4, 122.7, 80.19, 74.23, 70.29, 58.08, 49.94, 32.70, 31.96, 27.69, 27.12, 23.14, 23.10 ppm.

Characteristic signals for $\Delta_{2,7}$-**35**: **^{1}H NMR** (400 MHz, $CDCl_3$) δ 4.85 (dq, *J* = 1.9, 1.0 Hz, 1H), 4.69 (br s, 1H), 0.99 (s, 3H), 0.84 (s, 3H) ppm.

Mixture of $\Delta_{2,3}$-**35** and $\Delta_{2,7}$-**35**: **IR** (neat) $\tilde{\nu}_{max}$ = 2956, 2914, 1452, 1364, 1096, 663 cm^{-1}; **HRMS** (EI) calcd. for $C_{13}H_{20}O^+$ $[M]^+$ 192.1509, found 192.1511.

1-((But-2'-en-1'-yloxy)methyl)-2,6,6-trimethylcyclohex-2-ene (**36**), prepared from geranyl but-2-en ether (31.5 mg, 151 µmol, *E*/*Z* = 87:13); colorless oil (31.5 mg, 151 µmol, *quant*., 86:14 inseperable mixture of (*E*/*Z*)-isomers).

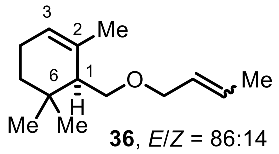

36, *E*/*Z* = 86:14

TLC: R_f = 0.38 (silica gel, *n*-hexane/EtOAc; 95:5); *E*-isomer: **^{1}H NMR** (300 MHz, $CDCl_3$) δ 5.77 – 5.63 (m, 1H), 5.63 – 5.51 (m, 1H), 5.40 (br, 1H), 3.88 – 3.84 (m, 2H), 3.43 (dd, *J* = 10.0, 5.6 Hz, 1H), 3.34 (dd, *J* = 10.0, 3.6 Hz, 1H), 1.96 (br, 2H), 1.74 – 1.69 (m, 6H), 1.58 – 1.42 (m, 1H), 1.21 – 1.11 (m, 1H), 1.08 – 0.99 (m, 1H), 0.94 (s, 3H), 0.90 (s, 3H) ppm; **^{13}C NMR** (75 MHz, $CDCl_3$) δ 133.9, 129.0, 128.1, 122.2, 71.61, 70.58, 50.11, 32.56, 31.90, 27.60, 27.15, 23.29, 23.15, 17.91 ppm.

Characteristic signal of the *Z*-isomer: **^{1}H NMR** (300 MHz, $CDCl_3$) δ 4.00 (dt, *J* = 6.2, 1.0 Hz, 2H) ppm; **^{13}C NMR** (75 MHz, $CDCl_3$) δ 127.5, 70.72, 66.18 ppm.

Mixture of *E*- and *Z*-**36**: **IR** (neat) $\tilde{\nu}_{max}$ = 2957, 1452, 1365, 1102, 966 cm^{-1}; **HRMS** (EI) calcd. for $C_{14}H_{24}O^+$ $[M]^+$ 208.1822, found 208.1811.

(((2,6,6-Trimethylcyclohex-2-en-1-yl)methoxy)methyl)benzene (**37**), prepared from geranyl benzyl ether (35.2 mg, 144 µmol); colorless oil (35.2 mg, 144 µmol, *quant.*, single regioisomer).

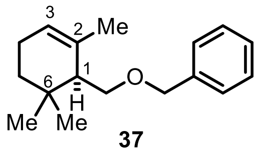

37

TLC: R_f = 0.33 (silica gel, *n*-hexane/EtOAc; 95:5); **^{1}H NMR** (300 MHz, $CDCl_3$) δ 7.41 – 7.25 (m, 5H), 5.43 (dt, *J* = 3.6, 2.0 Hz, 1H), 4.48 (s, 2H), 3.52 (dd, *J* = 9.9, 5.4 Hz, 1H), 3.44 (dd, *J* = 9.9, 3.5 Hz, 1H), 1.97 (br, 2H), 1.74 (q, *J* = 1.9 Hz, 3H), 1.64 – 1.53 (m, 1H), 1.53 – 1.43 (dt, *J* =13.1, 7.4 Hz, 1H), 1.19 (dtd, *J* = 13.2, 5.4, 1.5 Hz, 1H), 0.97 (s, 3H), 0.91 (s, 3H) ppm; **^{13}C NMR** (75 MHz, $CDCl_3$) δ 138.9, 133.7, 128.4, 127.7, 127.5, 122.4, 73.10, 70.93, 50.17, 32.58, 31.93, 27.63, 27.23, 23.28, 23.15 ppm; **IR** (neat) $\tilde{\nu}_{max}$ = 2955, 2868, 1454, 1363, 1101, 733, 697 cm^{-1}; **HRMS** (EI) calcd. for $C_{17}H_{24}O^+$ $[M]^+$ 244.1822, found 244.1815.

(2,6,6-trimethylcyclohex-2-en-1-yl)methyl 5'-((3a'*S*,4'*S*,6a'*R*)-2'-oxohexahydro-1'*H*-thieno[3',4'-d]imidazol-4'-yl)pentanoate (**39**), prepared from geranyl biotinate (39.4 mg, 104 µmol); colorless semisolid (28.1 mg, 73.8 µmol, 71%, d.r. = 50:50 (epimers); 85:15 mixture of inseperable regioisomers).

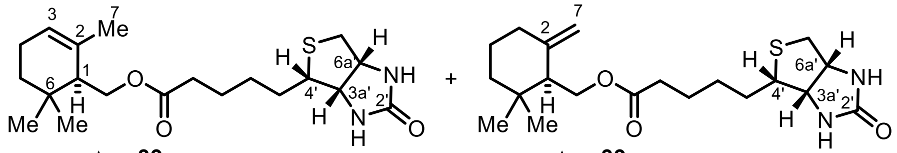

$\Delta_{2,3}$-**39** 85 : 15 $\Delta_{2,7}$-**39**

TLC: R_f = 0.36 (silica gel, EtOAc/MeOH; 90:10); diastereomeric mixture of $\Delta_{2,3}$-**39**: **^{1}H NMR** (400 MHz, $CDCl_3$) δ 5.67 (s, 1H), 5.44 (s, 1H), 5.36 (s, 1H), 4.51 (dd, *J* = 7.8, 5.0 Hz, 1H), 4.31 (dd, *J* = 7.0, 5.3 Hz, 1H), 4.17 (m, 1H), 4.05 (dd, *J* = 11.7, 3.5 Hz, 1H), 3.15 (br, 1H), 2.91 (dd, *J* = 12.9, 5.0 Hz, 1H), 2.74 (d, *J* = 12.8 Hz, 1H), 2.31 (t, *J* = 7.5 Hz, 2H), 1.97 (br, 2H), 1.79 – 1.76 (m, 1H), 1.72 (q, *J* = 1.9 Hz, 3H), 1.70 – 1.59 (m, 4H), 1.48 – 1.40 (m, 3H), 1.19 (dt, *J* = 12.9, 4.9 Hz, 1H), 0.93 (s, 3H), 0.91 (s, 3H) ppm; **^{13}C NMR** (101 MHz, $CDCl_3$) δ 173.7, 163.7, 132.33, 132.31, 123.38, 123.36, 64.27, 64.24, 62.12, 60.30, 55.55, 48.97, 40.70, 34.28, 32.19, 31.89, 28.56, 28.51, 27.51, 27.28, 27.26, 24.87, 23.12, 23.04 ppm.

Characteristic signal of $\Delta_{2,7}$-**39**: **^{1}H NMR** (400 MHz, $CDCl_3$) δ 4.81 (s, 1H), 4.58 (d, *J* = 2.0 Hz, 1H), 4.25 – 4.21 (m, 1H), 2.15 (dt, *J* = 8.9, 4.8 Hz, 2H), 0.98 (s, 3H), 0.86 (s, 3H) ppm.

Mixture of $\Delta_{2,3}$-**39** and $\Delta_{2,7}$-**39**: **IR** (neat) $\tilde{\nu}_{max}$ = 3215, 2921, 1733, 1698, 1472, 1168, 731 cm^{-1}; **HRMS** (ESI+) calcd. for $C_{20}H_{33}N_2O_3S^+$ $[M+H]^+$ 381.2206, found 381.2210.

(2,6,6-Trimethylcyclohex-2-en-1-yl)methyl 2''-(-3a',5'-dimethyl-6',8'-dioxooctahydro-1'*H*-1',4'-methanoinden-1'-yl)propanoate (**40**), prepared from geranyl santonate (41.4 mg, 103 µmol); colorless oil (27.3 mg, 68.0 µmol, 66%, 92:8 mixture of inseperable regioisomers, d.r = 55:45 [*C1*-epimer]).

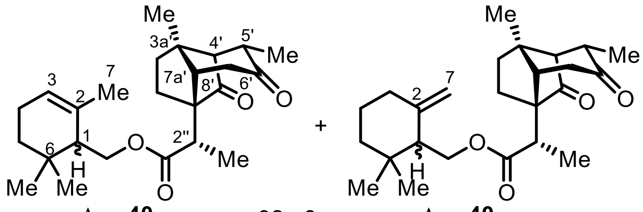

$\Delta_{2,3}$-**40** 92 : 8 $\Delta_{2,3}$-**40**

Major *C1*-epimer of the $\Delta_{2,3}$-isomer: **TLC:** R_f = 0.26 (silica gel, *n*-hexane/EtOAc; 80:20); **^{1}H NMR** (400 MHz, $CDCl_3$) δ 5.45 (s, 1H), 4.23 (dd, *J* = 11.8, 5.7 Hz, 1H), 3.96 (dd, *J* = 11.7, 2.9 Hz, 1H), 2.78 (q, *J* = 7.2 Hz, 1H), 2.67 (dd, *J* = 7.1, 3.9 Hz, 1H), 2.63 (dd, *J* = 18.1, 2.3 Hz, 1H), 2.48 (dd, *J* = 18.1, 4.9 Hz, 1H), 2.18 (td, *J* = 12.4, 5.6 Hz, 1H), 2.08 (t, *J* = 3.6 Hz, 1H), 2.02 (br, 1H), 1.97 (bt, 2H), 1.71 (q, *J* = 1.9 Hz, 3H), 1.62 (dd, *J* = 9.4, 5.7 Hz, 1H), 1.55 – 1.43 (m, 2H), 1.33 (s, 3H), 1.32 (d, *J* = 7.2 Hz, 3H), 1.15 (d, *J* = 6.7 Hz, 3H), 0.94 (s, 3H), 0.91 (s, 3H) ppm; **^{13}C NMR** (101 MHz, $CDCl_3$) δ 216.3, 209.5, 174.4, 132.1, 123.6, 64.19, 63.09, 61.34, 51.62, 49.04, 44.76, 44.22, 38.03, 37.83, 33.46, 32.10, 31.89, 27.58, 27.47, 26.42, 23.07, 23.03, 16.79, 13.59, 12.67 ppm.

Characteristic signals of the major *C1*-epimer of the $\Delta_{2,7}$-isomer: **^{1}H NMR** (400 MHz, $CDCl_3$) δ 4.82 (s, 1H), 4.59 (s, 1H), 4.32 – 4.24 (m, 1H), 4.10 (dd, *J* = 8.6, 4.3 Hz, 1H), 0.98 (s, 3H), 0.85 (s, 3H) ppm.

Mixture of $\Delta_{2,3}$-**40** and $\Delta_{2,7}$-**40** major *C1*-epimer: **IR** (neat) $\tilde{\nu}_{max}$ = 2931, 2875, 1727, 1713, 1458, 1382, 1324, 1259, 1162, 1123, 1103, 1068, 1021, 921, 846, 801 cm^{-1}; **HRMS** (ESI+) calcd. for $C_{25}H_{37}O_4^+$ $[M+H]^+$ 401.2686, found 401.2688;

Minor *C1*-epimer of the $\Delta_{2,3}$-isomer: **TLC:** R_f = 0.23 (silica gel, *n*-hexane/EtOAc; 80:20); **^{1}H NMR** (400 MHz, $CDCl_3$) δ 5.47 (s, 1H), 4.13 (dd, *J* = 11.8, 5.1 Hz, 1H), 4.08 (dd, *J* = 11.8, 3.4 Hz, 1H), 2.77 (q, *J* = 7.1 Hz, 1H), 2.69 – 2.56 (m, 2H), 2.45 (dd, *J* = 18.2, 4.9 Hz, 1H), 2.18 (td, *J* = 12.3, 5.4 Hz, 1H), 2.07 (t, *J* = 3.6 Hz, 1H), 2.05 – 1.94 (m, 3H), 1.71 (q, *J* = 1.9 Hz, 3H), 1.65 – 1.58 (m, 1H), 1.56 – 1.45 (m, 3H), 1.34 (d, *J* = 7.1 Hz, 3H), 1.33 (s, 3H), 1.32 – 1.28 (m, 2H), 1.14 (d, *J* = 6.8 Hz, 3H), 0.94 (s, 3H), 0.91 (s, 3H) ppm; **^{13}C NMR** (101 MHz, $CDCl_3$) δ 216.2, 209.5, 174.6, 132.0, 123.8, 64.34, 63.06, 61.39, 51.78, 48.94, 44.70, 44.27, 38.02, 37.61, 33.46, 32.38, 31.89, 27.63, 27.41, 26.21, 23.00, 22.96, 16.77, 13.61, 12.63 ppm; **IR** (neat) $\tilde{\nu}_{max}$ = 2937, 1728, 1458, 1382, 1323, 1256, 1160, 1105, 1058, 1022, 846, 800 cm^{-1}; **HRMS** (ESI+) calcd. for $C_{25}H_{37}O_4^+$ $[M+H]^+$ 401.2686, found 401.2689.

1-((9'*H*-Fluoren-9'-yl)methyl) 2-((2,6,6-trimethylcyclohex-2-en-1-yl)methyl) (2*S*)-pyrrolidine-1',2'-dicarboxylate (**41**), prepared from *N*-Fmoc-L-proline geranyl ester (70.6 mg, 149 µmol); colorless oil (41.5 mg, 87.6 µmol, 59%, 84:16 mixture of inseperable regioisomers, d.r. = 52:48 [*C1*-epimers]).

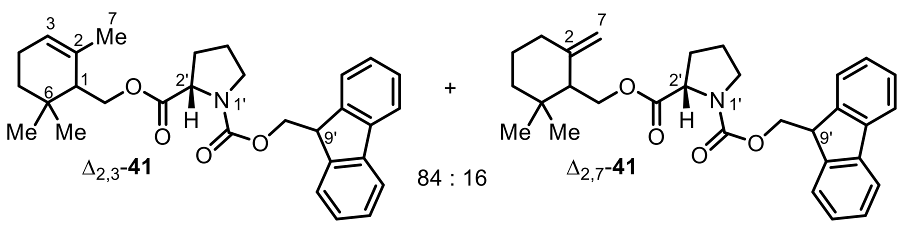

$\Delta_{2,3}$-**41** 84 : 16 $\Delta_{2,7}$-**41**

TLC: R_f = 0.38 (silica gel, *n*-hexane/EtOAc; 80:20); Major *C1*-epimer of $\Delta_{2,3}$-**41**: **^{1}H NMR** (400 MHz, $CDCl_3$) δ 7.76 (dd, *J* = 7.5, 4.9 Hz, 2H), 7.65 – 7.52 (m, 2H), 7.40 (td, *J* = 7.5, 3.3 Hz, 2H), 7.31 (tdd, *J* = 7.5, 2.6, 1.2 Hz, 2H), 5.43 (br s, 1H), 4.45 – 4.23 (m, 4H), 4.23 – 4.01 (m, 2H), 3.65 (br, 1H), 3.54 (br, H), 2.34 – 2.12 (m, 1H), 2.12 – 1.85 (m, 5H), 1.72 (d, *J* = 1.8 Hz, 1H), 1.66 (q, *J* = 2.0 Hz, 1H), 1.64 – 1.58 (m, 1H), 1.58 – 1.37 (m, 1H), 1.17 (dtd, *J* = 13.0, 9.6, 8.8, 6.6 Hz, 1H), 1.00 – 0.81 (m, 7H) ppm; **^{13}C NMR** (101 MHz, $CDCl_3$) δ 172.8, 172.7, 172.6, 155.0, 154.5, 144.3, 144.1, 143.9, 141.4, 132.2, 132.1, 131.8, 131.7, 127.8, 127.2, 127.2, 125.4, 125.3, 125.2, 123.7, 123.7, 123.5, 120.1, 67.89, 67.81, 67.59, 64.96, 64.68, 59.65, 59.59, 59.28, 49.03, 48.96, 48.91, 47.41, 47.15, 46.62, 32.35, 32.22, 31.86, 31.25, 31.19, 30.00, 29.93, 27.61, 27.50, 27.37, 27.29, 27.18, 27.06, 24.52, 23.58, 23.51, 23.03, 22.99 ppm.

Characteristic signals for $\Delta_{2,7}$-**41**: **^{1}H NMR** (400 MHz, $CDCl_3$) δ 4.81 (dd, *J* = 12.4, 7.4 Hz, 1H), 4.65 – 4.54 (m, 1H) ppm.

Mixture of $\Delta_{2,3}$-**41** and $\Delta_{2,7}$-**41**: **IR** (KBr) $\tilde{\nu}_{max}$ = 2957, 1740, 1704, 1451, 1419, 1350, 1176, 758 cm^{-1}; **HRMS** (ESI+) calcd. for $C_{30}H_{36}NO_4^+$ $[M+H]^+$ 474.2639, found 474.2639.

(2,6,6-Trimethylcyclohex-2-en-1-yl)methyl *N6'*-((benzyloxy)carbonyl)-*N2'*-(*tert*-butoxycarbonyl)-L-lysinate (**42**), prepared from *N*2'-Boc-*N*6'-Cbz-L-lysine geranyl ester (30.6 mg, 59.2 µmol); colorless semisolid (23.6 mg, 45.6 µmol, 77%, 88:12 mixture of inseperable regioisomers; d.r. = 50:50 [*C1*-epimers]).

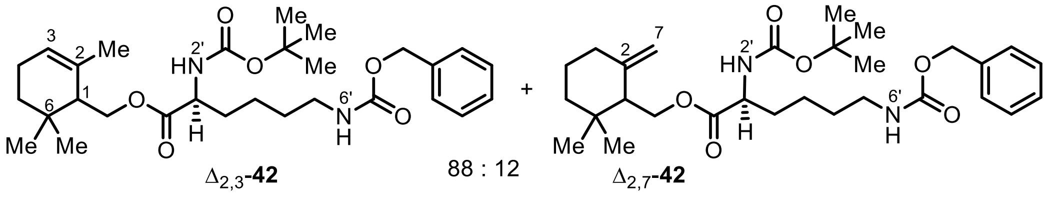

$\Delta_{2,3}$-**42** 88 : 12 $\Delta_{2,7}$-**42**

TLC: R_f = 0.18 (silica gel, *n*-hexane/EtOAc; 80:20); $\Delta_{2,3}$-**42**: **^{1}H NMR** (400 MHz, $CDCl_3$) δ 7.38 – 7.29 (m, 4H), 7.33 – 7.29 (m, 1H), 5.46 (s, 1H), 5.09 (s, 2H), 5.08 – 5.00 (m, 1H), 4.81 (br s, 1H), 4.29 (dd, *J* = 17.8, 8.5 Hz, 1H), 4.23 (dd, *J* = 11.7, 5.7 Hz, 1H), 4.11 (ddd, *J* = 11.4, 7.5, 3.2 Hz, 1H), 3.18 (d, *J* = 7.4 Hz, 2H), 2.18 – 2.01 (m, 1H), 1.97 (br, 2H), 1.78 (br, 2H), 1.71 (dd, *J* = 3.6, 1.8 Hz, 3H), 1.68 – 1.47 (m, 3H), 1.43 (s, 9H), 1.39 – 1.28 (m, 2H), 1.20 (dt, *J* = 13.7, 4.7 Hz, 1H), 0.93 (d, *J* = 2.6 Hz, 3H), 0.91 (s, 3H) ppm; **^{13}C NMR** (101 MHz, $CDCl_3$) δ 173.6, 156.4, 154.7, 136.6, 131.8, 131.7, 128.5, 128.14, 128.09, 123.6, 123.5, 79.89, 66.65, 65.02, 64.79, 53.26, 48.88, 48.82, 40.73, 32.37, 32.06, 31.99,

31.74, 29.40, 28.33, 27.36, 27.29, 27.19, 22.91, 22.88, 22.84, 22.37 ppm further signals could not be identified due to overlapped with the signals of the major isomer.

Characteristic signals of $\Delta_{2,3}$-**42**: **^{1}H NMR** (400 MHz, $CDCl_3$) δ 4.65 (s, 1H), 4.63 (s, 1H), 4.58 (s, 1H), 4.56 (s, 1H) ppm.

Mixture of $\Delta_{2,3}$-**42** and $\Delta_{2,7}$-**42**: **IR** (neat) $\tilde{\nu}_{max}$ = 3342, 2932, 1712, 1524, 1250, 1168, 1023, 698 cm^{-1}; **HRMS** (ESI+) calcd. for $C_{29}H_{44}N_2O_6Na^+$ $[M+Na]^+$ 539.3091, found 539.3102.

2-(2,6,6-Trimethylcyclohex-2-en-1-yl)acetonitrile (**43**), prepared from geranyl nitrile (37.0 mg, 227 µmol); yellow oil (33.3 mg, 204 µmol, 90%, 55:32:13 mixture of regioisomers).

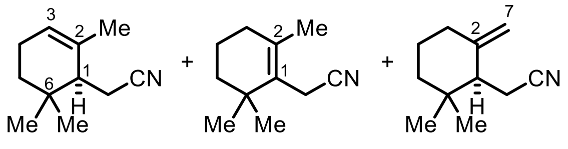

TLC: R_f = 0.33 (silica gel, *n*-hexane/EtOAc; 95:5) [CAM]; $\Delta_{2,3}$-**43**: **^{1}H NMR** (500 MHz, $CDCl_3$) δ 5.50 (t, *J* = 3.3 Hz, 1H), 2.45 (dd, *J* = 17.3, 6.3 Hz, 1H), 2.37 (dd, *J* = 17.4, 4.6 Hz, 1H), 2.01 (br, 2H), 1.75 (q, *J* = 1.9 Hz, 3H), 1.62 – 1.50 (m, 2H), 1.26 – 1.19 (m, 1H), 1.01 (s, 3H), 0.93 (s, 3H) ppm; **^{13}C NMR** (75 MHz, $CDCl_3$) δ 132.4, 124.0, 120.2, 46.28, 32.36, 31.08, 27.82, 27.37, 27.01, 22.77, 17.78 ppm.

Characteristic signals for $\Delta_{1,2}$-**43**: **^{1}H NMR** (500 MHz, $CDCl_3$) δ 2.98 (s, 2H), 1.98 (t, *J* = 6.3 Hz, 2H), 1.70 (s, 3H), 1.04 (s, 6H) ppm further signals could not be identified due to overlapped with the signals of the other isomer.

Characteristic signals of $\Delta_{2,7}$-**43**: **^{1}H NMR** (500 MHz, $CDCl_3$) δ 4.95 (s, 1H), 4.74 (s, 1H), 2.55 (dd, *J* = 16.7, 4.3 Hz, 1H), 2.09 (dd, *J* = 7.8, 5.4 Hz, 1H), 0.99 (s, 3H), 0.80 (s, 3H) ppm further signals could not be identified due to overlapped with the signals of the other isomer.

The spectroscopic data are in accordance with those reported in the literature.[12]

Diethyl 2-((2,6,6-trimethylcyclohex-2-en-1-yl)methyl)malonate (**44**), prepared from diethyl 2-geranyl malonate (44.2 mg, 149 µmol); colorless oil (38.0 mg, 128 µmol, 86%, 44:23:33 mixture of inseperable regioisomers);

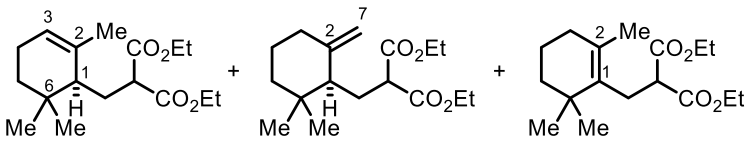

$\Delta_{2,3}$-**44** $\Delta_{2,7}$-**44** $\Delta_{1,2}$-**44**

44 : 23 : 33

TLC: R_f = 0.32 (silica gel, *n*-hexane/EtOAc; 90:10); Characteristic signals of $\Delta_{2,3}$-**44**: **^{1}H NMR** (400 MHz, $CDCl_3$) δ 5.30 (s, 1H), 4.23 – 4.13 (m, 4H), 3.47 (t, *J* = 7.0 Hz, 1H), 2.04 – 1.89 (m, 4H), 1.68 (q, *J* = 1.7 Hz, 3H), 1.30 – 1.23 (m, 6H), 1.13 (dt, *J* = 13.6, 4.4 Hz, 1H), 0.93 (s, 3H), 0.87 (s, 3H) ppm further signals could not be identified due to overlapped with the signals of the other isomers.

Characteristic signals of $\Delta_{2,7}$-**44**: **^{1}H NMR** (400 MHz, $CDCl_3$) δ 4.76 (s, 1H), 4.49 (s, 1H), 3.27 (dd, *J* = 11.2, 3.4 Hz, 1H), 2.12 (ddd, *J* = 14.1, 11.2, 3.1 Hz, 1H), 1.71 (dd, *J* = 12.2, 3.1 Hz, 1H), 0.86 (s, 3H), 0.82 (s, 3H) ppm further signals could not be identified due to overlapped with the signals of the other isomers.

Characteristic signals of $\Delta_{1,2}$-**44**: **^{1}H NMR** (400 MHz, $CDCl_3$) δ 2.67 (d, *J* = 7.0 Hz, 2H), 1.50 (s, 3H), 0.92 (s, 6H) ppm further signals could not be identified due to overlapped with the signals of the other isomers.

Mixture of $\Delta_{2,3}$-**44**, $\Delta_{2,7}$-**44**, and $\Delta_{1,2}$-**44**: **^{13}C NMR** (75 MHz, $CDCl_3$) δ 170.3, 170.0, 169.9, 169.7, 148.1, 135.9, 134.1, 130.6, 121.2, 110.6, 110.1, 61.45, 61.42, 61.23, 52.89, 52.77, 51.66, 50.55, 46.80, 40.25, 34.94, 33.16, 32.91, 30.96, 30.35, 28.80, 28.40, 27.53, 27.30, 27.24, 26.21, 23.60, 23.48, 23.13, 20.58, 19.42, 14.29, 14.26, 14.23, 14.19 ppm further signals could not be identified due to overlapped with the signals of the isomers.

The spectroscopic data are in accordance with those reported in the literature. [13]

2-((2,6,6-Trimethylcyclohex-2-en-1-yl)methyl)isoindolinedione (**45**), prepared from *N*-geranyl phtalimide (18.5 mg, 65.4 µmol); colorless oil (13.4 mg, 47.3 µmol, 72%, 72:13:15 mixture of inseperable regioisomers).

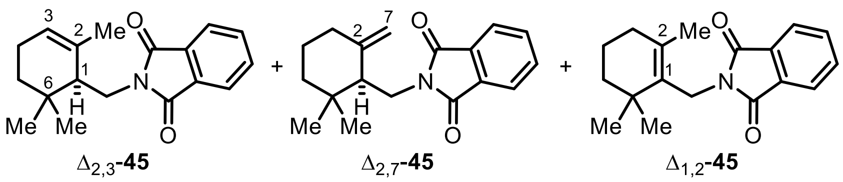

$\Delta_{2,3}$-**45** $\Delta_{2,7}$-**45** $\Delta_{1,2}$-**45**

72 : 13 : 15

TLC: R_f = 0.37 (silica gel, *n*-hexane/EtOAc; 90:10); $\Delta_{2,3}$-**45**: **^{1}H NMR** (400 MHz, $CDCl_3$) δ 7.83 (dd, *J* = 5.4, 3.1 Hz, 2H), 7.70 (dd, *J* = 5.5, 3.0 Hz, 2H), 5.41 (s, 1H), 3.82 (dd, *J* = 14.0, 7.0 Hz, 1H), 3.54 (dd, *J* = 14.0, 5.9 Hz, 1H), 2.25 (t, *J* = 6.5 Hz, 1H), 2.10 – 1.96 (m, 2H), 1.72 (q, *J* = 1.9 Hz, 3H), 1.65 – 1.55 (m, 1H); 1.26 – 1.17 (m, 1H), 0.96 (s, 3H), 0.89 (s, 3H) ppm; **^{13}C NMR** (101 MHz, $CDCl_3$) δ 168.8, 134.0, 132.3, 123.3, 123.2, 122.8, 47.29, 40.21, 32.21, 30.40, 27.31, 26.96, 23.77, 23.20 ppm.

Characteristic signals of $\Delta_{2,7}$-**45**: **^{1}H NMR** (400 MHz, $CDCl_3$) δ 4.59 (t, *J* = 2.2 Hz, 1H), 4.41 (t, *J* = 2.0 Hz, 1H), 4.00 (dd, *J* = 13.4, 12.2 Hz, 1H), 3.69 (dd, *J* = 13.4, 4.6 Hz, 1H) ppm further signals could not be identified due to overlapped with the signals of the other isomers.

Characteristic signals of $\Delta_{1,2}$-**45**: **^{1}H NMR** (400 MHz, $CDCl_3$) δ 4.39 (s, 2H), 1.81 (s, 3H), 1.04 (s, 3H) ppm further signals could not be identified due to overlapped with the signals of the other isomers.

Mixture of $\Delta_{2,3}$-**45**, $\Delta_{2,7}$-**45**, and $\Delta_{1,2}$-**45**: **IR** (neat) $\tilde{\nu}_{max}$ = 2916, 1771, 1713, 1436, 1398, 1353, 717 cm^{-1}; **HRMS** (ESI+) calcd. for $C_{18}H_{22}NO_2^+$ $[M+H]^+$ 284.1645, found 284.1646.

1,1,4a,10b-tetramethyl-1,2,3,4,4a,4b,5,6,10b,11,12,12a-dodecahydrochrysene (**46**), prepared from (*E*,*E*)-homofarnesyl benzene (35.2 mg, 119 µmol); colorless oil (24.3 mg, 82.1 µmol, 69%, d.r > 95:5).

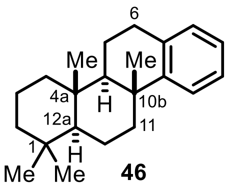

46

TLC: R_f = 0.56 (silica gel, *n*-hexane); **^{1}H NMR** (500 MHz, $CDCl_3$) δ 7.25 (d, *J* = 7.5 Hz, 1H), 7.12 (t, *J* = 7.5 Hz, 1H), 7.08 – 7.01 (m, 2H), 2.93 (ddd, *J* = 17.2, 6.6, 1.8 Hz, 1H), 2.86 – 2.78 (m, 1H), 2.39 (dd, *J* = 9.5, 3.2 Hz, 1H), 1.85-1.76 (m, 2H), 1.73 – 1.62 (m, 3H), 1.53 – 1.35 (m, 6H), 1.30 – 1.25 (m, 2H), 1.20 (s, 3H), 0.93 (s, 3H), 0.87 (s, 3H), 0.86 (s, 3H); **^{13}C NMR** (126 MHz, $CDCl_3$) δ 150.6, 135.3, 128.9, 125.8, 125.2, 124.7, 56.40, 55.30, 42.20, 40.71, 39.95, 38.29, 37.80, 33.48, 33.44, 31.02, 26.31, 21.60, 19.27, 18.76, 18.09, 16.44 ppm.

The spectroscopic data are in accordance with those reported in the literature. [13]

(±)-Drimenol acetate (**47**), prepared from (*E,E*)-farnesyl acetate (39.4 mg, 149 µmol); colorless oil (25.9 mg, 98.0 µmol, 66%, d.r. = 65:35 [*C1*-epimer]).

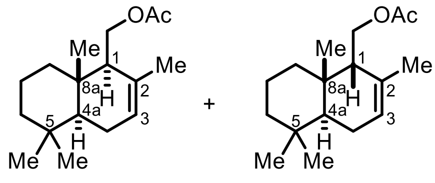

trans,trans-**47** *trans,cis*-**47**

TLC: R_f = 0.61 (silica gel, *n*-hexane/EtOAc; 90:10); major diastereoisomer *trans,trans*-**47**: **^{1}H NMR** (300 MHz, $CDCl_3$) δ 5.52 – 5.42 (m, 1H), 4.24 (dd, *J* = 11.6, 3.3 Hz, 1H), 4.08 (dd, *J* = 11.7, 6.4 Hz, 1H), 2.03 (s, 3H), 2.05 – 1.82 (m, 4H), 1.67 (dt, *J* = 2.5, 1.4 Hz, 3H), 1.54 – 1.37 (m, 3H), 1.24 – 1.08 (m, 3H), 0.89 (s, 3H), 0.86 (s, 3H), 0.81 (s, 3H) ppm.

Characteristic signals of the minor diastereoisomer *trans,cis*-**47**: **^{1}H NMR** (300 MHz, $CDCl_3$) δ 5.44 (br s, 1H) ppm further signals could not be identified due to overlapped with the signals of the major isomers.

Mixture of *trans,trans*-**47** and *trans,cis*-**47**: **^{13}C NMR** (101 MHz, $CDCl_3$) δ 171.3, 132.7, 123.8, 63.36, 53.52, 50.01, 42.23, 39.70, 36.10, 33.45, 33.11, 23.75, 22.08, 21.85, 21.40, 18.87, 14.62 ppm.

The spectroscopic data are in accordance with those reported in the literature. [14]

(2,5,5,8a-Tetramethyl-1,4,4a,5,6,7,8,8a-octahydronaphthalen-1-yl)methyl 3’,5’-dimethoxybenzoate (**48**), prepared from (*E*,*E*)-farnesyl 3,5-dimethoxybenzoate (48.2 mg, 125 µmol); colorless oil (29.7 mg, 76.8 µmol, 62%, d.r = 65:35 [*C1*-epimer]).

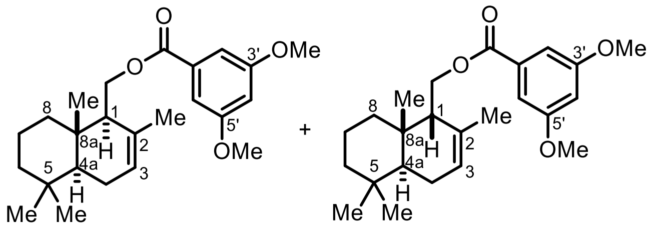

trans,trans-**48** *trans,cis*-**48**

TLC: R_f = 0.61 (silica gel, *n*-hexane/EtOAc; 90:10); major diastereoisomer *trans,trans*-**48**: **^{1}H NMR** (400 MHz, $CDCl_3$) δ 7.17 (d, *J* = 2.3 Hz, 2H), 6.64 (t, *J* = 2.4 Hz, 1H), 5.56 – 5.53 (m, 1H), 4.55 (dd, *J* = 11.7, 3.4 Hz, 1H), 4.30 (dd, *J* = 11.7, 6.0 Hz, 1H), 3.82 (s, 6H), 2.18 (br s, 1H), 2.08 – 1.86 (m, 2H), 1.74 (s, 3H), 1.64 – 1.53 (m, 2H), 1.52 – 1.38 (m, 3H), 1.25 (q, *J* = 5.1 Hz, 1H), 1.17 (dd, *J* = 12.9, 3.9 Hz, 1H), 0.90 (s, 6H), 0.88 (s, 3H) ppm; **^{13}C NMR** (75 MHz, $CDCl_3$) δ 166.5, 160. 8, 132.6, 132.5, 124.1, 107.3, 105.7, 63.85, 55.68, 53.77, 50.04, 42.23, 39.83, 36.21, 33.48, 33.13, 23.80, 22.12, 22.05, 18.90, 14.87 ppm.

Characteristic signals of the minor diastereoisomer trans,cis-**48**: **^{1}H NMR** (400 MHz, $CDCl_3$) δ 5.48 (t, *J* = 7.4 Hz, 1H), 1.00 (s, 3H) ppm further signals could not be identified due to overlapped with the signals of the major isomers.

Mixture of *trans,trans*-**48** and *trans,cis*-**48**: **IR** (KBr) $\tilde{\nu}_{max}$ = 2926, 1716, 1598, 1459, 1303, 1230, 1157, 1052, 759 cm^{-1}; **HRMS** (ESI+) calcd. for $C_{24}H_{35}O_4^+$ $[M+H]^+$ 387.2530, found 387.2537.

(±)-sclareolide (**49**), prepared from (*E*,*E*)-homofarnesoic acid (31.0 mg, 124 µmol); colorless solid (17.7 mg, 70.7 µmol, 57%); 9-*epi*-sclareolide (minor diastereomer) could not be separated from further side products.

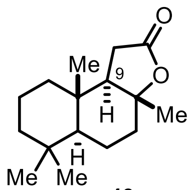

49

(±)-49: **TLC:** R_f = 0.44 (silica gel, *n*-hexane/EtOAc; 80:20) [$KMnO_4$]; **^{1}H NMR** (300 MHz, $CDCl_3$) δ 2.41 (dd, *J* = 16.2, 14.7 Hz, 1H), 2.23 (ddd, *J* = 16.2, 6.5, 0.6 Hz, 1H), 2.08 (dt, *J* = 11.8, 3.3 Hz, 1H), 1.97 (dd, *J* = 14.7, 6.5 Hz, 1H), 1.88 (ddt, *J* = 14.0, 4.2, 2.9 Hz, 1H), 1.75 – 1.62 (m, 2H), 1.51 – 1.35 (m, 4H), 1.33 (d, *J* = 1.0 Hz, 3H), 1.19 (td, *J* = 13.6, 4.3

Hz, 1H), 1.04 (ddd, *J* = 13.8, 10.3, 3.3 Hz, 2H), 0.91 (s, 3H), 0.89 (s, 3H), 0.84 (s, 3H) ppm; **^{13}C NMR** (75 MHz, $CDCl_3$) δ 177.0, 86.52, 59.28, 56.82, 42.33, 39.67, 38.88, 36.22, 33.32, 33.28, 28.87, 21.72, 21.07, 20.71, 18.24, 15.22 ppm.

The spectroscopic data are in accordance with those reported in the literature. [15]

1,3,3-Trimethyl-2-oxabicyclo[2.2.2]octane (**50**), prepared from (-)-α-terpineol (17.6 mg, 114 µmol); colorless liquid (13.5 mg, 87.5 µmol, 77%; d.r. ≥ 95:5).

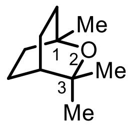

50

TLC: R_f = 0.63 (silica gel, *n*-hexane/EtOAc; 95:5); **^{1}H NMR** (400 MHz, $CDCl_3$) δ 2.01 (tdd, *J* = 9.1, 3.8, 1.2 Hz, 2H), 1.73 – 1.57 (m, 2H), 1.56 – 1.43 (m, 4H), 1.39 (tq, *J* = 3.4, 1.2 Hz, 1H), 1.23 (s, 6H), 1.04 (s, 3H) ppm; **^{13}C NMR** (101 MHz, $CDCl_3$) δ 73.76, 69.92, 33.06, 31.63, 29.02, 27.71, 22.95 ppm.

The spectroscopic data are in accordance with those reported in the literature. [16]

2,2-Dimethyl-3,4-dihydro-2H-benzo[h]chromene-5,6-dione (**51**), prepared from lapachol (31.0 mg, 128 µmol); orange crystals (27.0 mg, 111 µmol, 87%).

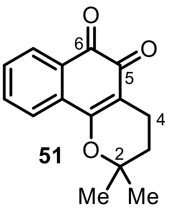

51

TLC: R_f = 0.20 (silica gel, n-hexane/EtOAc; 80:20); **^{1}H NMR** (400 MHz, $CDCl_3$) δ 8.04 (dd, *J* = 7.6, 1.2 Hz, 1H), 7.80 (dd, *J* = 7.9, 1.2 Hz, 1H), 7.63 (td, *J* = 7.6, 1.4 Hz, 1H), 7.49 (td, *J* = 7.5, 1.2 Hz, 1H), 2.56 (t, *J* = 6.7 Hz, 2H), 1.85 (t, *J* = 6.7 Hz, 2H), 1.46 (s, 6H) ppm; **^{13}C NMR** (101 MHz, $CDCl_3$) δ 180.0, 178.7, 162.2, 134.9, 132.8, 130.8, 130.3, 128.7, 124.2, 112.9, 79.40, 31.74, 26.89 (2C), 16.29 ppm.

The spectroscopic data are in accordance witAnalysiseported in the literature. [17]

7. X-ray Crystal Analysis of DABCO-PFTB 54 (**CCDC 2205029/ArnAn8**)

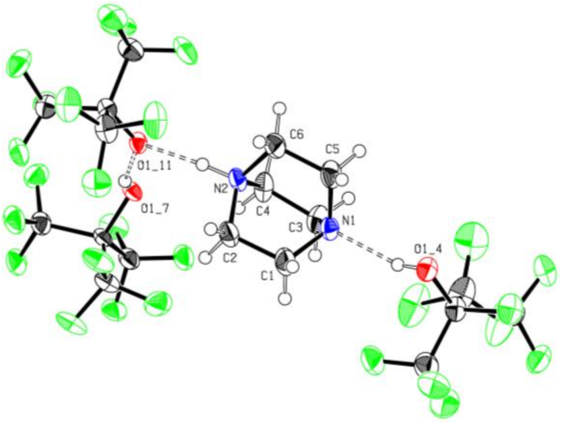

Supplementary Figure 15. X-ray crystal structure of DABCO-$(PFTB)_2$ salt (**54**). Ortez drawing with 50% ellipsoids shown.

A suitable crystal of compound **54** was grown by slow diffusion of a mixture of DABCO in excess PFTB. A colorless fragment-like specimen of $C_{72}H_{60}F_{108}N_8O_{12}$, approximate dimensions 0.252 mm x 0.276 mm x 0.295 mm, was used for the X-ray crystallographic analysis.

Data were collected on a Bruker D8 Venture single crystal x-ray diffractometer equipped with a CMOS detector (Bruker Photon-100), a TXS rotating anode with MoK_{α} radiation (λ = 0.71073 Å) and a Helios optic using the APEX3 software package. [18] Measurements were performed on single crystals coated with perfluorinated ether. The crystals were fixed on top of a kapton micro sampler and frozen under a stream of cold nitrogen. A matrix scan was used to determine the initial lattice parameters. Reflections were corrected for Lorentz and polarisation effects, scan speed, and background using SAINT. [19] Absorption correction, including odd and even ordered spherical harmonics was performed using SADABS. [19] Space group assignments were based upon systematic absences, E statistics, and successful refinement of the structures. The structure was solved using SHELXT with the aid of successive difference Fourier maps and was refined as a perfect inversion twin against all data using SHELXL in conjunction with SHELXLE. [20] Hydrogen atoms (except on heteroatoms) were calculated in ideal positions as follows: Methyl hydrogen atoms were refined as part of rigid rotating groups with a C–H distance of 0.98 Å and $U_{iso}(H) = 1.5 \cdot U_{eq}(C)$. Non-methyl H atoms were placed in calculated positions and refined using a riding model with methylene, aromatic, and other C–H distances of 0.99 Å, 0.95 Å, and 1.00 Å, respectively, all with $U_{iso}(H) = 1.2 \cdot U_{eq}(C)$. H atoms bound to heteroatoms were refined freely, if possible, and with appropriate restraints otherwise. Non-hydrogen atoms were refined with anisotropic displacement parameters. Full-matrix least-squares refinements were carried out by minimizing $\Sigma w(F_o^2 - F_c^2)^2$ with the SHELXL weighting scheme.[3] Neutral atom scattering factors for all atoms and anomalous dispersion corrections for the non-hydrogen atoms were taken from *International Tables for Crystallography*. [21] Disordered PFTB molecules were refined using a split layer refinement and appropriate geometries and restraints provided by DSR. [22] Images of

the crystal structures were generated with PLATON. [23] CCDC 2205029 contains the supplementary crystallographic data for this paper. These data are provided free of charge by The Cambridge Crystallographic Data Centre.

Diffractometer operator C. Jandl
scanspeed 1-10 s per frame
dx 100 mm
5876 frames measured in 19 data sets
phi-scans with delta_phi = 0.5
omega-scans with delta_omega = 0.5
shutterless mode

7.1 Crystal data

$2(C_4HF_9O)\cdot C_4F_9O\cdot C_6H_{13}N_2$

M_r = 820.32	D_x = 1.962 Mg m^{-3}
Monoclinic, *Cc*	Melting point: ? K
Hall symbol: C -2yc	Mo *K*α radiation, λ = 0.71073 Å
a = 51.072 (5) Å	Cell parameters from 9763 reflections
b = 10.0761 (11) Å	θ = 2.7–26.9°
c = 22.372 (3) Å	μ = 0.25 mm^{-1}
β = 105.274 (4)°	*T* = 103 K
V = 11106 (2) Å^3	Fragment, colorless
Z = 16	0.30 × 0.28 × 0.25 mm
F(000) = 6464	

7.2 Data Collection

Bruker Photon CMOS diffractometer	20316 independent reflections
Radiation source: TXS rotating anode	19605 reflections with $I > 2\sigma(I)$
Helios optic monochromator	R_{int} = 0.037
Detector resolution: 16 pixels mm^{-1}	θ_{max} = 25.4°, θ_{min} = 2.1°
phi– and ω–rotation scans	*h* = −61 61
Absorption correction: multi-scan *SADABS* 2016/2, Bruker	*k* = −12 12

T_{min} = 0.695, T_{max} = 0.745

l = −26 26

188659 measured reflections

7.3 Refinement

Refinement on *F*2

Least-squares matrix: full

$R[F^2 > 2\sigma(F^2)]$ = 0.033

$wR(F^2)$ = 0.089

S = 1.01

20316 reflections

2217 parameters

28488 restraints

0 constraints

Primary atom site location: iterative

Secondary atom site location: difference Fourier map

Hydrogen site location: mixed

H atoms treated by a mixture of independent and constrained refinement

W = 1/[Σ2(*F*O2) + (0.0563*P*)2 + 8.9206*P*] WHERE *P* = (*F*O2 + 2*F*C2)/3

$(\Delta/\sigma)_{max}$ = 0.001

$\Delta\rho_{max}$ = 0.42 e Å^{-3}

$\Delta\rho_{min}$ = −0.30 e Å^{-3}

Extinction correction: none

Extinction coefficient: -

Absolute structure: Flack[9]

Absolute structure parameter: 0.5 (4)

8. Simulation Details

MD investigations were performed with the GROMACS simulation package[24] (versions ranging from 2018-2020) with the GAFF[25] force field with a timestep of 2 fs and the Verlet scheme. The corresponding point charges were obtained from the wfx-files with Multiwfn 3.6[26] after geometry optimization at B3LYP 6-311G** level with ORCA (versions 4.1.2 and 4.2.1)[27] employing a RESP-fitting procedure for equivalence of hydrogen CH3 and CH2. One must note that no equivalence criteria were defined for CF3 Groups. The charges on cyclohexane were averaged to obtain equivalent positions. For the bromide, the parameters in literature[28] were used. (Likewise for the control simulation containing sodium cations.)

Two boxes used for umbrella sampling contained 300 solvent molecules (either cyclohexane or PFTB), 10 py-H cations, 10 bromide anions, and one homogeranyl benzene molecule (**19**). Two other boxes were prepared with the same numbers but without the ionic species.

They were equilibrated for at least 10ns at the reference temperature of 300K with the velocity-rescaling thermostat ($\tau_T = 1$ps) and a reference-pressure of 1 bar given the Parrinello-Rahman barostat ($\tau_p = 2$ps, compressibility = $4.5 * 10^{-5}$/bar). Coulomb interactions were treated by PME. The VdW modifier was chosen as 'Potential-Shift-Verlet'.

For the umbrella windows, a force constant of 1000 kJ/mol/nm^2 was chosen. The coordinate determined was the distance between the carbon atoms, which are involved in the bond formation. This allows for the determination of the free energy difference between the different solvent cases in the presence and without the presence of the number of ions mentioned above. This corresponds to the amount of stabilization of the folded to the unfolded state of **19**. Every 50th frame was written to file. From this, the PMF was obtained with the program gmx wham once equilibration was reached. The number of umbrella windows (4-12) was adjusted so that all histograms yielded sufficient overlap and equilibration could be assumed.

Every umbrella window was simulated at least for 30ns. In the case where ions were present in the solvent PTFB, the windows were sampled up to 200ns (of which at least the first 20 were discarded in addition to the regular equilibration period) because equilibration needed to take the slow formation of the supramolecular structures into account.

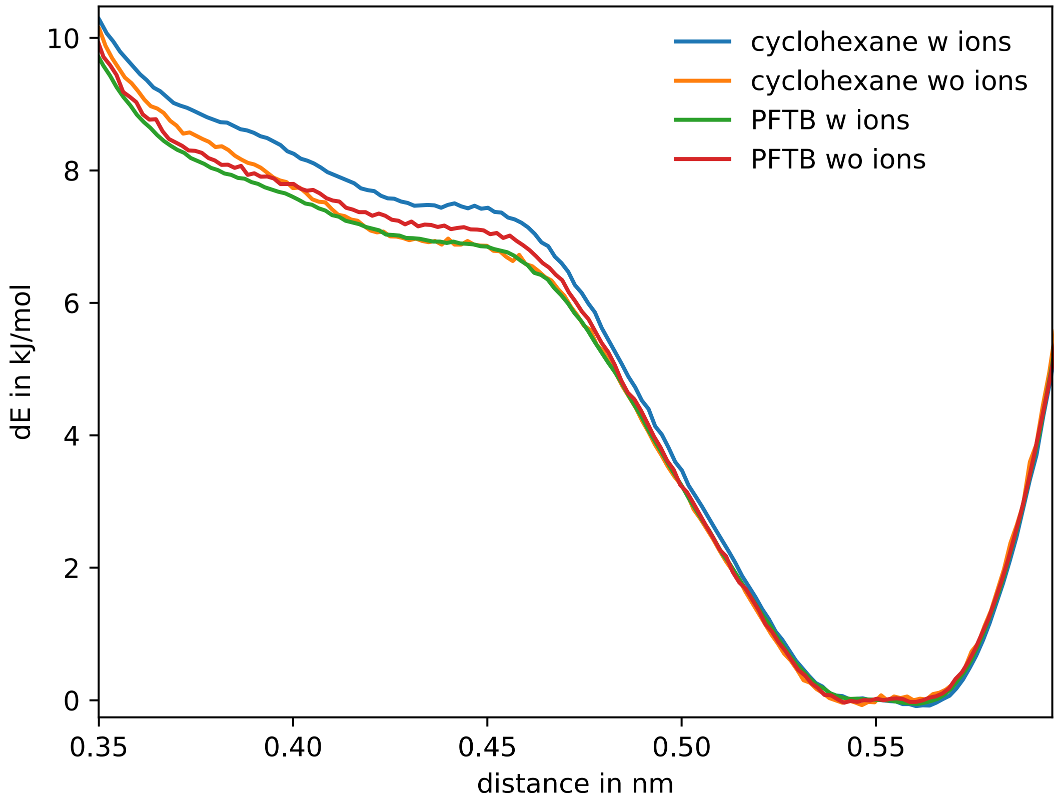

Supplementary Figure 16. PMF of the C-C distance for the reacting carbon atoms in homogeranyl benzene (**19**) in PFTB as opposed to cyclohexane at conditions described above.

To compare the ionic aggregates of catalysts **20b** and **20c** in PTBF we simulated DABCO-H^+, TfOH, TfO^- as those are expected to play the main role in the experiments. The forcefields were obtained employing the same procedure as described above and the simulations run with conditions as before except no umbrella sampling was employed for the conformational stability of homogeranyl benzene (**19**) in solution. The system size employed was set to 8 times the previous size (2400 solvent molecules, 80 anions and cations) for efficiency (Figure S9).

Additionally, a second system was simulated where 172 of the solvent were replaced by 80 TfOH (1:1 to TfO^-) molecules and keeping the DABCO concentration roughly identical (Figure S10). Both DABCO bistriflate systems showed the familiar extended ionic aggregates as previously observed for the pyridinium-HBr system.

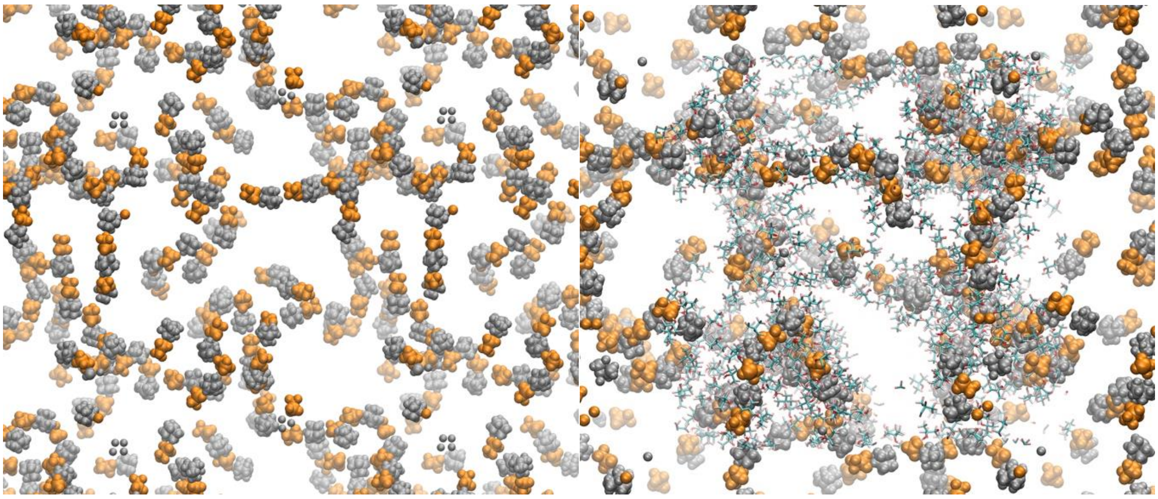

Supplementary Figure 17. Snapshot of the $DABCOH^+/TfO^-$ simulation – PFTB shown on the right-hand side revealing a preferential OH orientation towards the aggregates.

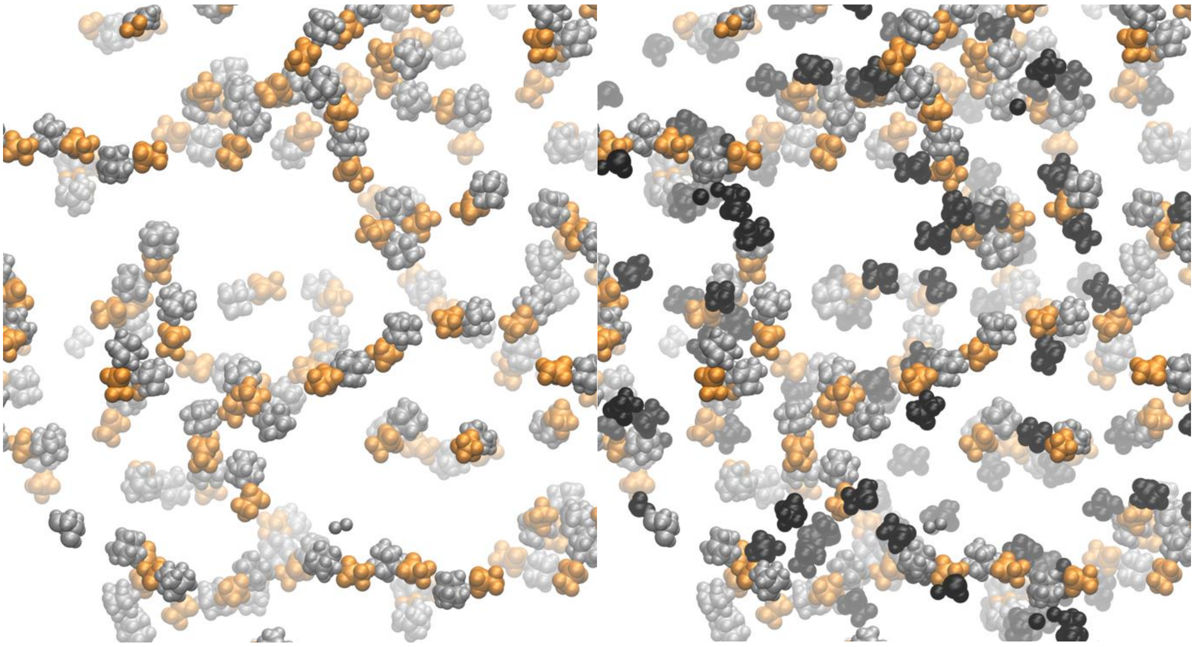

Supplementary Figure 18. Snapshot of the $DABCOH^+/TfO^-/TfOH$ simulation – TfOH displayed in black on the right-hand side.

9. Supplementary References

1 Glazunov, V. P. & Odinokov, S. E. Infrared spectra of pyridinium salts in solution—I. The region of middle frequencies. *Spectrochim. Act. A* **38**, 399-408, doi:https://doi.org/10.1016/0584-8539(82)80014-7 (1982).

2 a) Stonehouse, J., Adell, P., Keeler, J. & Shaka, A. J. Ultrahigh-Quality NOE Spectra. *J. Am. Chem. Soc.* **116**, 6037-6038, doi:10.1021/ja00092a092 (1994), b) Thrippleton, M. J. & Keeler, J. Elimination of Zero-Quantum Interference in Two-Dimensional NMR Spectra. *Angew. Chem. Int. Ed.* **42**, 3938-3941, doi:https://doi.org/10.1002/anie.200351947 (2003).

3 a) Mayer, U., Gutmann, V. & Gerger, W. The acceptor number — A quantitative empirical parameter for the electrophilic properties of solvents. *Monatsh. Chemie* **106**, 1235-1257, doi:10.1007/BF00913599 (1975), b) Beckett, M. A., Strickland, G. C., Holland, J. R. & Sukumar Varma, K. A convenient n.m.r. method for the measurement of Lewis acidity at boron centres: correlation of reaction rates of Lewis acid initiated epoxide polymerizations with Lewis acidity. *Polymer* **37**, 4629-4631, doi:https://doi.org/10.1016/0032-3861(96)00323-0 (1996).

4 Chen, Y., Zhao, Y.-F., Yin, Y.-W. & Yang, X.-Q. N-Phosphoryl Amino Acids and Peptides: Part V: O-Alkyl substituted effects on the 31P-NMR Spectra of Phosphoramidates. *Phosphorus Sulfur* **61**, 31-39, doi:10.1080/10426509108027334 (1991).

5 Beckett, M. A., Brassington, D. S., Coles, S. J. & Hursthouse, M. B. Lewis acidity of tris(pentafluorophenyl)borane: crystal and molecular structure of B(C6F5)3·OPEt3. *Inorg. Chem. Commun.* **3**, 530-533, doi:https://doi.org/10.1016/S1387-7003(00)00129-5 (2000).

6 Surendra, K. & Corey, E. J. Highly Enantioselective Proton-Initiated Polycyclization of Polyenes. *J. Am. Chem. Soc.* **134**, 11992-11994, doi:10.1021/ja305851h (2012).

7 Chou, T.-H., Yu, B.-H. & Chein, R.-J. ZnI_2/$Zn(OTf)_2$-TsOH: a versatile combined-acid system for catalytic intramolecular hydrofunctionalization and polyene cyclization. *Chem. Commun.* **55**, 13522-13525, doi:10.1039/C9CC07242J (2019).

8 Sakakura, A., Sakuma, M. & Ishihara, K. Chiral Lewis Base-Assisted Brønsted Acid (LBBA)-Catalyzed Enantioselective Cyclization of 2-Geranylphenols. *Org. Lett.* **13**, 3130-3133, doi:10.1021/ol201032t (2011).

9 Imamura, P. M. & Santiago, G. M. P. Chlorosulfonic Acid Mediated Cyclization of Homoterpenic Acid. *Synth. Commun.* **27**, 2479-2485, doi:10.1080/00397919708004111 (1997).

10 Upar, K. B., Mishra, S. J., Nalawade, S. P., Singh, S. A., Khandare, R. P. & Bhat, S. V. Efficient enantioselective synthesis of (+)-sclareolide and (+)-tetrahydroactinidiolide: chiral LBA-induced biomimetic cyclization. *Tetrahedron Asym.* **20**, 1637-1640, doi:https://doi.org/10.1016/j.tetasy.2009.06.020 (2009).

11 Tsangarakis, C. & Stratakis, M. Biomimetic Cyclization of Small Terpenoids Promoted by Zeolite NaY: Tandem Formation of α-Ambrinol from Geranyl Acetone. *Adv. Synth. Catal.* **347**, 1280-1284, doi:10.1002/adsc.200505059 (2005).

12 Fernandez Mateos, A. & Lopez Barba, A. M. Limonoid Model Insect Antifeedants. A Stereoselective Synthesis of Azadiradione C, D, and E Fragments through Intramolecular Diazo Ketone Cyclization. *J. Org. Chem.* **60**, 3580-3585, doi:10.1021/jo00117a005 (1995).

13 Fernández Mateos, A., Pascual Coca, G., Pérez Alonso, J. J., González, R. R. & Hernández, C. T. π Participation in nucleophilic displacement of α-cyclogeranyl tosylate. *Tetrahedron Lett.* **36**, 621-624, doi:https://doi.org/10.1016/0040-4039(94)02318-6 (1995).

14 Hayakawa, I., Nakamura, T., Ohno, O., Suenaga, K. & Kigoshi, H. Synthesis and structure–activity relationships for cytotoxicity and apoptosis-inducing activity of (+)-halichonine B. *Org. Biomol. Chem.* **13**, 9969-9976, doi:10.1039/C5OB01488C (2015).

15 Laudadio, G., Govaerts, S., Wang, Y., Ravelli, D., Koolman, H. F., Fagnoni, M., Djuric, S. W. & Noël, T. Selective C(sp3)−H Aerobic Oxidation Enabled by Decatungstate Photocatalysis in Flow. *Angew. Chem. Int. Ed.* **57**, 4078-4082, doi:10.1002/anie.201800818 (2018).

16 J. Abraham, R., A. Warne, M. & Griffiths, L. Proton chemical shifts in NMR. Part 12.1 Steric, electric field and conformational effects in acyclic and cyclic ethers. *J. Chem. Soc. Perkin 2*, 1751-1758, doi:10.1039/A802950D (1998).

17 Gontijo, T. B., de Freitas, R. P., de Lima, G. F., de Rezende, L. C. D., Pedrosa, L. F., Silva, T. L., F. Goulart, M. O., Cavalcanti, B. C., Pessoa, C., Bruno, M. P., Corrêa, J. R., Emery, F. S. & da Silva Júnior, E. N. Novel fluorescent lapachone-based BODIPY: synthesis, computational and electrochemical aspects, and subcellular localisation of a potent antitumour hybrid quinone. *Chem. Commun.* **52**, 13281-13284, doi:10.1039/C6CC07054J (2016).

18 APEX suite of crystallographic software, APEX 3, Version 2016-9.0, Bruker AXS Inc., Madison, Wisconsin, USA, 2016.

19 SAINT, Version 8.40A and SADABS, Version 2016/2, Bruker AXS Inc., Madison, Wisconsin, USA, 2016/2019.

20 a) Hübschle, C. B., Sheldrick, G. M. & Dittrich, B. ShelXle: a Qt graphical user interface for SHELXL. *J. Appl. Crystallogr.* **44**, 1281-1284, doi:doi:10.1107/S0021889811043202 (2011), b) Sheldrick, G. Crystal structure refinement with SHELXL. *Acta Crystallogr. Sect. C* **71**, 3-8, doi:doi:10.1107/S2053229614024218 (2015), c) Sheldrick, G. SHELXT - Integrated space-group and crystal-structure determination. *Acta Crystallogr. Sect. A* **71**, 3-8, doi:doi:10.1107/S2053273314026370 (2015).

21 *International Tables for Crystallography*, Vol. C (Ed.: A. J. Wilson), Kluwer Academic Publishers, Dordrecht, The Netherlands, **1992**, Tables 6.1.1.4 (pp. 500–502), 4.2.6.8 (pp. 219–222), and 4.2.4.2 (pp. 193–199).

22 Spek, A. Structure validation in chemical crystallography. *Act. Cryst. D* **65**, 148-155, doi:doi:10.1107/S090744490804362X (2009).

23 Kratzert, D. & Krossing, I. Recent improvements in DSR. *J. Appl. Crystallogr.* **51**, 928-934, doi:doi:10.1107/S1600576718004508 (2018).

24 Abraham, M. J., Murtola, T., Schulz, R., Páll, S., Smith, J. C., Hess, B. & Lindahl, E. GROMACS: High performance molecular simulations through multi-level parallelism from laptops to supercomputers. *SoftwareX* **1-2**, 19-25, doi:https://doi.org/10.1016/j.softx.2015.06.001 (2015).

25 Wang, J., Wolf Rm Fau - Caldwell, J. W., Caldwell Jw Fau - Kollman, P. A., Kollman Pa Fau - Case, D. A. & Case, D. A. Development and testing of a general amber force field. *J. Comput. Chem.* **25**, 1157-1174 (2004).

26 Lu, T. & Chen, F. Multiwfn: A multifunctional wavefunction analyzer. *J. Comp. Chem.* **33**, 580–592 (2012).

27 Neese, F. Software update: the ORCA program system, version 4.0. *WIREs Comp. Mol. Sci.* **8**, e1327 (2018).

28 Horinek, D., Mamatkulov, S. I. & Netz, R. R. Rational design of ion force fields based on thermodynamic solvation properties. *J. Chem. Phys.* **130**, 124507, doi:10.1063/1.3081142 (2009).
