# Supplementary material for: Enzyme-like polyene cyclizations catalyzed by dynamic, self-assembled, supramolecular fluoro alcohol-amine clusters
Source: Nat Commun. 2023 Feb 13;14:813. doi: 10.1038/s41467-023-36157-0 (PMC9925744; doi:10.1038/s41467-023-36157-0)
Supplement: Supplementary file 2 — Description of Additional Supplementary Files [file 41467_2023_36157_MOESM2_ESM.pdf]

Description of Additional Supplementary Files

Supplementary Data 1: The NMR data of compounds 21 – 51.

Supplementary Data 2: contains the structural data for compound 54 including its cartesian coordinates.

Supplementary Data 3: contains the parameters for the simulations conducted in this study.
