# Supplementary material for: Enzyme-like polyene cyclizations catalyzed by dynamic, self-assembled, supramolecular fluoro alcohol-amine clusters
Source: Nat Commun. 2023 Feb 13;14:813. doi: 10.1038/s41467-023-36157-0 (PMC9925744; doi:10.1038/s41467-023-36157-0)
Supplement: Supplementary file 3 — Supplementary Data 1 [file 41467_2023_36157_MOESM3_ESM.pdf]

Enzyme-Like Polyene Cyclizations Catalyzed by Dynamic, Self-Assembled, Supramolecular Fluoro Alcohol-Amine Clusters

Andreas M. Arnold,[1,2] Philipp Dullinger,[3] Aniruddha Biswas,[2] Christian Jandl,[4] Dominik Horinek[3] and Tanja Gulder[1,2,4*]

[1]Biomimetic Catalysis, Department of Chemistry, Technical University Munich, Lichtenbergstraße 4, 85747 Garching, Germany
[2]Chair of Organic Chemistry, Faculty of Chemistry and Mineralogy, Leipzig University, Johannisallee 29, 04103 Leipzig, Germany[3]Institute of Physical and Theoretical Chemistry, University of Regensburg, 93040 Regensburg, Germany
[4]Catalysis Research Center, Technical University Munich, Ernst-Otto-Fischer-Straße 1, 85747 Garching, Germany

SUPPLEMENTARY DATA 1: ^{1}H AND ^{13}C NMR SPECTRA OF COMPOUNDS 21-51

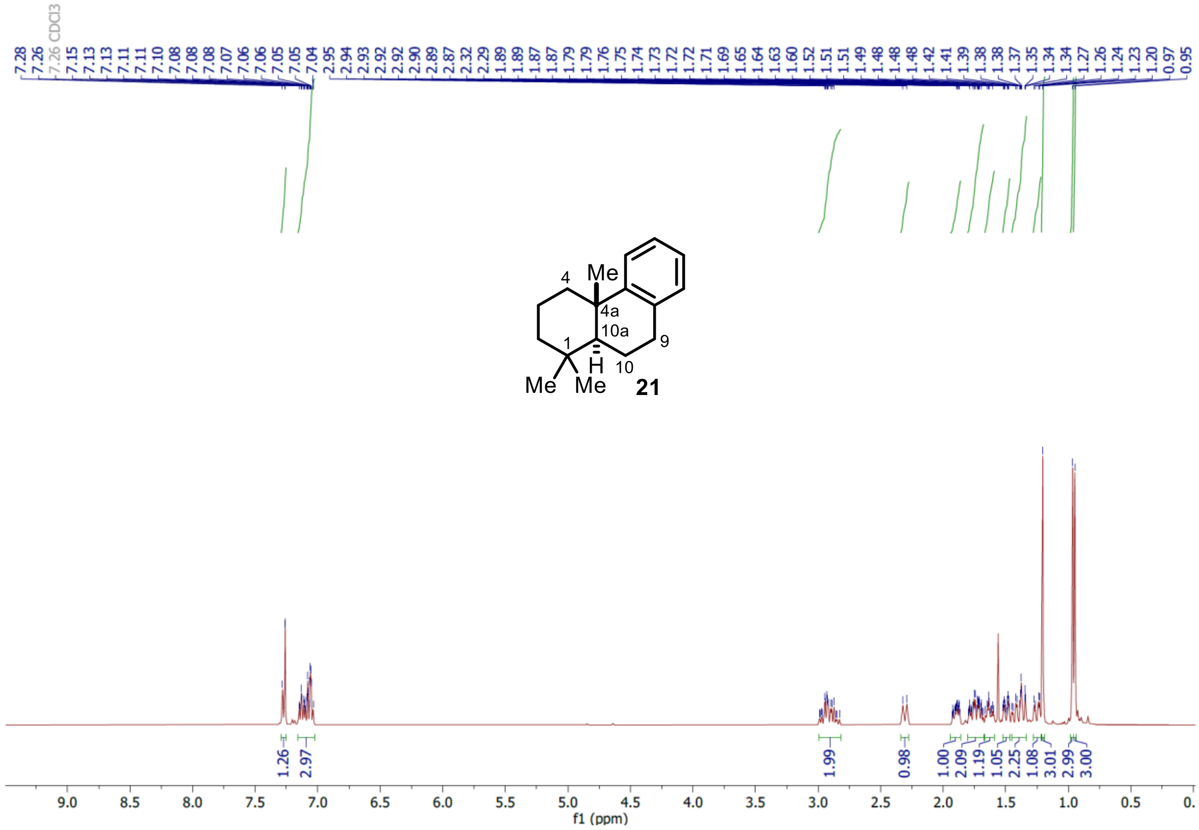
7.28 7.26 7.26 CDCl3 7.15 7.13 7.13 7.11 7.10 7.08 7.08 7.08 7.07 7.06 7.06 7.05 7.05 7.04 2.95 2.94 2.93 2.92 2.92 2.90 2.89 2.89 2.87 2.32 2.29 1.89 1.89 1.87 1.87 1.79 1.79 1.76 1.75 1.74 1.73 1.72 1.72 1.71 1.69 1.65 1.64 1.63 1.60 1.52 1.51 1.51 1.49 1.48 1.48 1.48 1.42 1.41 1.39 1.38 1.38 1.37 1.35 1.34 1.34 1.27 1.26 1.24 1.23 1.20 0.97 0.95
Me
4
4a
10a
1
9
10
H
Me
Me
21
1.26 2.97 1.99 0.98 1.00 2.09 1.19 1.05 2.25 1.08 3.01 2.99 3.00
f1 (ppm)

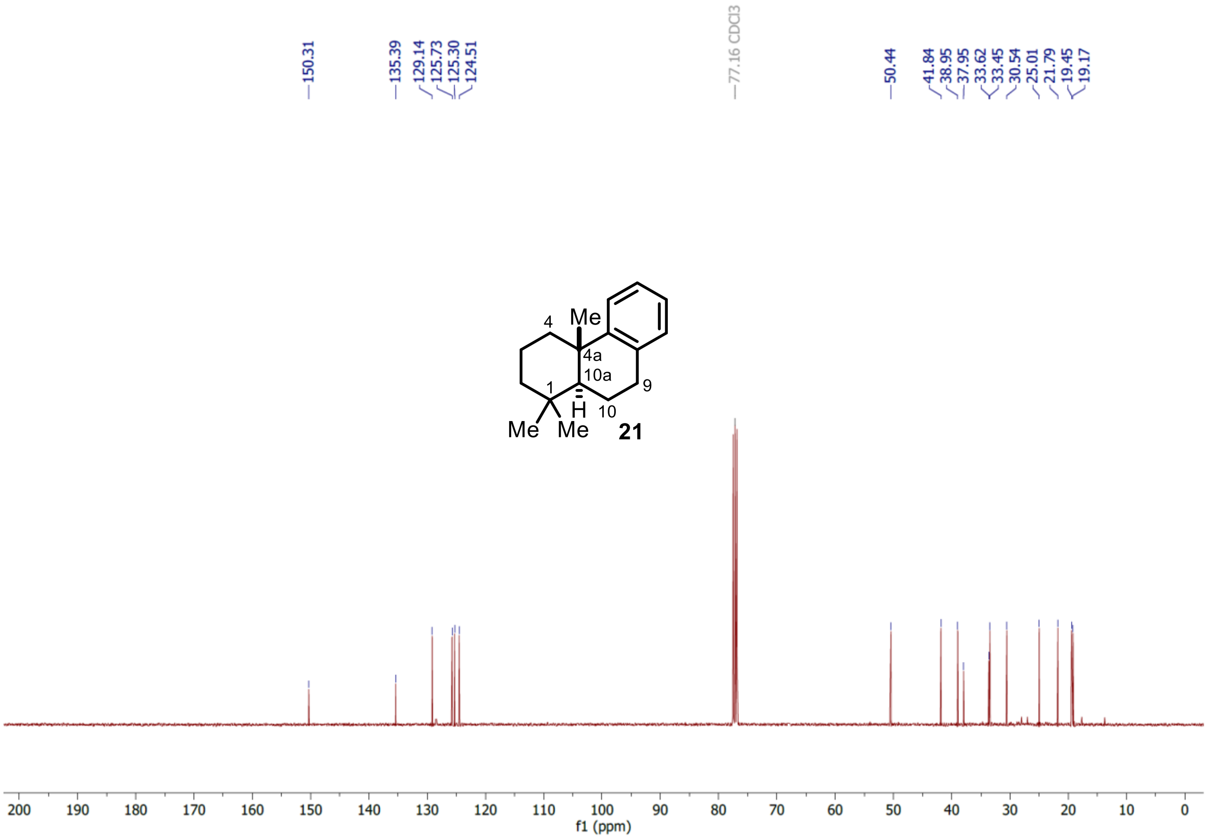
150.31 135.39 129.14 125.73 125.30 124.51 77.16 CDCl3 50.44 41.84 38.95 37.95 33.62 33.45 30.54 25.01 21.79 19.45 19.17
Me
4
4a
10a
1
9
10
H
Me
Me
21
f1 (ppm)

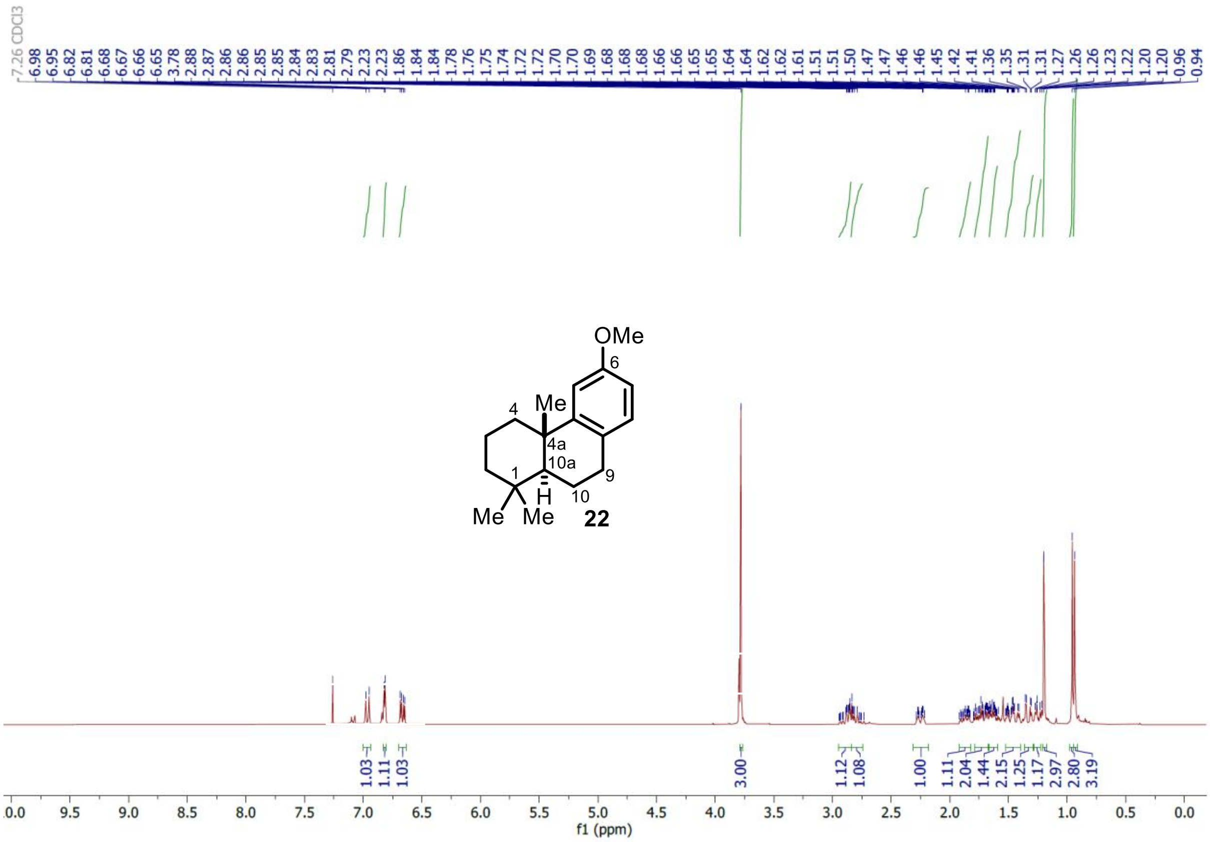

*4,4'-dimethoxybibenzyl

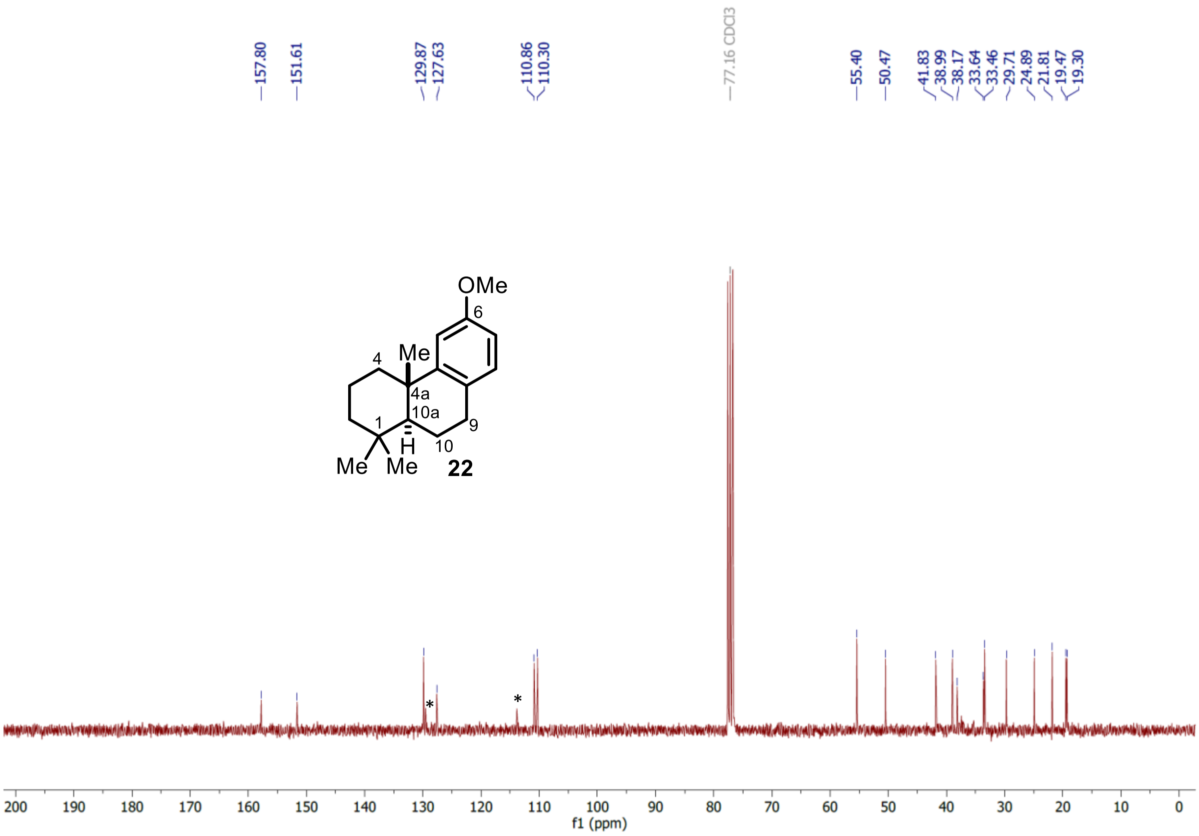

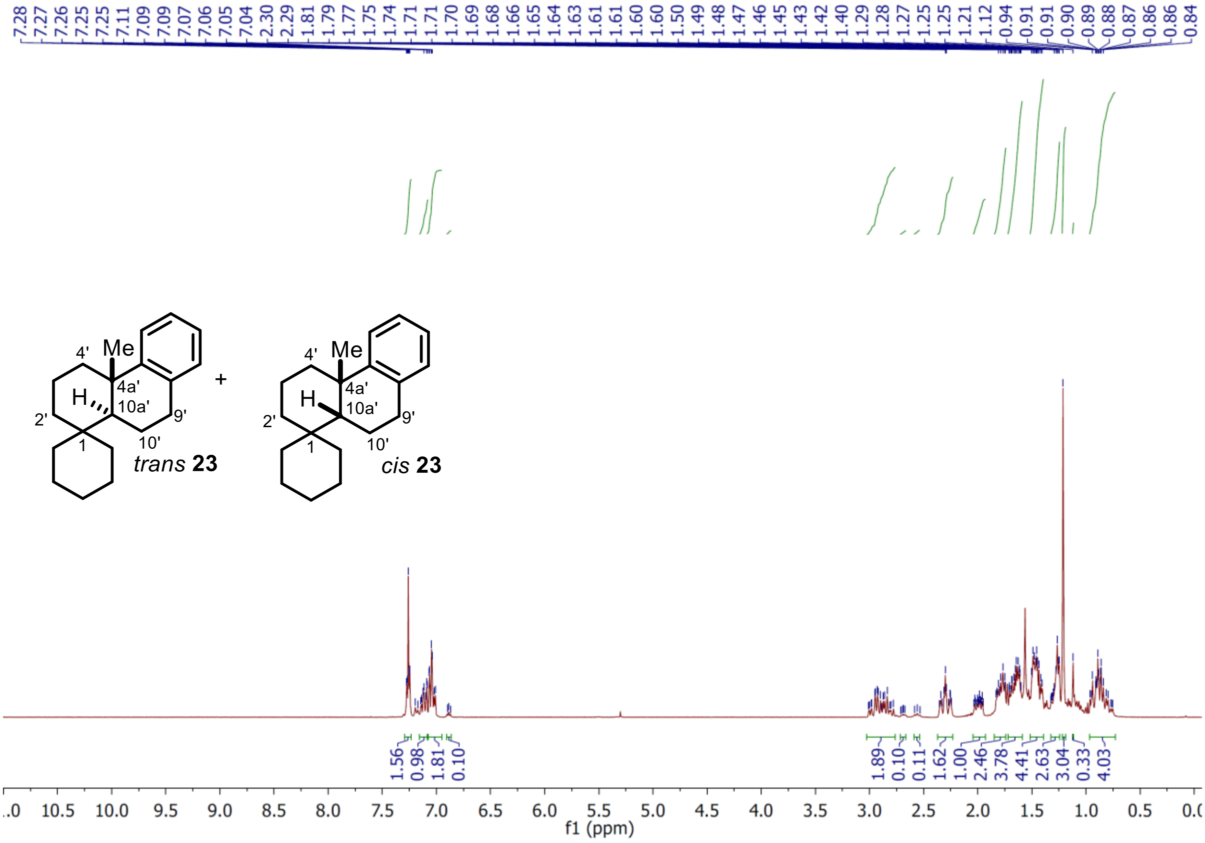
7.28 7.27 7.26 7.25 7.25 7.11 7.09 7.09 7.07 7.06 7.05 7.04 2.30 2.29 1.81 1.79 1.77 1.75 1.74 1.71 1.71 1.70 1.69 1.68 1.66 1.65 1.64 1.63 1.61 1.61 1.60 1.60 1.50 1.49 1.48 1.47 1.46 1.45 1.43 1.42 1.40 1.29 1.28 1.27 1.25 1.25 1.21 1.12 0.94 0.91 0.91 0.90 0.89 0.88 0.87 0.86 0.84
Me
4'
4a'
10a'
2'
9'
10'
1
H
+
trans **23**
cis **23**
1.56 0.98 1.81 0.10 1.89 0.10 0.11 1.62 1.00 2.46 3.78 4.41 2.63 3.04 0.33 4.03
f1 (ppm)

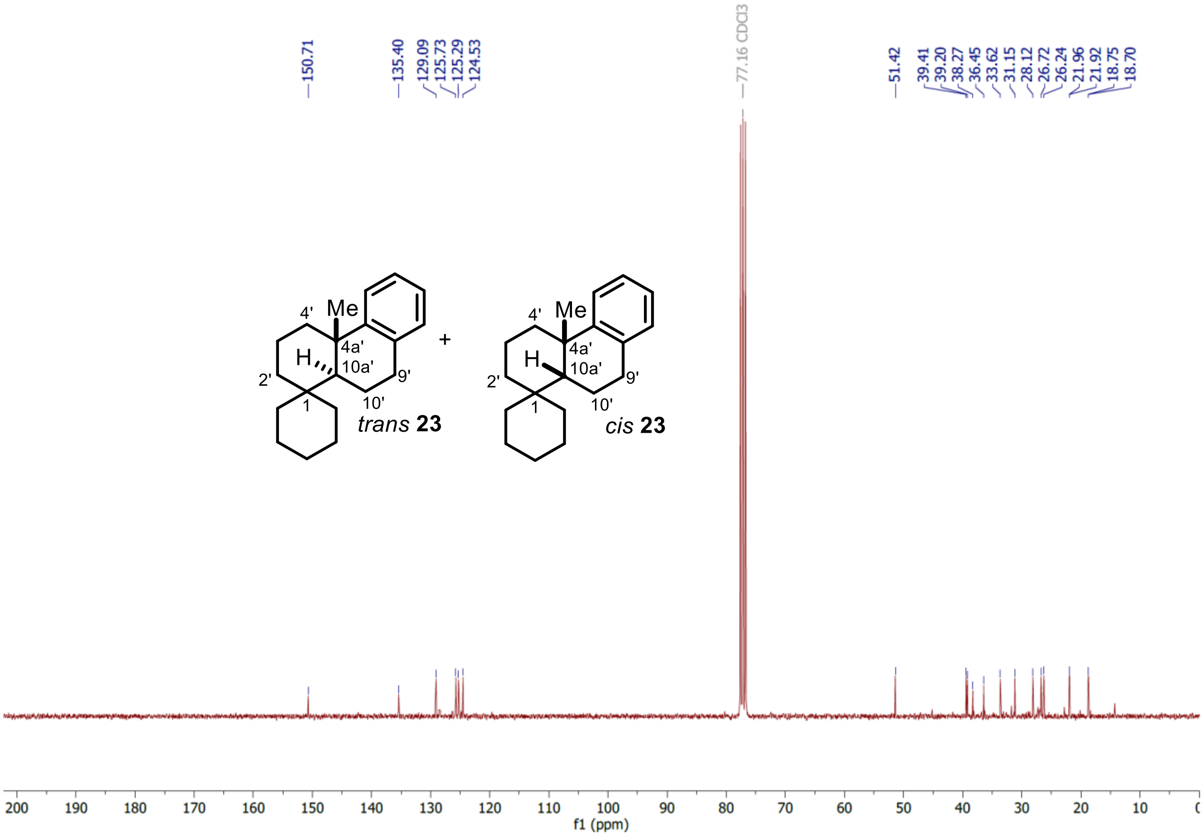
150.71
135.40 129.09 125.73 125.29 124.53
77.16 CDCl3
51.42
39.41 39.20 38.27 36.45 33.62 31.15 28.12 26.72 26.24 21.96 21.92 18.75 18.70
Me
4'
4a'
10a'
2'
9'
10'
1
H
+
trans **23**
cis **23**
f1 (ppm)

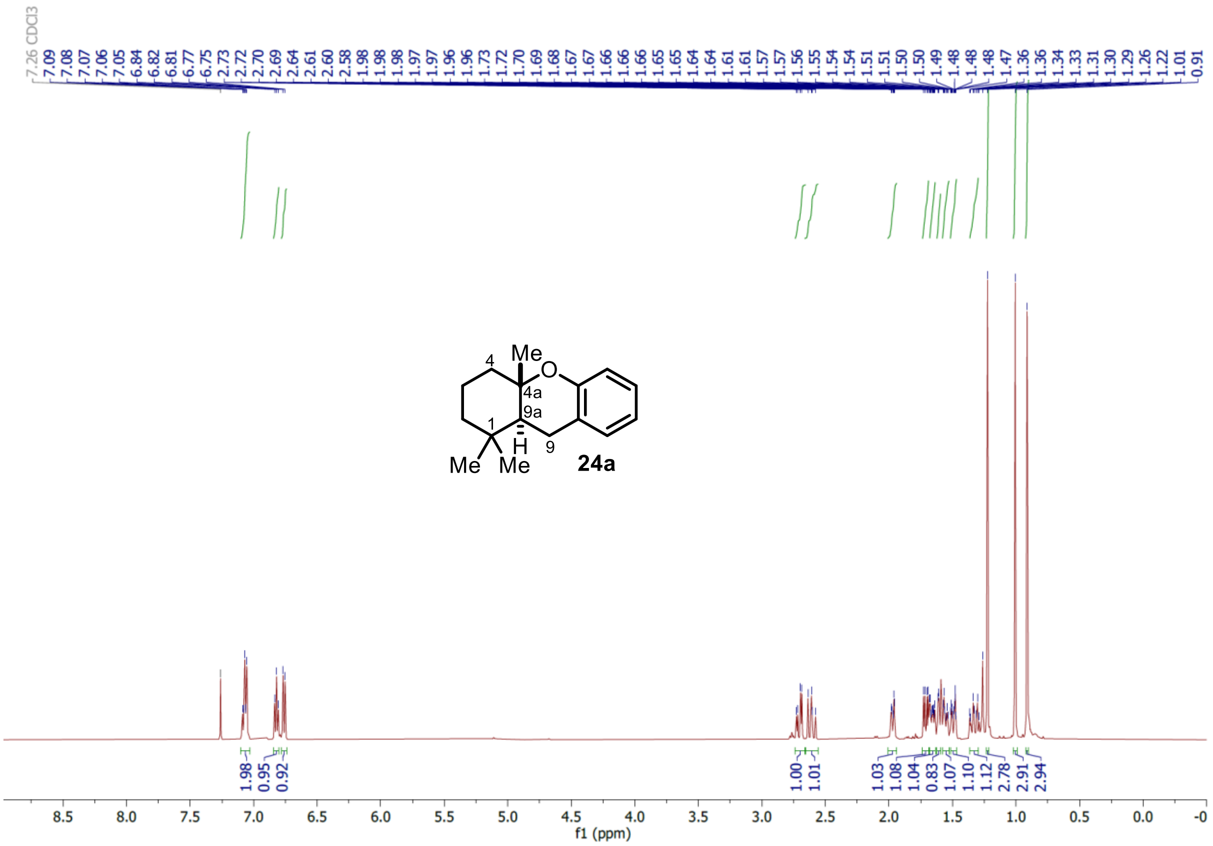

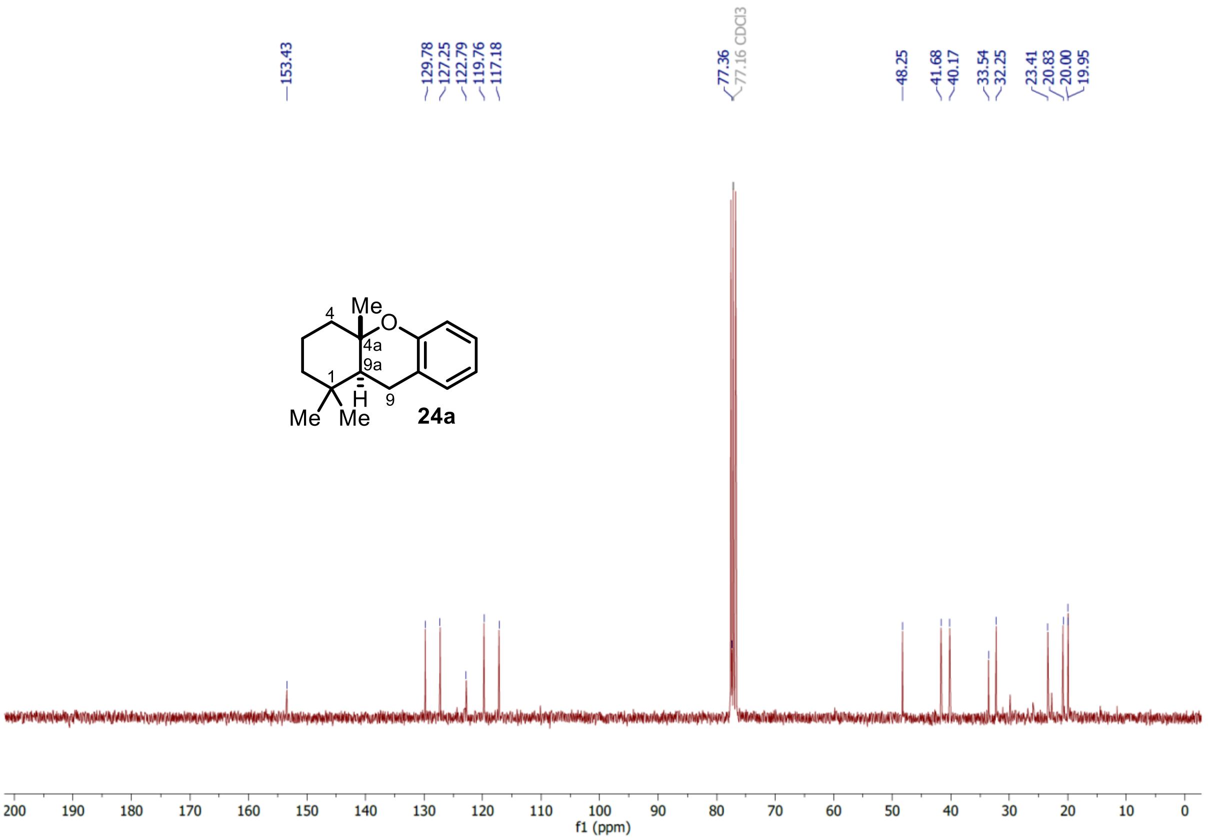

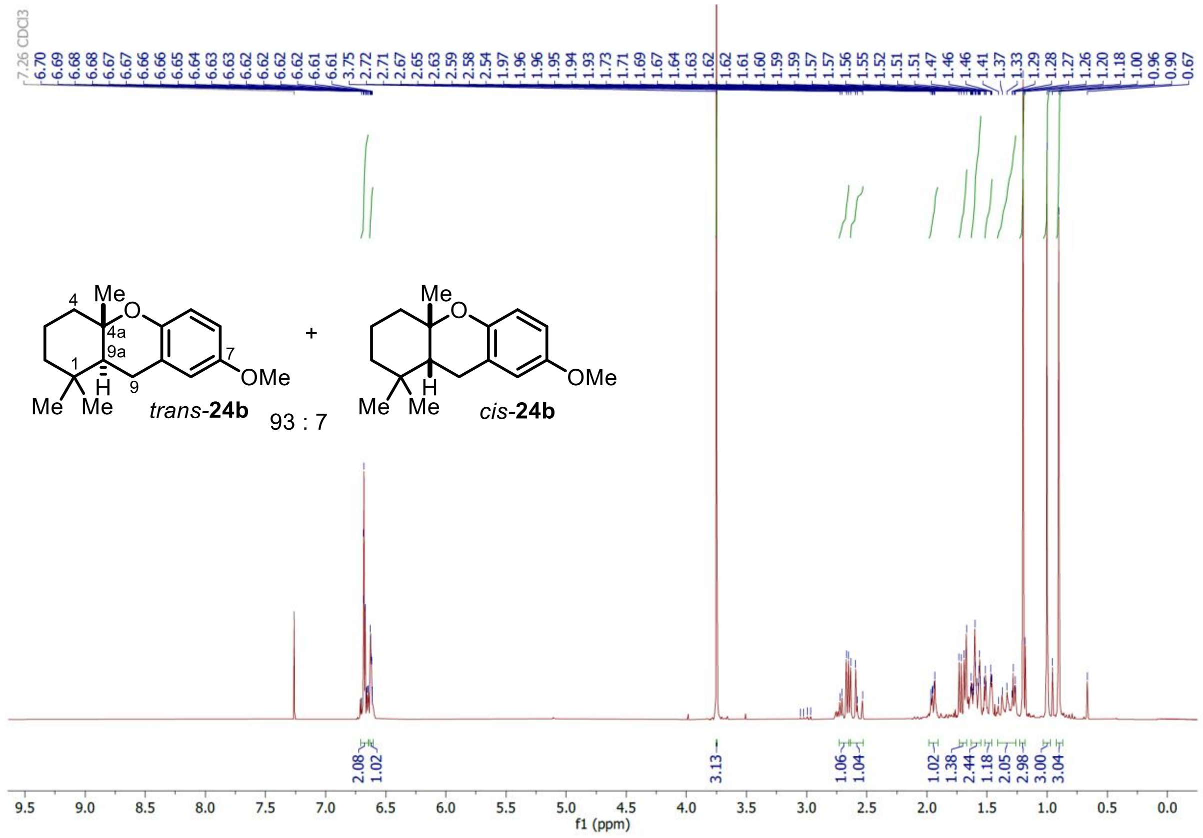
7.26 CDCl3
6.70
6.69
6.68
6.68
6.67
6.67
6.66
6.66
6.65
6.64
6.63
6.63
6.62
6.62
6.62
6.62
6.61
6.61
3.75
2.72
2.71
2.67
2.65
2.63
2.59
2.58
2.54
1.97
1.96
1.96
1.95
1.94
1.93
1.73
1.71
1.69
1.67
1.64
1.63
1.62
1.62
1.61
1.60
1.59
1.59
1.57
1.57
1.56
1.55
1.52
1.51
1.51
1.47
1.46
1.46
1.41
1.37
1.33
1.29
1.28
1.27
1.26
1.20
1.18
1.00
0.96
0.90
0.67
4
Me
O
4a
9a
7
OMe
1
H
9
Me
Me
trans-24b
+
cis-24b
93 : 7
2.08
1.02
3.13
1.06
1.04
1.02
1.38
2.44
1.18
2.05
2.98
3.00
3.04
f1 (ppm)

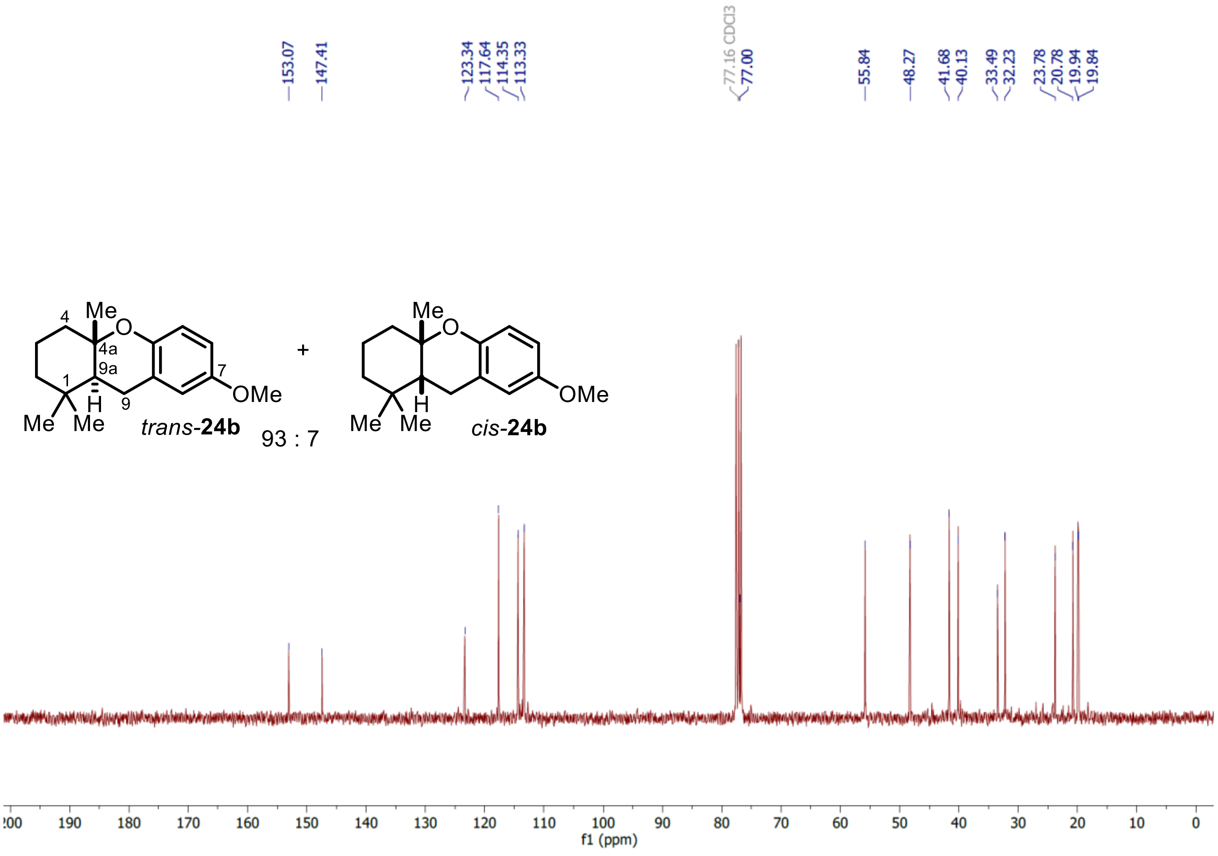
153.07
147.41
123.34
117.64
114.35
113.33
77.16 CDCl3
77.00
55.84
48.27
41.68
40.13
33.49
32.23
23.78
20.78
19.94
19.84
4
Me
O
4a
9a
7
OMe
1
H
9
Me
Me
trans-24b
+
cis-24b
93 : 7
f1 (ppm)

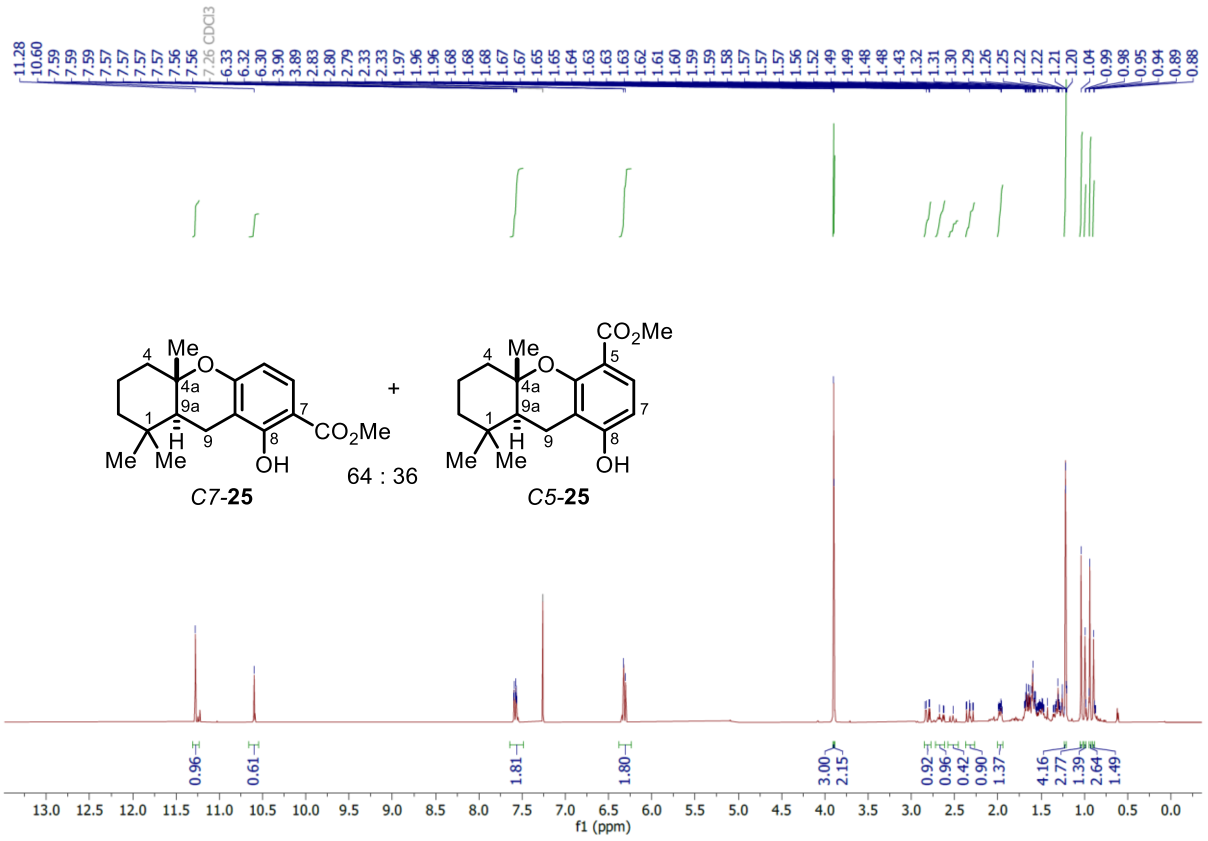

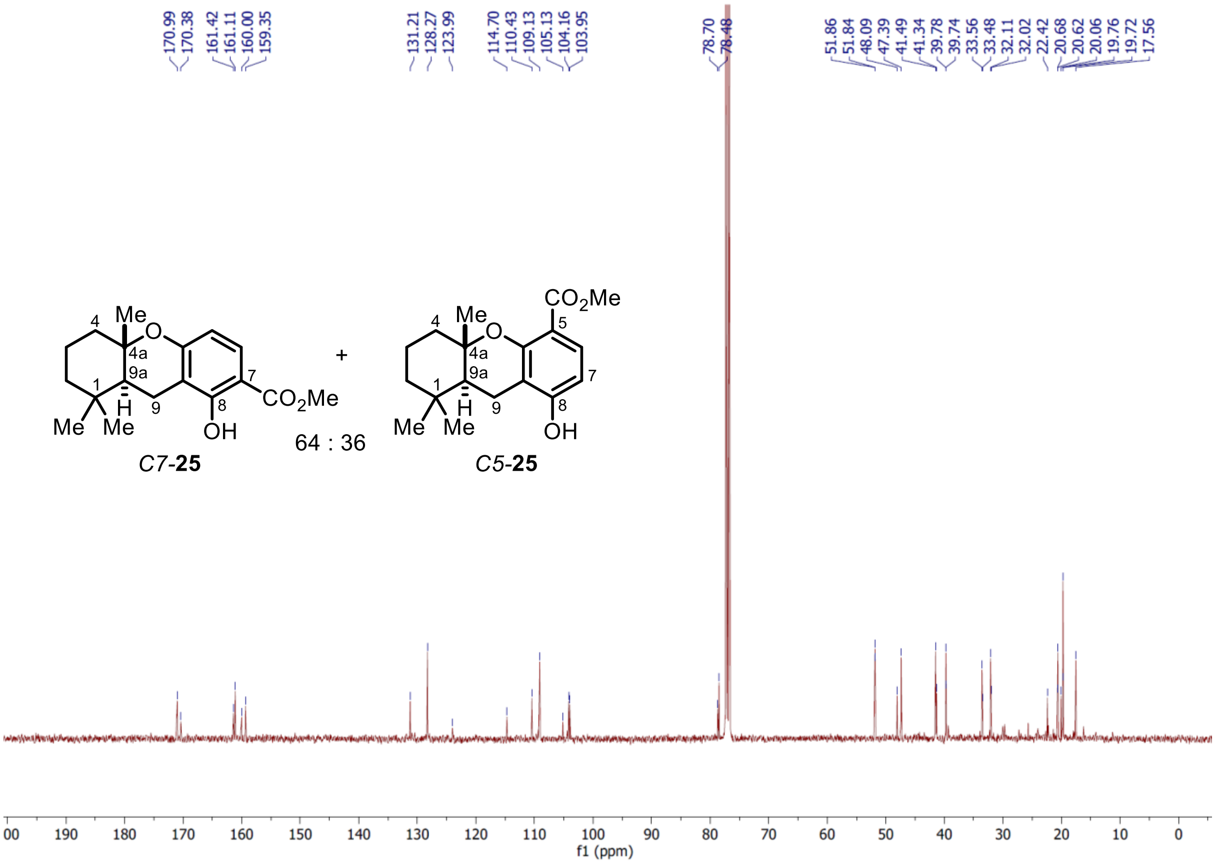

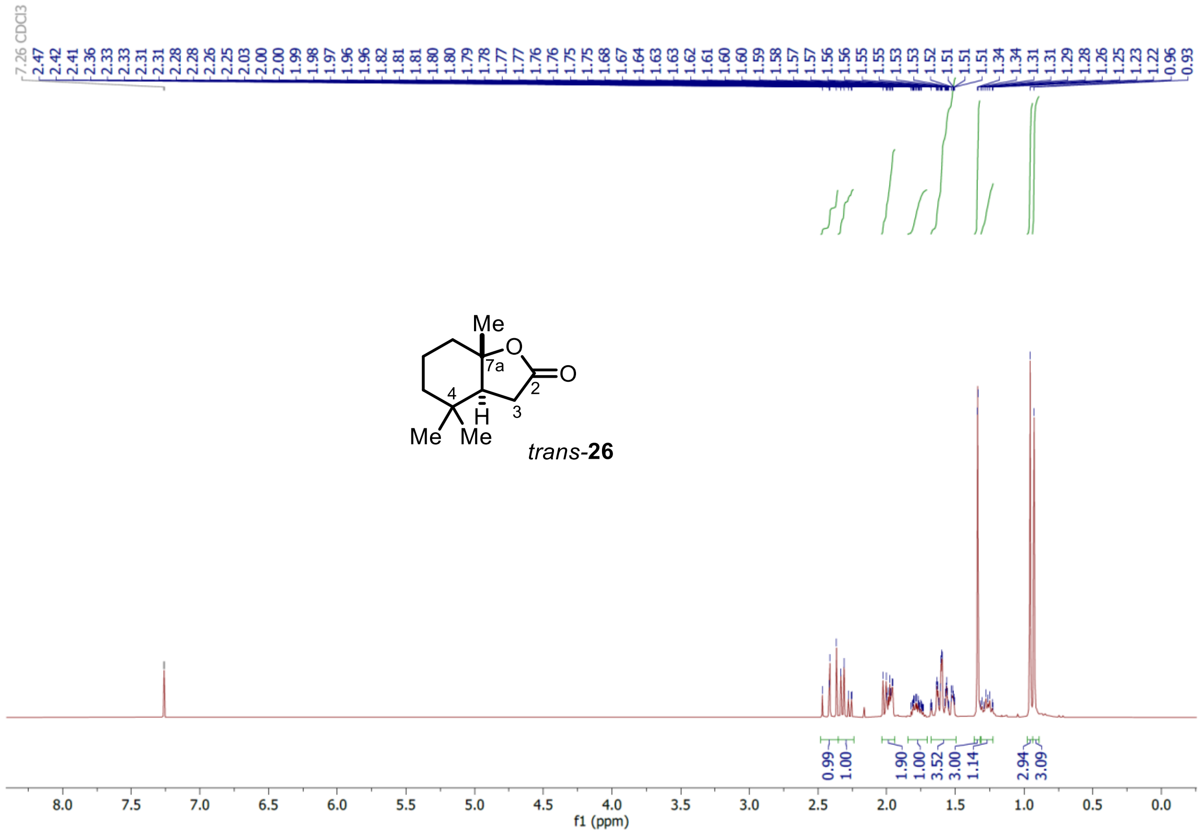
7.26 CDCl3
2.47 2.42 2.41 2.36 2.33 2.33 2.31 2.31 2.28 2.28 2.26 2.25 2.03 2.00 2.00 1.99 1.98 1.97 1.96 1.96 1.82 1.81 1.81 1.80 1.80 1.79 1.78 1.77 1.77 1.76 1.76 1.75 1.75 1.68 1.67 1.64 1.63 1.63 1.62 1.61 1.60 1.60 1.59 1.58 1.57 1.57 1.56 1.56 1.55 1.55 1.53 1.53 1.52 1.51 1.51 1.51 1.34 1.34 1.31 1.31 1.29 1.28 1.26 1.25 1.23 1.22 0.96 0.93
Me
O
7a
2
O
4
H
3
Me Me
trans-26
0.99 1.00 1.90 1.00 3.52 3.00 1.14 2.94 3.09
f1 (ppm)

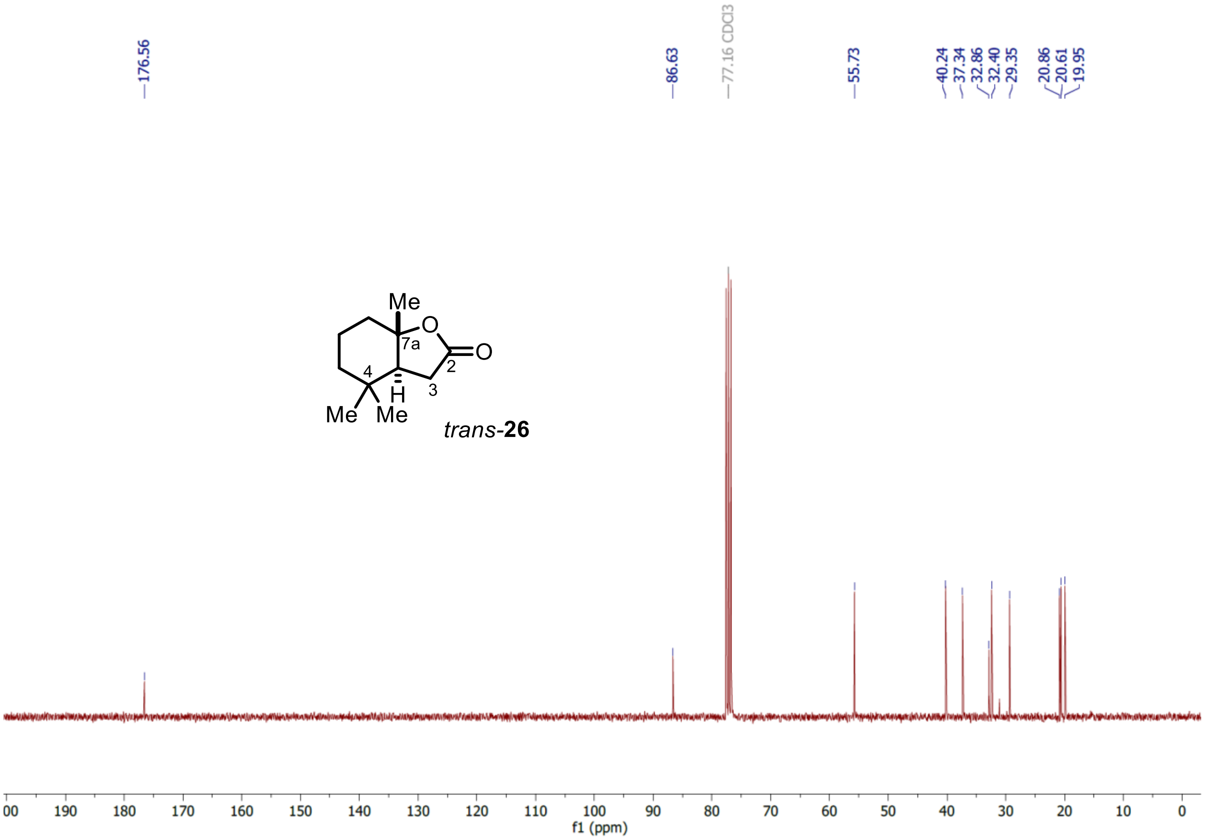
176.56
86.63
77.16 CDCl3
55.73
40.24 37.34 32.86 32.40 29.35
20.86 20.61 19.95
Me
O
7a
2
O
4
H
3
Me Me
trans-26
f1 (ppm)

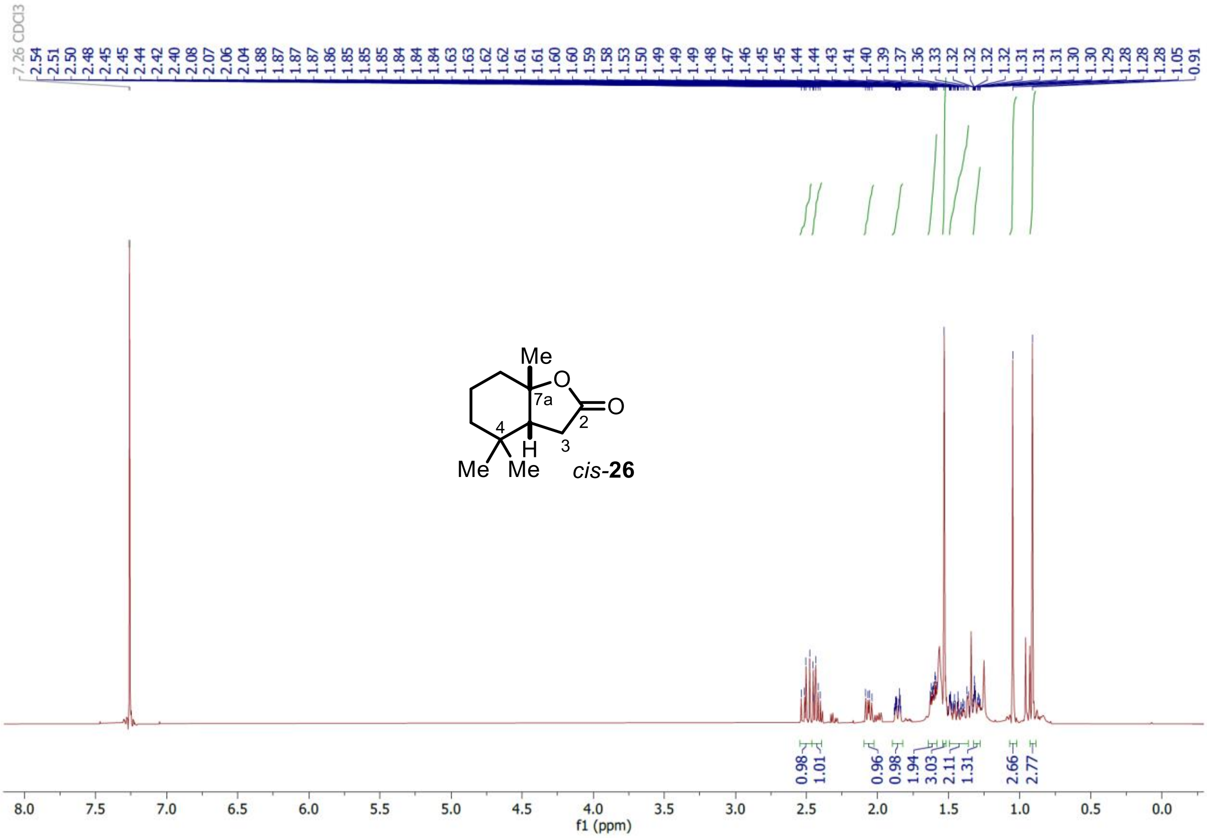
7.26 CDCl3
2.54 2.51 2.50 2.48 2.45 2.45 2.44 2.42 2.40 2.08 2.07 2.06 2.04 1.88 1.87 1.87 1.86 1.85 1.85 1.85 1.84 1.84 1.84 1.63 1.63 1.62 1.62 1.61 1.61 1.60 1.60 1.59 1.58 1.53 1.50 1.49 1.49 1.49 1.48 1.47 1.46 1.45 1.45 1.44 1.44 1.43 1.41 1.40 1.39 1.37 1.36 1.33 1.32 1.32 1.32 1.32 1.31 1.31 1.30 1.30 1.29 1.28 1.28 1.28 1.05 0.91
Me
O
7a
O
2
4
H
3
Me
Me
cis-26
0.98
1.01
0.96
0.98
1.94
3.03
2.11
1.31
2.66
2.77
f1 (ppm)

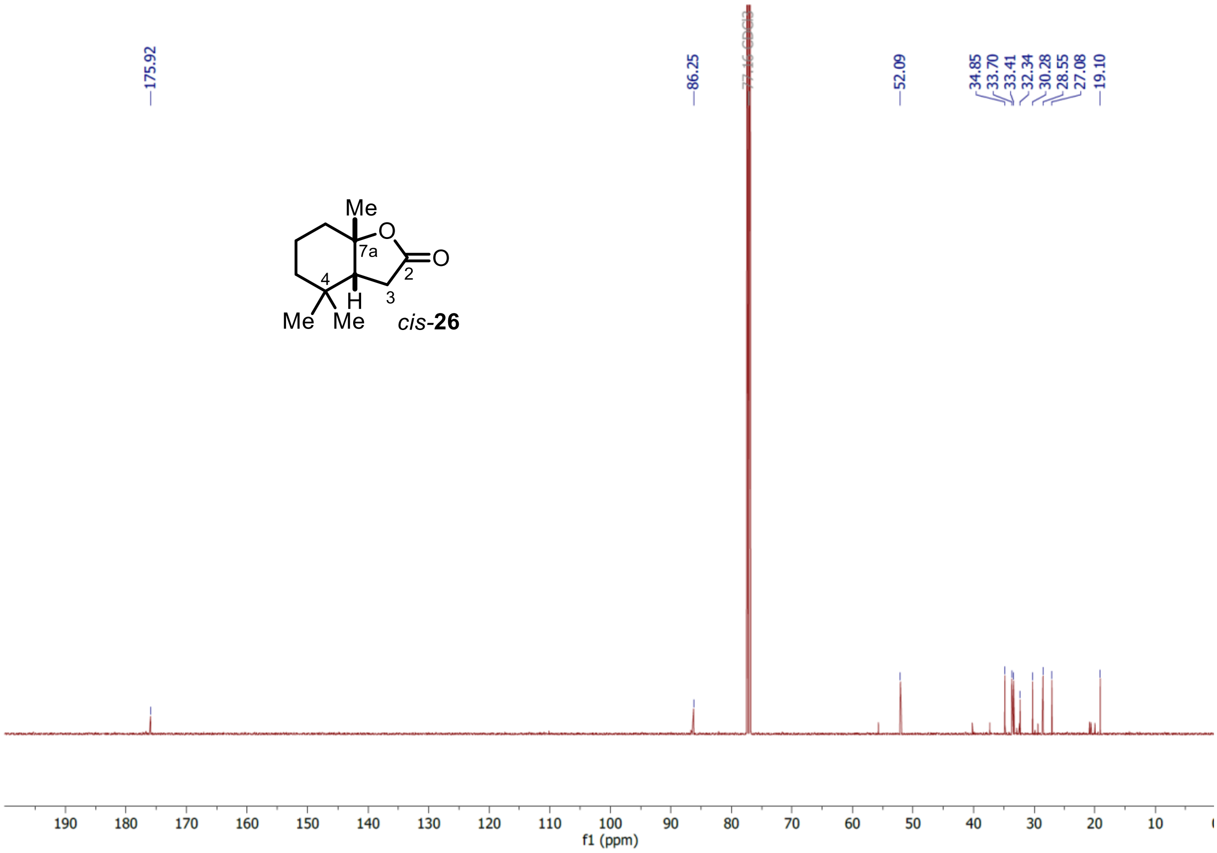
175.92
86.25
77.16 CDCl3
52.09
34.85 33.70 33.41 32.34 30.28 28.55 27.08
19.10
Me
O
7a
O
2
4
H
3
Me
Me
cis-26
f1 (ppm)

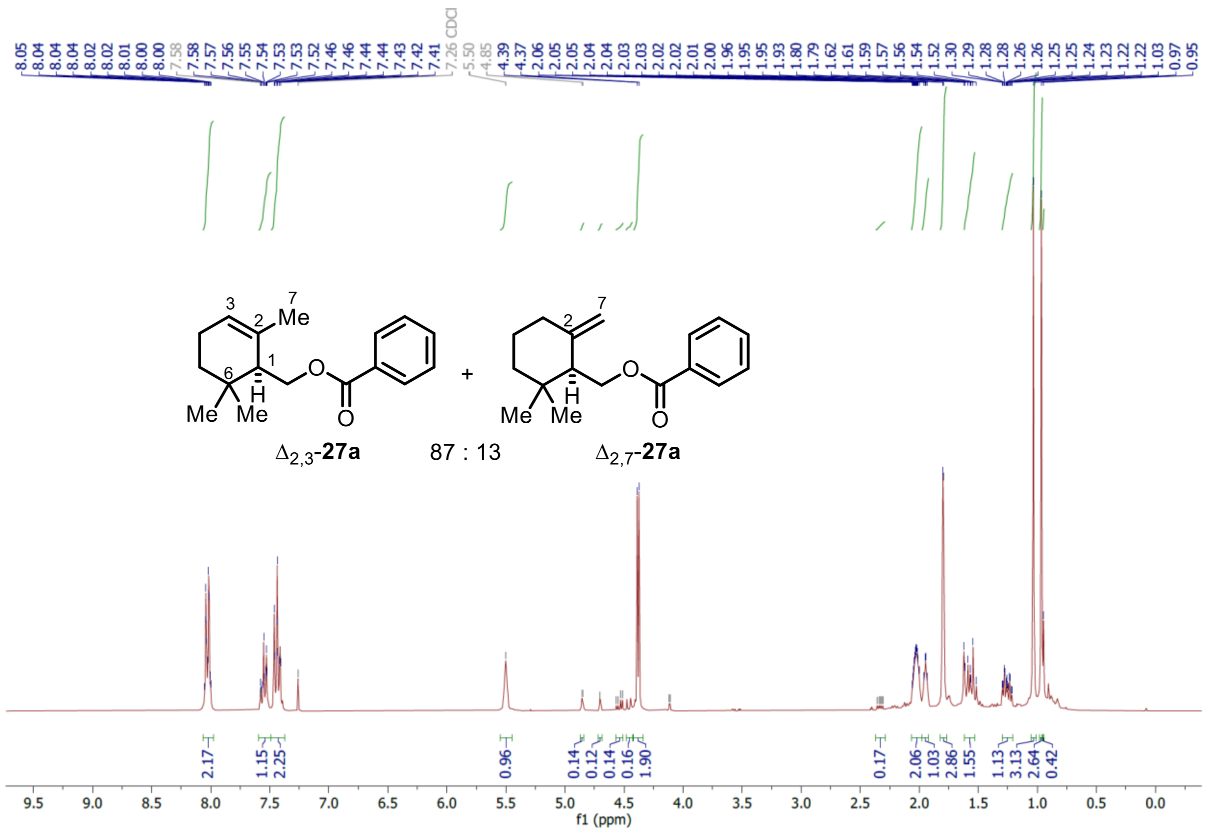
Δ2,3-27a
87 : 13
Δ2,7-27a
f1 (ppm)

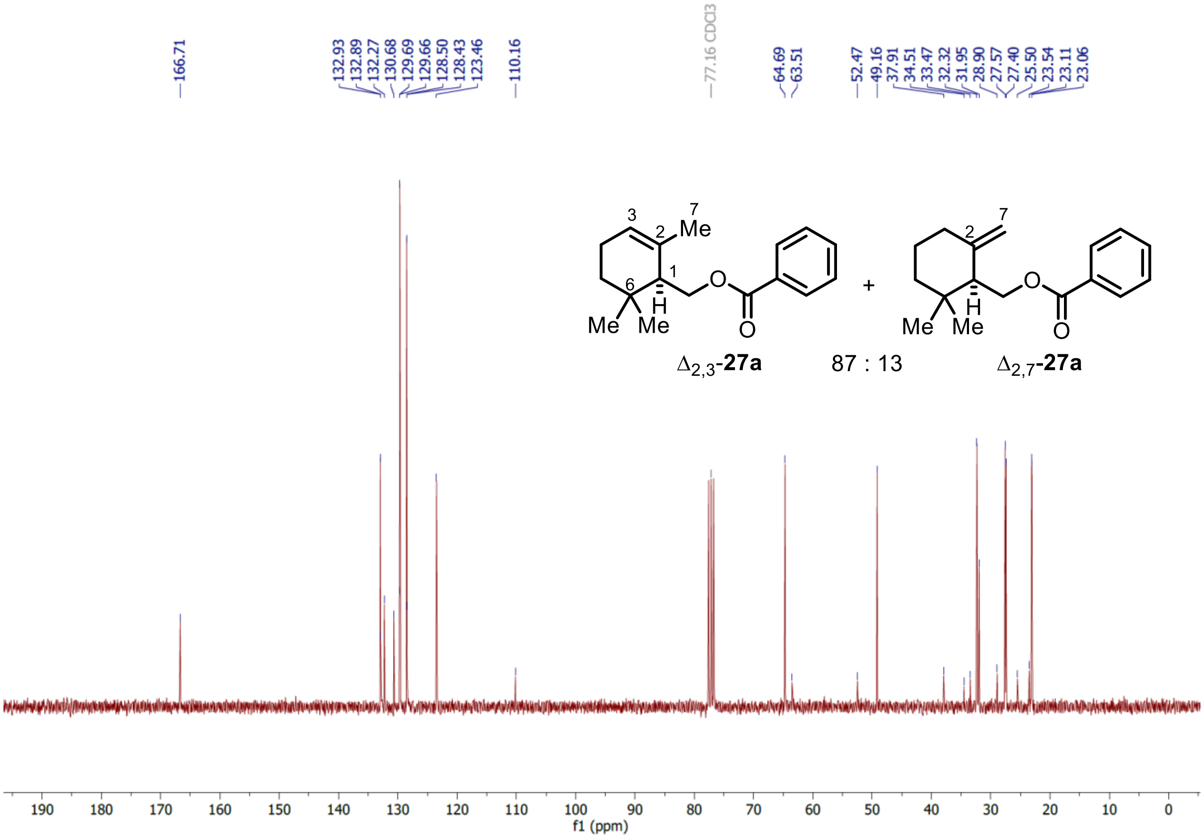
Δ2,3-27a
87 : 13
Δ2,7-27a
f1 (ppm)

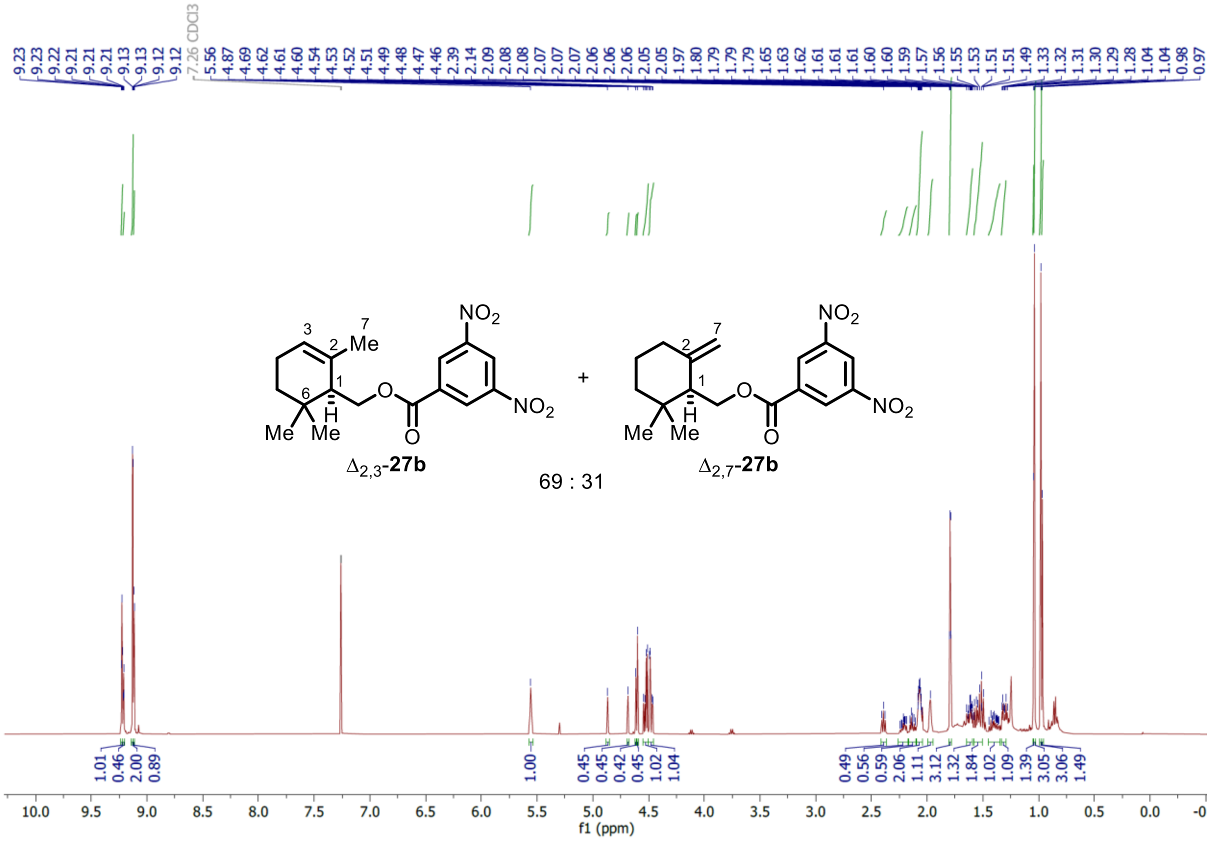

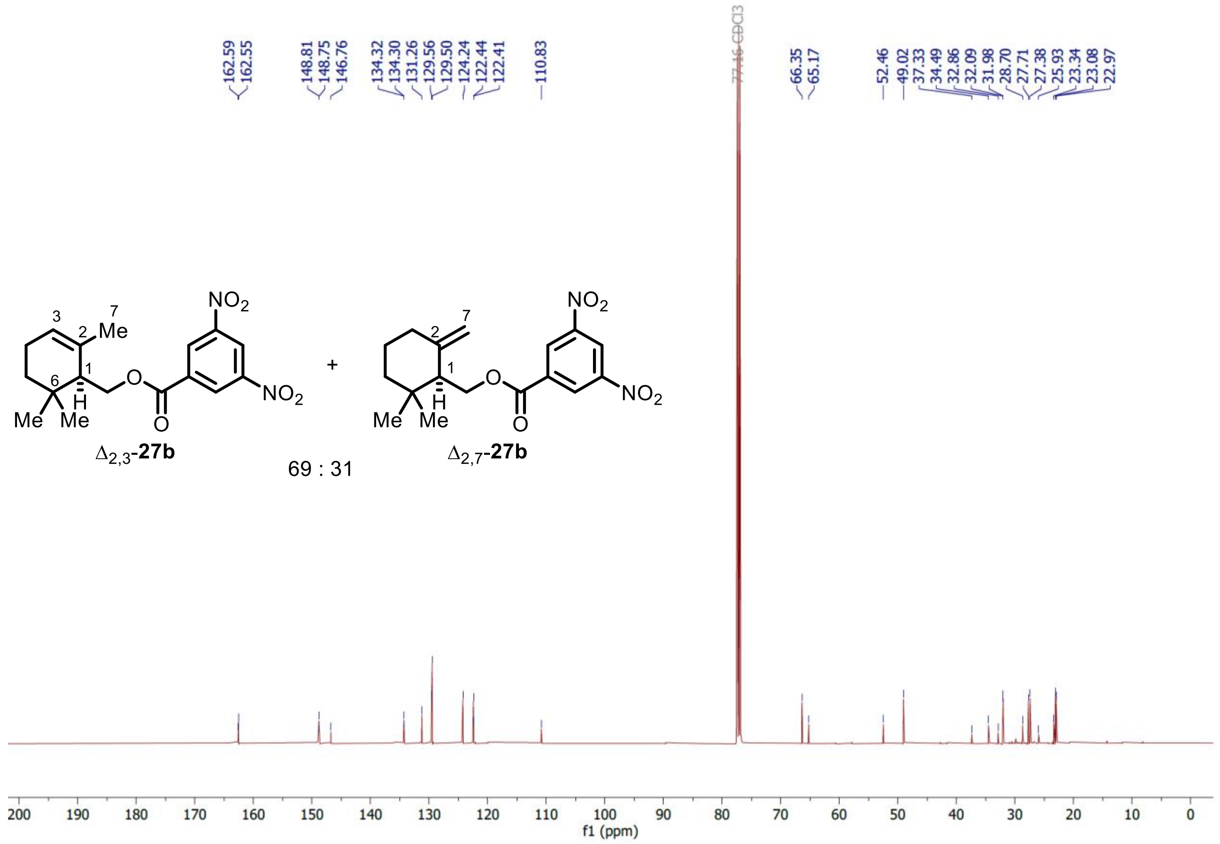

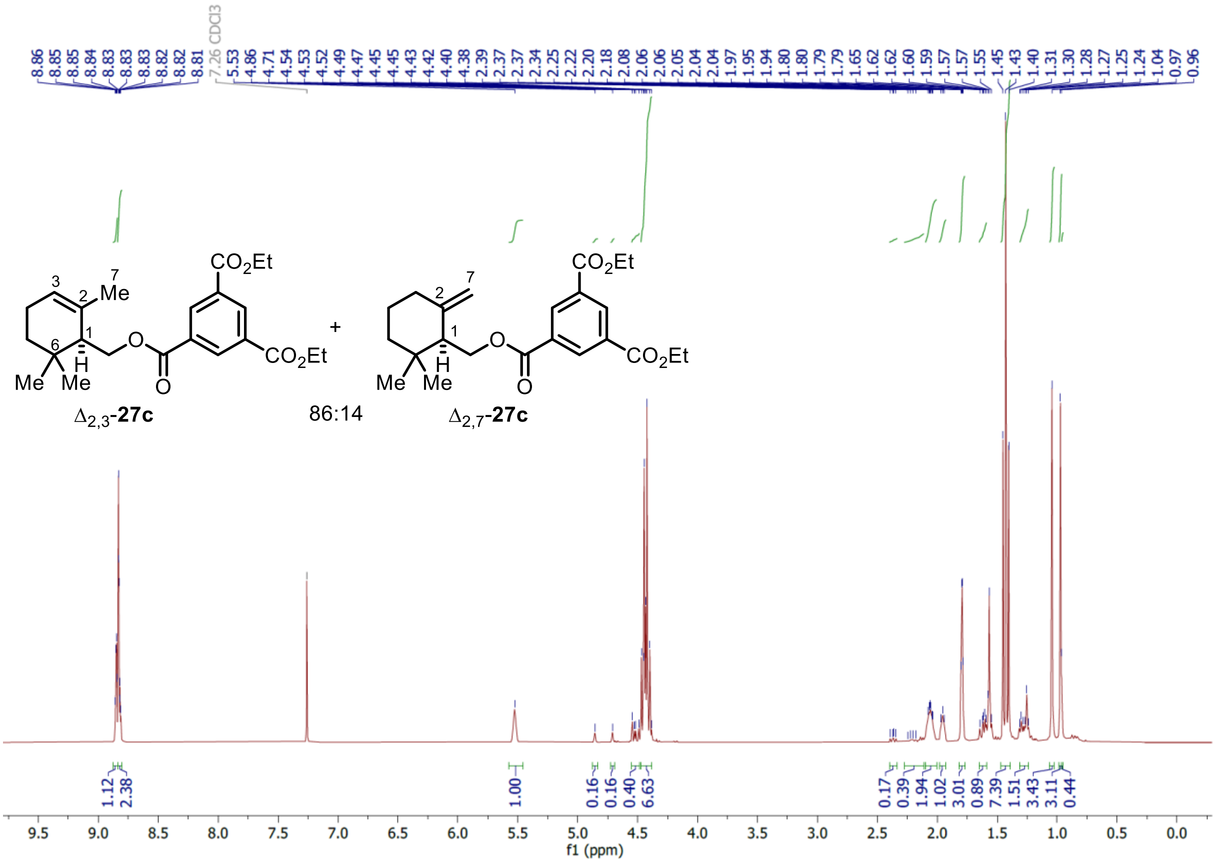
8.86 8.85 8.85 8.84 8.83 8.83 8.83 8.83 8.82 8.82 8.81
7.26 CDCl3
5.53 4.86 4.71 4.54 4.53 4.52 4.49 4.47 4.45 4.45 4.43 4.42 4.40 4.38 2.39 2.37 2.37 2.34 2.25 2.22 2.20 2.18 2.08 2.06 2.06 2.05 2.04 2.04 1.97 1.95 1.94 1.80 1.80 1.79 1.65 1.62 1.62 1.60 1.59 1.57 1.57 1.55 1.45 1.43 1.40 1.31 1.30 1.28 1.27 1.25 1.24 1.04 0.97 0.96
CO2Et
Me
CO2Et
+
Me Me
Δ2,3-27c
86:14
Δ2,7-27c
1.12 2.38 1.00 0.16 0.16 0.40 6.63 0.17 0.39 1.94 1.02 3.01 0.89 7.39 1.51 3.43 3.11 0.44
f1 (ppm)

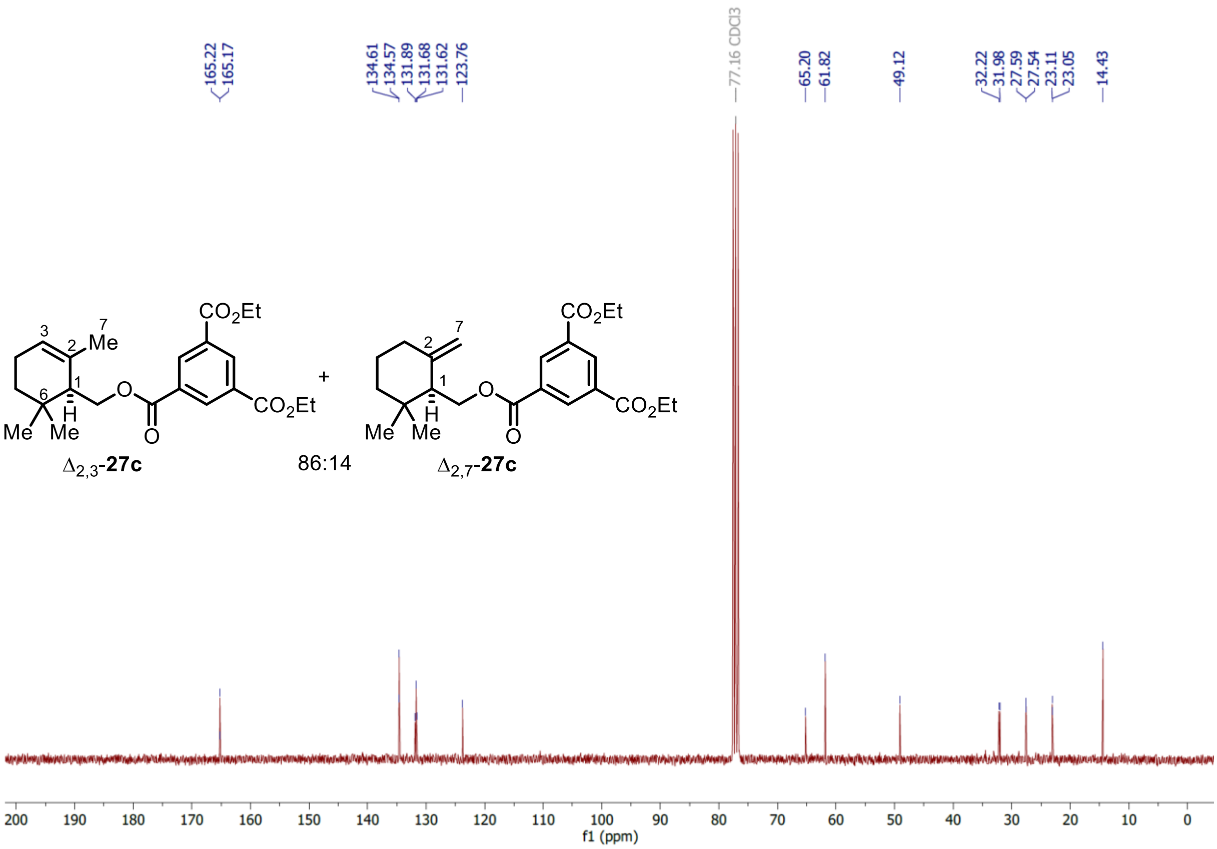
165.22 165.17
134.61 134.57 131.89 131.68 131.62 123.76
77.16 CDCl3
65.20 61.82 49.12 32.22 31.98 27.59 27.54 23.11 23.05 14.43
CO2Et
Me
CO2Et
+
Me Me
Δ2,3-27c
86:14
Δ2,7-27c
f1 (ppm)

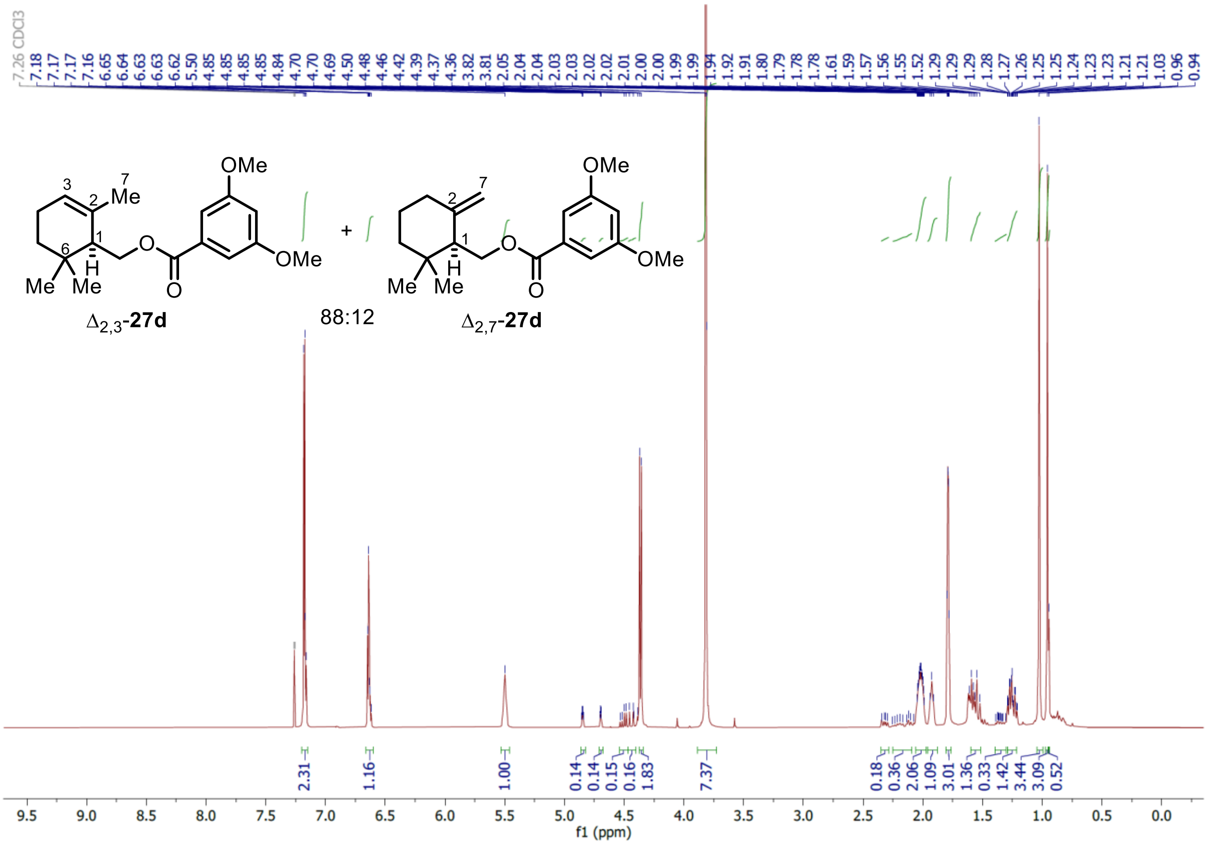
7.26 CDCl3
7.18 7.17 7.17 7.16 6.65 6.64 6.63 6.63 6.62 5.50 4.85 4.85 4.85 4.84 4.70 4.70 4.69 4.50 4.48 4.46 4.42 4.39 4.37 4.36 3.82 3.81 2.05 2.04 2.04 2.03 2.03 2.02 2.02 2.01 2.00 2.00 1.99 1.99 1.94 1.92 1.91 1.80 1.79 1.78 1.78 1.61 1.59 1.57 1.56 1.55 1.52 1.29 1.29 1.29 1.28 1.27 1.26 1.25 1.25 1.24 1.23 1.23 1.21 1.21 1.03 0.96 0.94
OMe
OMe
OMe
OMe
Me
Me Me
Me Me
H
H
O
O
O
O
+
$\Delta_{2,3}$-**27d**
88:12
$\Delta_{2,7}$-**27d**
2.31 1.16 1.00 0.14 0.14 0.15 0.16 1.83 7.37 0.18 0.36 2.06 1.09 3.01 1.36 0.33 1.42 3.44 3.09 0.52
9.5 9.0 8.5 8.0 7.5 7.0 6.5 6.0 5.5 5.0 4.5 4.0 3.5 3.0 2.5 2.0 1.5 1.0 0.5 0.0
f1 (ppm)

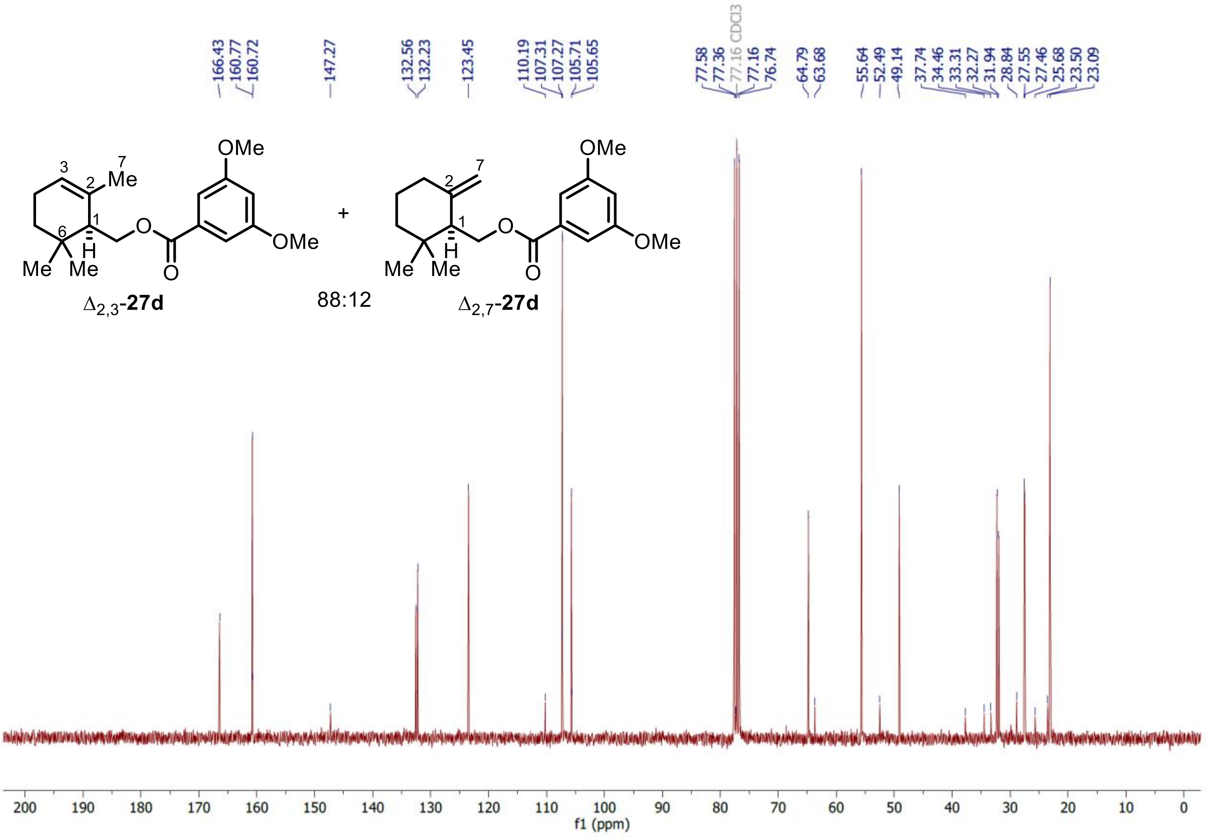
166.43 160.77 160.72 147.27 132.56 132.23 123.45 110.19 107.31 107.27 105.71 105.65 77.58 77.36 77.16 CDCl3 77.16 76.74 64.79 63.68 55.64 52.49 49.14 37.74 34.46 33.31 32.27 31.94 28.84 27.55 27.46 25.68 23.50 23.09
OMe
OMe
OMe
OMe
Me
Me Me
Me Me
H
H
O
O
O
O
+
$\Delta_{2,3}$-**27d**
88:12
$\Delta_{2,7}$-**27d**
200 190 180 170 160 150 140 130 120 110 100 90 80 70 60 50 40 30 20 10 0
f1 (ppm)

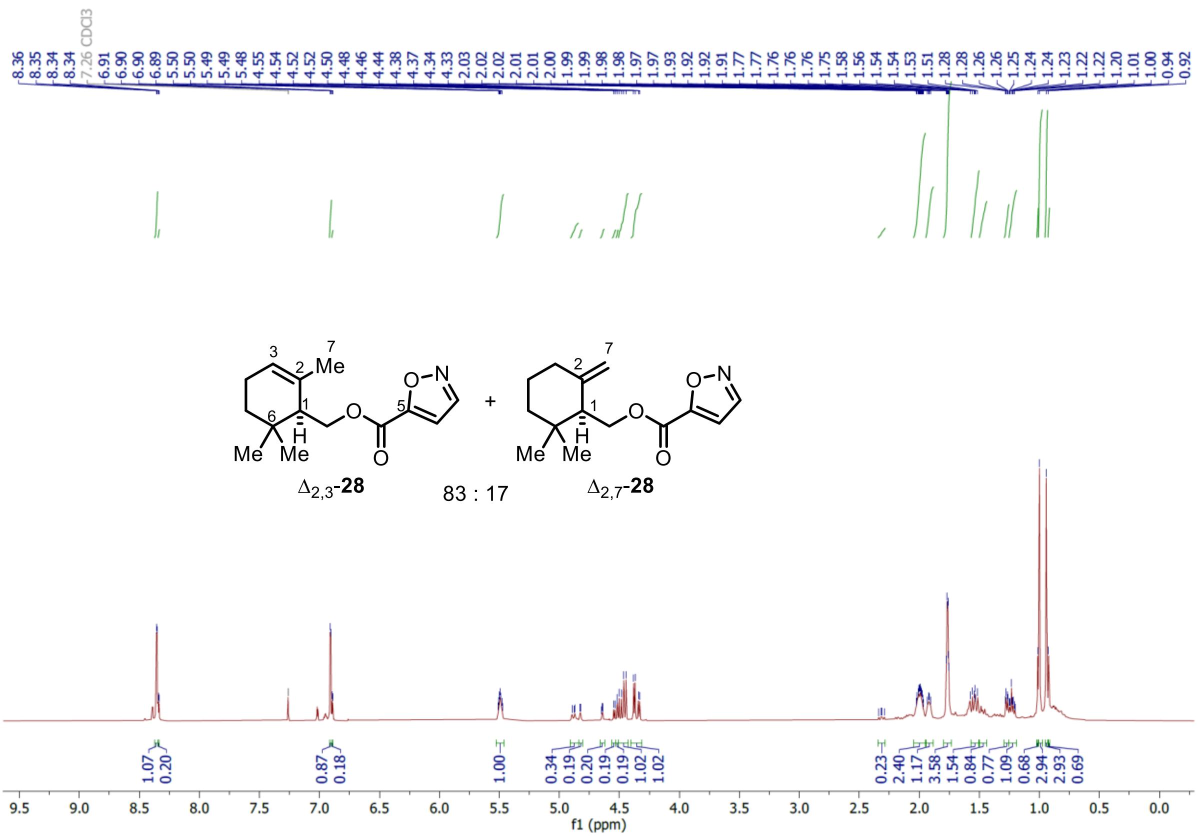
Δ2,3-28
83 : 17
Δ2,7-28
f1 (ppm)

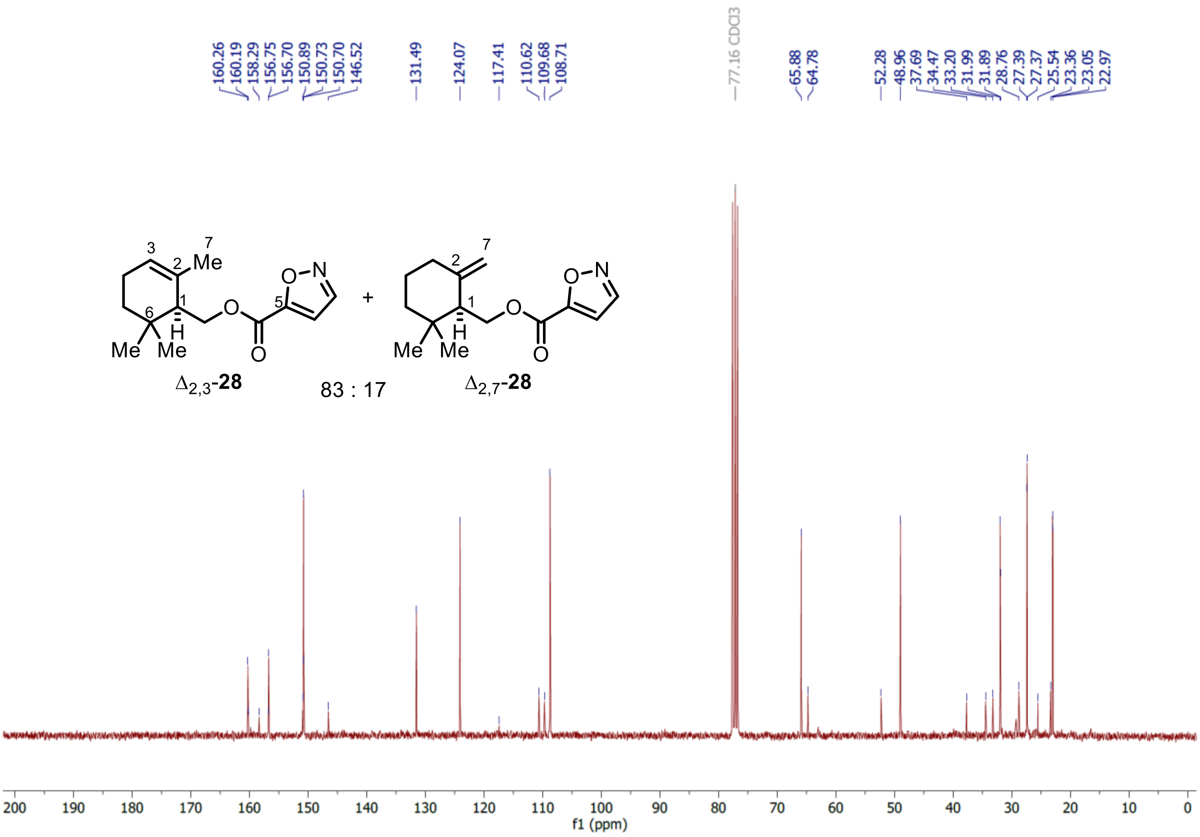
Δ2,3-28
83 : 17
Δ2,7-28
f1 (ppm)

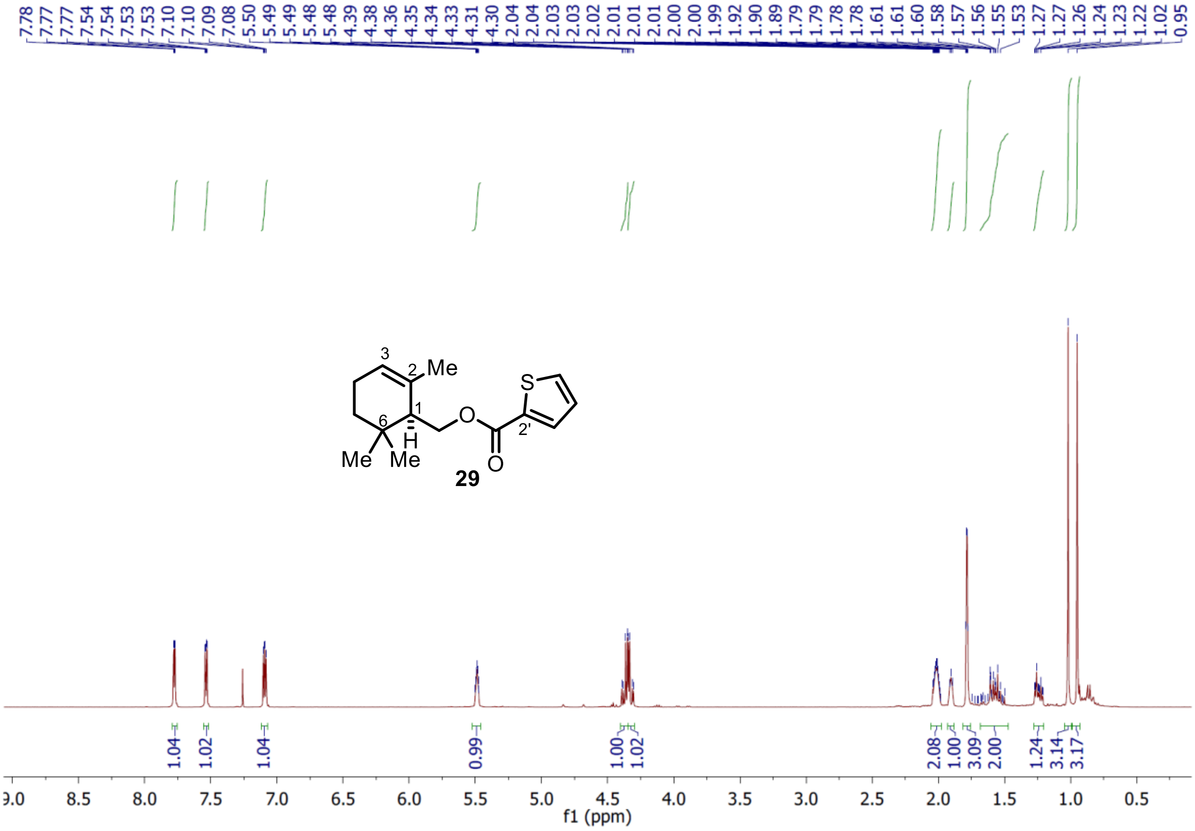
7.78 7.77 7.77 7.54 7.54 7.53 7.53 7.10 7.10 7.09 7.08 5.50 5.49 5.48 5.48 4.39 4.38 4.36 4.35 4.34 4.33 4.31 4.30 2.04 2.04 2.03 2.03 2.02 2.01 2.01 2.01 2.00 2.00 1.99 1.92 1.90 1.89 1.79 1.79 1.78 1.78 1.61 1.61 1.60 1.58 1.57 1.56 1.55 1.53 1.27 1.27 1.26 1.24 1.23 1.22 1.02 0.95
3
2
Me
1
6
H
Me
Me
S
2'
O
O
29
1.04 1.02 1.04 0.99 1.00 1.02 2.08 1.00 3.09 2.00 1.24 3.14 3.17
f1 (ppm)

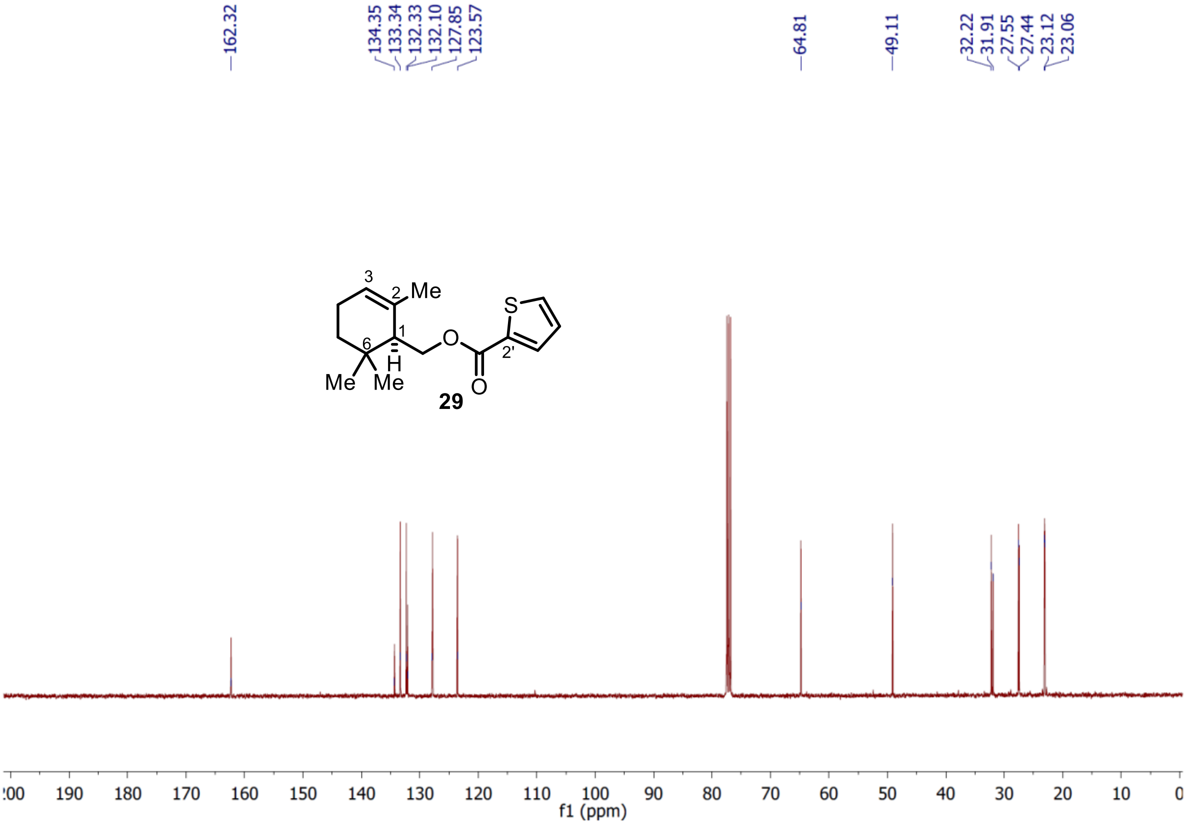
162.32
134.35 133.34 132.33 132.10 127.85 123.57
64.81
49.11
32.22 31.91 27.55 27.44 23.12 23.06
3
2
Me
1
6
H
Me
Me
S
2'
O
O
29
f1 (ppm)

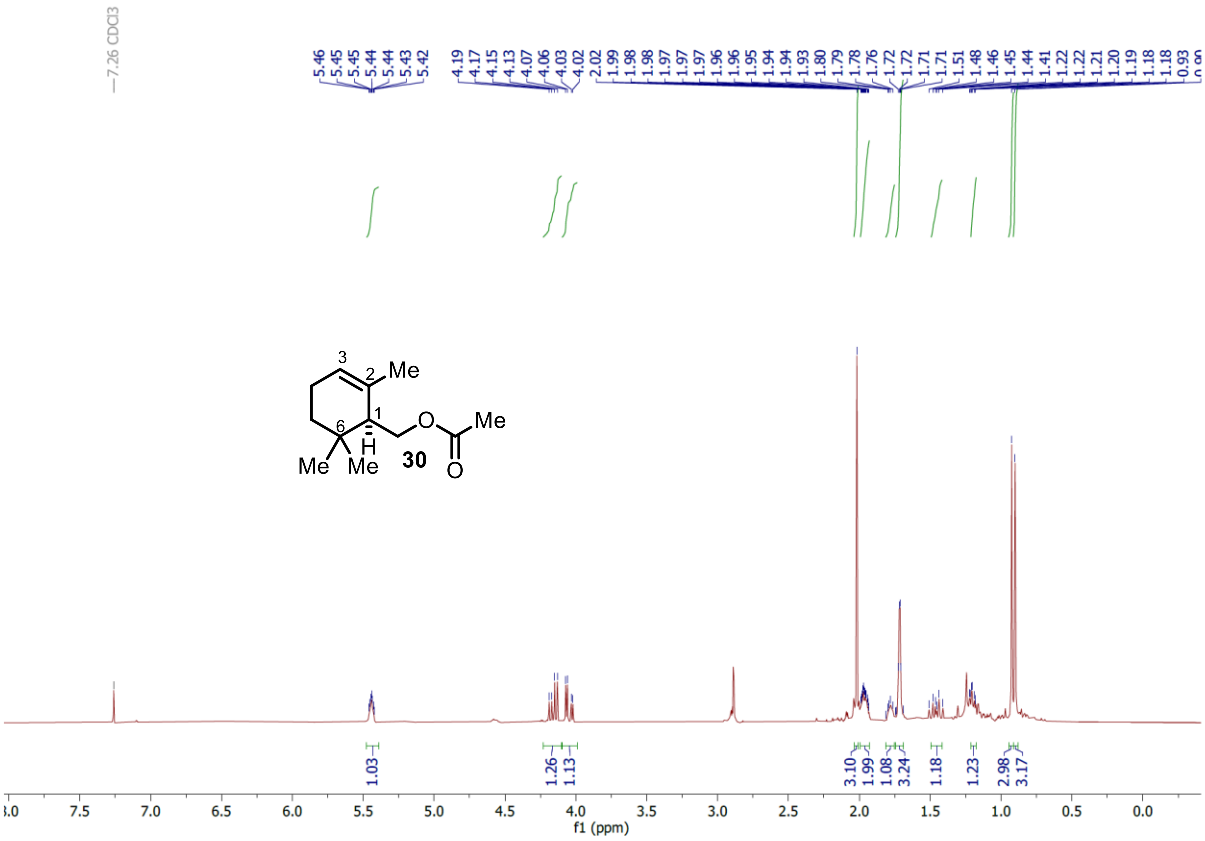
7.26 CDCl3
5.46 5.45 5.45 5.44 5.44 5.43 5.42
4.19 4.17 4.15 4.13 4.07 4.06 4.03 4.02
2.02 1.99 1.98 1.98 1.97 1.97 1.96 1.96 1.95 1.94 1.94 1.93 1.80 1.79 1.78 1.76 1.72 1.72 1.71 1.71 1.51 1.48 1.46 1.45 1.44 1.41 1.22 1.22 1.21 1.20 1.19 1.18 1.18 0.93
3
2
Me
1
O
Me
6
H
Me
Me
30
O
1.03
1.26
1.13
3.10
1.99
1.08
3.24
1.18
1.23
2.98
3.17
f1 (ppm)

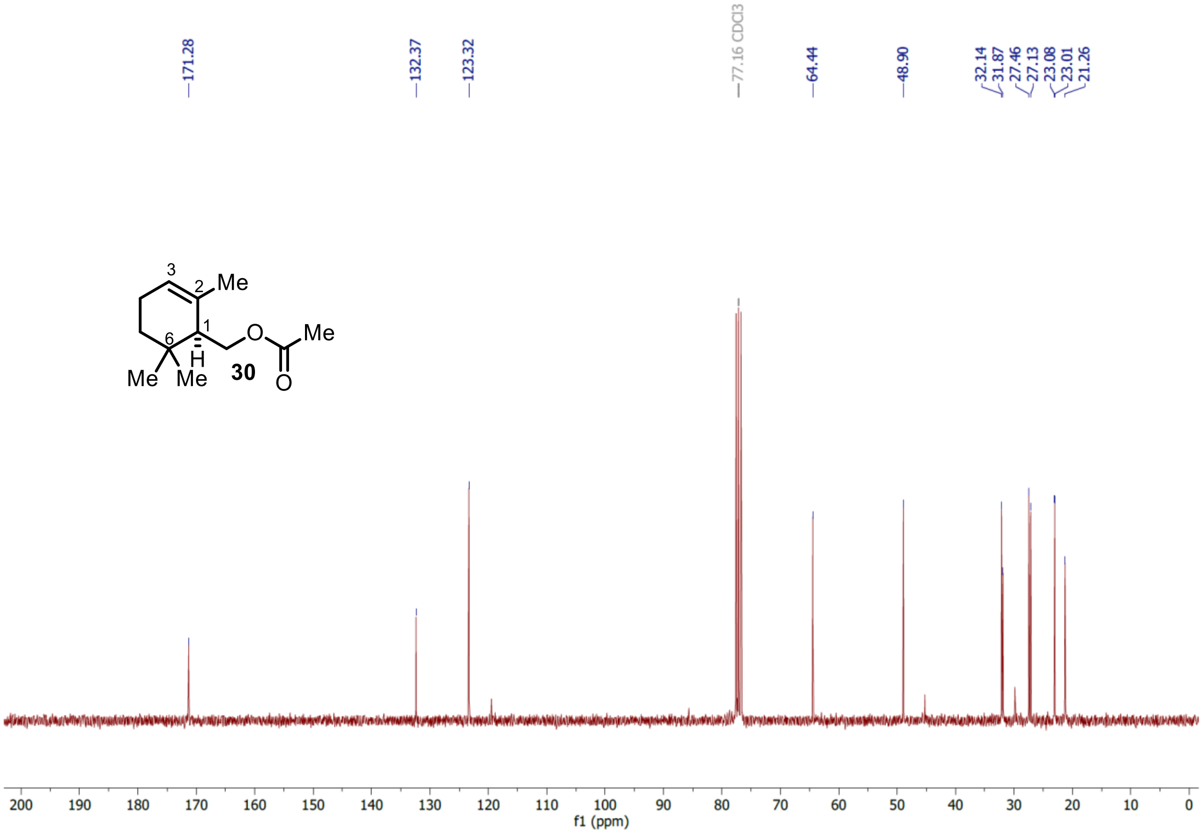
171.28
132.37
123.32
77.16 CDCl3
64.44
48.90
32.14 31.87 27.46 27.13 23.08 23.01 21.26
3
2
Me
1
O
Me
6
H
Me
Me
30
O
f1 (ppm)

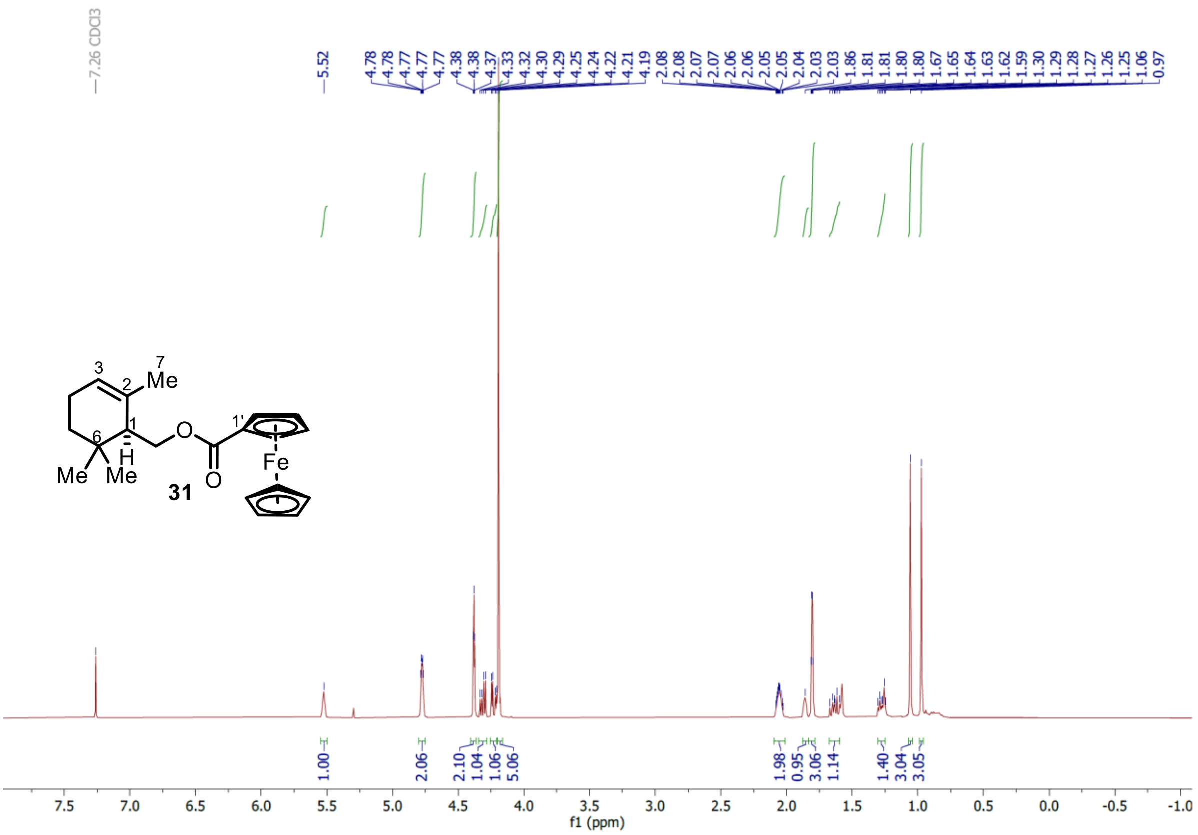
7.26 CDCl3
5.52
4.78 4.78 4.77 4.77 4.38 4.38 4.37 4.33 4.32 4.30 4.29 4.25 4.24 4.22 4.21 4.19
2.08 2.07 2.07 2.06 2.06 2.05 2.05 2.04 2.03 2.03 1.86 1.81 1.81 1.80 1.80 1.67 1.65 1.64 1.63 1.62 1.59 1.30 1.29 1.28 1.27 1.26 1.25 1.06 0.97
Me
Fe
Me Me
O
31
1.00 2.06 2.10 1.04 1.06 5.06 1.98 0.95 3.06 1.14 1.40 3.04 3.05
f1 (ppm)

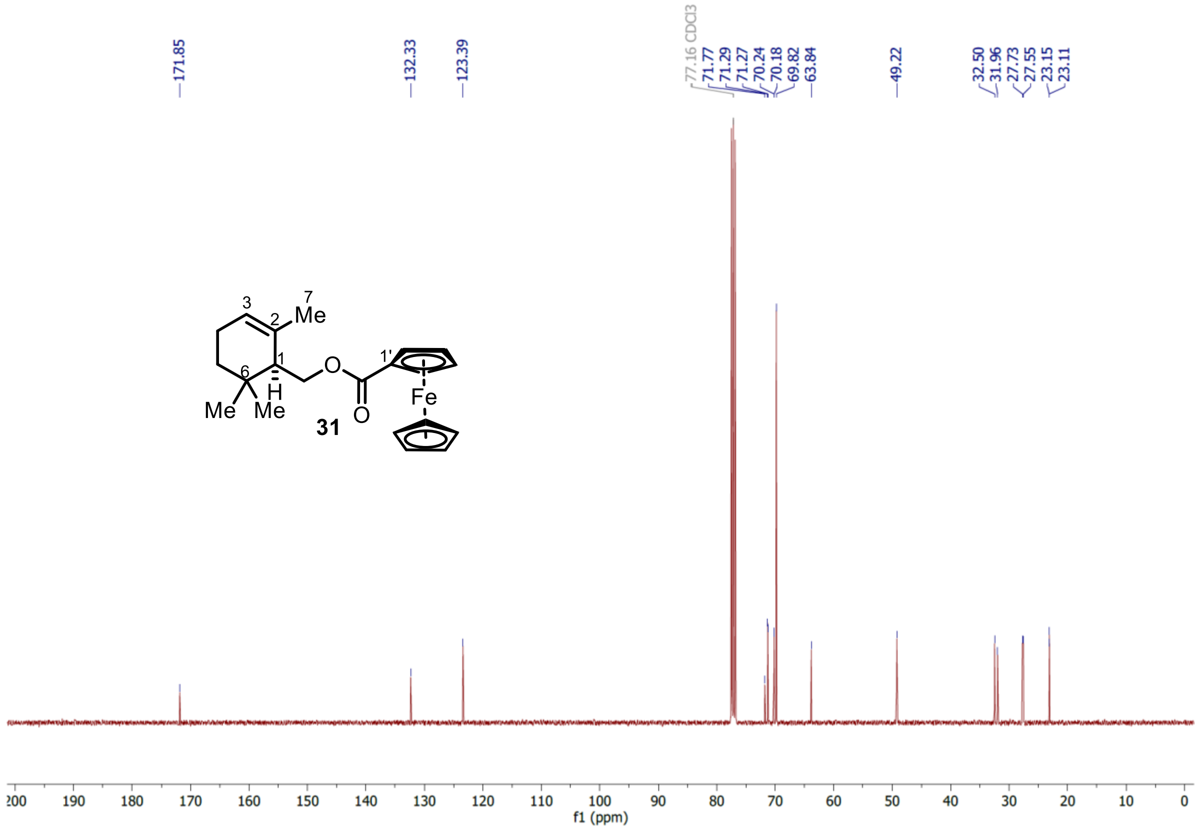
171.85
132.33
123.39
77.16 CDCl3
71.77 71.29 71.27 70.24 70.18 69.82 63.84
49.22
32.50 31.96 27.73 27.55 23.15 23.11
Me
Fe
Me Me
O
31
f1 (ppm)

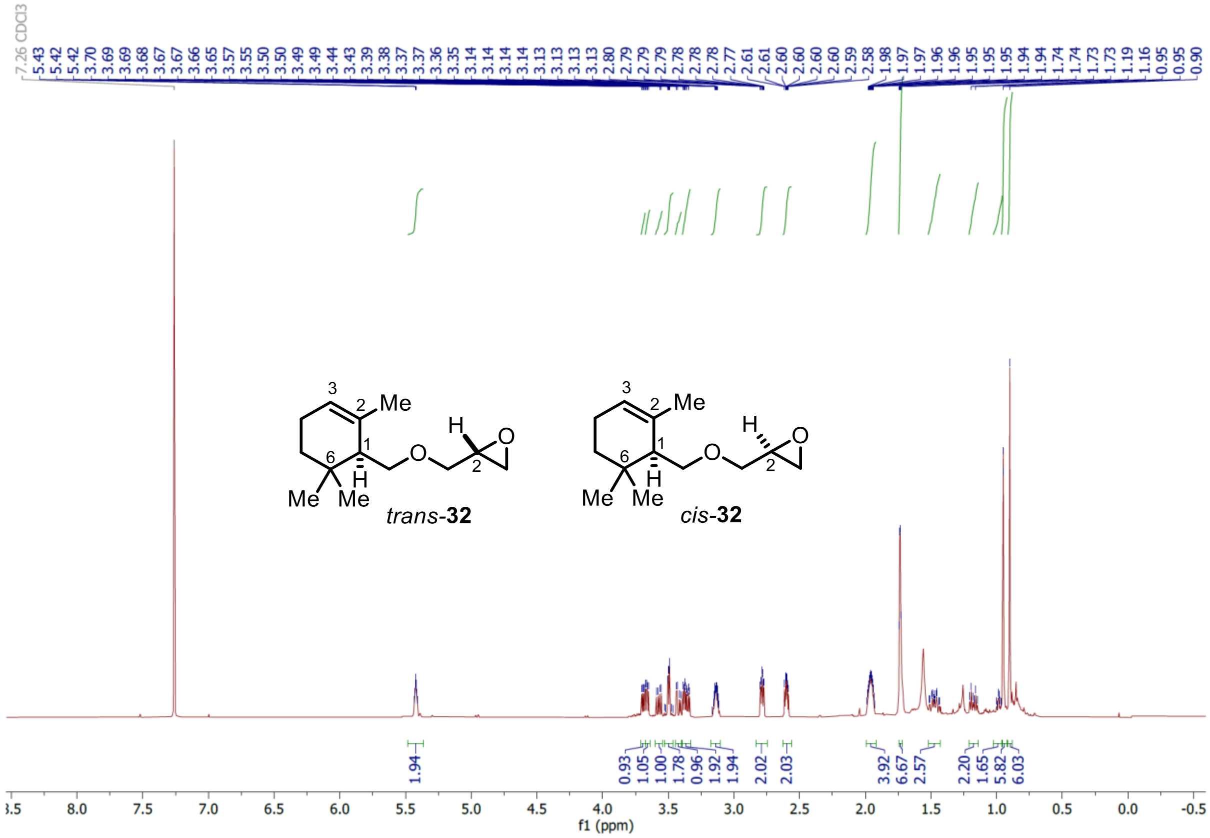
7.26 CDCl3
5.43 5.42 5.42 3.70 3.69 3.69 3.68 3.67 3.67 3.66 3.65 3.57 3.55 3.50 3.50 3.49 3.49 3.44 3.43 3.39 3.38 3.37 3.37 3.36 3.35 3.14 3.14 3.14 3.14 3.13 3.13 3.13 3.13 2.80 2.79 2.79 2.79 2.78 2.78 2.78 2.77 2.61 2.61 2.60 2.60 2.60 2.60 2.59 2.58 1.98 1.97 1.97 1.96 1.96 1.95 1.95 1.95 1.94 1.94 1.74 1.74 1.73 1.73 1.19 1.16 0.95 0.95 0.90
trans-32
cis-32
1.94
0.93 1.05 1.00 1.78 0.96 1.92 1.94 2.02 2.03 3.92 6.67 2.57 2.20 1.65 5.82 6.03
f1 (ppm)

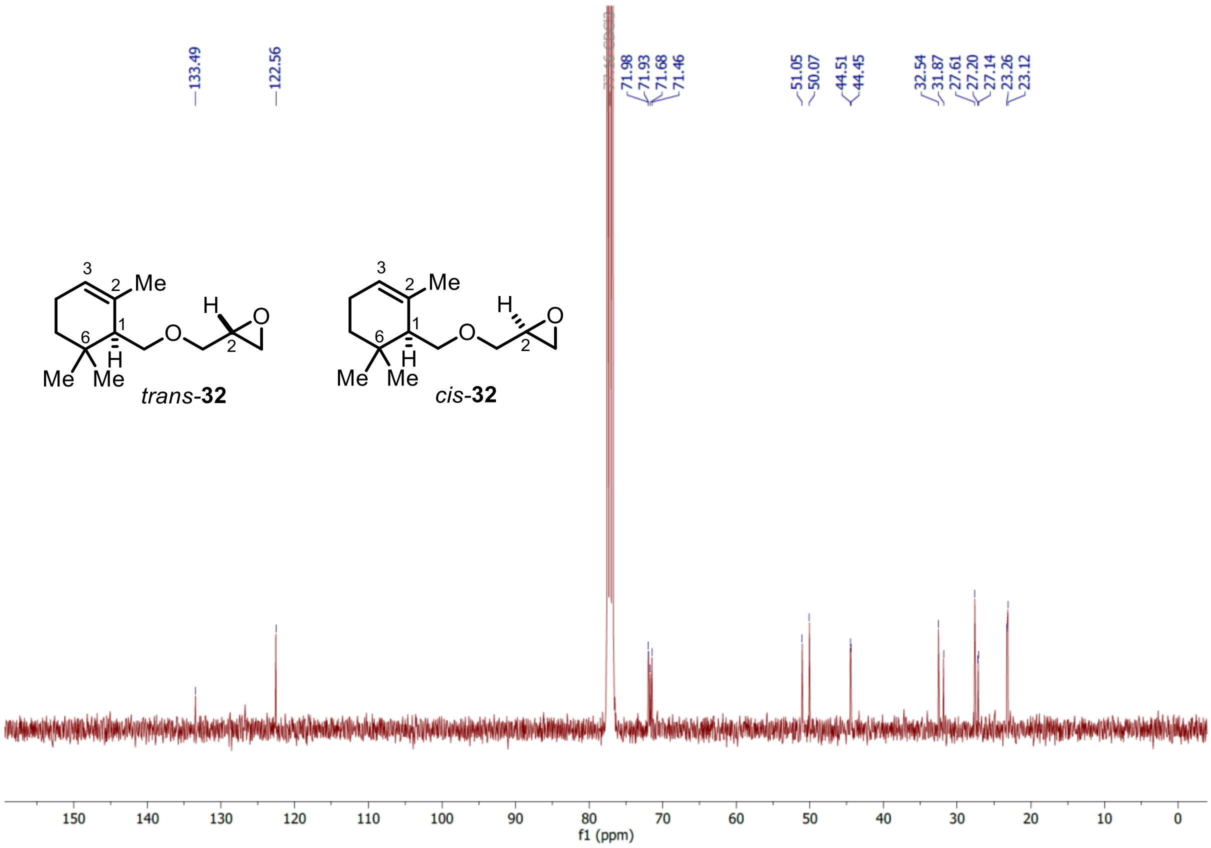
133.49
122.56
77.16 CDCl3
71.98 71.93 71.68 71.46
51.05 50.07
44.51 44.45
32.54 31.87 27.61 27.20 27.14 23.26 23.12
trans-32
cis-32
f1 (ppm)

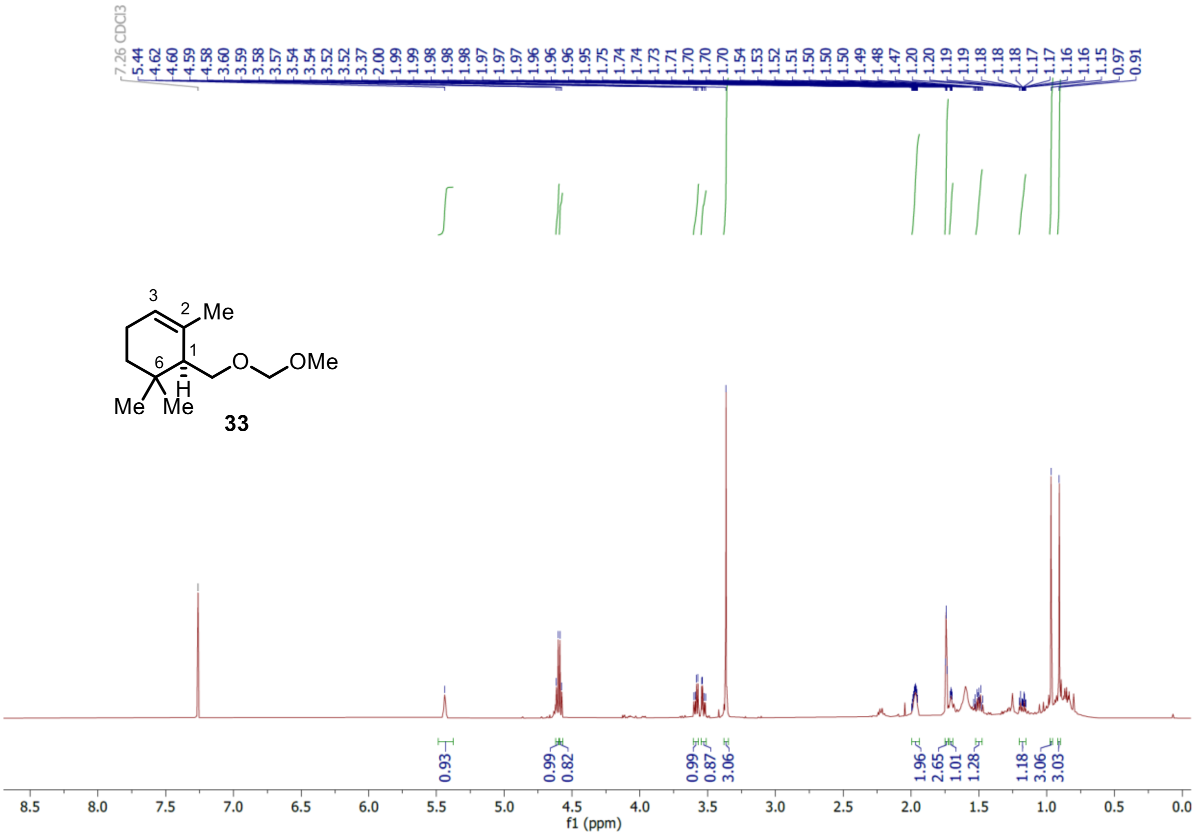

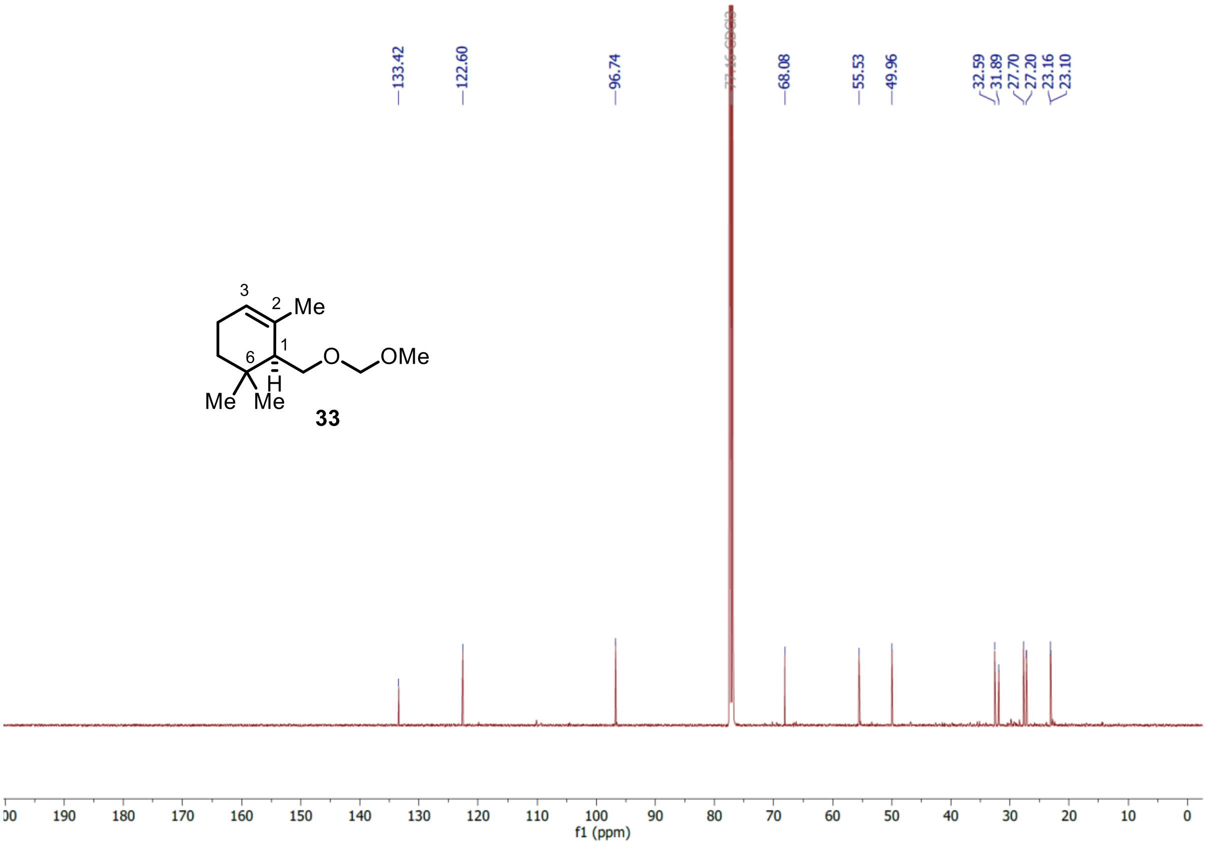

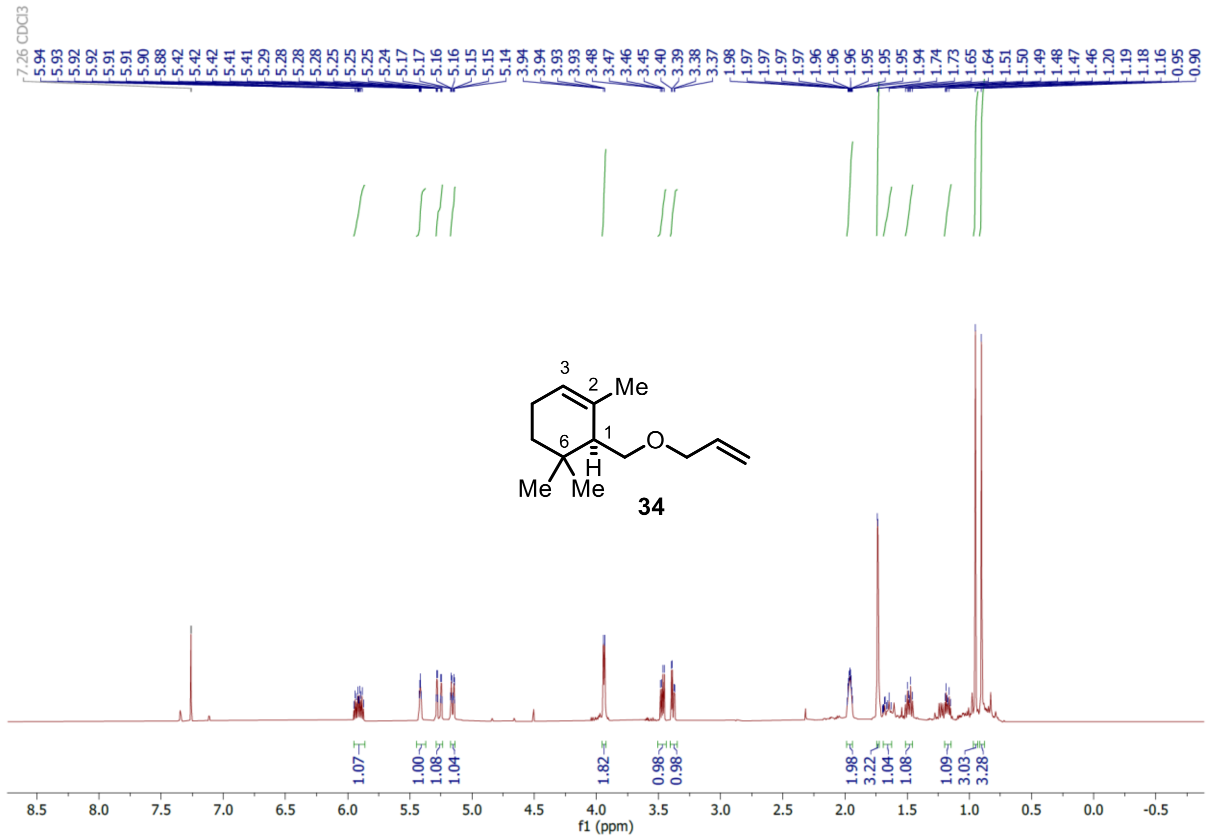
7.26 CDCl3
5.94 5.93 5.92 5.92 5.91 5.91 5.90 5.88 5.42 5.42 5.41 5.41 5.29 5.28 5.28 5.28 5.25 5.25 5.25 5.24 5.17 5.17 5.16 5.16 5.15 5.14 3.94 3.94 3.93 3.93 3.48 3.47 3.46 3.45 3.40 3.39 3.38 3.37 1.98 1.97 1.97 1.97 1.97 1.96 1.96 1.96 1.95 1.95 1.95 1.94 1.74 1.73 1.65 1.64 1.51 1.50 1.49 1.48 1.47 1.46 1.20 1.19 1.18 1.16 0.95 0.90
3
2
Me
1
6
O
H
Me
Me
34
1.07
1.00
1.08
1.04
1.82
0.98
0.98
1.98
3.22
1.04
1.08
1.09
3.03
3.28
f1 (ppm)

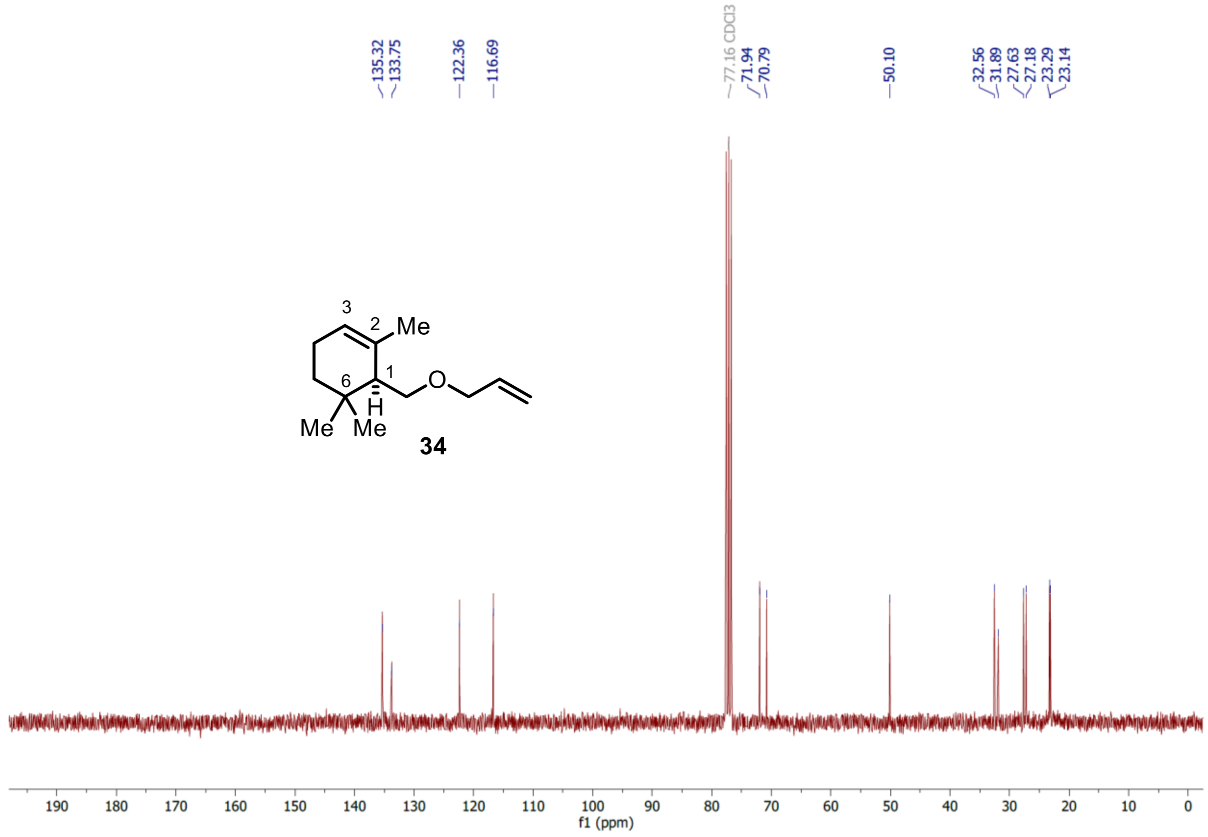
135.32
133.75
122.36
116.69
77.16 CDCl3
71.94
70.79
50.10
32.56
31.89
27.63
27.18
23.29
23.14
3
2
Me
1
6
O
H
Me
Me
34
f1 (ppm)

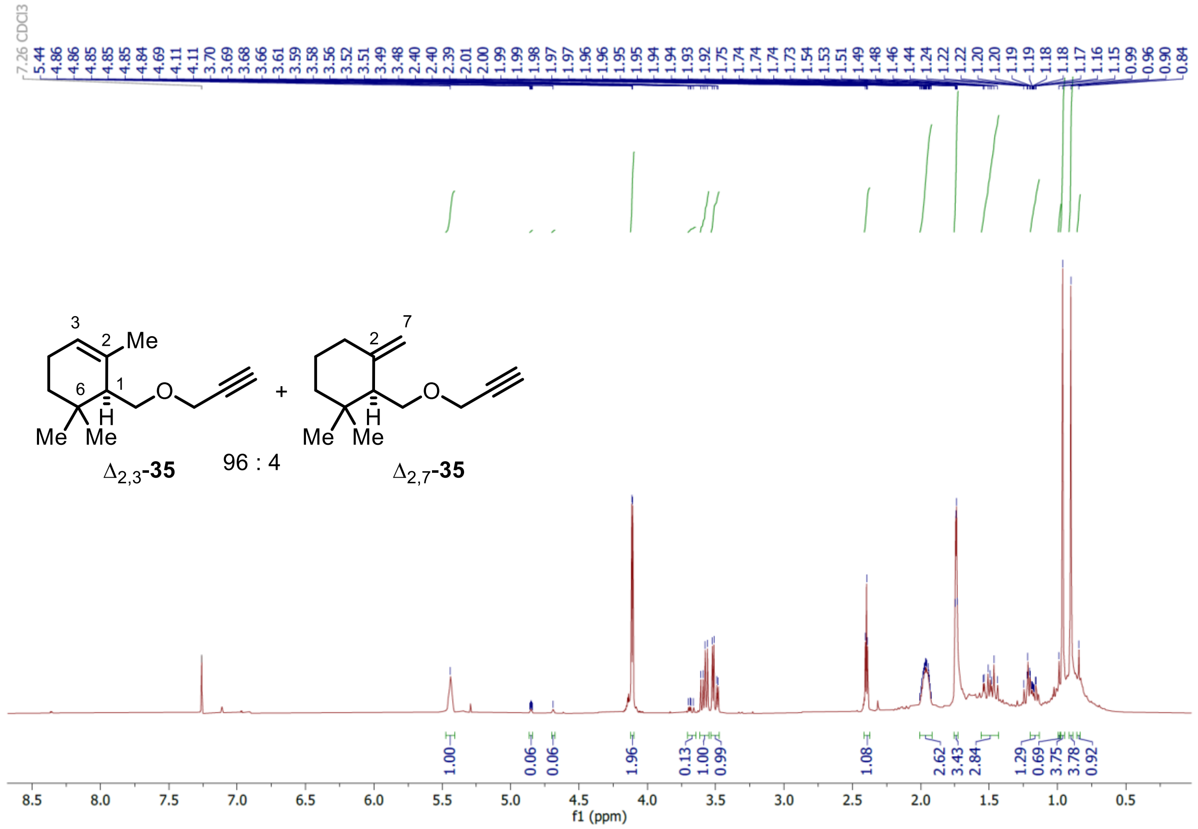
7.26 CDCl3
5.44
4.86
4.86
4.85
4.85
4.85
4.84
4.69
4.11
4.11
3.70
3.69
3.68
3.66
3.61
3.59
3.58
3.56
3.52
3.51
3.49
3.48
2.40
2.40
2.39
2.01
2.00
1.99
1.99
1.98
1.97
1.97
1.96
1.96
1.95
1.95
1.95
1.94
1.94
1.93
1.92
1.75
1.74
1.74
1.74
1.73
1.54
1.53
1.51
1.49
1.48
1.46
1.44
1.24
1.22
1.22
1.20
1.20
1.19
1.19
1.18
1.18
1.17
1.16
1.15
0.99
0.96
0.90
0.84
3
2
Me
6
1
O
H
Me
Me
+
7
2
O
H
Me
Me
Δ2,3-35
96 : 4
Δ2,7-35
1.00
0.06
0.06
1.96
0.13
1.00
0.99
1.08
2.62
3.43
2.84
1.29
0.69
3.75
3.78
0.92
8.5
8.0
7.5
7.0
6.5
6.0
5.5
5.0
4.5
4.0
3.5
3.0
2.5
2.0
1.5
1.0
0.5
f1 (ppm)

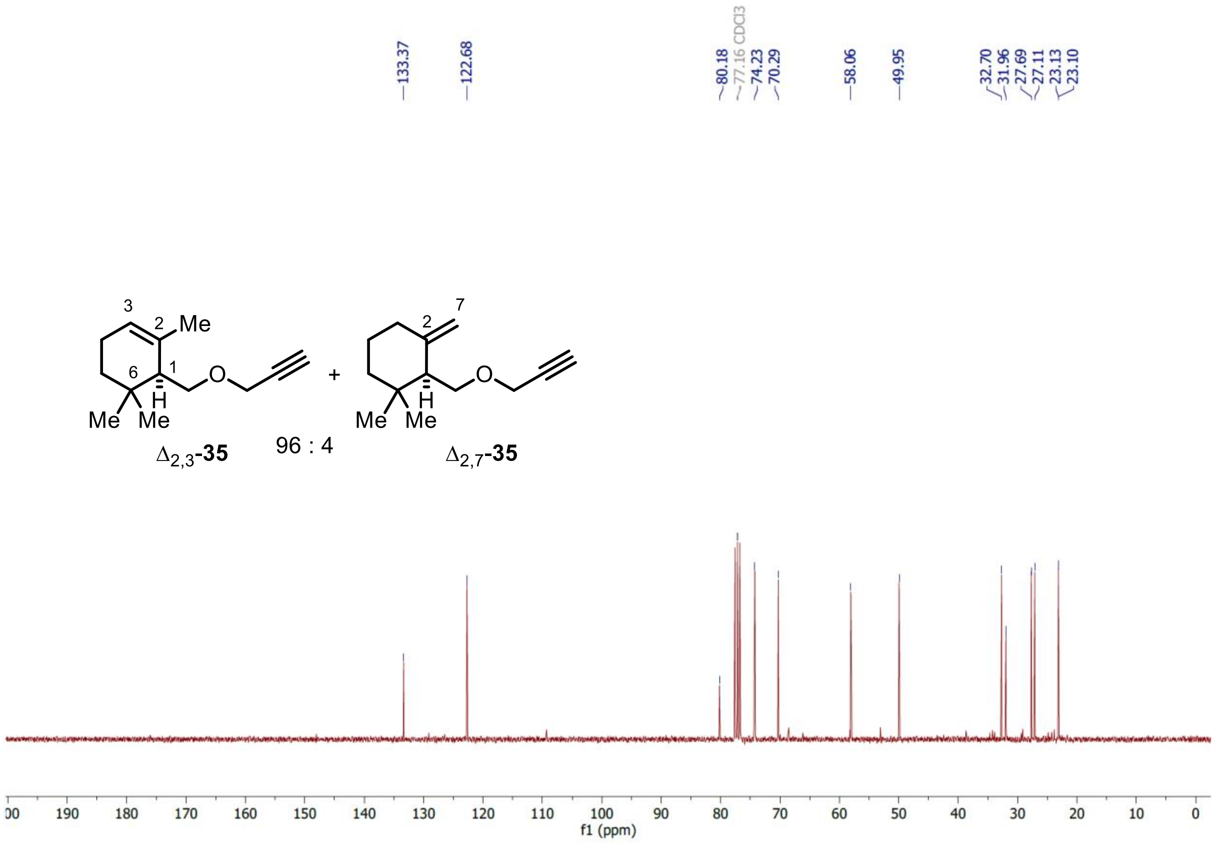
133.37
122.68
80.18
77.16 CDCl3
74.23
70.29
58.06
49.95
32.70
31.96
27.69
27.11
23.13
23.10
3
2
Me
6
1
O
H
Me
Me
+
7
2
O
H
Me
Me
Δ2,3-35
96 : 4
Δ2,7-35
00
190
180
170
160
150
140
130
120
110
100
90
80
70
60
50
40
30
20
10
0
f1 (ppm)

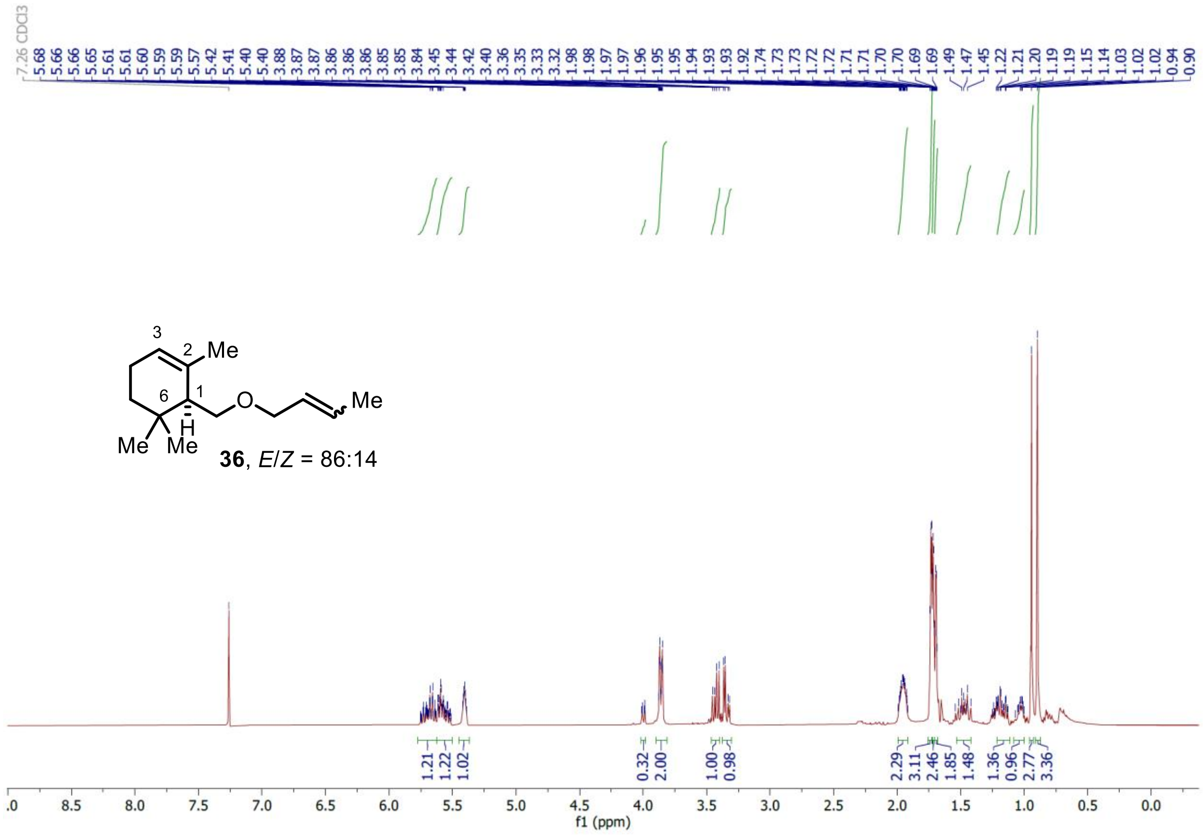

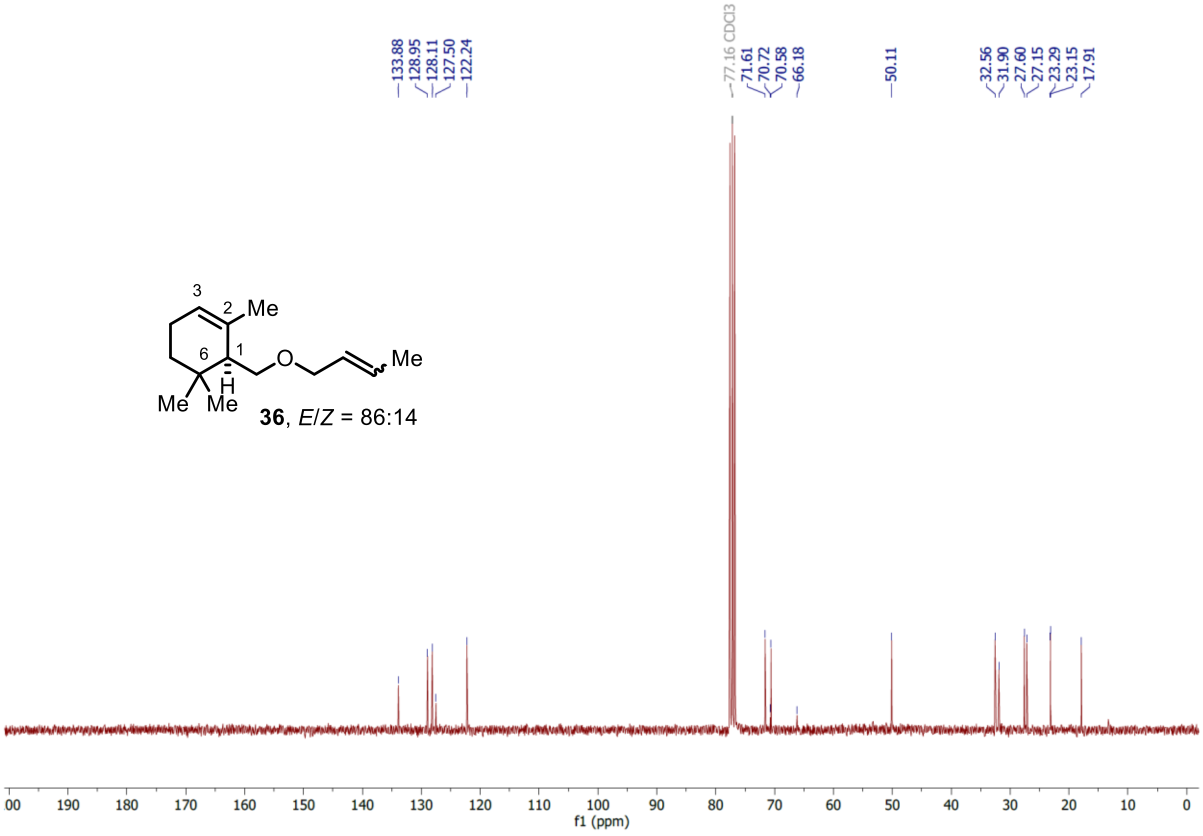

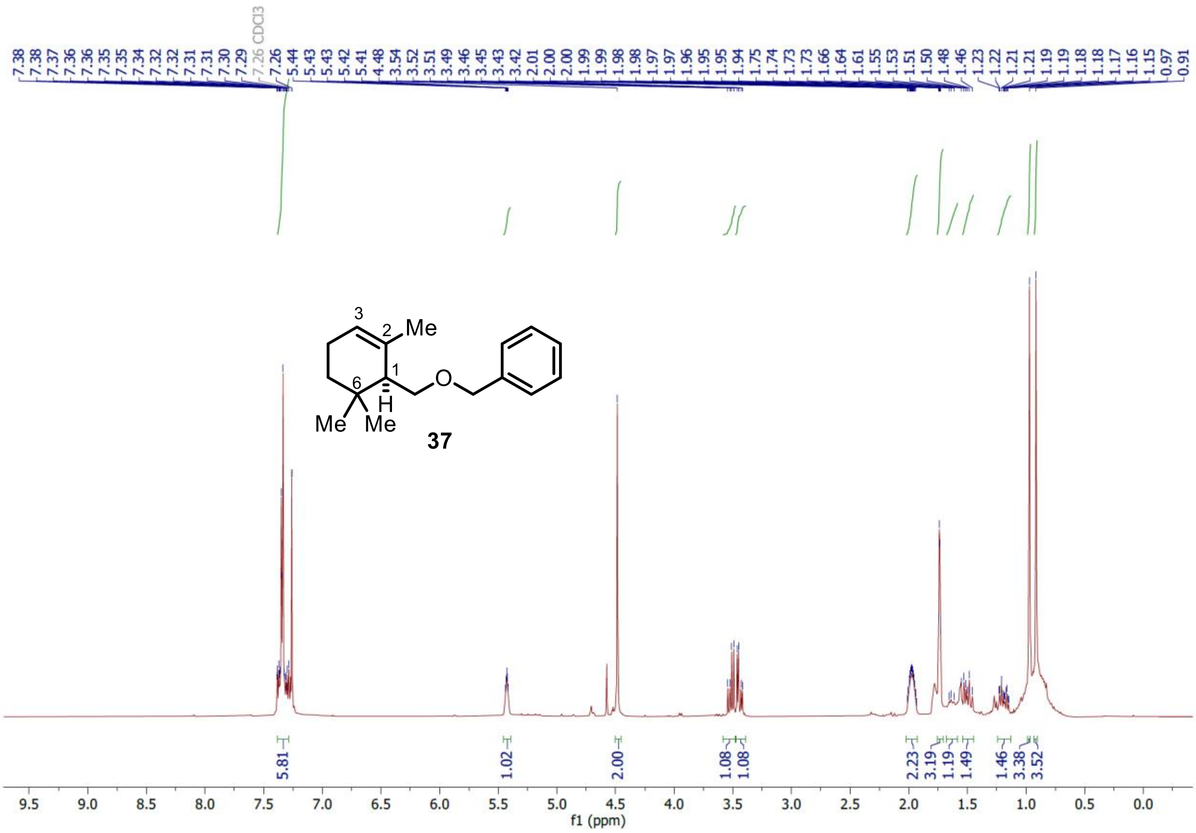
7.38 7.38 7.37 7.36 7.36 7.35 7.35 7.34 7.32 7.32 7.31 7.31 7.30 7.29 7.26 CDCl3 7.26 5.44 5.43 5.43 5.42 5.41 4.48 3.54 3.52 3.51 3.49 3.46 3.45 3.43 3.42 2.01 2.00 2.00 1.99 1.99 1.98 1.98 1.97 1.97 1.96 1.95 1.95 1.94 1.75 1.74 1.73 1.66 1.64 1.61 1.55 1.53 1.51 1.50 1.48 1.46 1.23 1.22 1.21 1.21 1.19 1.19 1.18 1.18 1.17 1.16 1.15 0.97 0.91
3
2
Me
1
O
6
H
Me
Me
37
5.81
1.02
2.00
1.08
1.08
2.23
3.19
1.19
1.49
1.46
3.38
3.52
9.5 9.0 8.5 8.0 7.5 7.0 6.5 6.0 5.5 5.0 4.5 4.0 3.5 3.0 2.5 2.0 1.5 1.0 0.5 0.0
f1 (ppm)

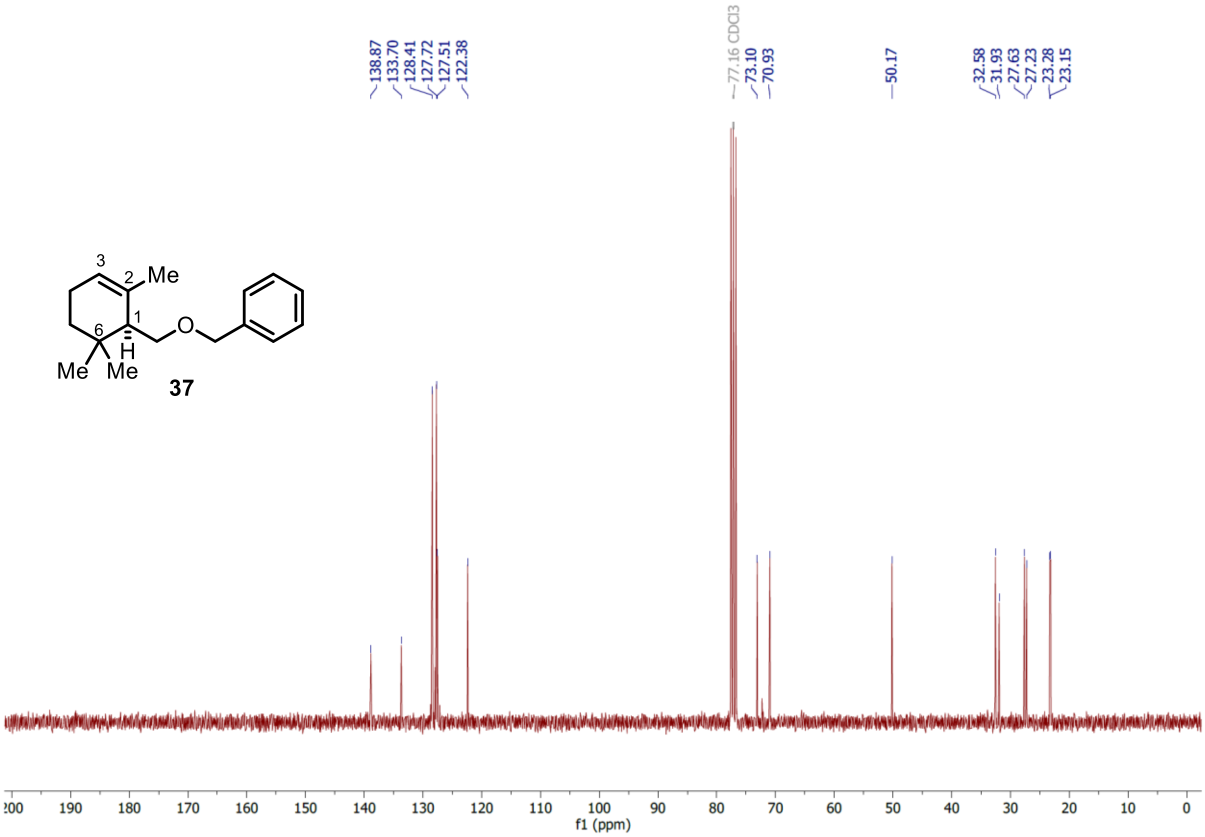
138.87 133.70 128.41 127.72 127.51 122.38
77.16 CDCl3 73.10 70.93
50.17
32.58 31.93 27.63 27.23 23.28 23.15
3
2
Me
1
O
6
H
Me
Me
37
200 190 180 170 160 150 140 130 120 110 100 90 80 70 60 50 40 30 20 10 0
f1 (ppm)

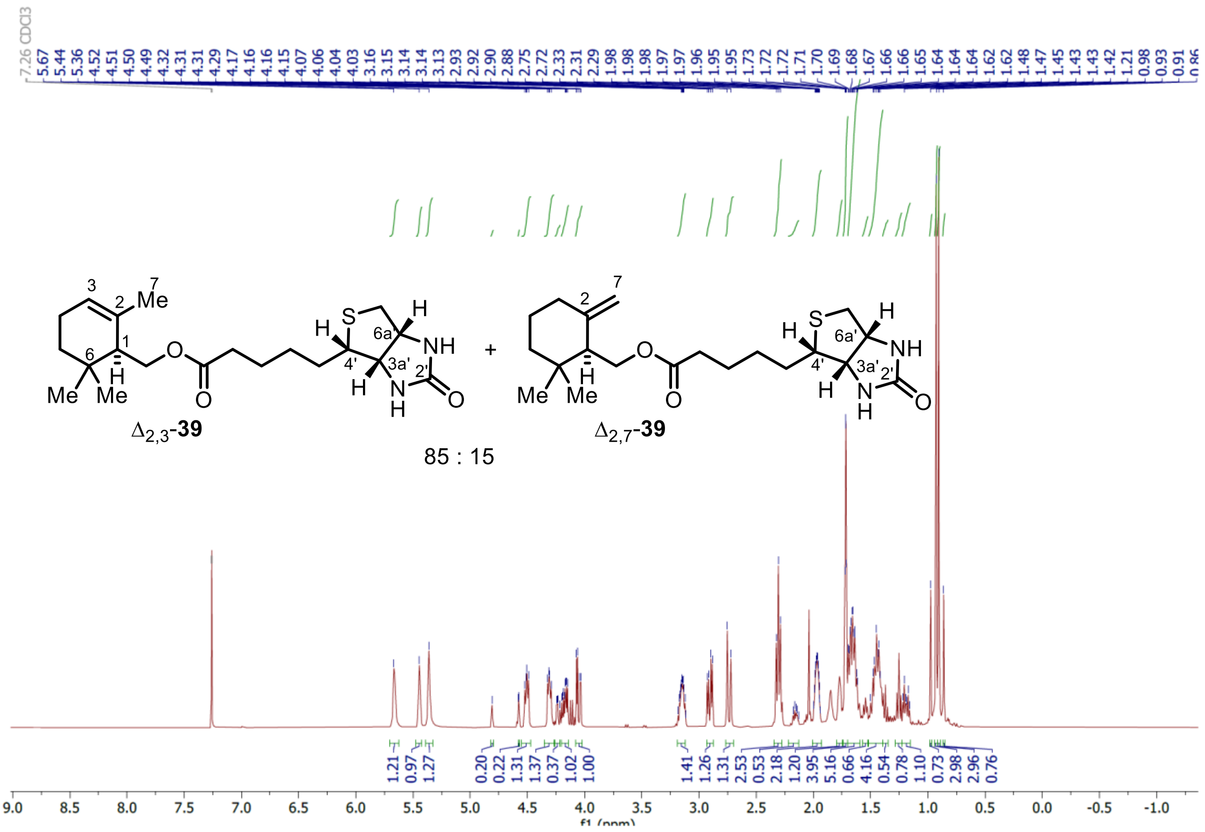
Δ2,3-39
Δ2,7-39
85 : 15
f1 (ppm)

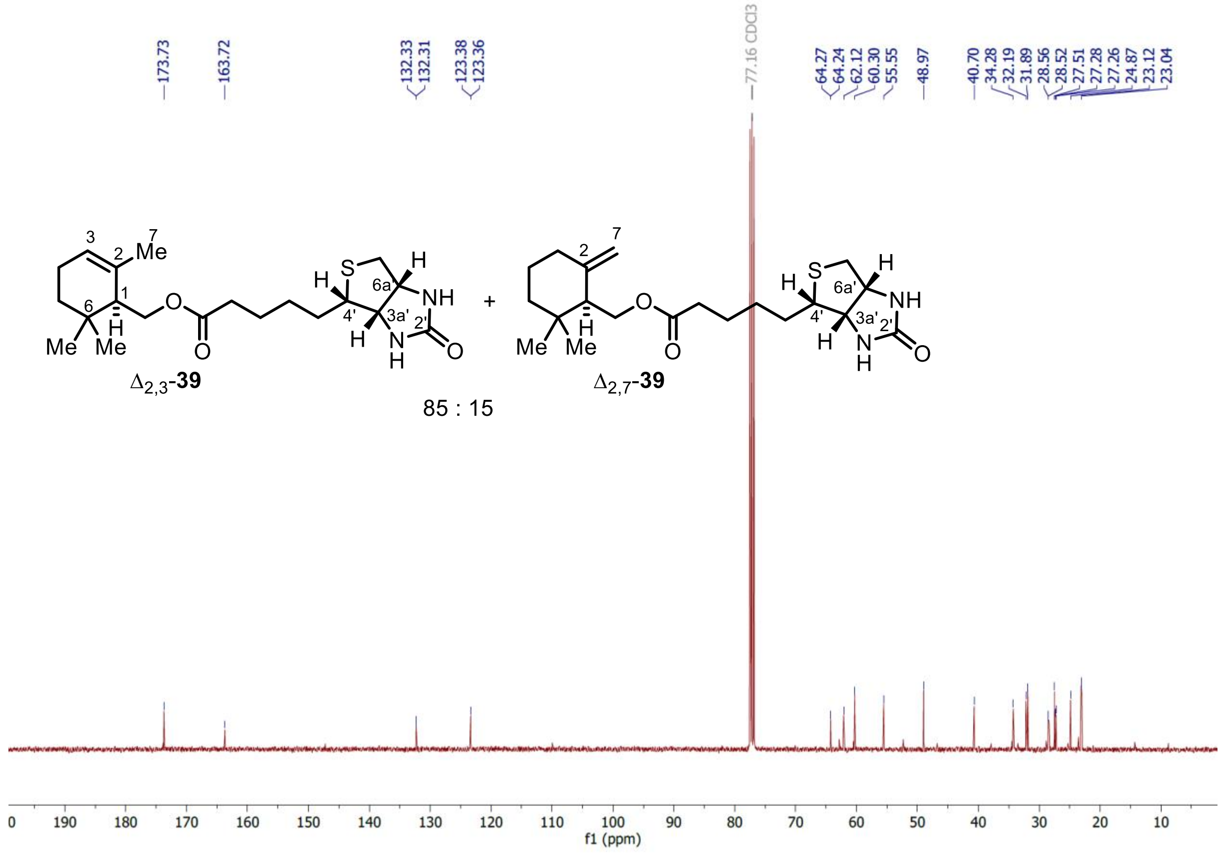
Δ2,3-39
Δ2,7-39
85 : 15
f1 (ppm)

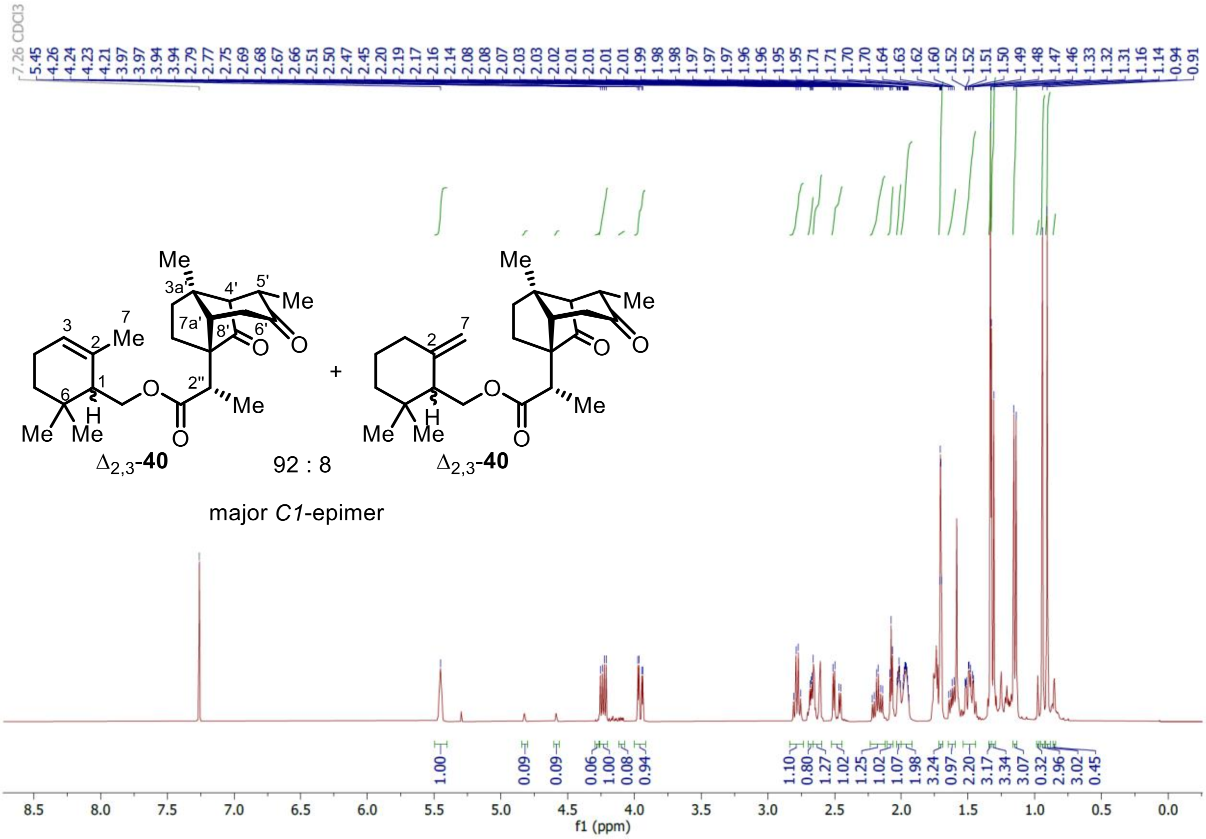
7.26 CDCl3
Δ2,3-40
92 : 8
Δ2,3-40
major C1-epimer
f1 (ppm)

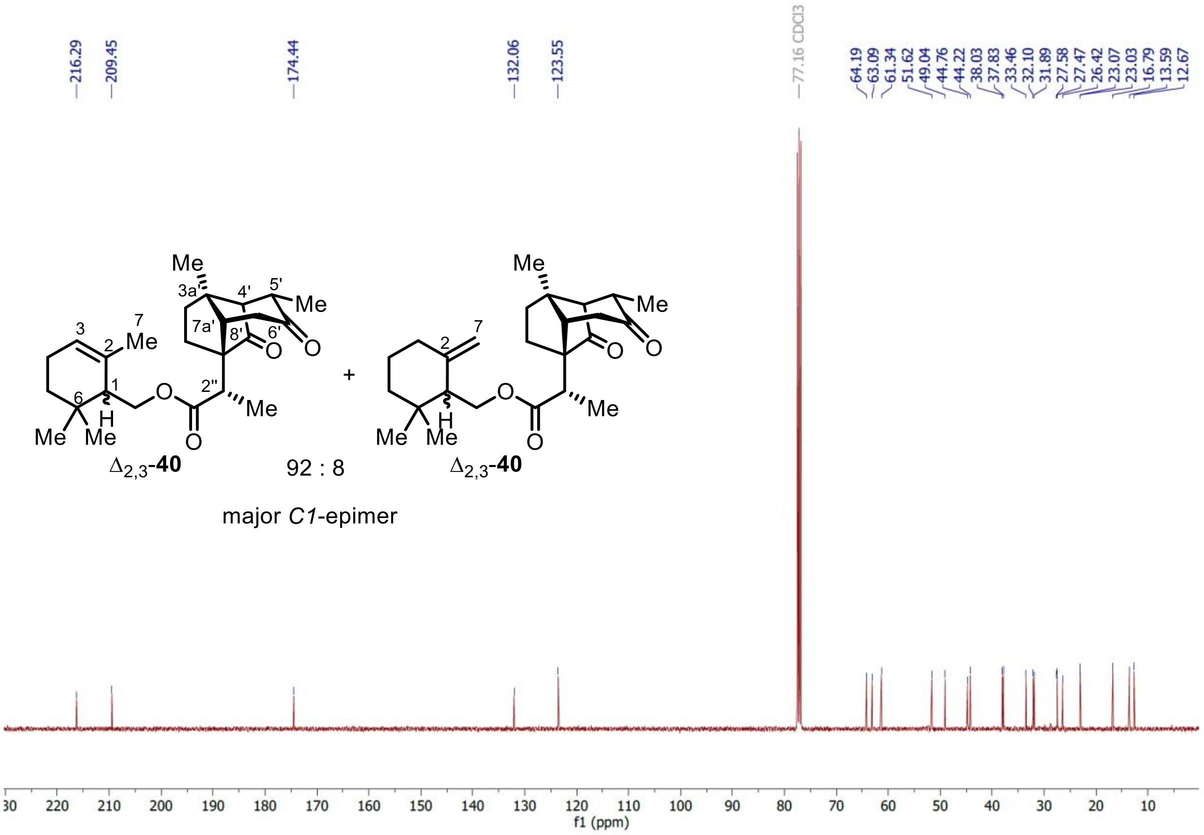
216.29
209.45
174.44
132.06
123.55
77.16 CDCl3
64.19
63.09
61.34
51.62
49.04
44.76
44.22
38.03
37.83
33.46
32.10
31.89
27.58
27.47
26.42
23.07
23.03
16.79
13.59
12.67
Δ2,3-40
92 : 8
Δ2,3-40
major C1-epimer
f1 (ppm)

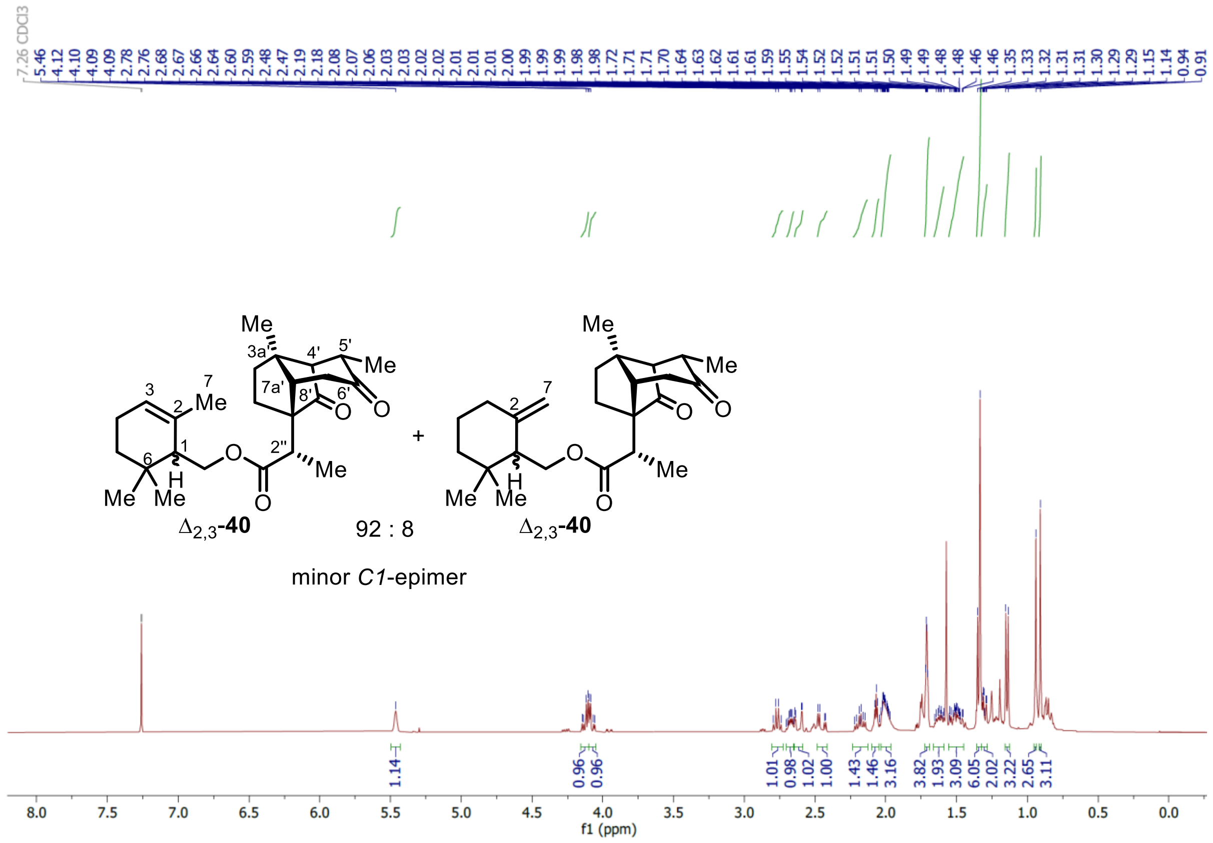
7.26 CDCl3
5.46 4.12 4.10 4.09 4.09 2.78 2.76 2.68 2.67 2.66 2.64 2.60 2.59 2.48 2.47 2.19 2.18 2.08 2.07 2.06 2.03 2.03 2.02 2.02 2.01 2.01 2.01 2.00 1.99 1.99 1.99 1.98 1.98 1.72 1.71 1.71 1.70 1.64 1.63 1.62 1.61 1.61 1.59 1.55 1.54 1.52 1.52 1.51 1.51 1.50 1.49 1.49 1.48 1.48 1.46 1.46 1.35 1.33 1.32 1.31 1.31 1.30 1.29 1.29 1.15 1.14 0.94 0.91
$\Delta_{2,3}$-40
92 : 8
$\Delta_{2,3}$-40
minor *C1*-epimer
1.14 0.96 0.96 1.01 0.98 1.02 1.00 1.43 1.46 3.16 3.82 1.93 3.09 6.05 2.02 3.22 2.65 3.11
f1 (ppm)

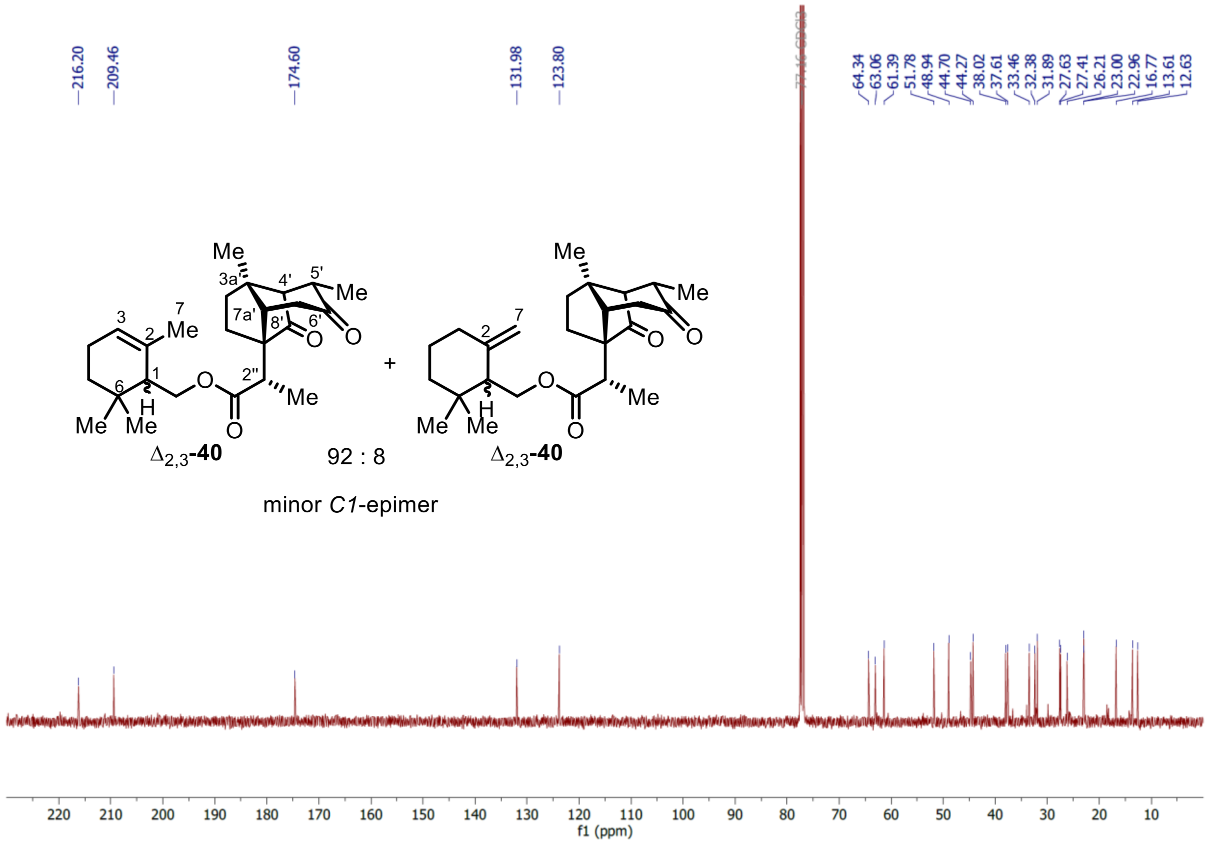
216.20 209.46 174.60 131.98 123.80
77.16 CDCl3
64.34 63.06 61.39 51.78 48.94 44.70 44.27 38.02 37.61 33.46 32.38 31.89 27.63 27.41 26.21 23.00 22.96 16.77 13.61 12.63
$\Delta_{2,3}$-40
92 : 8
$\Delta_{2,3}$-40
minor *C1*-epimer
f1 (ppm)

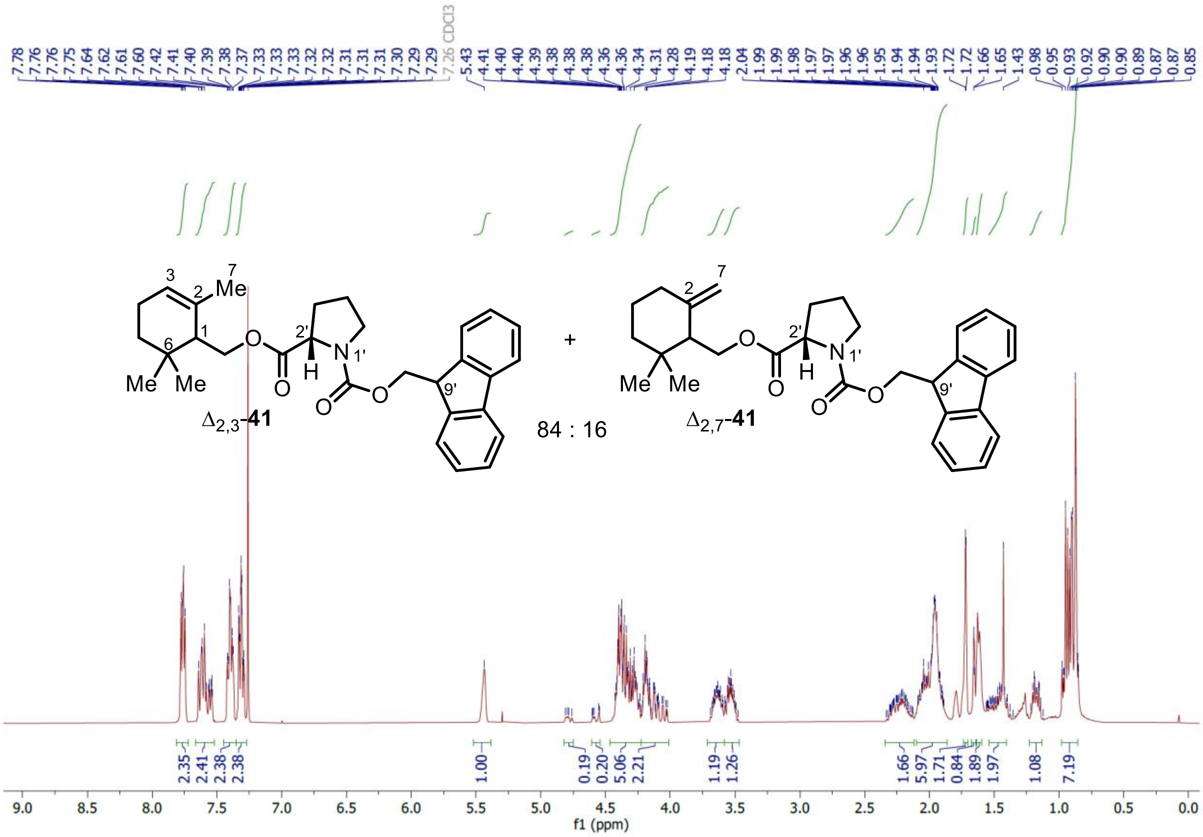

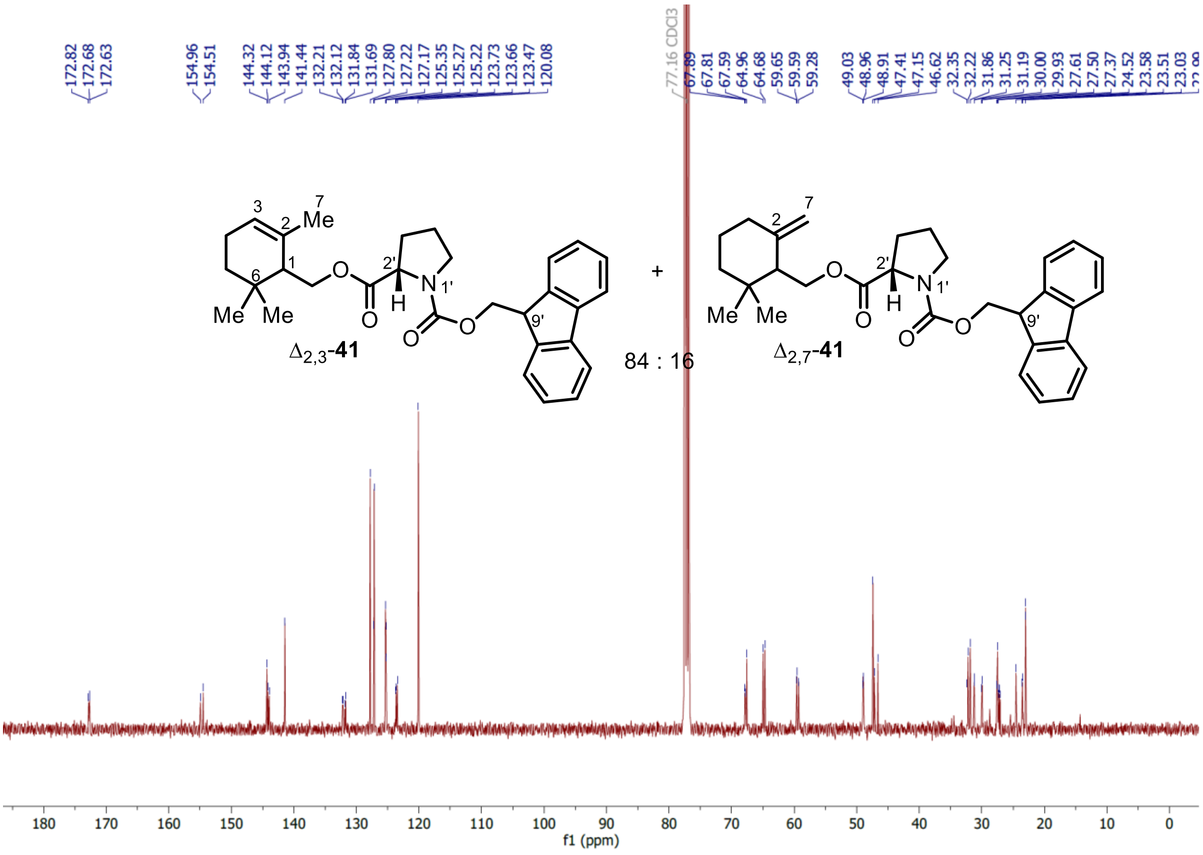

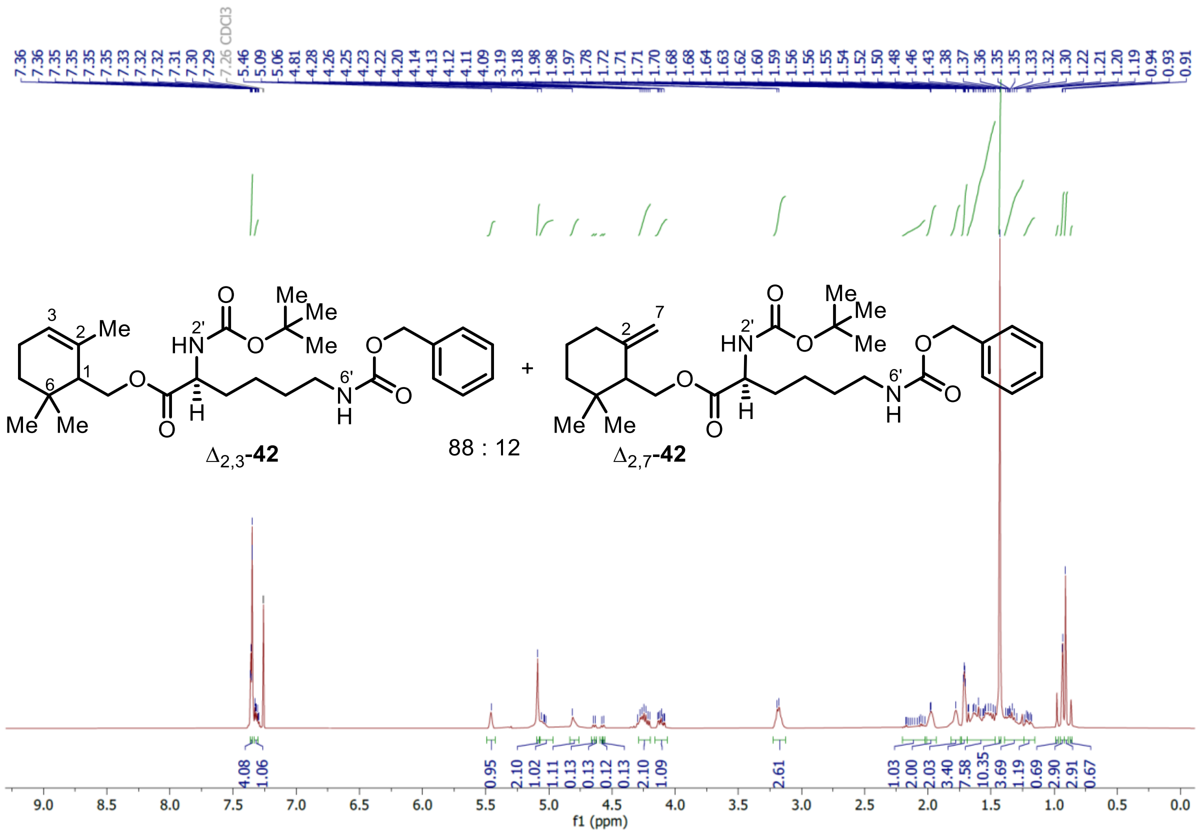
7.36 7.36 7.35 7.35 7.35 7.33 7.32 7.32 7.31 7.30 7.29 7.26 CDCl3 5.46 5.09 5.06 4.81 4.28 4.26 4.25 4.23 4.22 4.20 4.14 4.13 4.12 4.11 4.09 3.19 3.18 1.98 1.98 1.97 1.78 1.72 1.71 1.71 1.70 1.68 1.68 1.64 1.63 1.62 1.60 1.59 1.56 1.56 1.55 1.54 1.52 1.50 1.48 1.46 1.43 1.38 1.37 1.36 1.35 1.35 1.33 1.32 1.30 1.22 1.21 1.20 1.19 0.94 0.93 0.91
Δ2,3-42
88 : 12
Δ2,7-42
4.08 1.06 0.95 2.10 1.02 1.11 0.13 0.13 0.12 0.13 2.10 1.09 2.61 1.03 2.00 2.03 3.40 7.58 10.35 3.69 1.19 0.69 2.90 2.91 0.67
f1 (ppm)

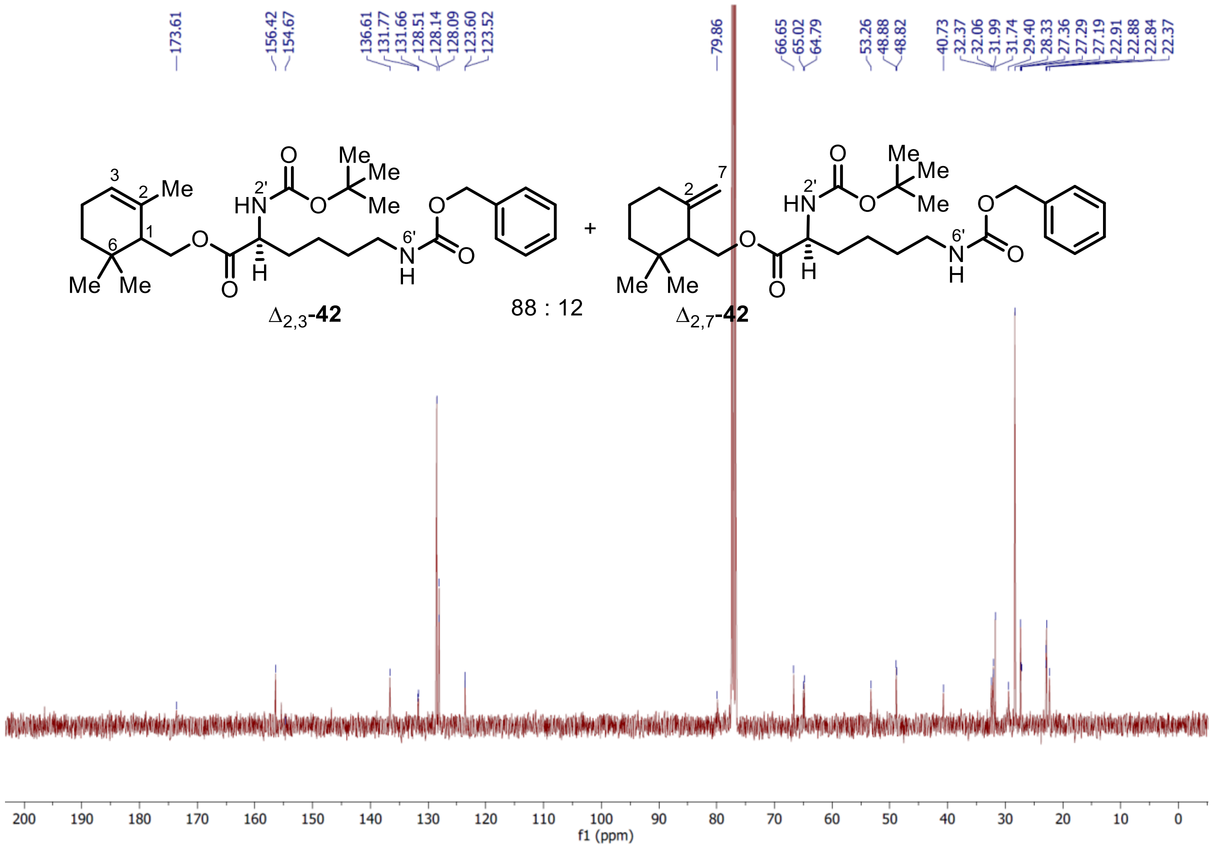
173.61 156.42 154.67 136.61 131.77 131.66 128.51 128.14 128.09 123.60 123.52 79.86 66.65 65.02 64.79 53.26 48.88 48.82 40.73 32.37 32.06 31.99 31.74 29.40 28.33 27.36 27.29 27.19 22.91 22.88 22.84 22.37
Δ2,3-42
88 : 12
Δ2,7-42
f1 (ppm)

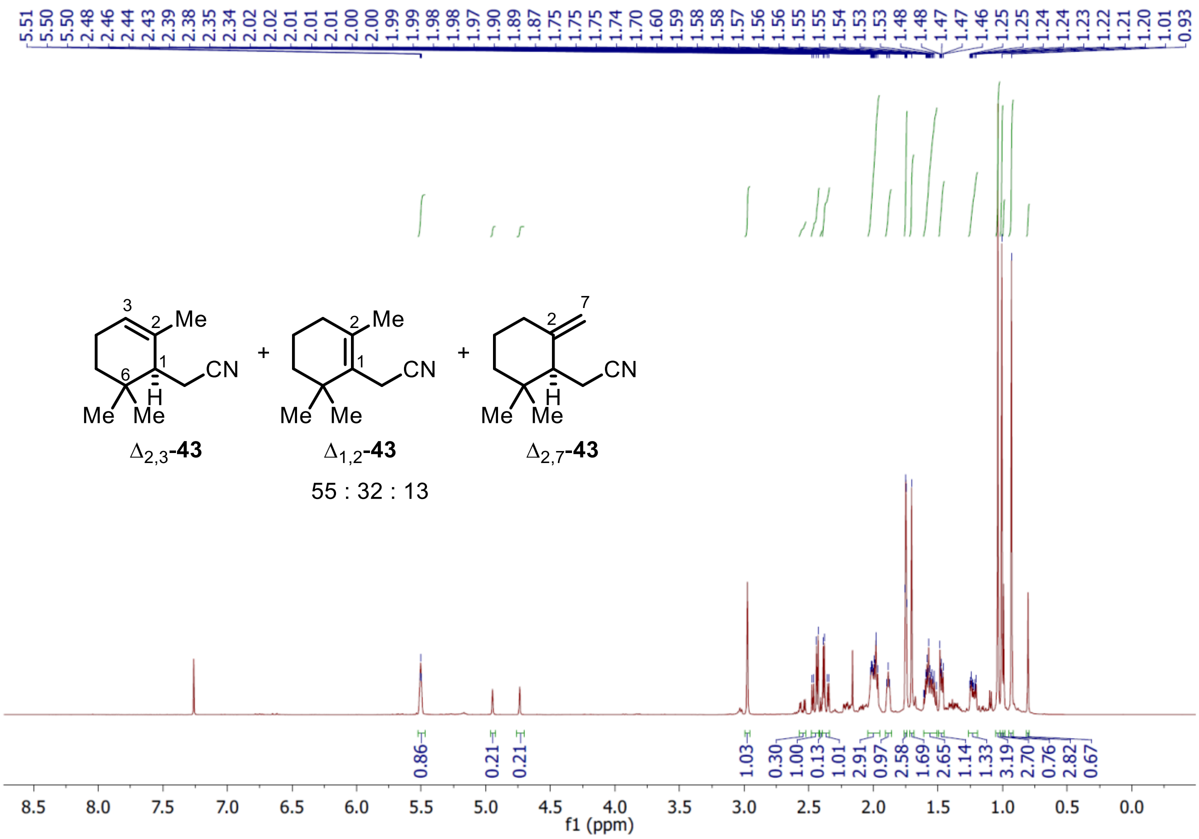

Δ2,3-43
Δ1,2-43
Δ2,7-43
55 : 32 : 13
f1 (ppm)

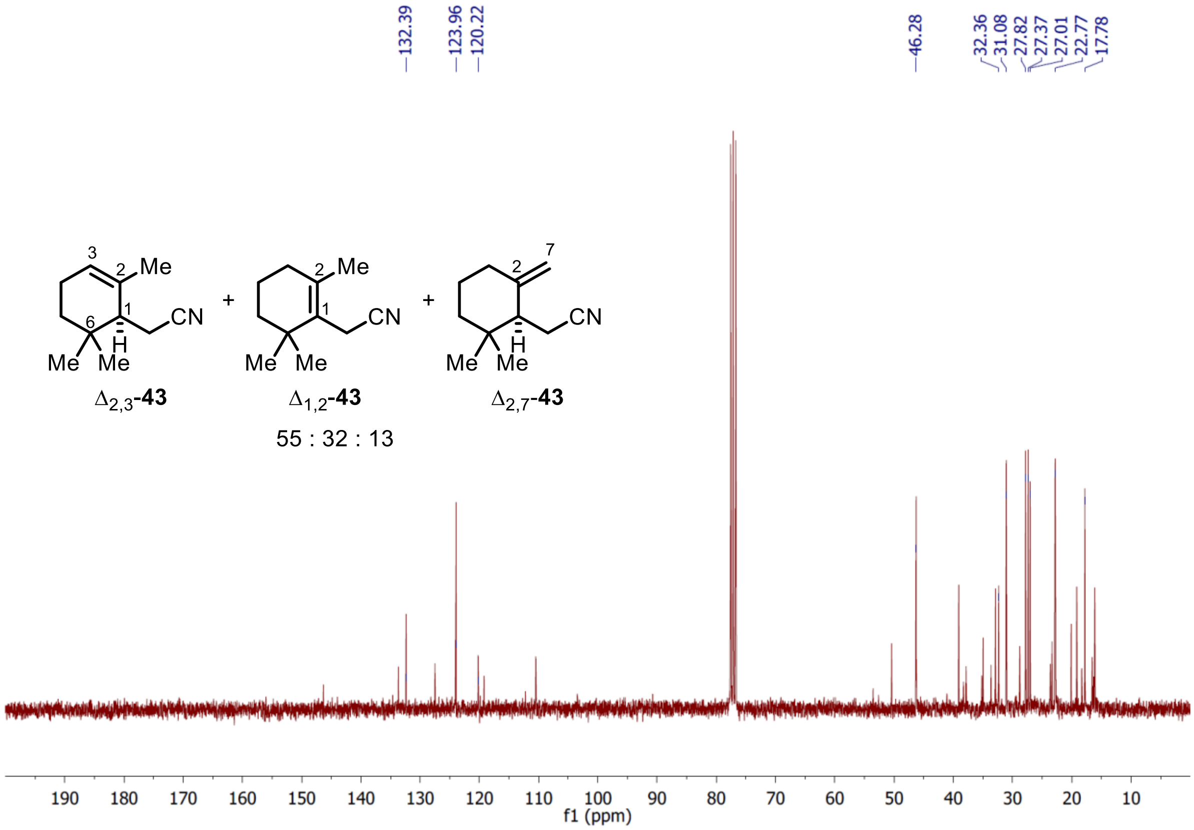

132.39
123.96
120.22
46.28
32.36
31.08
27.82
27.37
27.01
22.77
17.78
Δ2,3-43
Δ1,2-43
Δ2,7-43
55 : 32 : 13
f1 (ppm)

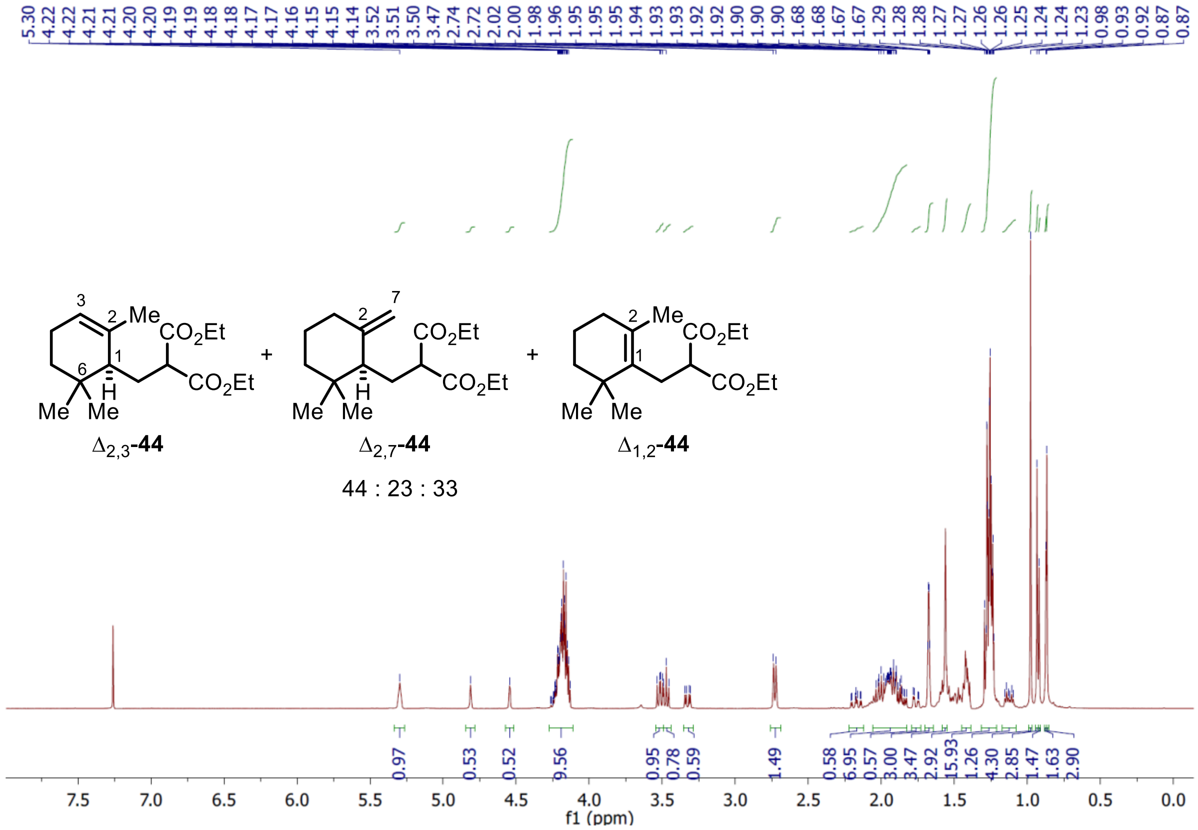
Δ2,3-44
Δ2,7-44
Δ1,2-44
44 : 23 : 33
f1 (ppm)

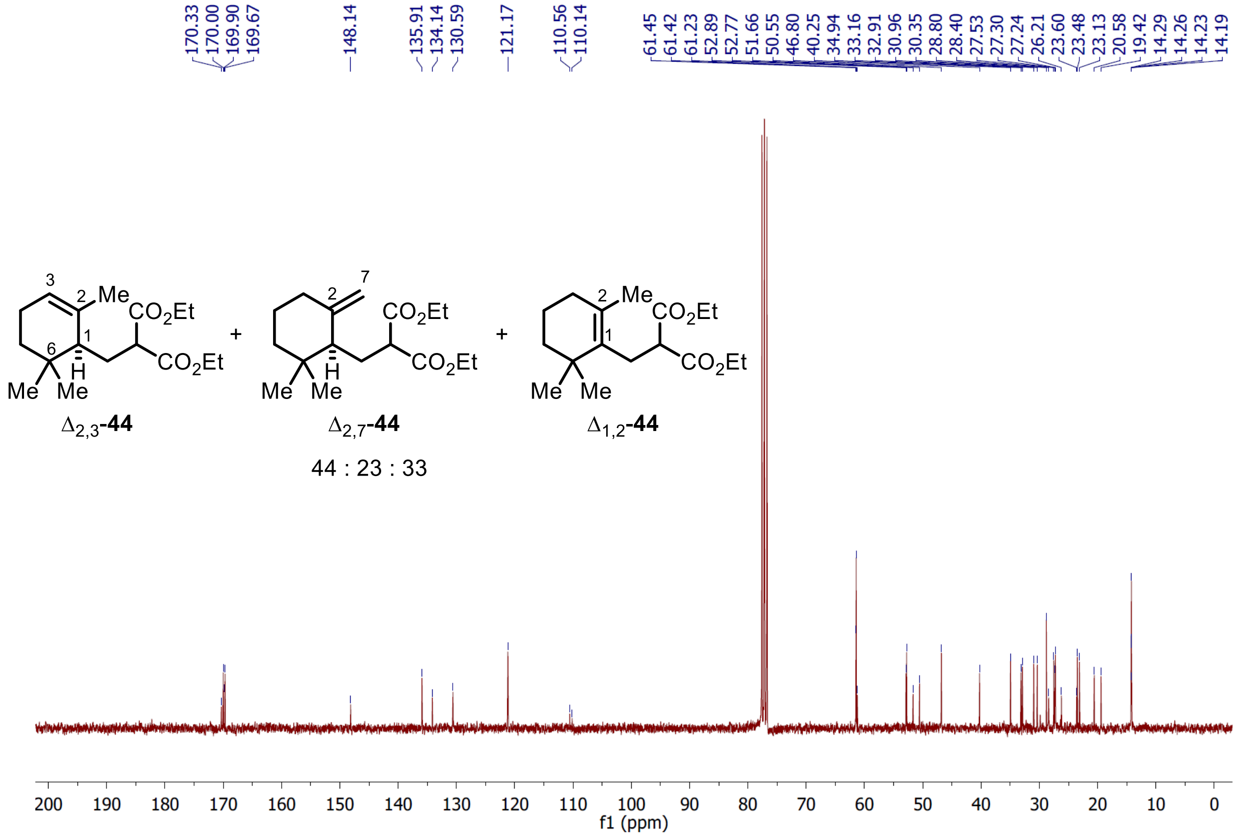
Δ2,3-44
Δ2,7-44
Δ1,2-44
44 : 23 : 33
f1 (ppm)

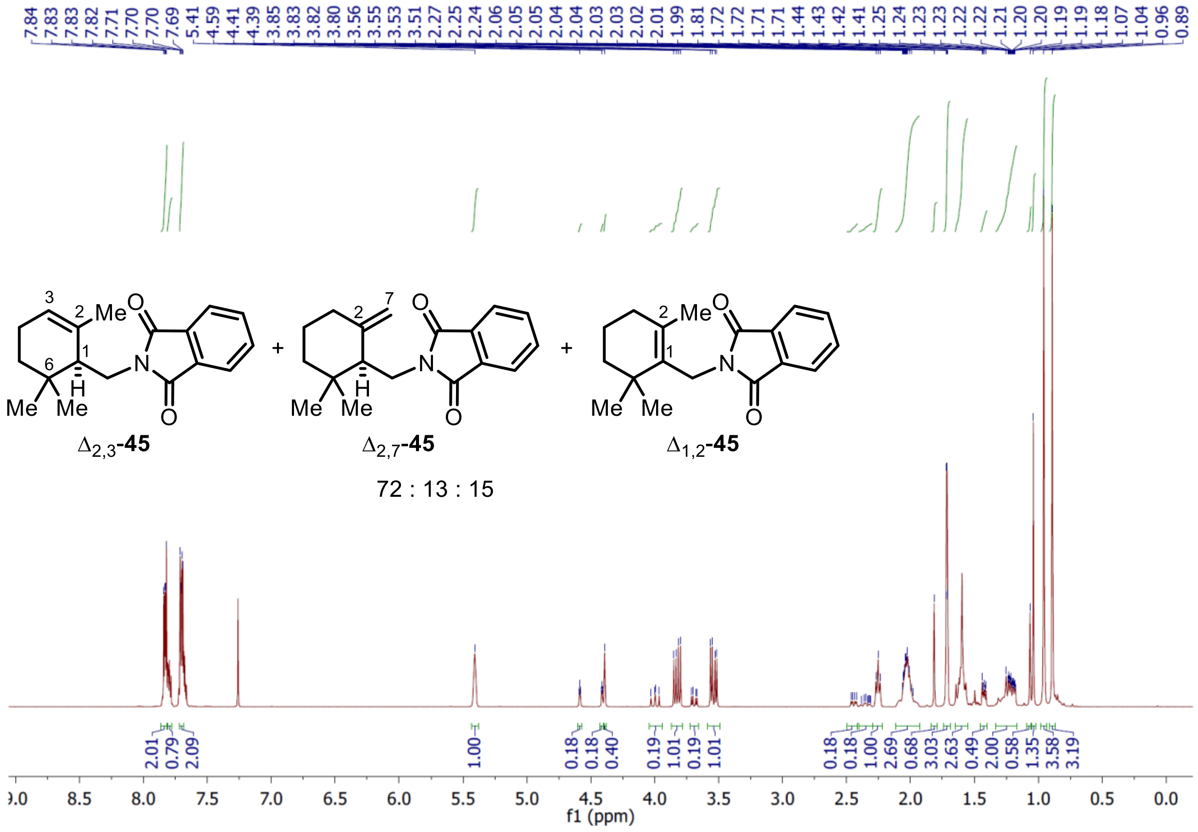

7.84 7.83 7.83 7.82 7.71 7.70 7.70 7.69 5.41 4.59 4.41 4.39 3.85 3.83 3.82 3.80 3.56 3.55 3.53 3.51 2.27 2.25 2.24 2.06 2.05 2.05 2.04 2.04 2.03 2.03 2.02 2.01 1.99 1.81 1.72 1.72 1.71 1.71 1.44 1.43 1.42 1.41 1.25 1.24 1.23 1.23 1.22 1.22 1.21 1.20 1.20 1.19 1.19 1.18 1.07 1.04 0.96 0.89
Δ2,3-45
Δ2,7-45
Δ1,2-45
72 : 13 : 15
2.01 0.79 2.09 1.00 0.18 0.18 0.40 0.19 1.01 0.19 1.01 0.18 0.18 1.00 2.69 0.68 3.03 2.63 0.49 2.00 0.58 1.35 3.58 3.19
f1 (ppm)

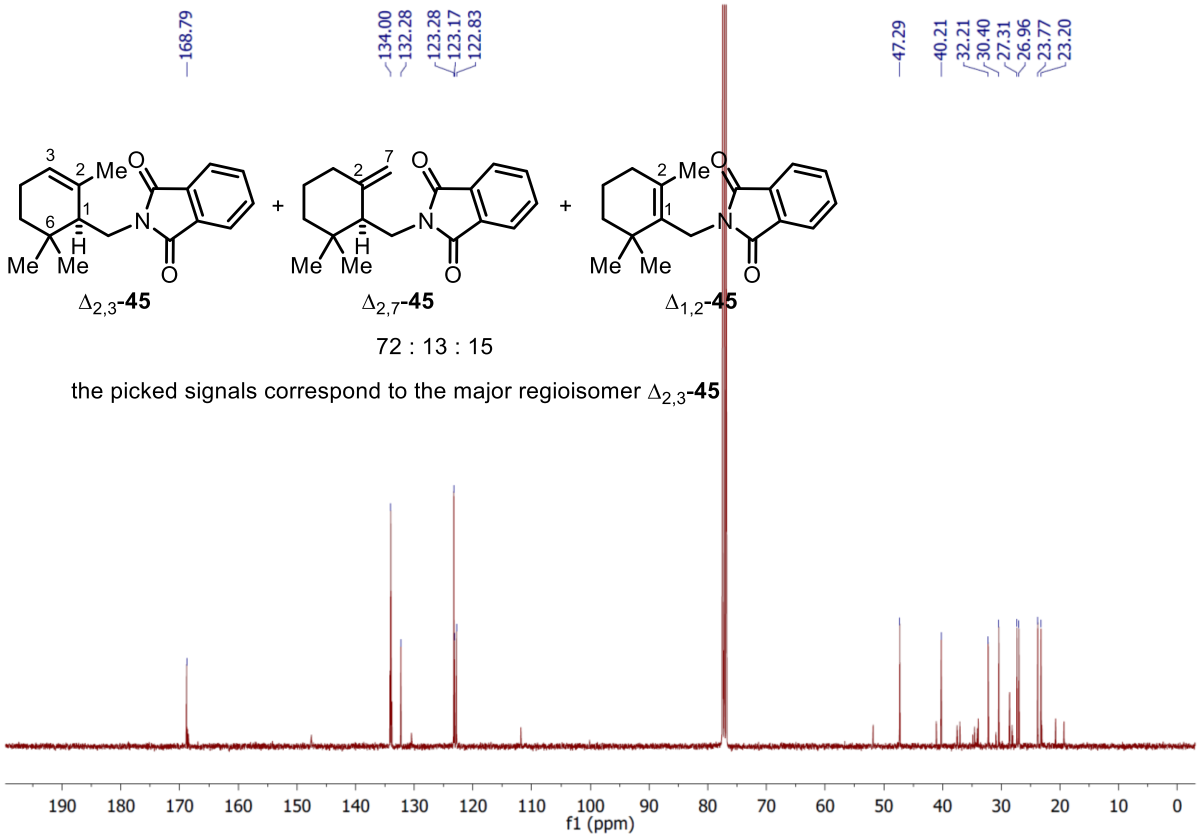

168.79
134.00 132.28 123.28 123.17 122.83
47.29 40.21 32.21 30.40 27.31 26.96 23.77 23.20
Δ2,3-45
Δ2,7-45
Δ1,2-45
72 : 13 : 15
the picked signals correspond to the major regioisomer Δ2,3-45
f1 (ppm)

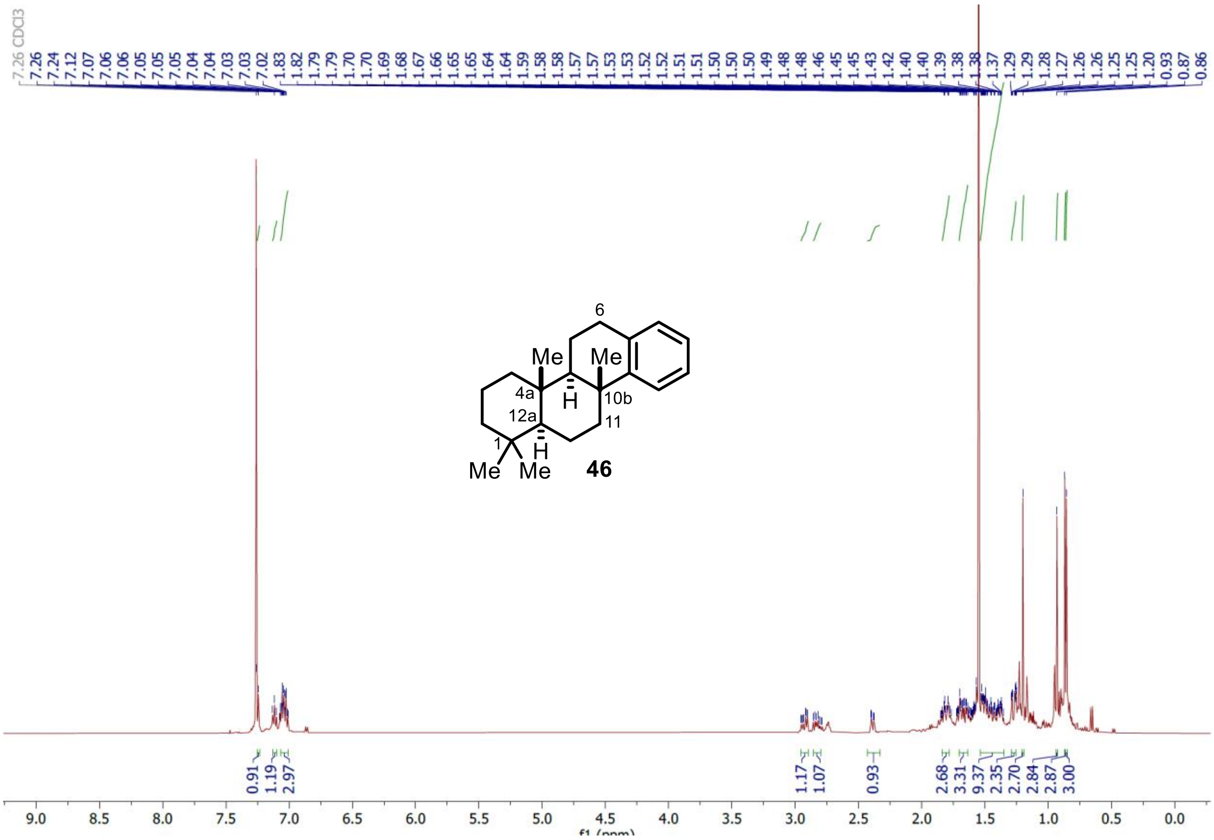
7.26 CDCl3
7.26
7.24
7.12
7.07
7.06
7.06
7.05
7.05
7.05
7.04
7.04
7.03
7.03
7.02
1.83
1.82
1.79
1.79
1.70
1.70
1.69
1.68
1.67
1.66
1.65
1.65
1.64
1.64
1.59
1.58
1.58
1.57
1.57
1.53
1.53
1.52
1.52
1.51
1.51
1.50
1.50
1.50
1.49
1.48
1.48
1.46
1.45
1.45
1.43
1.42
1.40
1.40
1.39
1.38
1.38
1.37
1.29
1.29
1.28
1.27
1.26
1.25
1.25
1.20
0.93
0.87
0.86
6
Me
Me
4a
10b
12a
11
1
H
H
Me
Me
46
0.91
1.19
2.97
1.17
1.07
0.93
2.68
3.31
9.37
2.35
2.70
2.84
2.87
3.00
9.0
8.5
8.0
7.5
7.0
6.5
6.0
5.5
5.0
4.5
4.0
3.5
3.0
2.5
2.0
1.5
1.0
0.5
0.0
f1 (ppm)

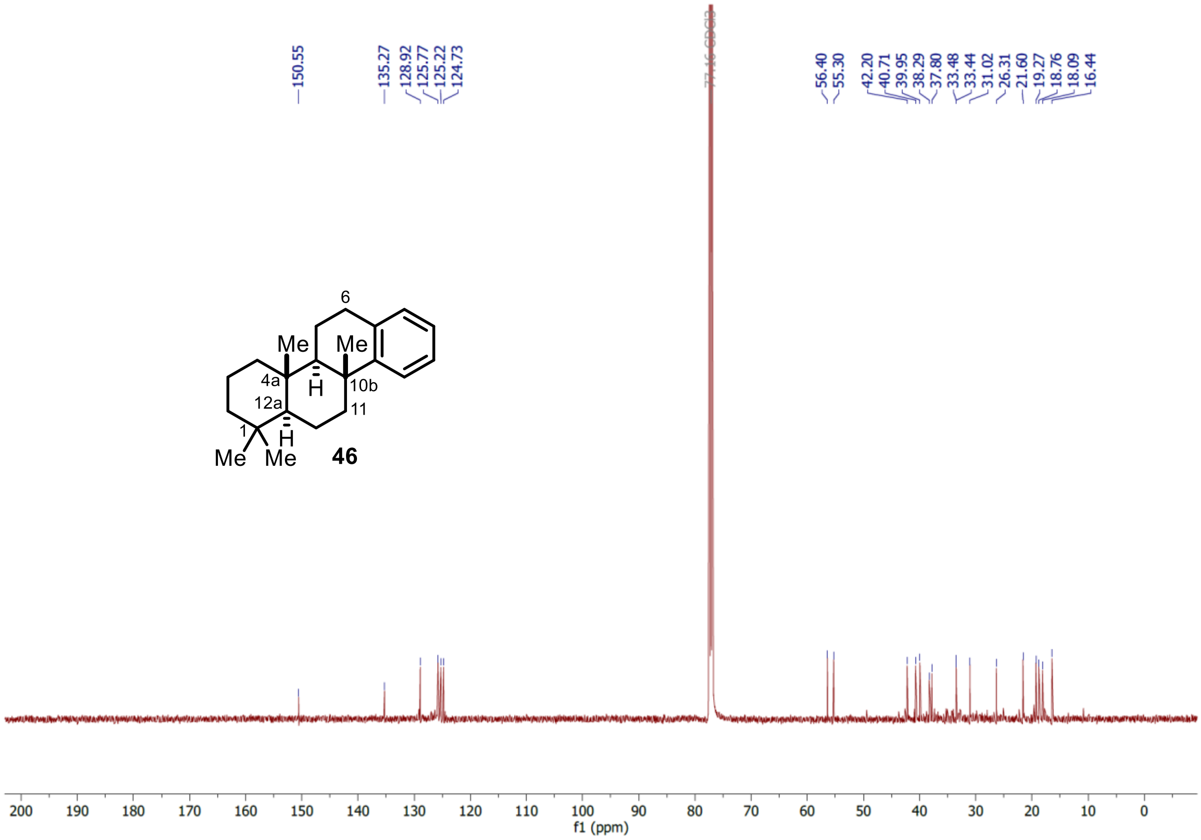
150.55
135.27
128.92
125.77
125.22
124.73
77.16 CDCl3
56.40
55.30
42.20
40.71
39.95
38.29
37.80
33.48
33.44
31.02
26.31
21.60
19.27
18.76
18.09
16.44
6
Me
Me
4a
10b
12a
11
1
H
H
Me
Me
46
200
190
180
170
160
150
140
130
120
110
100
90
80
70
60
50
40
30
20
10
0
f1 (ppm)

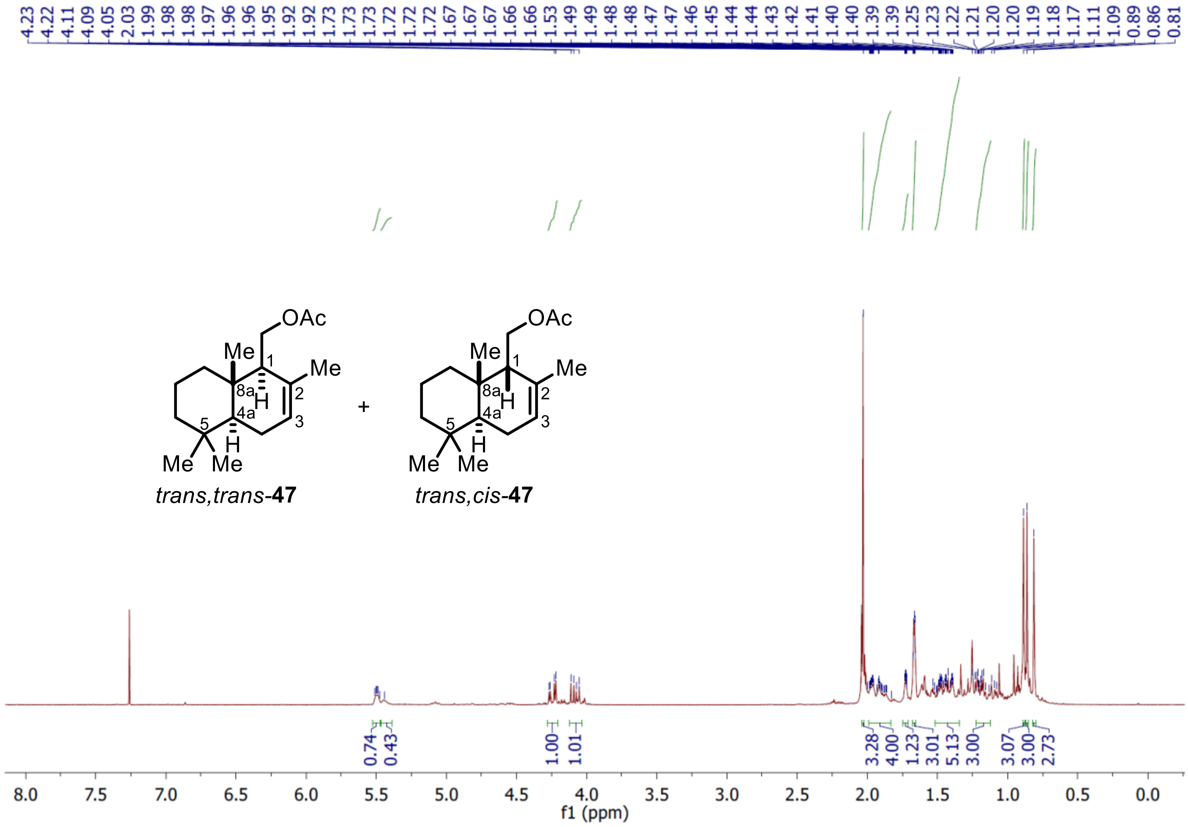
4.23 4.22 4.11 4.09 4.05 2.03 1.99 1.98 1.98 1.97 1.96 1.96 1.95 1.92 1.92 1.73 1.73 1.73 1.72 1.72 1.72 1.67 1.67 1.67 1.66 1.66 1.53 1.49 1.49 1.48 1.48 1.47 1.47 1.46 1.45 1.44 1.44 1.43 1.42 1.41 1.40 1.40 1.39 1.39 1.25 1.23 1.22 1.21 1.20 1.20 1.19 1.18 1.17 1.11 1.09 0.89 0.86 0.81
OAc
Me
Me
1
2
3
8a
4a
5
H
Me Me
trans,trans-47
+
trans,cis-47
0.74 0.43 1.00 1.01 3.28 4.00 1.23 3.01 5.13 3.00 3.07 3.00 2.73
f1 (ppm)

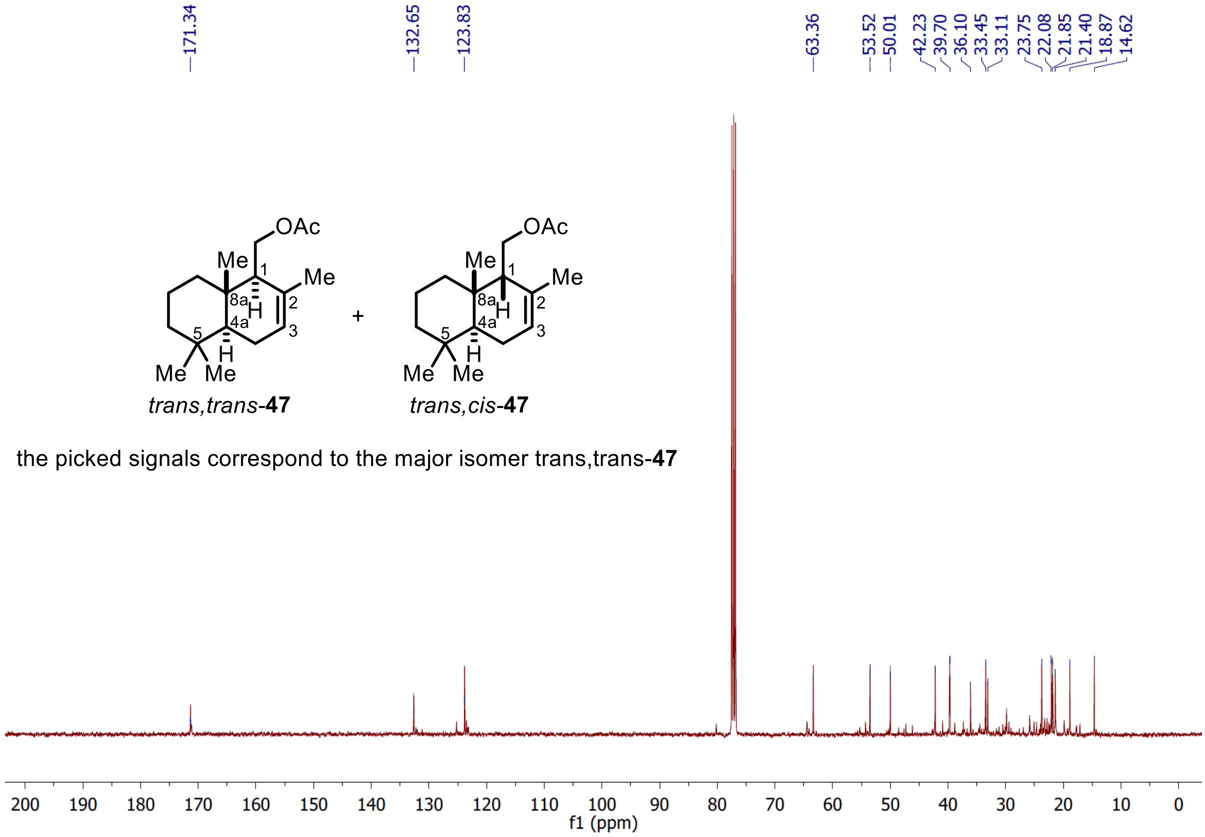
171.34 132.65 123.83 63.36 53.52 50.01 42.23 39.70 36.10 33.45 33.11 23.75 22.08 21.85 21.40 18.87 14.62
OAc
Me
Me
1
2
3
8a
4a
5
H
Me Me
trans,trans-47
+
trans,cis-47
the picked signals correspond to the major isomer trans,trans-47
f1 (ppm)

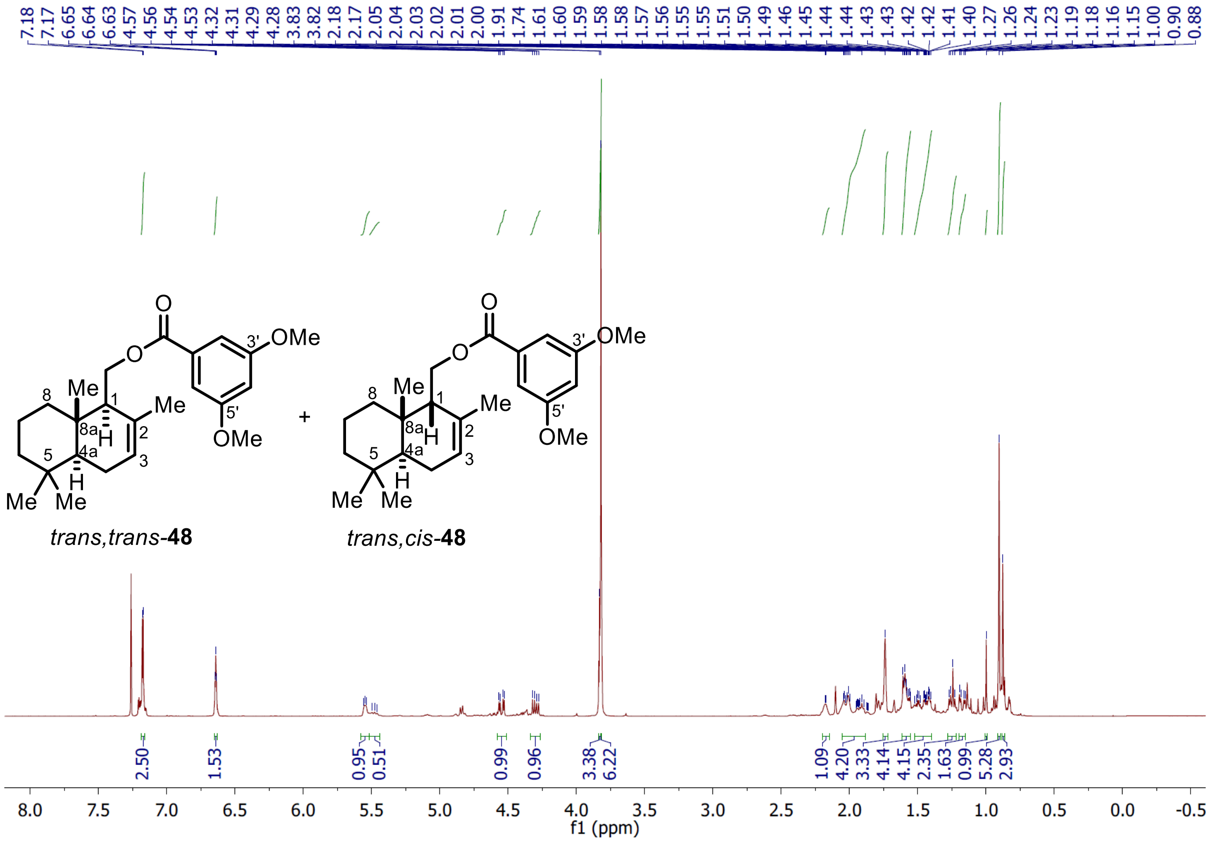

7.18 7.17 6.65 6.64 6.63 4.57 4.56 4.54 4.53 4.32 4.31 4.29 4.28 3.83 3.82 2.18 2.17 2.05 2.04 2.03 2.02 2.01 2.00 1.91 1.74 1.61 1.60 1.59 1.58 1.58 1.57 1.56 1.55 1.55 1.51 1.50 1.49 1.46 1.45 1.44 1.44 1.43 1.43 1.42 1.42 1.41 1.40 1.27 1.26 1.24 1.23 1.19 1.18 1.16 1.15 1.00 0.90 0.88
2.50 1.53 0.95 0.51 0.99 0.96 3.38 6.22 1.09 4.20 3.33 4.14 4.15 2.35 1.63 0.99 5.28 2.93
trans,trans-48
trans,cis-48
f1 (ppm)

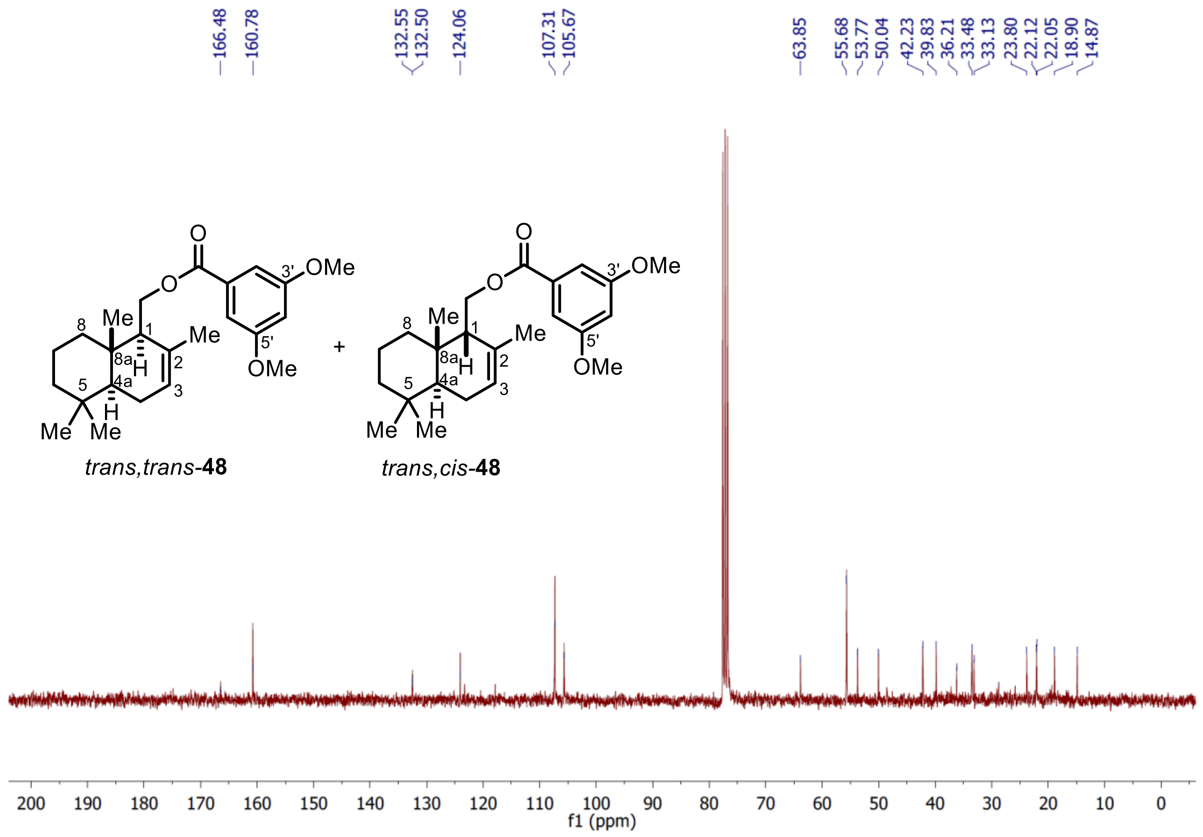

166.48 160.78 132.55 132.50 124.06 107.31 105.67 63.85 55.68 53.77 50.04 42.23 39.83 36.21 33.48 33.13 23.80 22.12 22.05 18.90 14.87
trans,trans-48
trans,cis-48
f1 (ppm)

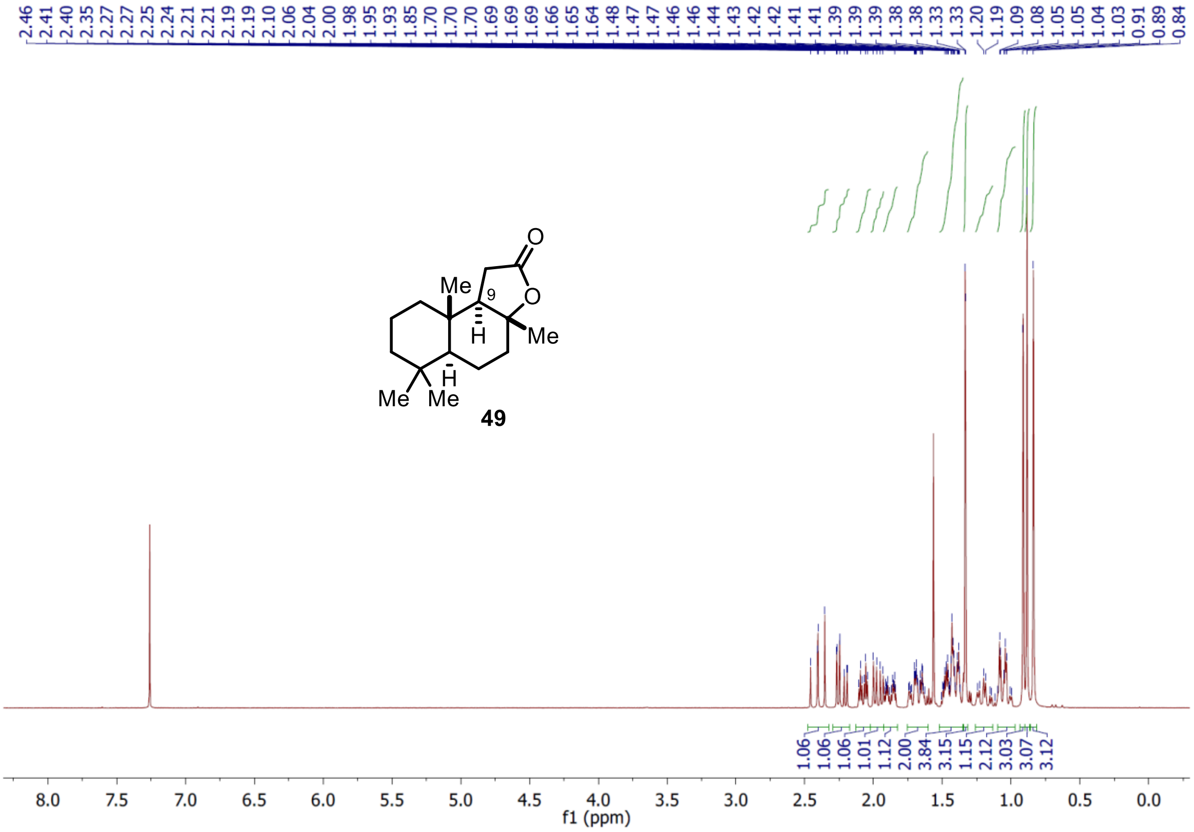

2.46 2.41 2.40 2.35 2.27 2.27 2.25 2.24 2.21 2.21 2.19 2.19 2.10 2.06 2.04 2.00 1.98 1.95 1.93 1.85 1.70 1.70 1.70 1.69 1.69 1.69 1.66 1.65 1.64 1.48 1.47 1.47 1.46 1.46 1.44 1.43 1.42 1.42 1.41 1.41 1.39 1.39 1.39 1.38 1.38 1.33 1.33 1.20 1.19 1.09 1.08 1.05 1.05 1.04 1.03 0.91 0.89 0.84
O
O
Me
9
H
Me
H
Me
Me
49
1.06 1.06 1.06 1.01 1.12 2.00 3.84 3.15 1.15 2.12 3.03 3.07 3.12
8.0 7.5 7.0 6.5 6.0 5.5 5.0 4.5 4.0 3.5 3.0 2.5 2.0 1.5 1.0 0.5 0.0
f1 (ppm)

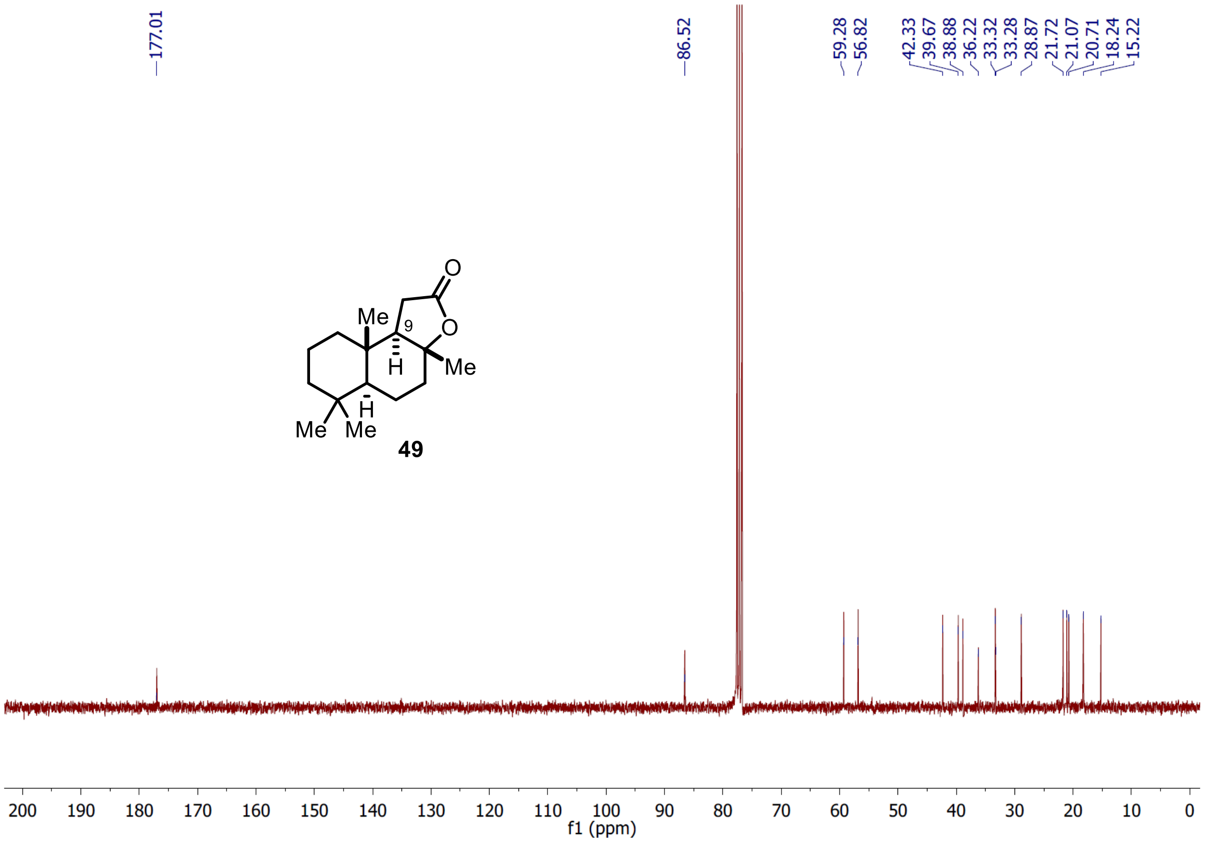

177.01
86.52
59.28 56.82
42.33 39.67 38.88 36.22 33.32 33.28 28.87 21.72 21.07 20.71 18.24 15.22
O
O
Me
9
H
Me
H
Me
Me
49
200 190 180 170 160 150 140 130 120 110 100 90 80 70 60 50 40 30 20 10 0
f1 (ppm)

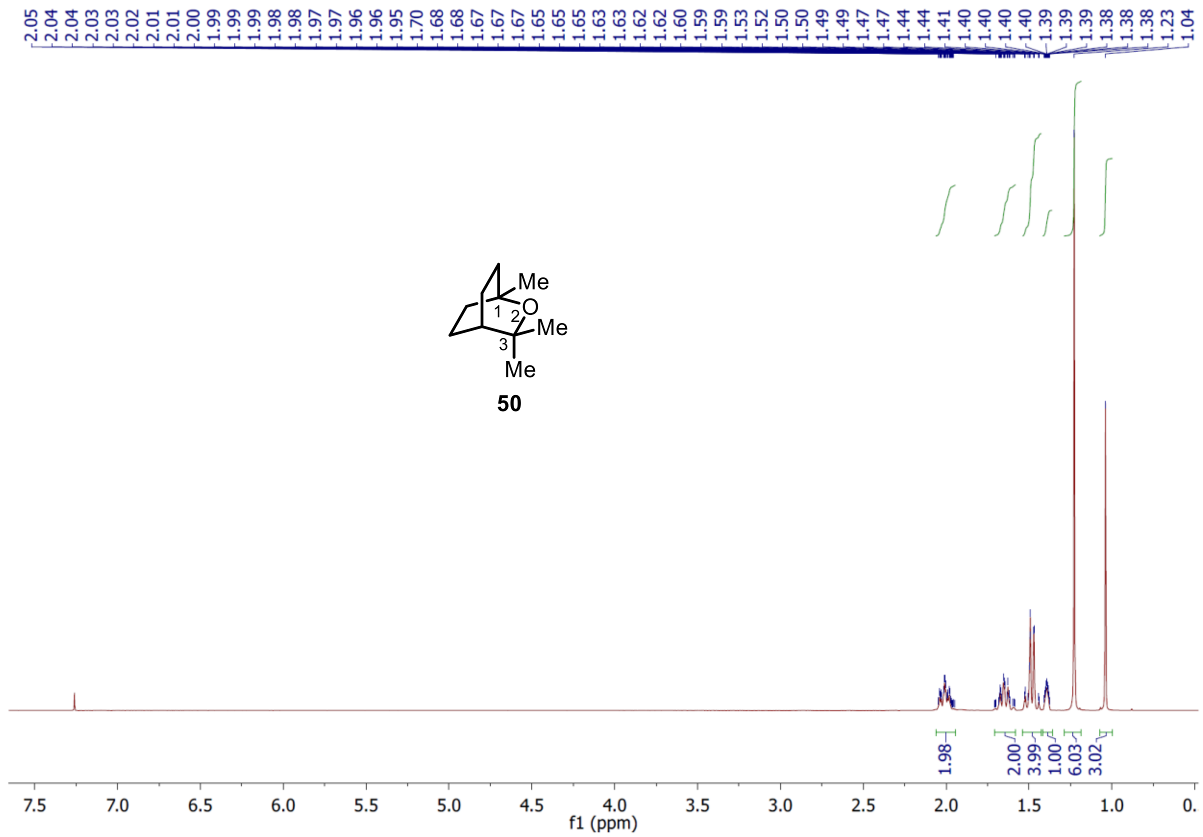
Me
O
1
2
Me
3
Me
50
1.98
2.00
3.99
1.00
6.03
3.02
f1 (ppm)

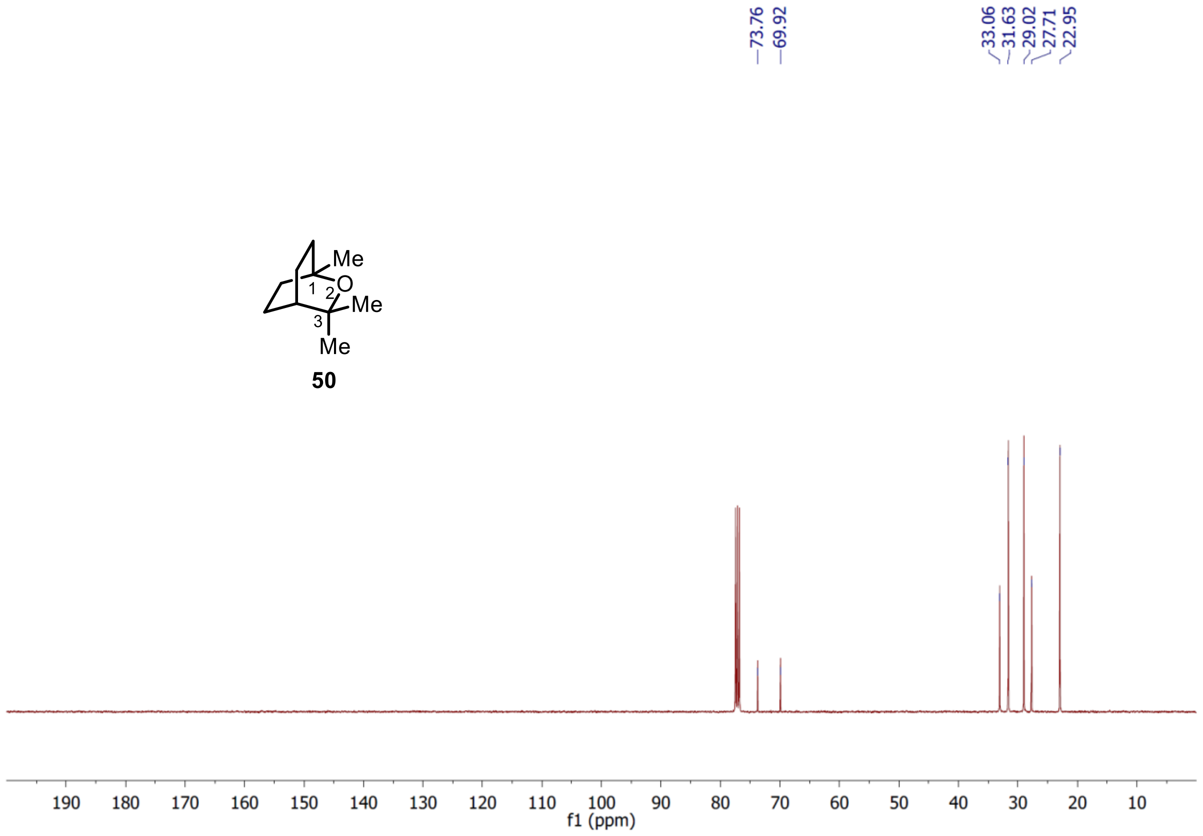
73.76
69.92
33.06
31.63
29.02
27.71
22.95
Me
O
1
2
Me
3
Me
50
f1 (ppm)

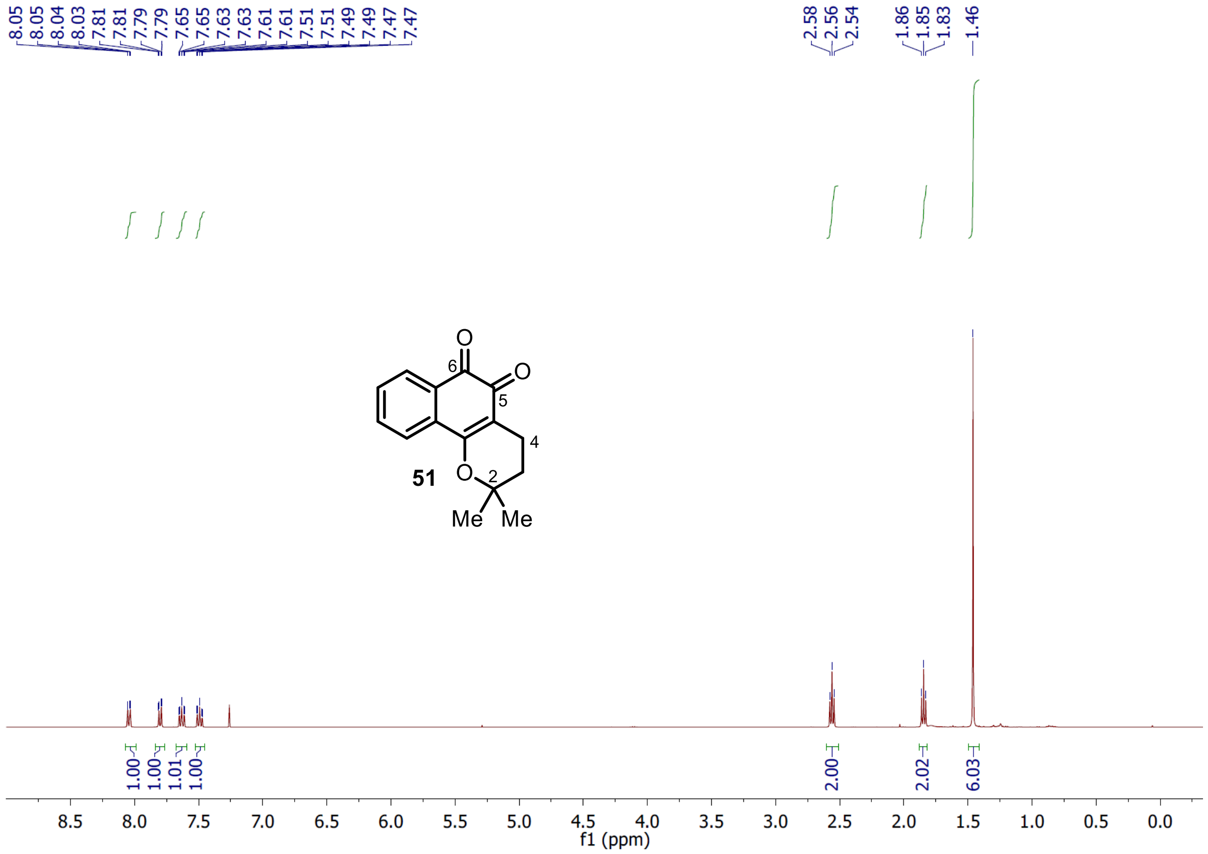
8.05
8.05
8.04
8.03
7.81
7.79
7.79
7.65
7.65
7.63
7.63
7.61
7.61
7.51
7.51
7.49
7.49
7.47
7.47
2.58
2.56
2.54
1.86
1.85
1.83
1.46
6
5
4
2
O
O
O
51
Me
Me
1.00
1.00
1.01
1.00
2.00
2.02
6.03
8.5
8.0
7.5
7.0
6.5
6.0
5.5
5.0
4.5
4.0
3.5
3.0
2.5
2.0
1.5
1.0
0.5
0.0
f1 (ppm)

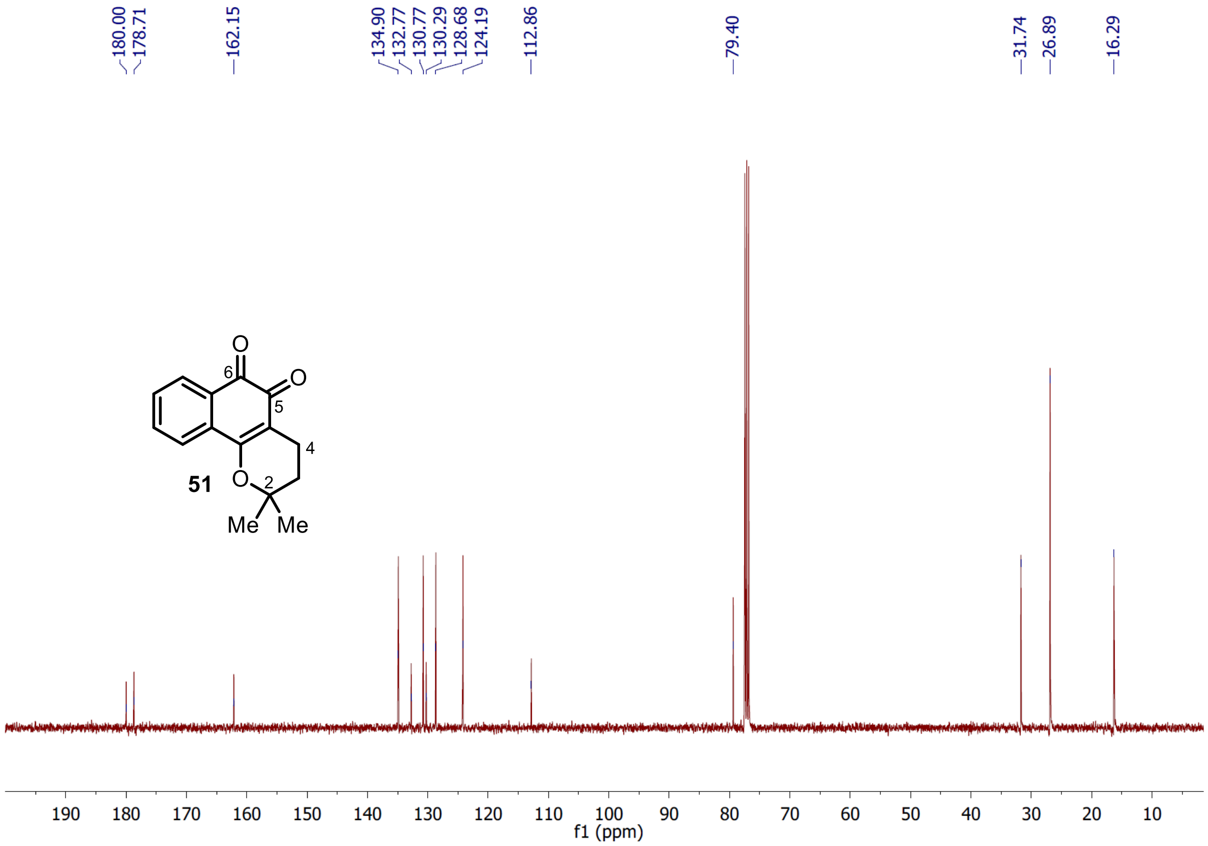
180.00
178.71
162.15
134.90
132.77
130.77
130.29
128.68
124.19
112.86
79.40
31.74
26.89
16.29
6
5
4
2
O
O
O
51
Me
Me
190
180
170
160
150
140
130
120
110
100
90
80
70
60
50
40
30
20
10
f1 (ppm)
