# Supplementary material for: Enzyme-like polyene cyclizations catalyzed by dynamic, self-assembled, supramolecular fluoro alcohol-amine clusters
Source: Nat Commun. 2023 Feb 13;14:813. doi: 10.1038/s41467-023-36157-0 (PMC9925744; doi:10.1038/s41467-023-36157-0)
Supplement: Supplementary file 4 — Supplementary Data 2 [file 41467_2023_36157_MOESM4_ESM.pdf]

Enzyme-Like Polyene Cyclizations Catalyzed by Dynamic, Self-Assembled, Supramolecular Fluoro Alcohol-Amine Clusters

Andreas M. Arnold,[1,2] Philipp Dullinger,[3] Aniruddha Biswas,[2] Christian Jandl,[4] Dominik Horinek[3] and Tanja Gulder[1,2,4*]

[1]Biomimetic Catalysis, Department of Chemistry, Technical University Munich, Lichtenbergstraße 4, 85747 Garching, Germany
[2]Chair of Organic Chemistry, Faculty of Chemistry and Mineralogy, Leipzig University, Johannisallee 29, 04103 Leipzig, Germany[3]Institute of Physical and Theoretical Chemistry, University of Regensburg, 93040 Regensburg, Germany
[4]Catalysis Research Center, Technical University Munich, Ernst-Otto-Fischer-Straße 1, 85747 Garching, Germany

SUPPLEMENTARY DATA 2: STRUCTURAL DATA REPORT FOR COMPOUND 54 (CCDC 2205029; ARNAN8)

Crystal Structure Report for ArnAn8

A colorless fragment-like specimen of $C_{72}H_{60}F_{108}N_8O_{12}$, approximate dimensions 0.252 mm x 0.276 mm x 0.295 mm, was used for the X-ray crystallographic analysis. The X-ray intensity data were measured on a Bruker D8 Venture system equipped with a Helios optic monochromator and a Mo TXS rotating anode (λ = 0.71073 Å).

Table 1: Data collection details for ArnAn8.

Axis	dx/mm	2θ/°	ω/°	φ/°	χ/°	Width/°	Frames	Time/s	Wavelength/Å	Voltage/kV	Current/mA	Temperature/K
Phi	100.002	0.00	0.00	360.00	54.74	0.50	360	1.00	0.71073	50	50.0	102.78
Phi	100.002	27.67	256.77	360.00	23.00	0.50	720	10.00	0.71073	50	50.0	102.78
Omega	100.002	28.77	319.28	257.09	65.05	0.50	213	10.00	0.71073	50	50.0	102.78
Omega	100.002	28.77	327.43	78.51	72.25	0.50	198	10.00	0.71073	50	50.0	102.78
Omega	100.002	27.67	339.56	160.00	-44.50	0.50	377	10.00	0.71073	50	50.0	102.78
Omega	100.002	27.67	339.56	320.00	-44.50	0.50	377	10.00	0.71073	50	50.0	102.78
Omega	100.002	27.67	339.56	40.00	-44.50	0.50	377	10.00	0.71073	50	50.0	102.78
Omega	100.002	28.77	248.68	70.49	42.69	0.50	380	10.00	0.71073	50	50.0	102.78
Omega	100.002	-28.77	300.00	48.38	55.12	0.50	140	10.00	0.71073	50	50.0	102.78
Omega	100.002	-28.77	305.51	252.08	59.59	0.50	126	10.00	0.71073	50	50.0	102.78
Omega	100.002	28.77	350.43	298.47	-56.68	0.50	140	10.00	0.71073	50	50.0	102.78
Phi	100.002	27.67	276.44	0.00	-23.00	0.50	720	10.00	0.71073	50	50.0	102.78
Omega	100.002	-28.77	303.30	311.62	57.63	0.50	131	10.00	0.71073	50	50.0	102.78
Omega	100.002	-28.77	302.57	80.28	57.18	0.50	133	10.00	0.71073	50	50.0	102.78
Omega	100.002	-28.77	301.89	212.98	56.55	0.50	135	10.00	0.71073	50	50.0	102.78
Omega	100.002	27.67	339.56	120.00	-44.50	0.50	377	10.00	0.71073	50	50.0	102.78
Omega	100.002	27.67	339.56	360.00	-44.50	0.50	377	10.00	0.71073	50	50.0	102.78
Omega	100.002	27.67	339.56	200.00	-44.50	0.50	377	10.00	0.71073	50	50.0	102.78
Omega	100.002	27.67	339.56	280.00	-44.50	0.50	218	10.00	0.71073	50	50.0	102.78

A total of 5876 frames were collected. The total exposure time was 15.42 hours. The frames were integrated with the Bruker SAINT software package using a narrow-frame algorithm. The integration of the data using a monoclinic unit cell yielded a total of 188659 reflections to a maximum θ angle of 25.35° (0.83 Å resolution), of which 20316 were independent (average redundancy 9.286, completeness = 99.9%, R_{int} = 3.65%, R_{sig} = 2.10%) and 19605 (96.50%) were greater than $2\sigma(F^2)$. The final cell constants of a = 51.072(5) Å, b = 10.0761(11) Å, c = 22.372(3) Å, β = 105.274(4)°, volume = 11106.(2) $Å^3$, are based upon the refinement of the XYZ-centroids of 216 reflections above 20 σ(I) with 4.336° < 2θ < 32.43°. Data were corrected for absorption effects using the Multi-Scan method (SADABS). The ratio of minimum to maximum apparent transmission was 0.932. The calculated minimum and maximum transmission coefficients (based on crystal size) are 0.9300 and 0.9390.

The structure was solved and refined using the Bruker SHELXTL Software Package in conjunction with SHELXLE, using the space group C 1 c 1, with Z = 4 for the formula unit, $C_{72}H_{60}F_{108}N_8O_{12}$. The final anisotropic full-matrix least-squares refinement on F^2 with 2217 variables converged at R1 = 3.27%, for the observed data and wR2 = 8.87% for all data. The goodness-of-fit was 1.015. The largest peak in the final difference electron density synthesis was 0.420 $e^-/Å^3$ and the largest hole was -0.296 $e^-/Å^3$ with an RMS deviation of 0.049 $e^-/Å^3$. On the basis of the final model, the calculated density was 1.962 g/cm^3 and F(000), 6464 e^-.

Figure 1. Ortep drawing with 50% ellipsoids for ArnAn8.

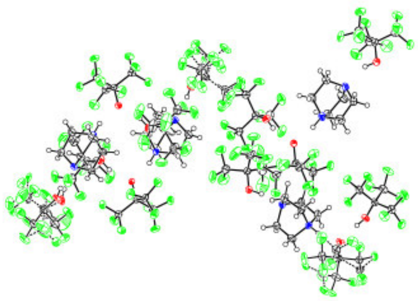

Table 2. Sample and crystal data for ArnAn8.

**Identification code**	ArnAn8	
**Chemical formula**	$C_{72}H_{60}F_{108}N_8O_{12}$	
**Formula weight**	3281.28	
**Temperature**	103(2) K	
**Wavelength**	0.71073 Å	
**Crystal size**	0.252 x 0.276 x 0.295 mm	
**Crystal habit**	colorless fragment	
**Crystal system**	monoclinic	
**Space group**	C 1 c 1	
**Unit cell dimensions**	a = 51.072(5) Å	α = 90°
	b = 10.0761(11) Å	β = 105.274(4)°
	c = 22.372(3) Å	γ = 90°
**Volume**	11106.(2) $Å^3$	
**Z**	4	
**Density (calculated)**	1.962 g/cm^3	
**Absorption coefficient**	0.251 mm^{-1}	
**F(000)**	6464	

Table 3. Data collection and structure refinement for ArnAn8.

**Diffractometer**	Bruker D8 Venture	
**Radiation source**	TXS rotating anode, Mo	
**Theta range for data collection**	2.06 to 25.35°	
**Index ranges**	-61<=h<=61, -12<=k<=12, -26<=l<=26	
**Reflections collected**	188659	
**Independent reflections**	20316 [R(int) = 0.0365]	
**Coverage of independent reflections**	99.9%	
**Absorption correction**	Multi-Scan	
**Max. and min. transmission**	0.9390 and 0.9300	
**Structure solution technique**	direct methods	
**Structure solution program**	SHELXT 2014/5 (Sheldrick, 2014)	
**Refinement method**	Full-matrix least-squares on F^2	
**Refinement program**	SHELXL-2017/1 (Sheldrick, 2017)	
**Function minimized**	$\Sigma w(F_o^2 - F_c^2)^2$	
**Data / restraints / parameters**	20316 / 28488 / 2217	
**Goodness-of-fit on F^2**	1.015	
**Δ/σ_{max}**	0.001	
**Final R indices**	19605 data; I>2σ(I)	R1 = 0.0327, wR2 = 0.0874
	all data	R1 = 0.0340, wR2 = 0.0887
**Weighting scheme**	$w=1/[\sigma^2(F_o^2)+(0.0563P)^2+8.9206P]$ where $P=(F_o^2+2F_c^2)/3$	
**Absolute structure parameter**	0.5(4)	
**Largest diff. peak and hole**	0.420 and -0.296 $eÅ^{-3}$	
**R.M.S. deviation from mean**	0.049 $eÅ^{-3}$	

Table 4. Bond lengths (Å) for ArnAn8.

N1-C3	1.461(5)	N1-C1	1.469(5)
N1-C5	1.475(5)	C1-C2	1.534(6)
C1-H1A	0.99	C1-H1B	0.99
N2-C4	1.487(5)	N2-C2	1.490(5)
N2-C6	1.498(5)	N2-H2N	0.94(5)
C2-H2A	0.99	C2-H2B	0.99
N3-C9	1.485(4)	N3-C11	1.487(5)
N3-C7	1.496(5)	N3-H3N	0.92(5)
C3-C4	1.536(5)	C3-H3A	0.99
C3-H3B	0.99	N7-C21	1.482(5)
N7-C23	1.486(5)	N7-C19	1.488(5)
N7-H7N	0.88(3)	C7-C8	1.530(5)
C7-H7A	0.99	C7-H7B	0.99
N6-C18	1.473(5)	N6-C16	1.486(5)
N6-C14	1.503(5)	N6-H6N	0.88(5)
C6-C5	1.529(6)	C6-H6A	0.99
C6-H6B	0.99	C8-N4	1.474(4)
C8-H8A	0.99	C8-H8B	0.99
N8-C22	1.469(4)	N8-C24	1.471(4)
N8-C20	1.472(4)	C9-C10	1.531(5)

C9-H9A	0.99	C9-H9B	0.99
C12-N4	1.479(5)	C12-C11	1.534(5)
C12-H12A	0.99	C12-H12B	0.99
C11-H11A	0.99	C11-H11B	0.99
C10-N4	1.482(4)	C10-H10A	0.99
C10-H10B	0.99	C17-N5	1.461(5)
C17-C18	1.537(6)	C17-H17A	0.99
C17-H17B	0.99	C16-C15	1.539(5)
C16-H16A	0.99	C16-H16B	0.99
C15-N5	1.462(5)	C15-H15A	0.99
C15-H15B	0.99	C14-C13	1.531(5)
C14-H14A	0.99	C14-H14B	0.99
C13-N5	1.463(5)	C13-H13A	0.99
C13-H13B	0.99	C4-H4A	0.99
C4-H4B	0.99	C21-C22	1.530(5)
C21-H21A	0.99	C21-H21B	0.99
C23-C24	1.533(5)	C23-H23A	0.99
C23-H23B	0.99	C24-H24A	0.99
C24-H24B	0.99	C22-H22A	0.99
C22-H22B	0.99	C20-C19	1.535(5)
C20-H20A	0.99	C20-H20B	0.99
C19-H19A	0.99	C19-H19B	0.99
C18-H18A	0.99	C18-H18B	0.99
C5-H5A	0.99	C5-H5B	0.99
O1_1-C1_1	1.348(4)	C1_1-C2_1	1.553(4)
C1_1-C4_1	1.554(4)	C1_1-C3_1	1.562(4)
C2_1-F3_1	1.331(4)	C2_1-F1_1	1.333(4)
C2_1-F2_1	1.334(4)	C3_1-F6_1	1.326(4)
C3_1-F5_1	1.330(4)	C3_1-F4_1	1.335(4)
C4_1-F9_1	1.330(4)	C4_1-F8_1	1.336(4)
C4_1-F7_1	1.338(4)	O1_2-C1_2	1.367(4)
O1_2-H1_2	0.84(6)	C1_2-C2_2	1.550(4)
C1_2-C3_2	1.551(4)	C1_2-C4_2	1.553(5)
C2_2-F1_2	1.325(5)	C2_2-F2_2	1.327(4)
C2_2-F3_2	1.334(5)	C3_2-F6_2	1.318(4)
C3_2-F5_2	1.326(5)	C3_2-F4_2	1.330(4)
C4_2-F7_2	1.320(5)	C4_2-F9_2	1.334(5)
C4_2-F8_2	1.334(4)	O1_3-C1_3	1.380(4)
O1_3-H1_3	0.93(7)	C1_3-C3_3	1.549(4)
C1_3-C2_3	1.550(4)	C1_3-C4_3	1.554(4)
C2_3-F2_3	1.328(4)	C2_3-F1_3	1.334(4)
C2_3-F3_3	1.349(4)	C3_3-F5_3	1.331(4)
C3_3-F6_3	1.332(4)	C3_3-F4_3	1.342(4)
C4_3-F7_3	1.326(4)	C4_3-F9_3	1.335(4)
C4_3-F8_3	1.343(4)	O1_4-C1_4	1.374(4)
O1_4-H1_4	0.84(6)	C1_4-C4_4	1.536(5)
C1_4-C3_4	1.548(5)	C1_4-C2_4	1.561(5)
C2_4-F1_4	1.325(5)	C2_4-F3_4	1.332(5)
C2_4-F2_4	1.337(5)	C3_4-F5_4	1.315(4)
C3_4-F6_4	1.336(4)	C3_4-F4_4	1.341(5)
C4_4-F8_4	1.320(5)	C4_4-F7_4	1.322(6)
C4_4-F9_4	1.346(5)	O1_5-C1_5	1.354(4)
C1_5-C4_5	1.550(4)	C1_5-C2_5	1.554(4)
C1_5-C3_5	1.558(4)	C2_5-F2_5	1.333(4)
C2_5-F3_5	1.336(4)	C2_5-F1_5	1.336(4)
C3_5-F6_5	1.326(4)	C3_5-F5_5	1.329(4)
C3_5-F4_5	1.336(4)	C4_5-F7_5	1.332(4)
C4_5-F9_5	1.336(4)	C4_5-F8_5	1.341(4)
O1_6-C1_6	1.381(4)	O1_6-H1_6	0.86(6)
C1_6-C2_6	1.540(4)	C1_6-C3_6	1.547(5)
C1_6-C4_6	1.550(4)	C2_6-F1_6	1.322(5)
C2_6-F2_6	1.328(4)	C2_6-F3_6	1.330(5)
C3_6-F5_6	1.323(5)	C3_6-F6_6	1.331(4)
C3_6-F4_6	1.332(5)	C4_6-F7_6	1.321(5)
C4_6-F8_6	1.324(4)	C4_6-F9_6	1.330(5)
O1_7-C1_7	1.384(4)	O1_7-H1_7	0.76(5)
C1_7-C2_7	1.549(4)	C1_7-C3_7	1.550(4)
C1_7-C4_7	1.555(4)	C2_7-F1_7	1.326(4)
C2_7-F2_7	1.336(4)	C2_7-F3_7	1.338(4)
C3_7-F6_7	1.326(4)	C3_7-F5_7	1.329(4)
C3_7-F4_7	1.333(4)	C4_7-F7_7	1.319(4)
C4_7-F9_7	1.329(4)	C4_7-F8_7	1.342(4)
O1_8-C1_8	1.367(4)	O1_8-H1_8	0.92(5)

C1_8-C3_8	1.541(5)	C1_8-C2_8	1.543(5)
C1_8-C4_8	1.551(4)	C2_8-F3_8	1.330(4)
C2_8-F2_8	1.330(5)	C2_8-F1_8	1.334(5)
C3_8-F6_8	1.325(5)	C3_8-F4_8	1.328(6)
C3_8-F5_8	1.335(5)	C4_8-F7_8	1.318(5)
C4_8-F9_8	1.322(4)	C4_8-F8_8	1.332(4)
O1_9-C1_9	1.357(6)	C1_9-C4_9	1.553(6)
C1_9-C3_9	1.554(6)	C1_9-C2_9	1.562(6)
C2_9-F1_9	1.323(7)	C2_9-F2_9	1.326(7)
C2_9-F3_9	1.338(6)	C3_9-F5_9	1.331(8)
C3_9-F6_9	1.334(6)	C3_9-F4_9	1.341(6)
C4_9-F7_9	1.328(8)	C4_9-F9_9	1.332(6)
C4_9-F8_9	1.337(6)	O1_10-C1_10	1.368(4)
O1_10-H1_10	0.83(5)	C1_10-C2B_10	1.517(16)
C1_10-C4A_10	1.525(18)	C1_10-C3A_10	1.557(13)
C1_10-C3B_10	1.569(16)	C1_10-C4B_10	1.575(15)
C1_10-C2A_10	1.587(14)	C2A_10-F1A_10	1.322(10)
C2A_10-F3A_10	1.326(10)	C2A_10-F2A_10	1.340(10)
C2B_10-F1B_10	1.317(10)	C2B_10-F3B_10	1.332(10)
C2B_10-F2B_10	1.345(10)	C3A_10-F5A_10	1.308(9)
C3A_10-F4A_10	1.328(9)	C3A_10-F6A_10	1.341(9)
C3B_10-F5B_10	1.312(12)	C3B_10-F4B_10	1.317(12)
C3B_10-F6B_10	1.336(11)	C4A_10-F8A_10	1.313(11)
C4A_10-F9A_10	1.334(11)	C4A_10-F7A_10	1.339(10)
C4B_10-F8B_10	1.310(9)	C4B_10-F7B_10	1.334(10)
C4B_10-F9B_10	1.344(10)	O1_11-C1_11	1.347(4)
C1_11-C2_11	1.554(4)	C1_11-C4_11	1.555(4)
C1_11-C3_11	1.559(4)	C2_11-F2_11	1.331(4)
C2_11-F1_11	1.331(4)	C2_11-F3_11	1.339(5)
C3_11-F6_11	1.332(4)	C3_11-F4_11	1.335(4)
C3_11-F5_11	1.337(4)	C4_11-F9_11	1.330(4)
C4_11-F7_11	1.335(4)	C4_11-F8_11	1.345(4)
O1_12-C1_12	1.371(5)	O1_12-H1_12	0.85(3)
C1_12-C4_12	1.538(6)	C1_12-C2_12	1.543(6)
C1_12-C3_12	1.551(6)	C2_12-F2_12	1.321(6)
C2_12-F1_12	1.330(5)	C2_12-F3_12	1.331(6)
C3_12-F6_12	1.315(6)	C3_12-F5_12	1.322(6)
C3_12-F4_12	1.342(6)	C4_12-F7_12	1.321(6)
C4_12-F8_12	1.332(6)	C4_12-F9_12	1.339(6)
O1_13-C1_13	1.362(9)	C1_13-C2_13	1.551(8)
C1_13-C4_13	1.554(8)	C1_13-C3_13	1.559(8)
C2_13-F2_13	1.330(9)	C2_13-F1_13	1.332(8)
C2_13-F3_13	1.333(9)	C3_13-F5_13	1.324(8)
C3_13-F6_13	1.325(9)	C3_13-F4_13	1.332(9)
C4_13-F7_13	1.321(8)	C4_13-F9_13	1.334(9)
C4_13-F8_13	1.344(8)	O1_14-C1_14	1.362(9)
O1_14-H1_14	0.84(3)	C1_14-C3_14	1.546(9)
C1_14-C2_14	1.547(9)	C1_14-C4_14	1.550(9)
C2_14-F3_14	1.319(9)	C2_14-F1_14	1.320(9)
C2_14-F2_14	1.331(9)	C3_14-F6_14	1.318(9)
C3_14-F5_14	1.324(9)	C3_14-F4_14	1.334(9)
C4_14-F7_14	1.322(9)	C4_14-F9_14	1.328(9)
C4_14-F8_14	1.334(9)		

Table 5. Bond angles (°) for ArnAn8.

C3-N1-C1	109.4(3)	C3-N1-C5	107.9(3)
C1-N1-C5	108.8(3)	N1-C1-C2	111.2(3)
N1-C1-H1A	109.4	C2-C1-H1A	109.4
N1-C1-H1B	109.4	C2-C1-H1B	109.4
H1A-C1-H1B	108.0	C4-N2-C2	110.3(3)
C4-N2-C6	108.7(3)	C2-N2-C6	108.9(3)
C4-N2-H2N	111.(3)	C2-N2-H2N	105.(3)
C6-N2-H2N	113.(3)	N2-C2-C1	108.5(3)
N2-C2-H2A	110.0	C1-C2-H2A	110.0
N2-C2-H2B	110.0	C1-C2-H2B	110.0
H2A-C2-H2B	108.4	C9-N3-C11	109.6(3)
C9-N3-C7	109.7(3)	C11-N3-C7	109.4(3)
C9-N3-H3N	112.(3)	C11-N3-H3N	108.(3)
C7-N3-H3N	108.(3)	N1-C3-C4	110.8(3)
N1-C3-H3A	109.5	C4-C3-H3A	109.5

N1-C3-H3B	109.5	C4-C3-H3B	109.5
H3A-C3-H3B	108.1	C21-N7-C23	109.4(3)
C21-N7-C19	107.9(3)	C23-N7-C19	110.6(3)
C21-N7-H7N	107.(3)	C23-N7-H7N	111.(3)
C19-N7-H7N	110.(3)	N3-C7-C8	108.6(3)
N3-C7-H7A	110.0	C8-C7-H7A	110.0
N3-C7-H7B	110.0	C8-C7-H7B	110.0
H7A-C7-H7B	108.3	C18-N6-C16	110.3(3)
C18-N6-C14	109.3(3)	C16-N6-C14	108.4(3)
C18-N6-H6N	104.(3)	C16-N6-H6N	113.(3)
C14-N6-H6N	112.(3)	N2-C6-C5	108.3(3)
N2-C6-H6A	110.0	C5-C6-H6A	110.0
N2-C6-H6B	110.0	C5-C6-H6B	110.0
H6A-C6-H6B	108.4	N4-C8-C7	110.8(3)
N4-C8-H8A	109.5	C7-C8-H8A	109.5
N4-C8-H8B	109.5	C7-C8-H8B	109.5
H8A-C8-H8B	108.1	C22-N8-C24	108.8(3)
C22-N8-C20	108.5(3)	C24-N8-C20	108.7(3)
N3-C9-C10	108.5(3)	N3-C9-H9A	110.0
C10-C9-H9A	110.0	N3-C9-H9B	110.0
C10-C9-H9B	110.0	H9A-C9-H9B	108.4
N4-C12-C11	110.9(3)	N4-C12-H12A	109.5
C11-C12-H12A	109.5	N4-C12-H12B	109.5
C11-C12-H12B	109.5	H12A-C12-H12B	108.1
N3-C11-C12	108.4(3)	N3-C11-H11A	110.0
C12-C11-H11A	110.0	N3-C11-H11B	110.0
C12-C11-H11B	110.0	H11A-C11-H11B	108.4
N4-C10-C9	111.0(3)	N4-C10-H10A	109.4
C9-C10-H10A	109.4	N4-C10-H10B	109.4
C9-C10-H10B	109.4	H10A-C10-H10B	108.0
N5-C17-C18	110.9(3)	N5-C17-H17A	109.5
C18-C17-H17A	109.5	N5-C17-H17B	109.5
C18-C17-H17B	109.5	H17A-C17-H17B	108.0
N6-C16-C15	108.7(3)	N6-C16-H16A	110.0
C15-C16-H16A	110.0	N6-C16-H16B	110.0
C15-C16-H16B	110.0	H16A-C16-H16B	108.3
N5-C15-C16	110.9(3)	N5-C15-H15A	109.5
C16-C15-H15A	109.5	N5-C15-H15B	109.5
C16-C15-H15B	109.5	H15A-C15-H15B	108.0
N6-C14-C13	108.3(3)	N6-C14-H14A	110.0
C13-C14-H14A	110.0	N6-C14-H14B	110.0
C13-C14-H14B	110.0	H14A-C14-H14B	108.4
N5-C13-C14	111.3(3)	N5-C13-H13A	109.4
C14-C13-H13A	109.4	N5-C13-H13B	109.4
C14-C13-H13B	109.4	H13A-C13-H13B	108.0
N2-C4-C3	108.9(3)	N2-C4-H4A	109.9
C3-C4-H4A	109.9	N2-C4-H4B	109.9
C3-C4-H4B	109.9	H4A-C4-H4B	108.3
C8-N4-C12	107.8(3)	C8-N4-C10	108.8(3)
C12-N4-C10	109.1(3)	N7-C21-C22	109.1(3)
N7-C21-H21A	109.9	C22-C21-H21A	109.9
N7-C21-H21B	109.9	C22-C21-H21B	109.9
H21A-C21-H21B	108.3	N7-C23-C24	108.5(3)
N7-C23-H23A	110.0	C24-C23-H23A	110.0
N7-C23-H23B	110.0	C24-C23-H23B	110.0
H23A-C23-H23B	108.4	N8-C24-C23	111.0(3)
N8-C24-H24A	109.4	C23-C24-H24A	109.4
N8-C24-H24B	109.4	C23-C24-H24B	109.4
H24A-C24-H24B	108.0	N8-C22-C21	110.6(3)
N8-C22-H22A	109.5	C21-C22-H22A	109.5
N8-C22-H22B	109.5	C21-C22-H22B	109.5
H22A-C22-H22B	108.1	N8-C20-C19	110.7(3)
N8-C20-H20A	109.5	C19-C20-H20A	109.5
N8-C20-H20B	109.5	C19-C20-H20B	109.5
H20A-C20-H20B	108.1	N7-C19-C20	108.5(3)
N7-C19-H19A	110.0	C20-C19-H19A	110.0
N7-C19-H19B	110.0	C20-C19-H19B	110.0
H19A-C19-H19B	108.4	N6-C18-C17	108.9(3)
N6-C18-H18A	109.9	C17-C18-H18A	109.9
N6-C18-H18B	109.9	C17-C18-H18B	109.9
H18A-C18-H18B	108.3	N1-C5-C6	111.3(3)
N1-C5-H5A	109.4	C6-C5-H5A	109.4
N1-C5-H5B	109.4	C6-C5-H5B	109.4

H5A-C5-H5B	108.0	C17-N5-C15	109.5(4)
C17-N5-C13	109.0(4)	C15-N5-C13	108.2(4)
O1_1-C1_1-C2_1	109.8(3)	O1_1-C1_1-C4_1	111.8(3)
C2_1-C1_1-C4_1	108.7(3)	O1_1-C1_1-C3_1	109.7(3)
C2_1-C1_1-C3_1	108.5(3)	C4_1-C1_1-C3_1	108.2(2)
F3_1-C2_1-F1_1	106.5(3)	F3_1-C2_1-F2_1	107.3(3)
F1_1-C2_1-F2_1	107.4(3)	F3_1-C2_1-C1_1	110.4(3)
F1_1-C2_1-C1_1	110.8(3)	F2_1-C2_1-C1_1	114.0(3)
F6_1-C3_1-F5_1	108.0(3)	F6_1-C3_1-F4_1	107.4(3)
F5_1-C3_1-F4_1	106.3(3)	F6_1-C3_1-C1_1	114.1(3)
F5_1-C3_1-C1_1	110.6(3)	F4_1-C3_1-C1_1	110.1(3)
F9_1-C4_1-F8_1	107.9(3)	F9_1-C4_1-F7_1	107.1(3)
F8_1-C4_1-F7_1	107.3(3)	F9_1-C4_1-C1_1	110.7(3)
F8_1-C4_1-C1_1	113.2(3)	F7_1-C4_1-C1_1	110.5(3)
C1_2-O1_2-H1_2	117.(4)	O1_2-C1_2-C2_2	111.2(3)
O1_2-C1_2-C3_2	105.9(3)	C2_2-C1_2-C3_2	109.5(3)
O1_2-C1_2-C4_2	111.2(3)	C2_2-C1_2-C4_2	109.9(3)
C3_2-C1_2-C4_2	109.0(3)	F1_2-C2_2-F2_2	108.7(3)
F1_2-C2_2-F3_2	107.4(3)	F2_2-C2_2-F3_2	108.3(3)
F1_2-C2_2-C1_2	109.8(3)	F2_2-C2_2-C1_2	112.0(3)
F3_2-C2_2-C1_2	110.5(3)	F6_2-C3_2-F5_2	108.2(3)
F6_2-C3_2-F4_2	107.9(3)	F5_2-C3_2-F4_2	107.2(3)
F6_2-C3_2-C1_2	113.1(3)	F5_2-C3_2-C1_2	110.7(3)
F4_2-C3_2-C1_2	109.5(3)	F7_2-C4_2-F9_2	107.8(3)
F7_2-C4_2-F8_2	107.6(3)	F9_2-C4_2-F8_2	108.0(3)
F7_2-C4_2-C1_2	110.8(3)	F9_2-C4_2-C1_2	110.1(3)
F8_2-C4_2-C1_2	112.3(3)	C1_3-O1_3-H1_3	115.(4)
O1_3-C1_3-C3_3	105.5(2)	O1_3-C1_3-C2_3	111.2(3)
C3_3-C1_3-C2_3	109.8(3)	O1_3-C1_3-C4_3	111.3(3)
C3_3-C1_3-C4_3	109.2(3)	C2_3-C1_3-C4_3	109.7(3)
F2_3-C2_3-F1_3	107.6(3)	F2_3-C2_3-F3_3	108.9(3)
F1_3-C2_3-F3_3	107.9(3)	F2_3-C2_3-C1_3	112.7(3)
F1_3-C2_3-C1_3	109.9(3)	F3_3-C2_3-C1_3	109.6(3)
F5_3-C3_3-F6_3	107.0(3)	F5_3-C3_3-F4_3	108.3(3)
F6_3-C3_3-F4_3	108.6(3)	F5_3-C3_3-C1_3	110.8(3)
F6_3-C3_3-C1_3	112.1(3)	F4_3-C3_3-C1_3	109.9(3)
F7_3-C4_3-F9_3	108.2(3)	F7_3-C4_3-F8_3	107.9(3)
F9_3-C4_3-F8_3	107.6(3)	F7_3-C4_3-C1_3	110.7(3)
F9_3-C4_3-C1_3	110.4(3)	F8_3-C4_3-C1_3	112.0(3)
C1_4-O1_4-H1_4	106.(4)	O1_4-C1_4-C4_4	111.7(3)
O1_4-C1_4-C3_4	106.2(3)	C4_4-C1_4-C3_4	109.8(3)
O1_4-C1_4-C2_4	109.5(3)	C4_4-C1_4-C2_4	110.9(3)
C3_4-C1_4-C2_4	108.6(3)	F1_4-C2_4-F3_4	108.4(4)
F1_4-C2_4-F2_4	107.0(3)	F3_4-C2_4-F2_4	108.8(4)
F1_4-C2_4-C1_4	109.9(3)	F3_4-C2_4-C1_4	110.8(3)
F2_4-C2_4-C1_4	111.8(4)	F5_4-C3_4-F6_4	108.6(3)
F5_4-C3_4-F4_4	107.3(3)	F6_4-C3_4-F4_4	107.6(3)
F5_4-C3_4-C1_4	111.4(3)	F6_4-C3_4-C1_4	111.3(3)
F4_4-C3_4-C1_4	110.5(3)	F8_4-C4_4-F7_4	107.7(4)
F8_4-C4_4-F9_4	108.3(4)	F7_4-C4_4-F9_4	107.1(4)
F8_4-C4_4-C1_4	113.2(3)	F7_4-C4_4-C1_4	110.7(3)
F9_4-C4_4-C1_4	109.6(4)	O1_5-C1_5-C4_5	111.8(2)
O1_5-C1_5-C2_5	109.8(3)	C4_5-C1_5-C2_5	108.4(2)
O1_5-C1_5-C3_5	109.5(2)	C4_5-C1_5-C3_5	108.5(3)
C2_5-C1_5-C3_5	108.8(2)	F2_5-C2_5-F3_5	107.4(3)
F2_5-C2_5-F1_5	107.2(3)	F3_5-C2_5-F1_5	106.7(3)
F2_5-C2_5-C1_5	113.8(3)	F3_5-C2_5-C1_5	110.9(3)
F1_5-C2_5-C1_5	110.4(3)	F6_5-C3_5-F5_5	107.3(3)
F6_5-C3_5-F4_5	107.3(3)	F5_5-C3_5-F4_5	106.5(3)
F6_5-C3_5-C1_5	114.2(3)	F5_5-C3_5-C1_5	110.8(2)
F4_5-C3_5-C1_5	110.3(2)	F7_5-C4_5-F9_5	107.3(3)
F7_5-C4_5-F8_5	107.1(3)	F9_5-C4_5-F8_5	107.8(3)
F7_5-C4_5-C1_5	110.7(3)	F9_5-C4_5-C1_5	110.6(3)
F8_5-C4_5-C1_5	113.2(3)	C1_6-O1_6-H1_6	119.(4)
O1_6-C1_6-C2_6	111.5(3)	O1_6-C1_6-C3_6	110.9(3)
C2_6-C1_6-C3_6	109.9(3)	O1_6-C1_6-C4_6	105.7(3)
C2_6-C1_6-C4_6	109.9(3)	C3_6-C1_6-C4_6	109.0(3)
F1_6-C2_6-F2_6	108.1(4)	F1_6-C2_6-F3_6	107.2(4)
F2_6-C2_6-F3_6	107.6(4)	F1_6-C2_6-C1_6	111.4(3)
F2_6-C2_6-C1_6	112.1(3)	F3_6-C2_6-C1_6	110.3(3)
F5_6-C3_6-F6_6	108.4(3)	F5_6-C3_6-F4_6	107.1(4)
F6_6-C3_6-F4_6	107.7(3)	F5_6-C3_6-C1_6	110.4(3)
F6_6-C3_6-C1_6	112.6(3)	F4_6-C3_6-C1_6	110.5(3)

F7_6-C4_6-F8_6	108.2(3)	F7_6-C4_6-F9_6	107.1(3)
F8_6-C4_6-F9_6	108.4(3)	F7_6-C4_6-C1_6	110.5(3)
F8_6-C4_6-C1_6	112.1(3)	F9_6-C4_6-C1_6	110.4(3)
C1_7-O1_7-H1_7	112.(3)	O1_7-C1_7-C2_7	111.1(3)
O1_7-C1_7-C3_7	105.7(2)	C2_7-C1_7-C3_7	109.8(3)
O1_7-C1_7-C4_7	110.9(3)	C2_7-C1_7-C4_7	109.7(3)
C3_7-C1_7-C4_7	109.5(3)	F1_7-C2_7-F2_7	108.0(3)
F1_7-C2_7-F3_7	107.0(3)	F2_7-C2_7-F3_7	108.1(3)
F1_7-C2_7-C1_7	110.5(3)	F2_7-C2_7-C1_7	112.6(3)
F3_7-C2_7-C1_7	110.4(3)	F6_7-C3_7-F5_7	108.0(3)
F6_7-C3_7-F4_7	108.1(3)	F5_7-C3_7-F4_7	108.0(3)
F6_7-C3_7-C1_7	112.4(3)	F5_7-C3_7-C1_7	110.6(3)
F4_7-C3_7-C1_7	109.6(3)	F7_7-C4_7-F9_7	108.2(3)
F7_7-C4_7-F8_7	108.0(3)	F9_7-C4_7-F8_7	108.1(3)
F7_7-C4_7-C1_7	110.8(3)	F9_7-C4_7-C1_7	110.3(3)
F8_7-C4_7-C1_7	111.4(3)	C1_8-O1_8-H1_8	118.(3)
O1_8-C1_8-C3_8	110.7(3)	O1_8-C1_8-C2_8	110.4(3)
C3_8-C1_8-C2_8	109.9(3)	O1_8-C1_8-C4_8	106.7(3)
C3_8-C1_8-C4_8	109.5(3)	C2_8-C1_8-C4_8	109.5(3)
F3_8-C2_8-F2_8	107.1(3)	F3_8-C2_8-F1_8	107.2(3)
F2_8-C2_8-F1_8	106.6(3)	F3_8-C2_8-C1_8	110.8(3)
F2_8-C2_8-C1_8	113.7(3)	F1_8-C2_8-C1_8	111.1(3)
F6_8-C3_8-F4_8	109.2(4)	F6_8-C3_8-F5_8	108.1(4)
F4_8-C3_8-F5_8	107.1(4)	F6_8-C3_8-C1_8	111.9(4)
F4_8-C3_8-C1_8	110.3(4)	F5_8-C3_8-C1_8	110.2(4)
F7_8-C4_8-F9_8	107.4(3)	F7_8-C4_8-F8_8	108.0(3)
F9_8-C4_8-F8_8	107.0(3)	F7_8-C4_8-C1_8	111.6(3)
F9_8-C4_8-C1_8	111.0(3)	F8_8-C4_8-C1_8	111.6(3)
O1_9-C1_9-C4_9	109.8(6)	O1_9-C1_9-C3_9	110.0(6)
C4_9-C1_9-C3_9	108.9(4)	O1_9-C1_9-C2_9	112.1(5)
C4_9-C1_9-C2_9	108.1(4)	C3_9-C1_9-C2_9	108.0(4)
F1_9-C2_9-F2_9	108.1(7)	F1_9-C2_9-F3_9	107.5(6)
F2_9-C2_9-F3_9	106.4(5)	F1_9-C2_9-C1_9	110.8(6)
F2_9-C2_9-C1_9	113.7(6)	F3_9-C2_9-C1_9	109.9(4)
F5_9-C3_9-F6_9	107.3(6)	F5_9-C3_9-F4_9	106.5(6)
F6_9-C3_9-F4_9	107.6(4)	F5_9-C3_9-C1_9	110.8(6)
F6_9-C3_9-C1_9	113.8(4)	F4_9-C3_9-C1_9	110.5(4)
F7_9-C4_9-F9_9	107.2(6)	F7_9-C4_9-F8_9	107.7(6)
F9_9-C4_9-F8_9	106.9(5)	F7_9-C4_9-C1_9	110.5(6)
F9_9-C4_9-C1_9	110.8(4)	F8_9-C4_9-C1_9	113.5(4)
C1_10-O1_10-H1_10	114.(3)	O1_10-C1_10-C2B_10	111.8(5)
O1_10-C1_10-C4A_10	113.7(5)	O1_10-C1_10-C3A_10	106.1(4)
O1_10-C1_10-C3B_10	106.2(5)	O1_10-C1_10-C4B_10	110.1(4)
O1_10-C1_10-C2A_10	108.6(4)	F1A_10-C2A_10-F3A_10	105.6(9)
F1A_10-C2A_10-F2A_10	107.4(10)	F3A_10-C2A_10-F2A_10	107.3(10)
F1A_10-C2A_10-C1_10	109.3(8)	F3A_10-C2A_10-C1_10	112.2(8)
F2A_10-C2A_10-C1_10	114.5(9)	F1B_10-C2B_10-F3B_10	106.6(9)
F1B_10-C2B_10-F2B_10	109.1(10)	F3B_10-C2B_10-F2B_10	107.7(9)
F1B_10-C2B_10-C1_10	111.6(9)	F3B_10-C2B_10-C1_10	109.2(8)
F2B_10-C2B_10-C1_10	112.4(9)	F5A_10-C3A_10-F4A_10	107.0(7)
F5A_10-C3A_10-F6A_10	106.6(10)	F4A_10-C3A_10-F6A_10	108.4(10)
F5A_10-C3A_10-C1_10	110.5(8)	F4A_10-C3A_10-C1_10	112.0(8)
F6A_10-C3A_10-C1_10	112.2(12)	F5B_10-C3B_10-F4B_10	107.4(9)
F5B_10-C3B_10-F6B_10	110.5(12)	F4B_10-C3B_10-F6B_10	107.2(13)
F5B_10-C3B_10-C1_10	113.2(10)	F4B_10-C3B_10-C1_10	109.5(10)
F6B_10-C3B_10-C1_10	108.9(14)	F8A_10-C4A_10-F9A_10	110.8(12)
F8A_10-C4A_10-F7A_10	108.8(11)	F9A_10-C4A_10-F7A_10	107.7(10)
F8A_10-C4A_10-C1_10	113.1(11)	F9A_10-C4A_10-C1_10	108.8(10)
F7A_10-C4A_10-C1_10	107.5(10)	F8B_10-C4B_10-F7B_10	108.6(10)
F8B_10-C4B_10-F9B_10	107.9(10)	F7B_10-C4B_10-F9B_10	106.0(9)
F8B_10-C4B_10-C1_10	113.0(10)	F7B_10-C4B_10-C1_10	111.5(9)
F9B_10-C4B_10-C1_10	109.6(8)	O1_11-C1_11-C2_11	110.4(3)
O1_11-C1_11-C4_11	110.2(3)	C2_11-C1_11-C4_11	108.2(3)
O1_11-C1_11-C3_11	111.9(2)	C2_11-C1_11-C3_11	107.9(3)
C4_11-C1_11-C3_11	108.2(3)	F2_11-C2_11-F1_11	108.0(3)
F2_11-C2_11-F3_11	106.9(3)	F1_11-C2_11-F3_11	106.9(3)
F2_11-C2_11-C1_11	113.9(3)	F1_11-C2_11-C1_11	110.5(3)
F3_11-C2_11-C1_11	110.5(3)	F6_11-C3_11-F4_11	107.7(3)
F6_11-C3_11-F5_11	107.7(3)	F4_11-C3_11-F5_11	107.4(3)
F6_11-C3_11-C1_11	112.9(3)	F4_11-C3_11-C1_11	110.1(3)
F5_11-C3_11-C1_11	110.9(3)	F9_11-C4_11-F7_11	107.6(3)
F9_11-C4_11-F8_11	106.5(3)	F7_11-C4_11-F8_11	107.8(3)
F9_11-C4_11-C1_11	110.5(3)	F7_11-C4_11-C1_11	110.3(3)

F8_11-C4_11-C1_11	113.8(3)	C1_12-O1_12-H1_12	113.(6)
O1_12-C1_12-C4_12	112.0(4)	O1_12-C1_12-C2_12	110.0(4)
C4_12-C1_12-C2_12	109.7(4)	O1_12-C1_12-C3_12	106.2(4)
C4_12-C1_12-C3_12	110.3(4)	C2_12-C1_12-C3_12	108.5(4)
F2_12-C2_12-F1_12	106.5(4)	F2_12-C2_12-F3_12	108.5(4)
F1_12-C2_12-F3_12	107.1(5)	F2_12-C2_12-C1_12	113.5(4)
F1_12-C2_12-C1_12	110.1(4)	F3_12-C2_12-C1_12	110.8(4)
F6_12-C3_12-F5_12	108.5(5)	F6_12-C3_12-F4_12	107.5(5)
F5_12-C3_12-F4_12	106.3(5)	F6_12-C3_12-C1_12	113.1(5)
F5_12-C3_12-C1_12	111.2(4)	F4_12-C3_12-C1_12	109.9(4)
F7_12-C4_12-F8_12	108.3(5)	F7_12-C4_12-F9_12	106.9(5)
F8_12-C4_12-F9_12	107.3(5)	F7_12-C4_12-C1_12	111.7(4)
F8_12-C4_12-C1_12	112.4(4)	F9_12-C4_12-C1_12	110.0(4)
O1_13-C1_13-C2_13	111.6(12)	O1_13-C1_13-C4_13	110.5(12)
C2_13-C1_13-C4_13	108.5(7)	O1_13-C1_13-C3_13	109.0(12)
C2_13-C1_13-C3_13	108.7(7)	C4_13-C1_13-C3_13	108.5(7)
F2_13-C2_13-F1_13	107.4(9)	F2_13-C2_13-F3_13	107.7(12)
F1_13-C2_13-F3_13	108.1(11)	F2_13-C2_13-C1_13	113.1(8)
F1_13-C2_13-C1_13	109.7(7)	F3_13-C2_13-C1_13	110.7(11)
F5_13-C3_13-F6_13	108.0(10)	F5_13-C3_13-F4_13	107.4(11)
F6_13-C3_13-F4_13	108.8(13)	F5_13-C3_13-C1_13	110.3(8)
F6_13-C3_13-C1_13	112.4(11)	F4_13-C3_13-C1_13	109.8(12)
F7_13-C4_13-F9_13	107.4(10)	F7_13-C4_13-F8_13	108.2(9)
F9_13-C4_13-F8_13	108.1(11)	F7_13-C4_13-C1_13	111.0(7)
F9_13-C4_13-C1_13	110.5(10)	F8_13-C4_13-C1_13	111.6(8)
C1_14-O1_14-H1_14	118.(10)	O1_14-C1_14-C3_14	108.7(10)
O1_14-C1_14-C2_14	111.7(10)	C3_14-C1_14-C2_14	109.3(8)
O1_14-C1_14-C4_14	109.3(11)	C3_14-C1_14-C4_14	109.7(8)
C2_14-C1_14-C4_14	108.2(8)	F3_14-C2_14-F1_14	110.1(12)
F3_14-C2_14-F2_14	106.9(11)	F1_14-C2_14-F2_14	103.8(11)
F3_14-C2_14-C1_14	111.5(9)	F1_14-C2_14-C1_14	111.7(9)
F2_14-C2_14-C1_14	112.4(10)	F6_14-C3_14-F5_14	106.0(10)
F6_14-C3_14-F4_14	111.1(13)	F5_14-C3_14-F4_14	104.3(12)
F6_14-C3_14-C1_14	113.5(10)	F5_14-C3_14-C1_14	111.0(9)
F4_14-C3_14-C1_14	110.5(11)	F7_14-C4_14-F9_14	109.5(12)
F7_14-C4_14-F8_14	108.0(13)	F9_14-C4_14-F8_14	107.3(13)
F7_14-C4_14-C1_14	110.8(9)	F9_14-C4_14-C1_14	109.9(12)
F8_14-C4_14-C1_14	111.3(13)		

Table 6. Torsion angles (°) for ArnAn8.

C3-N1-C1-C2	-56.5(5)	C5-N1-C1-C2	61.1(5)
C4-N2-C2-C1	61.2(4)	C6-N2-C2-C1	-58.0(4)
N1-C1-C2-N2	-4.0(5)	C1-N1-C3-C4	61.5(4)
C5-N1-C3-C4	-56.7(4)	C9-N3-C7-C8	-57.0(4)
C11-N3-C7-C8	63.3(4)	C4-N2-C6-C5	-56.7(4)
C2-N2-C6-C5	63.5(4)	N3-C7-C8-N4	-5.4(4)
C11-N3-C9-C10	-57.2(4)	C7-N3-C9-C10	63.0(4)
C9-N3-C11-C12	64.4(4)	C7-N3-C11-C12	-55.9(4)
N4-C12-C11-N3	-7.0(5)	N3-C9-C10-N4	-5.4(4)
C18-N6-C16-C15	-59.2(5)	C14-N6-C16-C15	60.4(5)
N6-C16-C15-N5	-0.3(6)	C18-N6-C14-C13	59.9(5)
C16-N6-C14-C13	-60.3(5)	N6-C14-C13-N5	-0.1(6)
C2-N2-C4-C3	-56.4(4)	C6-N2-C4-C3	62.9(4)
N1-C3-C4-N2	-4.8(5)	C7-C8-N4-C12	-56.3(4)
C7-C8-N4-C10	62.0(4)	C11-C12-N4-C8	63.9(4)
C11-C12-N4-C10	-54.2(4)	C9-C10-N4-C8	-55.8(4)
C9-C10-N4-C12	61.6(4)	C23-N7-C21-C22	-63.5(4)
C19-N7-C21-C22	56.9(4)	C21-N7-C23-C24	55.8(4)
C19-N7-C23-C24	-62.8(4)	C22-N8-C24-C23	-62.8(4)
C20-N8-C24-C23	55.2(4)	N7-C23-C24-N8	6.5(5)
C24-N8-C22-C21	55.1(4)	C20-N8-C22-C21	-63.0(4)
N7-C21-C22-N8	6.5(5)	C22-N8-C20-C19	54.1(4)
C24-N8-C20-C19	-64.1(4)	C21-N7-C19-C20	-65.5(4)
C23-N7-C19-C20	54.1(5)	N8-C20-C19-N7	8.8(5)
C16-N6-C18-C17	59.1(5)	C14-N6-C18-C17	-59.9(5)
N5-C17-C18-N6	0.3(7)	C3-N1-C5-C6	63.1(4)
C1-N1-C5-C6	-55.5(4)	N2-C6-C5-N1	-5.5(5)
C18-C17-N5-C15	-59.1(6)	C18-C17-N5-C13	59.1(6)
C16-C15-N5-C17	58.9(5)	C16-C15-N5-C13	-59.8(5)
C14-C13-N5-C17	-59.0(5)	C14-C13-N5-C15	60.0(5)

O1_1-C1_1-C2_1-F3_1	41.2(4)	C4_1-C1_1-C2_1-F3_1	163.8(3)
C3_1-C1_1-C2_1-F3_1	-78.7(3)	O1_1-C1_1-C2_1-F1_1	-76.6(3)
C4_1-C1_1-C2_1-F1_1	46.0(4)	C3_1-C1_1-C2_1-F1_1	163.5(3)
O1_1-C1_1-C2_1-F2_1	162.1(3)	C4_1-C1_1-C2_1-F2_1	-75.3(3)
C3_1-C1_1-C2_1-F2_1	42.2(4)	O1_1-C1_1-C3_1-F6_1	164.2(3)
C2_1-C1_1-C3_1-F6_1	-75.8(4)	C4_1-C1_1-C3_1-F6_1	42.0(4)
O1_1-C1_1-C3_1-F5_1	-73.8(3)	C2_1-C1_1-C3_1-F5_1	46.2(4)
C4_1-C1_1-C3_1-F5_1	164.0(3)	O1_1-C1_1-C3_1-F4_1	43.4(3)
C2_1-C1_1-C3_1-F4_1	163.4(3)	C4_1-C1_1-C3_1-F4_1	-78.8(3)
O1_1-C1_1-C4_1-F9_1	43.8(4)	C2_1-C1_1-C4_1-F9_1	-77.5(3)
C3_1-C1_1-C4_1-F9_1	164.8(3)	O1_1-C1_1-C4_1-F8_1	165.1(3)
C2_1-C1_1-C4_1-F8_1	43.7(4)	C3_1-C1_1-C4_1-F8_1	-74.0(4)
O1_1-C1_1-C4_1-F7_1	-74.6(3)	C2_1-C1_1-C4_1-F7_1	164.0(3)
C3_1-C1_1-C4_1-F7_1	46.3(3)	O1_2-C1_2-C2_2-F1_2	47.1(4)
C3_2-C1_2-C2_2-F1_2	163.8(3)	C4_2-C1_2-C2_2-F1_2	-76.5(4)
O1_2-C1_2-C2_2-F2_2	167.9(3)	C3_2-C1_2-C2_2-F2_2	-75.4(4)
C4_2-C1_2-C2_2-F2_2	44.3(4)	O1_2-C1_2-C2_2-F3_2	-71.3(4)
C3_2-C1_2-C2_2-F3_2	45.4(4)	C4_2-C1_2-C2_2-F3_2	165.1(3)
O1_2-C1_2-C3_2-F6_2	167.8(3)	C2_2-C1_2-C3_2-F6_2	47.8(4)
C4_2-C1_2-C3_2-F6_2	-72.4(4)	O1_2-C1_2-C3_2-F5_2	46.2(4)
C2_2-C1_2-C3_2-F5_2	-73.8(4)	C4_2-C1_2-C3_2-F5_2	165.9(3)
O1_2-C1_2-C3_2-F4_2	-71.8(4)	C2_2-C1_2-C3_2-F4_2	168.2(3)
C4_2-C1_2-C3_2-F4_2	48.0(4)	O1_2-C1_2-C4_2-F7_2	40.7(4)
C2_2-C1_2-C4_2-F7_2	164.2(3)	C3_2-C1_2-C4_2-F7_2	-75.7(4)
O1_2-C1_2-C4_2-F9_2	-78.6(4)	C2_2-C1_2-C4_2-F9_2	45.0(4)
C3_2-C1_2-C4_2-F9_2	165.0(3)	O1_2-C1_2-C4_2-F8_2	161.0(3)
C2_2-C1_2-C4_2-F8_2	-75.4(4)	C3_2-C1_2-C4_2-F8_2	44.6(4)
O1_3-C1_3-C2_3-F2_3	167.7(3)	C3_3-C1_3-C2_3-F2_3	-75.9(4)
C4_3-C1_3-C2_3-F2_3	44.1(4)	O1_3-C1_3-C2_3-F1_3	47.7(4)
C3_3-C1_3-C2_3-F1_3	164.1(3)	C4_3-C1_3-C2_3-F1_3	-75.9(3)
O1_3-C1_3-C2_3-F3_3	-70.8(3)	C3_3-C1_3-C2_3-F3_3	45.6(3)
C4_3-C1_3-C2_3-F3_3	165.6(3)	O1_3-C1_3-C3_3-F5_3	44.5(4)
C2_3-C1_3-C3_3-F5_3	-75.5(3)	C4_3-C1_3-C3_3-F5_3	164.2(3)
O1_3-C1_3-C3_3-F6_3	164.0(3)	C2_3-C1_3-C3_3-F6_3	44.0(4)
C4_3-C1_3-C3_3-F6_3	-76.3(3)	O1_3-C1_3-C3_3-F4_3	-75.1(3)
C2_3-C1_3-C3_3-F4_3	164.9(3)	C4_3-C1_3-C3_3-F4_3	44.6(3)
O1_3-C1_3-C4_3-F7_3	39.5(4)	C3_3-C1_3-C4_3-F7_3	-76.5(3)
C2_3-C1_3-C4_3-F7_3	163.0(3)	O1_3-C1_3-C4_3-F9_3	-80.2(3)
C3_3-C1_3-C4_3-F9_3	163.7(3)	C2_3-C1_3-C4_3-F9_3	43.3(4)
O1_3-C1_3-C4_3-F8_3	159.9(3)	C3_3-C1_3-C4_3-F8_3	43.9(4)
C2_3-C1_3-C4_3-F8_3	-76.5(3)	O1_4-C1_4-C2_4-F1_4	47.2(4)
C4_4-C1_4-C2_4-F1_4	-76.5(4)	C3_4-C1_4-C2_4-F1_4	162.8(3)
O1_4-C1_4-C2_4-F3_4	-72.6(4)	C4_4-C1_4-C2_4-F3_4	163.7(3)
C3_4-C1_4-C2_4-F3_4	43.0(4)	O1_4-C1_4-C2_4-F2_4	165.9(3)
C4_4-C1_4-C2_4-F2_4	42.1(5)	C3_4-C1_4-C2_4-F2_4	-78.6(4)
O1_4-C1_4-C3_4-F5_4	40.8(4)	C4_4-C1_4-C3_4-F5_4	161.8(3)
C2_4-C1_4-C3_4-F5_4	-76.9(4)	O1_4-C1_4-C3_4-F6_4	162.1(3)
C4_4-C1_4-C3_4-F6_4	-76.9(4)	C2_4-C1_4-C3_4-F6_4	44.5(4)
O1_4-C1_4-C3_4-F4_4	-78.3(4)	C4_4-C1_4-C3_4-F4_4	42.6(4)
C2_4-C1_4-C3_4-F4_4	164.0(3)	O1_4-C1_4-C4_4-F8_4	160.8(4)
C3_4-C1_4-C4_4-F8_4	43.3(5)	C2_4-C1_4-C4_4-F8_4	-76.7(5)
O1_4-C1_4-C4_4-F7_4	39.8(4)	C3_4-C1_4-C4_4-F7_4	-77.8(4)
C2_4-C1_4-C4_4-F7_4	162.2(3)	O1_4-C1_4-C4_4-F9_4	-78.2(4)
C3_4-C1_4-C4_4-F9_4	164.3(3)	C2_4-C1_4-C4_4-F9_4	44.3(5)
O1_5-C1_5-C2_5-F2_5	-165.3(3)	C4_5-C1_5-C2_5-F2_5	-42.9(4)
C3_5-C1_5-C2_5-F2_5	74.9(3)	O1_5-C1_5-C2_5-F3_5	73.4(3)
C4_5-C1_5-C2_5-F3_5	-164.2(3)	C3_5-C1_5-C2_5-F3_5	-46.4(3)
O1_5-C1_5-C2_5-F1_5	-44.7(3)	C4_5-C1_5-C2_5-F1_5	77.7(3)
C3_5-C1_5-C2_5-F1_5	-164.5(3)	O1_5-C1_5-C3_5-F6_5	-165.2(3)
C4_5-C1_5-C3_5-F6_5	72.5(3)	C2_5-C1_5-C3_5-F6_5	-45.3(3)
O1_5-C1_5-C3_5-F5_5	-43.9(3)	C4_5-C1_5-C3_5-F5_5	-166.3(2)
C2_5-C1_5-C3_5-F5_5	76.0(3)	O1_5-C1_5-C3_5-F4_5	73.8(3)
C4_5-C1_5-C3_5-F4_5	-48.5(3)	C2_5-C1_5-C3_5-F4_5	-166.2(3)
O1_5-C1_5-C4_5-F7_5	-43.0(4)	C2_5-C1_5-C4_5-F7_5	-164.2(3)
C3_5-C1_5-C4_5-F7_5	77.9(3)	O1_5-C1_5-C4_5-F9_5	75.7(3)
C2_5-C1_5-C4_5-F9_5	-45.5(3)	C3_5-C1_5-C4_5-F9_5	-163.4(3)
O1_5-C1_5-C4_5-F8_5	-163.2(3)	C2_5-C1_5-C4_5-F8_5	75.6(4)
C3_5-C1_5-C4_5-F8_5	-42.3(4)	O1_6-C1_6-C2_6-F1_6	70.9(4)
C3_6-C1_6-C2_6-F1_6	-165.8(3)	C4_6-C1_6-C2_6-F1_6	-45.9(4)
O1_6-C1_6-C2_6-F2_6	-167.9(4)	C3_6-C1_6-C2_6-F2_6	-44.6(5)
C4_6-C1_6-C2_6-F2_6	75.3(5)	O1_6-C1_6-C2_6-F3_6	-48.0(4)
C3_6-C1_6-C2_6-F3_6	75.3(4)	C4_6-C1_6-C2_6-F3_6	-164.8(3)
O1_6-C1_6-C3_6-F5_6	76.6(4)	C2_6-C1_6-C3_6-F5_6	-47.1(4)

C4_6-C1_6-C3_6-F5_6	-167.5(3)	O1_6-C1_6-C3_6-F6_6	-162.1(3)
C2_6-C1_6-C3_6-F6_6	74.3(4)	C4_6-C1_6-C3_6-F6_6	-46.2(4)
O1_6-C1_6-C3_6-F4_6	-41.7(4)	C2_6-C1_6-C3_6-F4_6	-165.3(3)
C4_6-C1_6-C3_6-F4_6	74.2(4)	O1_6-C1_6-C4_6-F7_6	68.6(4)
C2_6-C1_6-C4_6-F7_6	-171.0(3)	C3_6-C1_6-C4_6-F7_6	-50.6(4)
O1_6-C1_6-C4_6-F8_6	-170.7(3)	C2_6-C1_6-C4_6-F8_6	-50.3(4)
C3_6-C1_6-C4_6-F8_6	70.1(4)	O1_6-C1_6-C4_6-F9_6	-49.7(4)
C2_6-C1_6-C4_6-F9_6	70.7(4)	C3_6-C1_6-C4_6-F9_6	-168.9(3)
O1_7-C1_7-C2_7-F1_7	47.5(4)	C3_7-C1_7-C2_7-F1_7	164.0(3)
C4_7-C1_7-C2_7-F1_7	-75.6(3)	O1_7-C1_7-C2_7-F2_7	168.3(3)
C3_7-C1_7-C2_7-F2_7	-75.2(4)	C4_7-C1_7-C2_7-F2_7	45.2(4)
O1_7-C1_7-C2_7-F3_7	-70.8(3)	C3_7-C1_7-C2_7-F3_7	45.7(3)
C4_7-C1_7-C2_7-F3_7	166.1(3)	O1_7-C1_7-C3_7-F6_7	164.9(3)
C2_7-C1_7-C3_7-F6_7	45.0(4)	C4_7-C1_7-C3_7-F6_7	-75.5(3)
O1_7-C1_7-C3_7-F5_7	44.0(4)	C2_7-C1_7-C3_7-F5_7	-75.9(3)
C4_7-C1_7-C3_7-F5_7	163.6(3)	O1_7-C1_7-C3_7-F4_7	-74.9(3)
C2_7-C1_7-C3_7-F4_7	165.2(3)	C4_7-C1_7-C3_7-F4_7	44.7(3)
O1_7-C1_7-C4_7-F7_7	39.0(4)	C2_7-C1_7-C4_7-F7_7	162.1(3)
C3_7-C1_7-C4_7-F7_7	-77.3(3)	O1_7-C1_7-C4_7-F9_7	-80.8(3)
C2_7-C1_7-C4_7-F9_7	42.3(4)	C3_7-C1_7-C4_7-F9_7	162.8(3)
O1_7-C1_7-C4_7-F8_7	159.2(3)	C2_7-C1_7-C4_7-F8_7	-77.7(3)
C3_7-C1_7-C4_7-F8_7	42.9(4)	O1_8-C1_8-C2_8-F3_8	46.0(4)
C3_8-C1_8-C2_8-F3_8	-76.4(4)	C4_8-C1_8-C2_8-F3_8	163.2(3)
O1_8-C1_8-C2_8-F2_8	166.7(3)	C3_8-C1_8-C2_8-F2_8	44.3(4)
C4_8-C1_8-C2_8-F2_8	-76.1(4)	O1_8-C1_8-C2_8-F1_8	-73.1(4)
C3_8-C1_8-C2_8-F1_8	164.5(3)	C4_8-C1_8-C2_8-F1_8	44.2(4)
O1_8-C1_8-C3_8-F6_8	163.5(4)	C2_8-C1_8-C3_8-F6_8	-74.3(5)
C4_8-C1_8-C3_8-F6_8	46.1(5)	O1_8-C1_8-C3_8-F4_8	41.9(5)
C2_8-C1_8-C3_8-F4_8	164.1(3)	C4_8-C1_8-C3_8-F4_8	-75.6(4)
O1_8-C1_8-C3_8-F5_8	-76.2(4)	C2_8-C1_8-C3_8-F5_8	46.0(5)
C4_8-C1_8-C3_8-F5_8	166.4(4)	O1_8-C1_8-C4_8-F7_8	-77.1(4)
C3_8-C1_8-C4_8-F7_8	42.8(4)	C2_8-C1_8-C4_8-F7_8	163.4(3)
O1_8-C1_8-C4_8-F9_8	42.7(4)	C3_8-C1_8-C4_8-F9_8	162.6(3)
C2_8-C1_8-C4_8-F9_8	-76.8(4)	O1_8-C1_8-C4_8-F8_8	162.0(3)
C3_8-C1_8-C4_8-F8_8	-78.1(4)	C2_8-C1_8-C4_8-F8_8	42.5(4)
O1_9-C1_9-C2_9-F1_9	-42.3(9)	C4_9-C1_9-C2_9-F1_9	78.7(7)
C3_9-C1_9-C2_9-F1_9	-163.6(6)	O1_9-C1_9-C2_9-F2_9	-164.4(7)
C4_9-C1_9-C2_9-F2_9	-43.3(7)	C3_9-C1_9-C2_9-F2_9	74.4(7)
O1_9-C1_9-C2_9-F3_9	76.4(7)	C4_9-C1_9-C2_9-F3_9	-162.5(4)
C3_9-C1_9-C2_9-F3_9	-44.9(5)	O1_9-C1_9-C3_9-F5_9	-47.4(8)
C4_9-C1_9-C3_9-F5_9	-167.7(6)	C2_9-C1_9-C3_9-F5_9	75.2(6)
O1_9-C1_9-C3_9-F6_9	-168.4(6)	C4_9-C1_9-C3_9-F6_9	71.3(6)
C2_9-C1_9-C3_9-F6_9	-45.9(6)	O1_9-C1_9-C3_9-F4_9	70.4(7)
C4_9-C1_9-C3_9-F4_9	-50.0(6)	C2_9-C1_9-C3_9-F4_9	-167.1(5)
O1_9-C1_9-C4_9-F7_9	-41.9(8)	C3_9-C1_9-C4_9-F7_9	78.6(7)
C2_9-C1_9-C4_9-F7_9	-164.3(6)	O1_9-C1_9-C4_9-F9_9	76.8(7)
C3_9-C1_9-C4_9-F9_9	-162.7(5)	C2_9-C1_9-C4_9-F9_9	-45.6(6)
O1_9-C1_9-C4_9-F8_9	-162.9(7)	C3_9-C1_9-C4_9-F8_9	-42.4(6)
C2_9-C1_9-C4_9-F8_9	74.6(6)	O1_10-C1_10-C2A_10-F1A_10	-77.6(8)
O1_10-C1_10-C2A_10-F3A_10	39.2(8)	O1_10-C1_10-C2A_10-F2A_10	161.8(7)
O1_10-C1_10-C2B_10-F1B_10	-47.1(9)	O1_10-C1_10-C2B_10-F3B_10	70.4(7)
O1_10-C1_10-C2B_10-F2B_10	-170.1(7)	O1_10-C1_10-C3A_10-F5A_10	-42.5(7)
O1_10-C1_10-C3A_10-F4A_10	76.6(7)	O1_10-C1_10-C3A_10-F6A_10	-161.2(8)
O1_10-C1_10-C3B_10-F5B_10	-70.5(9)	O1_10-C1_10-C3B_10-F4B_10	49.3(9)
O1_10-C1_10-C3B_10-F6B_10	166.2(11)	O1_10-C1_10-C4A_10-F8A_10	168.3(8)
O1_10-C1_10-C4A_10-F9A_10	44.7(9)	O1_10-C1_10-C4A_10-F7A_10	-71.6(9)
O1_10-C1_10-C4B_10-F8B_10	-164.9(7)	O1_10-C1_10-C4B_10-F7B_10	-42.3(8)
O1_10-C1_10-C4B_10-F9B_10	74.7(8)	O1_11-C1_11-C2_11-F2_11	-167.4(3)
C4_11-C1_11-C2_11-F2_11	71.9(4)	C3_11-C1_11-C2_11-F2_11	-44.9(4)
O1_11-C1_11-C2_11-F1_11	70.9(4)	C4_11-C1_11-C2_11-F1_11	-49.8(4)
C3_11-C1_11-C2_11-F1_11	-166.6(3)	O1_11-C1_11-C2_11-F3_11	-47.2(4)
C4_11-C1_11-C2_11-F3_11	-167.8(3)	C3_11-C1_11-C2_11-F3_11	75.4(3)
O1_11-C1_11-C3_11-F6_11	-162.7(3)	C2_11-C1_11-C3_11-F6_11	75.7(3)
C4_11-C1_11-C3_11-F6_11	-41.1(4)	O1_11-C1_11-C3_11-F4_11	-42.3(4)

C2_11-C1_11-C3_11-F4_11	-163.9(3)	C4_11-C1_11-C3_11-F4_11	79.2(3)
O1_11-C1_11-C3_11-F5_11	76.3(3)	C2_11-C1_11-C3_11-F5_11	-45.2(3)
C4_11-C1_11-C3_11-F5_11	-162.1(3)	O1_11-C1_11-C4_11-F9_11	-42.1(4)
C2_11-C1_11-C4_11-F9_11	78.6(3)	C3_11-C1_11-C4_11-F9_11	-164.8(3)
O1_11-C1_11-C4_11-F7_11	76.7(3)	C2_11-C1_11-C4_11-F7_11	-162.5(3)
C3_11-C1_11-C4_11-F7_11	-45.9(4)	O1_11-C1_11-C4_11-F8_11	-161.9(3)
C2_11-C1_11-C4_11-F8_11	-41.2(4)	C3_11-C1_11-C4_11-F8_11	75.4(4)
O1_12-C1_12-C2_12-F2_12	-163.8(5)	C4_12-C1_12-C2_12-F2_12	-40.2(6)
C3_12-C1_12-C2_12-F2_12	80.4(5)	O1_12-C1_12-C2_12-F1_12	-44.4(6)
C4_12-C1_12-C2_12-F1_12	79.1(5)	C3_12-C1_12-C2_12-F1_12	-160.3(4)
O1_12-C1_12-C2_12-F3_12	73.9(5)	C4_12-C1_12-C2_12-F3_12	-162.5(4)
C3_12-C1_12-C2_12-F3_12	-42.0(5)	O1_12-C1_12-C3_12-F6_12	-163.9(5)
C4_12-C1_12-C3_12-F6_12	74.6(6)	C2_12-C1_12-C3_12-F6_12	-45.6(6)
O1_12-C1_12-C3_12-F5_12	-41.5(6)	C4_12-C1_12-C3_12-F5_12	-163.0(5)
C2_12-C1_12-C3_12-F5_12	76.8(5)	O1_12-C1_12-C3_12-F4_12	75.9(5)
C4_12-C1_12-C3_12-F4_12	-45.6(5)	C2_12-C1_12-C3_12-F4_12	-165.8(4)
O1_12-C1_12-C4_12-F7_12	-40.4(6)	C2_12-C1_12-C4_12-F7_12	-162.8(4)
C3_12-C1_12-C4_12-F7_12	77.7(5)	O1_12-C1_12-C4_12-F8_12	-162.3(5)
C2_12-C1_12-C4_12-F8_12	75.3(6)	C3_12-C1_12-C4_12-F8_12	-44.2(6)
O1_12-C1_12-C4_12-F9_12	78.2(5)	C2_12-C1_12-C4_12-F9_12	-44.2(5)
C3_12-C1_12-C4_12-F9_12	-163.7(4)	O1_13-C1_13-C2_13-F2_13	163.3(14)
C4_13-C1_13-C2_13-F2_13	-74.8(10)	C3_13-C1_13-C2_13-F2_13	43.1(11)
O1_13-C1_13-C2_13-F1_13	-76.8(14)	C4_13-C1_13-C2_13-F1_13	45.1(10)
C3_13-C1_13-C2_13-F1_13	163.0(8)	O1_13-C1_13-C2_13-F3_13	42.4(17)
C4_13-C1_13-C2_13-F3_13	164.3(12)	C3_13-C1_13-C2_13-F3_13	-77.8(13)
O1_13-C1_13-C3_13-F5_13	-77.1(14)	C2_13-C1_13-C3_13-F5_13	44.7(10)
C4_13-C1_13-C3_13-F5_13	162.5(9)	O1_13-C1_13-C3_13-F6_13	162.4(15)
C2_13-C1_13-C3_13-F6_13	-75.8(13)	C4_13-C1_13-C3_13-F6_13	42.0(13)
O1_13-C1_13-C3_13-F4_13	41.0(17)	C2_13-C1_13-C3_13-F4_13	162.8(13)
C4_13-C1_13-C3_13-F4_13	-79.3(13)	O1_13-C1_13-C4_13-F7_13	-74.0(14)
C2_13-C1_13-C4_13-F7_13	163.4(9)	C3_13-C1_13-C4_13-F7_13	45.5(10)
O1_13-C1_13-C4_13-F9_13	45.1(16)	C2_13-C1_13-C4_13-F9_13	-77.5(12)
C3_13-C1_13-C4_13-F9_13	164.5(11)	O1_13-C1_13-C4_13-F8_13	165.3(14)
C2_13-C1_13-C4_13-F8_13	42.7(11)	C3_13-C1_13-C4_13-F8_13	-75.3(10)
O1_14-C1_14-C2_14-F3_14	51.2(16)	C3_14-C1_14-C2_14-F3_14	-69.0(14)
C4_14-C1_14-C2_14-F3_14	171.6(12)	O1_14-C1_14-C2_14-F1_14	-72.5(15)
C3_14-C1_14-C2_14-F1_14	167.3(12)	C4_14-C1_14-C2_14-F1_14	47.9(13)
O1_14-C1_14-C2_14-F2_14	171.3(14)	C3_14-C1_14-C2_14-F2_14	51.0(14)
C4_14-C1_14-C2_14-F2_14	-68.4(13)	O1_14-C1_14-C3_14-F6_14	175.7(15)
C2_14-C1_14-C3_14-F6_14	-62.2(15)	C4_14-C1_14-C3_14-F6_14	56.2(14)
O1_14-C1_14-C3_14-F5_14	-65.0(15)	C2_14-C1_14-C3_14-F5_14	57.1(13)
C4_14-C1_14-C3_14-F5_14	175.5(11)	O1_14-C1_14-C3_14-F4_14	50.2(16)
C2_14-C1_14-C3_14-F4_14	172.3(13)	C4_14-C1_14-C3_14-F4_14	-69.3(14)
O1_14-C1_14-C4_14-F7_14	-64.7(13)	C3_14-C1_14-C4_14-F7_14	54.4(12)
C2_14-C1_14-C4_14-F7_14	173.5(10)	O1_14-C1_14-C4_14-F9_14	56.4(16)
C3_14-C1_14-C4_14-F9_14	175.5(14)	C2_14-C1_14-C4_14-F9_14	-65.4(14)
O1_14-C1_14-C4_14-F8_14	175.1(16)	C3_14-C1_14-C4_14-F8_14	-65.8(16)
C2_14-C1_14-C4_14-F8_14	53.3(16)		
