# Supplementary material for: Enzyme-like polyene cyclizations catalyzed by dynamic, self-assembled, supramolecular fluoro alcohol-amine clusters
Source: Nat Commun. 2023 Feb 13;14:813. doi: 10.1038/s41467-023-36157-0 (PMC9925744; doi:10.1038/s41467-023-36157-0)
Supplement: Supplementary file 5 — Supplementary Data 3 [file 41467_2023_36157_MOESM5_ESM.pdf]

Enzyme-Like Polyene Cyclizations Catalyzed by Dynamic, Self-Assembled, Supramolecular Fluoro Alcohol-Amine Clusters

Andreas M. Arnold,[1,2] Philipp Dullinger,[3] Aniruddha Biswas,[2] Christian Jandl,[4] Dominik Horinek[3] and Tanja Gulder[1,2,4*]

[1]*Biomimetic Catalysis, Department of Chemistry, Technical University Munich, Lichtenbergstraße 4, 85747 Garching, Germany*
[2]*Chair of Organic Chemistry, Faculty of Chemistry and Mineralogy, Leipzig University, Johannisallee 29, 04103 Leipzig, Germany*[3]*Institute of Physical and Theoretical Chemistry, University of Regensburg, 93040 Regensburg, Germany*
[4]*Catalysis Research Center, Technical University Munich, Ernst-Otto-Fischer-Straße 1, 85747 Garching, Germany*

SUPPLEMENTARY DATA 3: SIMULATION PARAMETERS

Forcefield parameters:

General information:

```
[ defaults ]
; nbfunc       comb-rule      gen-pairs       fudgeLJ fudgeQQ
1              2              yes             0.5     0.8333
```

Atom types: GAFF parameters

```
GerBn.itp
[ molecule type ]
;name           nrexcl
 HGB            3

[ atoms ]
    1   ca    1   HGB     C    1    -0.186730     12.01000 ; qtot 0.000
    2   ca    1   HGB    C1    2     0.183336     12.01000 ; qtot 0.000
    3   ca    1   HGB    C2    3    -0.196350     12.01000 ; qtot 0.000
    4   ca    1   HGB    C3    4    -0.104815     12.01000 ; qtot 0.000
    5   ca    1   HGB    C4    5    -0.150817     12.01000 ; qtot 0.000
    6   ca    1   HGB    C5    6    -0.114855     12.01000 ; qtot 0.000
    7   c3    1   HGB    C6    7    -0.446041     12.01000 ; qtot 0.000
    8   c3    1   HGB    C7    8     0.558880     12.01000 ; qtot 0.000
    9   c2    1   HGB    C8    9    -0.576308     12.01000 ; qtot 0.000
   10   c2    1   HGB    C9   10     0.242090     12.01000 ; qtot 0.000
   11   c3    1   HGB   C10   11    -0.366397     12.01000 ; qtot 0.000
   12   c3    1   HGB   C11   12     0.434900     12.01000 ; qtot 0.000
   13   c2    1   HGB   C12   13    -0.611442     12.01000 ; qtot 0.000
   14   c2    1   HGB   C13   14     0.291109     12.01000 ; qtot 0.000
   15   c3    1   HGB   C14   15    -0.336390     12.01000 ; qtot 0.000
   16   c3    1   HGB   C15   16    -0.246001     12.01000 ; qtot 0.000
   17   c3    1   HGB   C16   17    -0.269185     12.01000 ; qtot 0.000
   18   ha    1   HGB     H   18     0.098879      1.00800 ; qtot 0.000
   19   ha    1   HGB    H1   19     0.124961      1.00800 ; qtot 0.000
   20   ha    1   HGB    H2   20     0.117700      1.00800 ; qtot 0.000
   21   ha    1   HGB    H3   21     0.121519      1.00800 ; qtot 0.000
   22   ha    1   HGB    H4   22     0.122887      1.00800 ; qtot 0.000
   23   hc    1   HGB    H5   23     0.122172      1.00800 ; qtot 0.000
   24   hc    1   HGB    H6   24     0.122172      1.00800 ; qtot 0.000
   25   hc    1   HGB    H7   25    -0.056683      1.00800 ; qtot 0.000
   26   hc    1   HGB    H8   26    -0.056683      1.00800 ; qtot 0.000
   27   ha    1   HGB    H9   27     0.132368      1.00800 ; qtot 0.000
   28   hc    1   HGB   H10   28     0.105259      1.00800 ; qtot 0.000
   29   hc    1   HGB   H11   29     0.105259      1.00800 ; qtot 0.000
   30   hc    1   HGB   H12   30    -0.022929      1.00800 ; qtot 0.000
   31   hc    1   HGB   H13   31    -0.022929      1.00800 ; qtot 0.000
   32   ha    1   HGB   H14   32     0.183628      1.00800 ; qtot 0.000
   33   hc    1   HGB   H15   33     0.091792      1.00800 ; qtot 0.000
   34   hc    1   HGB   H16   34     0.091792      1.00800 ; qtot 0.000
   35   hc    1   HGB   H17   35     0.091792      1.00800 ; qtot 0.000
```

```
   36   hc     1   HGB   H18   36     0.067073      1.00800 ; qtot 0.000
   37   hc     1   HGB   H19   37     0.067073      1.00800 ; qtot 0.000
   38   hc     1   HGB   H20   38     0.067073      1.00800 ; qtot 0.000
   39   hc     1   HGB   H21   39     0.073614      1.00800 ; qtot 0.000
   40   hc     1   HGB   H22   40     0.073614      1.00800 ; qtot 0.000
   41   hc     1   HGB   H23   41     0.073614      1.00800 ; qtot 0.000

[ bonds ]
;   ai     aj funct   r            k
    1     2   1    1.3984e-01    3.8585e+05 ;     C - C1
    1     6   1    1.3984e-01    3.8585e+05 ;     C - C5
    1    18   1    1.0860e-01    2.8937e+05 ;     C - H
    2     3   1    1.3984e-01    3.8585e+05 ;    C1 - C2
    2     7   1    1.5156e-01    2.6861e+05 ;    C1 - C6
    3     4   1    1.3984e-01    3.8585e+05 ;    C2 - C3
    3    19   1    1.0860e-01    2.8937e+05 ;    C2 - H1
    4     5   1    1.3984e-01    3.8585e+05 ;    C3 - C4
    4    20   1    1.0860e-01    2.8937e+05 ;    C3 - H2
    5     6   1    1.3984e-01    3.8585e+05 ;    C4 - C5
    5    21   1    1.0860e-01    2.8937e+05 ;    C4 - H3
    6    22   1    1.0860e-01    2.8937e+05 ;    C5 - H4
    7     8   1    1.5375e-01    2.5179e+05 ;    C6 - C7
    7    23   1    1.0969e-01    2.7665e+05 ;    C6 - H5
    7    24   1    1.0969e-01    2.7665e+05 ;    C6 - H6
    8     9   1    1.5095e-01    2.7347e+05 ;    C7 - C8
    8    25   1    1.0969e-01    2.7665e+05 ;    C7 - H7
    8    26   1    1.0969e-01    2.7665e+05 ;    C7 - H8
    9    10   1    1.3343e-01    4.7647e+05 ;    C8 - C9
    9    27   1    1.0879e-01    2.8711e+05 ;    C8 - H9
   10    11   1    1.5095e-01    2.7347e+05 ;    C9 - C10
   10    17   1    1.5095e-01    2.7347e+05 ;    C9 - C16
   11    12   1    1.5375e-01    2.5179e+05 ;   C10 - C11
   11    28   1    1.0969e-01    2.7665e+05 ;   C10 - H10
   11    29   1    1.0969e-01    2.7665e+05 ;   C10 - H11
   12    13   1    1.5095e-01    2.7347e+05 ;   C11 - C12
   12    30   1    1.0969e-01    2.7665e+05 ;   C11 - H12
   12    31   1    1.0969e-01    2.7665e+05 ;   C11 - H13
   13    14   1    1.3343e-01    4.7647e+05 ;   C12 - C13
   13    32   1    1.0879e-01    2.8711e+05 ;   C12 - H14
   14    15   1    1.5095e-01    2.7347e+05 ;   C13 - C14
   14    16   1    1.5095e-01    2.7347e+05 ;   C13 - C15
   15    33   1    1.0969e-01    2.7665e+05 ;   C14 - H15
   15    34   1    1.0969e-01    2.7665e+05 ;   C14 - H16
   15    35   1    1.0969e-01    2.7665e+05 ;   C14 - H17
   16    36   1    1.0969e-01    2.7665e+05 ;   C15 - H18
   16    37   1    1.0969e-01    2.7665e+05 ;   C15 - H19
   16    38   1    1.0969e-01    2.7665e+05 ;   C15 - H20
   17    39   1    1.0969e-01    2.7665e+05 ;   C16 - H21
   17    40   1    1.0969e-01    2.7665e+05 ;   C16 - H22
   17    41   1    1.0969e-01    2.7665e+05 ;   C16 - H23
```

```
[ pairs ]
;  ai   aj  funct
   1    4    1 ;    C - C3
   1    8    1 ;    C - C7
   1   19    1 ;    C - H1
   1   21    1 ;    C - H3
   1   23    1 ;    C - H5
   1   24    1 ;    C - H6
   2    5    1 ;   C1 - C4
   2    9    1 ;   C1 - C8
   2   20    1 ;   C1 - H2
   2   22    1 ;   C1 - H4
   2   25    1;    C1 - H7
   2   26    1;    C1 - H8
   3    8    1 ;   C2 - C7
   3   21    1 ;   C2 - H3
   3   23    1 ;   C2 - H5
   3   24    1 ;   C2 - H6
   4    7    1 ;   C3 - C6
   4   22    1 ;   C3 - H4
   5   19    1 ;   C4 - H1
   6    3    1 ;   C5 - C2
   6    7    1 ;   C5 - C6
   6   20    1 ;   C5 - H2
   7   10    1 ;   C6 - C9
   7   19    1 ;   C6 - H1
   7   27    1 ;   C6 - H9
   8   11    1 ;   C7 - C10
   8   17    1 ;   C7 - C16
   9   12    1 ;   C8 - C11
   9   23    1 ;   C8 - H5
   9   24    1 ;   C8 - H6
   9   28    1 ;   C8 - H10
   9   29    1 ;   C8 - H11
   9   39    1 ;   C8 - H21
   9   40    1 ;   C8 - H22
   9   41    1 ;   C8 - H23
  10   13    1 ;   C9 - C12
  10   25    1;    C9 - H7
  10   26    1 ;   C9 - H8
  10   30    1 ;   C9 - H12
  10   31    1 ;   C9 - H13
  11   14    1 ;  C10 - C13
  11   27    1 ;  C10 - H9
  11   32    1 ;  C10 - H14
  11   39    1 ;  C10 - H21
  11   40    1 ;  C10 - H22
  11   41    1 ;  C10 - H23
  12   15    1 ;  C11 - C14
  12   16    1 ;  C11 - C15
  12   17    1 ;  C11 - C16
  13   28    1 ;  C12 - H10
  13   29    1 ;  C12 - H11
```

```
   13    33     1 ;    C12 - H15
   13    34     1 ;    C12 - H16
   13    35     1 ;    C12 - H17
   13    36     1 ;    C12 - H18
   13    37     1 ;    C12 - H19
   13    38     1 ;    C12 - H20
   14    30     1 ;    C13 - H12
   14    31     1 ;    C13 - H13
   15    32     1 ;    C14 - H14
   15    36     1 ;    C14 - H18
   15    37     1 ;    C14 - H19
   15    38     1 ;    C14 - H20
   16    32     1 ;    C15 - H14
   16    33     1 ;    C15 - H15
   16    34     1 ;    C15 - H16
   16    35     1 ;    C15 - H17
   17    27     1 ;    C16 - H9
   17    28     1 ;    C16 - H10
   17    29     1 ;    C16 - H11
   18     3     1 ;      H - C2
   18     5     1 ;      H - C4
   18     7     1 ;      H - C6
   18    22     1 ;      H - H4
   19    20     1 ;     H1 - H2
   20    21     1 ;     H2 - H3
   21    22     1 ;     H3 - H4
   23    25     1 ;     H5 - H7
   23    26     1 ;     H5 - H8
   24    25     1 ;     H6 - H7
   24    26     1 ;     H6 - H8
   25    27     1 ;     H7 - H9
   26    27     1 ;     H8 - H9
   28    30     1 ;    H10 - H12
   28    31     1 ;    H10 - H13
   29    30     1 ;    H11 - H12
   29    31     1 ;    H11 - H13
   30    32     1 ;    H12 - H14
   31    32     1 ;    H13 - H14

[ angles ]
;  ai    aj    ak    funct   theta         cth
    1     2     3     1    1.2002e+02    5.5731e+02 ;      C - C1     - C2
    1     2     7     1    1.2077e+02    5.3137e+02 ;      C - C1     - C6
    1     6     5     1    1.2002e+02    5.5731e+02 ;      C - C5     - C4
    1     6    22     1    1.1988e+02    4.0334e+02 ;      C - C5     - H4
    2     1     6     1    1.2002e+02    5.5731e+02 ;     C1 - C      - C5
    2     1    18     1    1.1988e+02    4.0334e+02 ;     C1 - C      - H
    2     3     4     1    1.2002e+02    5.5731e+02 ;     C1 - C2     - C3
    2     3    19     1    1.1988e+02    4.0334e+02 ;     C1 - C2     - H1
    2     7     8     1    1.1207e+02    5.2802e+02 ;     C1 - C6     - C7
    2     7    23     1    1.1047e+02    3.9162e+02 ;     C1 - C6     - H5
    2     7    24     1    1.1047e+02    3.9162e+02 ;     C1 - C6     - H6
    3     2     7     1    1.2077e+02    5.3137e+02 ;     C2 - C1     - C6
```

3	4	5	1	1.2002e+02	5.5731e+02 ;	C2 - C3 - C4
3	4	20	1	1.1988e+02	4.0334e+02 ;	C2 - C3 - H2
4	3	19	1	1.1988e+02	4.0334e+02 ;	C3 - C2 - H1
4	5	6	1	1.2002e+02	5.5731e+02 ;	C3 - C4 - C5
4	5	21	1	1.1988e+02	4.0334e+02 ;	C3 - C4 - H3
5	4	20	1	1.1988e+02	4.0334e+02 ;	C4 - C3 - H2
5	6	22	1	1.1988e+02	4.0334e+02 ;	C4 - C5 - H4
6	1	18	1	1.1988e+02	4.0334e+02 ;	C5 - C - H
6	5	21	1	1.1988e+02	4.0334e+02 ;	C5 - C4 - H3
7	8	9	1	1.1156e+02	5.3053e+02 ;	C6 - C7 - C8
7	8	25	1	1.0980e+02	3.8744e+02 ;	C6 - C7 - H7
7	8	26	1	1.0980e+02	3.8744e+02 ;	C6 - C7 - H8
8	7	23	1	1.0980e+02	3.8744e+02 ;	C7 - C6 - H5
8	7	24	1	1.0980e+02	3.8744e+02 ;	C7 - C6 - H6
8	9	10	1	1.2363e+02	5.3639e+02 ;	C7 - C8 - C9
8	9	27	1	1.1568e+02	3.8409e+02 ;	C7 - C8 - H9
9	8	25	1	1.1036e+02	3.9330e+02 ;	C8 - C7 - H7
9	8	26	1	1.1036e+02	3.9330e+02 ;	C8 - C7 - H8
9	10	11	1	1.2363e+02	5.3639e+02 ;	C8 - C9 - C10
9	10	17	1	1.2363e+02	5.3639e+02 ;	C8 - C9 - C16
10	9	27	1	1.2043e+02	4.1756e+02 ;	C9 - C8 - H9
10	11	12	1	1.1156e+02	5.3053e+02 ;	C9 - C10 - C11
10	11	28	1	1.1036e+02	3.9330e+02 ;	C9 - C10 - H10
10	11	29	1	1.1036e+02	3.9330e+02 ;	C9 - C10 - H11
10	17	39	1	1.1036e+02	3.9330e+02 ;	C9 - C16 - H21
10	17	40	1	1.1036e+02	3.9330e+02 ;	C9 - C16 - H22
10	17	41	1	1.1036e+02	3.9330e+02 ;	C9 - C16 - H23
11	10	17	1	1.1565e+02	5.2635e+02 ;	C10 - C9 - C16
11	12	13	1	1.1156e+02	5.3053e+02 ;	C10 - C11 - C12
11	12	30	1	1.0980e+02	3.8744e+02 ;	C10 - C11 - H12
11	12	31	1	1.0980e+02	3.8744e+02 ;	C10 - C11 - H13
12	11	28	1	1.0980e+02	3.8744e+02 ;	C11 - C10 - H10
12	11	29	1	1.0980e+02	3.8744e+02 ;	C11 - C10 - H11
12	13	14	1	1.2363e+02	5.3639e+02 ;	C11 - C12 - C13
12	13	32	1	1.1568e+02	3.8409e+02 ;	C11 - C12 - H14
13	12	30	1	1.1036e+02	3.9330e+02 ;	C12 - C11 - H12
13	12	31	1	1.1036e+02	3.9330e+02 ;	C12 - C11 - H13
13	14	15	1	1.2363e+02	5.3639e+02 ;	C12 - C13 - C14
13	14	16	1	1.2363e+02	5.3639e+02 ;	C12 - C13 - C15
14	13	32	1	1.2043e+02	4.1756e+02 ;	C13 - C12 - H14
14	15	33	1	1.1036e+02	3.9330e+02 ;	C13 - C14 - H15
14	15	34	1	1.1036e+02	3.9330e+02 ;	C13 - C14 - H16
14	15	35	1	1.1036e+02	3.9330e+02 ;	C13 - C14 - H17
14	16	36	1	1.1036e+02	3.9330e+02 ;	C13 - C15 - H18
14	16	37	1	1.1036e+02	3.9330e+02 ;	C13 - C15 - H19
14	16	38	1	1.1036e+02	3.9330e+02 ;	C13 - C15 - H20
15	14	16	1	1.1565e+02	5.2635e+02 ;	C14 - C13 - C15
23	7	24	1	1.0758e+02	3.2970e+02 ;	H5 - C6 - H6
25	8	26	1	1.0758e+02	3.2970e+02 ;	H7 - C7 - H8
28	11	29	1	1.0758e+02	3.2970e+02 ;	H10 - C10 - H11
30	12	31	1	1.0758e+02	3.2970e+02 ;	H12 - C11 - H13
33	15	34	1	1.0758e+02	3.2970e+02 ;	H15 - C14 - H16
33	15	35	1	1.0758e+02	3.2970e+02 ;	H15 - C14 - H17

```
  34    15    35     1    1.0758e+02    3.2970e+02 ;    H16 - C14    - H17
  36    16    37     1    1.0758e+02    3.2970e+02 ;    H18 - C15    - H19
  36    16    38     1    1.0758e+02    3.2970e+02 ;    H18 - C15    - H20
  37    16    38     1    1.0758e+02    3.2970e+02 ;    H19 - C15    - H20
  39    17    40     1    1.0758e+02    3.2970e+02 ;    H21 - C16    - H22
  39    17    41     1    1.0758e+02    3.2970e+02 ;    H21 - C16    - H23
  40    17    41     1    1.0758e+02    3.2970e+02 ;    H22 - C16    - H23

[ dihedrals ] ; propers
; treated as RBs in GROMACS to use combine multiple AMBER torsions per quartet
;    i     j     k     l  func   C0        C1        C2        C3        C4        C5
    1     2     3     4     3   30.33400    0.00000  -30.33400    0.00000    0.00000    0.00000 ;      C-   C1-
C2-   C3
    1     2     3    19     3   30.33400    0.00000  -30.33400    0.00000    0.00000    0.00000 ;      C-   C1-
C2-   H1
    1     2     7     8     3   0.00000   0.00000   0.00000   0.00000   0.00000   0.00000 ;    C-   C1-   C6-
C7
    1     2     7    23     3   0.00000   0.00000   0.00000   0.00000   0.00000   0.00000 ;    C-   C1-   C6-
H5
    1     2     7    24     3   0.00000   0.00000   0.00000   0.00000   0.00000   0.00000 ;    C-   C1-   C6-
H6
    1     6     5     4     3   30.33400    0.00000  -30.33400    0.00000    0.00000    0.00000 ;      C-   C5-
C4-   C3
    1     6     5    21     3   30.33400    0.00000  -30.33400    0.00000    0.00000    0.00000 ;      C-   C5-
C4-   H3
    2     1     6     5     3   30.33400    0.00000  -30.33400    0.00000    0.00000    0.00000 ;    C1-     C-
C5-   C4
    2     1     6    22     3   30.33400    0.00000  -30.33400    0.00000    0.00000    0.00000 ;    C1-     C-
C5-   H4
    2     3     4     5     3   30.33400    0.00000  -30.33400    0.00000    0.00000    0.00000 ;    C1-   C2-
C3-   C4
    2     3     4    20     3   30.33400    0.00000  -30.33400    0.00000    0.00000    0.00000 ;    C1-   C2-
C3-   H2
    2     7     8     9     3    0.65084    1.95253    0.00000   -2.60338    0.00000    0.00000 ;    C1-   C6-
C7-   C8
    2     7     8    25     3    0.65084    1.95253    0.00000   -2.60338    0.00000    0.00000 ;    C1-   C6-
C7-   H7
    2     7     8    26     3    0.65084    1.95253    0.00000   -2.60338    0.00000    0.00000 ;    C1-   C6-
C7-   H8
    3     2     7     8     3   0.00000   0.00000   0.00000   0.00000   0.00000   0.00000 ;   C2-   C1-   C6-
C7
    3     2     7    23     3    0.00000    0.00000    0.00000    0.00000    0.00000    0.00000 ;    C2 -   C1-
C6-   H5
    3     2     7    24     3    0.00000    0.00000    0.00000    0.00000    0.00000    0.00000 ;    C2-   C1-
C6-   H6
    3     4     5     6     3   30.33400    0.00000  -30.33400    0.00000    0.00000    0.00000 ;    C2-   C3-
C4-   C5
    3     4     5    21     3   30.33400    0.00000  -30.33400    0.00000    0.00000    0.00000 ;    C2-   C3-
C4-   H3
    4     3     2     7     3   30.33400    0.00000  -30.33400    0.00000    0.00000    0.00000 ;    C3-   C2-
C1-   C6
    4     5     6    22     3   30.33400    0.00000  -30.33400    0.00000    0.00000    0.00000 ;    C3-   C4-
C5-   H4
```

5 4 3 19 3 30.33400 0.00000 -30.33400 0.00000 0.00000 0.00000 ; C4- C3- C2- H1
6 1 2 3 3 30.33400 0.00000 -30.33400 0.00000 0.00000 0.00000 ; C5- C- C1- C2
6 1 2 7 3 30.33400 0.00000 -30.33400 0.00000 0.00000 0.00000 ; C5- C- C1- C6
6 5 4 20 3 30.33400 0.00000 -30.33400 0.00000 0.00000 0.00000 ; C5- C4- C3- H2
7 2 3 19 3 30.33400 0.00000 -30.33400 0.00000 0.00000 0.00000 ; C6- C1- C2- H1
7 8 9 10 3 0.00000 0.00000 0.00000 0.00000 0.00000 0.00000 ; C6 - C7- C8- C9
7 8 9 27 3 0.00000 0.00000 0.00000 0.00000 0.00000 0.00000 ; C6 - C7- C8- H9
8 9 10 11 3 63.59680 7.94960 -55.64720 0.00000 0.00000 0.00000 ; C7- C8- C9- C10
8 9 10 17 3 63.59680 7.94960 -55.64720 0.00000 0.00000 0.00000 ; C7- C8- C9- C16
9 8 7 23 3 0.65084 1.95253 0.00000 -2.60338 0.00000 0.00000 ; C8- C7- C6- H5
9 8 7 24 3 0.65084 1.95253 0.00000 -2.60338 0.00000 0.00000 ; C8- C7- C6- H6
9 10 11 12 3 0.00000 0.00000 0.00000 0.00000 0.00000 0.00000 ; C8 - C9- C10- C11
9 10 11 28 3 6.40152 -9.58136 0.00000 6.35968 0.00000 0.00000 ; C8- C9- C10- H10
9 10 11 29 3 6.40152 -9.58136 0.00000 6.35968 0.00000 0.00000 ; C8- C9- C10- H11
9 10 17 39 3 6.40152 -9.58136 0.00000 6.35968 0.00000 0.00000 ; C8- C9- C16- H21
9 10 17 40 3 6.40152 -9.58136 0.00000 6.35968 0.00000 0.00000 ; C8- C9- C16- H22
9 10 17 41 3 6.40152 -9.58136 0.00000 6.35968 0.00000 0.00000 ; C8- C9- C16- H23
10 9 8 25 3 6.40152 -9.58136 0.00000 6.35968 0.00000 0.00000 ; C9- C8- C7- H7
10 9 8 26 3 6.40152 -9.58136 0.00000 6.35968 0.00000 0.00000 ; C9- C8- C7- H8
10 11 12 13 3 0.65084 1.95253 0.00000 -2.60338 0.00000 0.00000 ; C9- C10- C11- C12
10 11 12 30 3 0.65084 1.95253 0.00000 -2.60338 0.00000 0.00000 ; C9- C10- C11- H12
10 11 12 31 3 0.65084 1.95253 0.00000 -2.60338 0.00000 0.00000 ; C9- C10- C11- H13
11 10 9 27 3 55.64720 0.00000 -55.64720 0.00000 0.00000 0.00000 ; C10- C9- C8- H9
11 10 17 39 3 0.00000 0.00000 0.00000 0.00000 0.00000 0.00000 ; C10- C9- C16- H21
11 10 17 40 3 0.00000 0.00000 0.00000 0.00000 0.00000 0.00000 ; C10 - C9- C16- H22
11 10 17 41 3 0.00000 0.00000 0.00000 0.00000 0.00000 0.00000 ; C10- C9- C16- H23

11 12 13 14 3 0.00000 0.00000 0.00000 0.00000 0.00000 0.00000 ; C10- C11-
C12- C13
11 12 13 32 3 0.00000 0.00000 0.00000 0.00000 0.00000 0.00000 ; C10- C11-
C12- H14
12 11 10 17 3 0.00000 0.00000 0.00000 0.00000 0.00000 0.00000 ; C11- C10-
C9- C16
12 13 14 15 3 63.59680 7.94960 -55.64720 0.00000 0.00000 0.00000 ; C11- C12-
C13- C14
12 13 14 16 3 63.59680 7.94960 -55.64720 0.00000 0.00000 0.00000 ; C11- C12-
C13- C15
13 12 11 28 3 0.65084 1.95253 0.00000 -2.60338 0.00000 0.00000 ; C12- C11-
C10- H10
13 12 11 29 3 0.65084 1.95253 0.00000 -2.60338 0.00000 0.00000 ; C12- C11-
C10- H11
13 14 15 33 3 6.40152 -9.58136 0.00000 6.35968 0.00000 0.00000 ; C12- C13-
C14- H15
13 14 15 34 3 6.40152 -9.58136 0.00000 6.35968 0.00000 0.00000 ; C12- C13-
C14- H16
13 14 15 35 3 6.40152 -9.58136 0.00000 6.35968 0.00000 0.00000 ; C12- C13-
C14- H17
13 14 16 36 3 6.40152 -9.58136 0.00000 6.35968 0.00000 0.00000 ; C12- C13-
C15- H18
13 14 16 37 3 6.40152 -9.58136 0.00000 6.35968 0.00000 0.00000 ; C12- C13-
C15- H19
13 14 16 38 3 6.40152 -9.58136 0.00000 6.35968 0.00000 0.00000 ; C12- C13-
C15- H20
14 13 12 30 3 6.40152 -9.58136 0.00000 6.35968 0.00000 0.00000 ; C13- C12-
C11- H12
14 13 12 31 3 6.40152 -9.58136 0.00000 6.35968 0.00000 0.00000 ; C13- C12-
C11- H13
15 14 13 32 3 55.64720 0.00000 -55.64720 0.00000 0.00000 0.00000 ; C14- C13-
C12- H14
15 14 16 36 3 0.00000 0.00000 0.00000 0.00000 0.00000 0.00000 ; C14- C13-
C15- H18
15 14 16 37 3 0.00000 0.00000 0.00000 0.00000 0.00000 0.00000 ; C14- C13-
C15- H19
15 14 16 38 3 0.00000 0.00000 0.00000 0.00000 0.00000 0.00000 ; C14- C13-
C15- H20
16 14 13 32 3 55.64720 0.00000 -55.64720 0.00000 0.00000 0.00000 ; C15- C13-
C12- H14
16 14 15 33 3 0.00000 0.00000 0.00000 0.00000 0.00000 0.00000 ; C15- C13-
C14- H15
16 14 15 34 3 0.00000 0.00000 0.00000 0.00000 0.00000 0.00000 ; C15- C13-
C14- H16
16 14 15 35 3 0.00000 0.00000 0.00000 0.00000 0.00000 0.00000 ; C15- C13-
C14- H17
17 10 9 27 3 55.64720 0.00000 -55.64720 0.00000 0.00000 0.00000 ; C16- C9-
C8- H9
17 10 11 28 3 0.00000 0.00000 0.00000 0.00000 0.00000 0.00000 ; C16- C9-
C10- H10
17 10 11 29 3 0.00000 0.00000 0.00000 0.00000 0.00000 0.00000 ; C16- C9-
C10- H11

```
   18     1     2     3     3  30.33400   0.00000  -30.33400   0.00000   0.00000   0.00000 ;     H-    C-   C1-   C2
   18     1     2     7     3  30.33400   0.00000  -30.33400   0.00000   0.00000   0.00000 ;     H-    C-   C1-   C6
   18     1     6     5     3  30.33400   0.00000  -30.33400   0.00000   0.00000   0.00000 ;     H-    C-   C5-   C4
   18     1     6    22     3  30.33400   0.00000  -30.33400   0.00000   0.00000   0.00000 ;     H-    C-   C5-   H4
   19     3     4    20     3  30.33400   0.00000  -30.33400   0.00000   0.00000   0.00000 ;    H1-   C2-   C3-   H2
   20     4     5    21     3  30.33400   0.00000  -30.33400   0.00000   0.00000   0.00000 ;    H2-   C3-   C4-   H3
   21     5     6    22     3  30.33400   0.00000  -30.33400   0.00000   0.00000   0.00000 ;    H3-   C4-   C5-   H4
   23     7     8    25     3   0.62760   1.88280   0.00000  -2.51040   0.00000   0.00000 ;    H5-   C6-   C7-   H7
   23     7     8    26     3   0.62760   1.88280   0.00000  -2.51040   0.00000   0.00000 ;    H5-   C6-   C7-   H8
   24     7     8    25     3   0.62760   1.88280   0.00000  -2.51040   0.00000   0.00000 ;    H6-   C6-   C7-   H7
   24     7     8    26     3   0.62760   1.88280   0.00000  -2.51040   0.00000   0.00000 ;    H6-   C6-   C7-   H8
   25     8     9    27     3   0.00000   0.00000   0.00000   0.00000   0.00000   0.00000 ;    H7-   C7-   C8-   H9
   26     8     9    27     3   0.00000   0.00000   0.00000   0.00000   0.00000   0.00000 ;    H8-   C7-   C8-   H9
   28    11    12    30     3   0.62760   1.88280   0.00000  -2.51040   0.00000   0.00000 ;   H10-  C10-  C11-  H12
   28    11    12    31     3   0.62760   1.88280   0.00000  -2.51040   0.00000   0.00000 ;   H10-  C10-  C11-  H13
   29    11    12    30     3   0.62760   1.88280   0.00000  -2.51040   0.00000   0.00000 ;   H11-  C10-  C11-  H12
   29    11    12    31     3   0.62760   1.88280   0.00000  -2.51040   0.00000   0.00000 ;   H11-  C10-  C11-  H13
   30    12    13    32     3   0.00000   0.00000   0.00000   0.00000   0.00000   0.00000 ;   H12-  C11-  C12-  H14
   31    12    13    32     3   0.00000   0.00000   0.00000   0.00000   0.00000   0.00000 ;   H13-  C11-  C12-  H14

[ dihedrals ] ; impropers
; treated as propers in GROMACS to use correct AMBER analytical function
;    i      j      k      l   func   phase     kd      pn
     1      3      2      7      1  180.00   4.60240   2 ;     C-   C2-   C1-   C6
     1      5      6     22      1  180.00   4.60240   2 ;     C-   C4-   C5-   H4
     2      4      3     19      1  180.00   4.60240   2 ;    C1-   C3-   C2-   H1
     3      5      4     20      1  180.00   4.60240   2 ;    C2-   C4-   C3-   H2
     4      6      5     21      1  180.00   4.60240   2 ;    C3-   C5-   C4-   H3
     9     11     10     17      1  180.00   4.60240   2 ;    C8-  C10-   C9-  C16
    10      8      9     27      1  180.00   4.60240   2 ;    C9-   C7-   C8-   H9
    13     15     14     16      1  180.00   4.60240   2 ;   C12-  C14-  C13-  C15
    14     12     13     32      1  180.00   4.60240   2 ;   C13-  C11-  C12-  H14
    18      1      6      2      1  180.00   4.60240   2 ;     H-    C-   C5-   C1
```

PFTB.itp

```
[ molecule type ]
;name           nrexcl
 SOL            3

[ atoms ]
    1  c3    1  SOL    C   1    -0.231634     12.01000 ; qtot 0.000
    2  c3    1  SOL   C1   2     0.561837     12.01000 ; qtot 0.000
    3  c3    1  SOL   C2   3     0.552406     12.01000 ; qtot 0.000
    4  oh    1  SOL    O   4    -0.455242     16.00000 ; qtot 0.000
    5  c3    1  SOL   C3   5     0.626784     12.01000 ; qtot 0.000
    6   f    1  SOL    F   6    -0.176568     19.00000 ; qtot 0.000
    7   f    1  SOL   F1   7    -0.158578     19.00000 ; qtot 0.000
    8   f    1  SOL   F2   8    -0.153817     19.00000 ; qtot 0.000
    9   f    1  SOL   F3   9    -0.173911     19.00000 ; qtot 0.000
   10   f    1  SOL   F4  10    -0.151273     19.00000 ; qtot 0.000
   11   f    1  SOL   F5  11    -0.157313     19.00000 ; qtot 0.000
   12   f    1  SOL   F6  12    -0.194871     19.00000 ; qtot 0.000
   13   f    1  SOL   F7  13    -0.155073     19.00000 ; qtot 0.000
   14   f    1  SOL   F8  14    -0.155624     19.00000 ; qtot 0.000
   15  ho    1  SOL    H  15     0.422876      1.00800 ; qtot 0.000

[ bonds ]

;   ai     aj funct   r           k
    1     2  1   1.5375e-01   2.5179e+05 ;     C - C1
    1     3  1   1.5375e-01   2.5179e+05 ;     C - C2
    1     4  1   1.4233e-01   2.6501e+05 ;     C - O
    1     5  1   1.5375e-01   2.5179e+05 ;     C - C3
    2     6  1   1.3497e-01   2.9865e+05 ;    C1 - F
    2     7  1   1.3497e-01   2.9865e+05 ;    C1 - F1
    2     8  1   1.3497e-01   2.9865e+05 ;    C1 - F2
    3     9  1   1.3497e-01   2.9865e+05 ;    C2 - F3
    3    10  1   1.3497e-01   2.9865e+05 ;    C2 - F4
    3    11  1   1.3497e-01   2.9865e+05 ;    C2 - F5
    4    15  1   9.7300e-02   3.1079e+05 ;     O - H
    5    12  1   1.3497e-01   2.9865e+05 ;    C3 - F6
    5    13  1   1.3497e-01   2.9865e+05 ;    C3 - F7
    5    14  1   1.3497e-01   2.9865e+05 ;    C3 - F8

[ pairs ]

;   ai     aj    funct
    2     9     1 ;    C1 - F3
    2    10     1 ;    C1 - F4
    2    11     1 ;    C1 - F5
    2    12     1 ;    C1 - F6
    2    13     1 ;    C1 - F7
    2    14     1 ;    C1 - F8
    2    15     1 ;    C1 - H
    3     6     1 ;    C2 - F
```

```
    3     7     1 ;     C2 - F1
    3     8     1 ;     C2 - F2
    3    12     1 ;     C2 - F6
    3    13     1 ;     C2 - F7
    3    14     1 ;     C2 - F8
    3    15     1 ;     C2 - H
    4     6     1 ;     O - F
    4     7     1 ;     O - F1
    4     8     1 ;     O - F2
    4     9     1 ;     O - F3
    4    10     1 ;     O - F4
    4    11     1 ;     O - F5
    4    12     1 ;     O - F6
    4    13     1 ;     O - F7
    4    14     1 ;     O - F8
    5     6     1 ;     C3 - F
    5     7     1 ;     C3 - F1
    5     8     1 ;     C3 - F2
    5     9     1 ;     C3 - F3
    5    10     1 ;     C3 - F4
    5    11     1 ;     C3 - F5
    5    15     1 ;     C3 - H

[ angles ]

;   ai    aj    ak    funct   theta        cth
    1     2     6     1   1.0924e+02    5.5312e+02 ;     C - C1    - F
    1     2     7     1   1.0924e+02    5.5312e+02 ;     C - C1    - F1
    1     2     8     1   1.0924e+02    5.5312e+02 ;     C - C1    - F2
    1     3     9     1   1.0924e+02    5.5312e+02 ;     C - C2    - F3
    1     3    10     1   1.0924e+02    5.5312e+02 ;     C - C2    - F4
    1     3    11     1   1.0924e+02    5.5312e+02 ;     C - C2    - F5
    1     4    15     1   1.0726e+02    3.9664e+02 ;     C - O     - H
    1     5    12     1   1.0924e+02    5.5312e+02 ;     C - C3    - F6
    1     5    13     1   1.0924e+02    5.5312e+02 ;     C - C3    - F7
    1     5    14     1   1.0924e+02    5.5312e+02 ;     C - C3    - F8
    2     1     3     1   1.1151e+02    5.2635e+02 ;    C1 - C     - C2
    2     1     4     1   1.1019e+02    5.6484e+02 ;    C1 - C     - O
    2     1     5     1   1.1151e+02    5.2635e+02 ;    C1 - C     - C3
    3     1     4     1   1.1019e+02    5.6484e+02 ;    C2 - C     - O
    3     1     5     1   1.1151e+02    5.2635e+02 ;    C2 - C     - C3
    4     1     5     1   1.1019e+02    5.6484e+02 ;     O - C     - C3
    6     2     7     1   1.0736e+02    5.9329e+02 ;     F - C1    - F1
    6     2     8     1   1.0736e+02    5.9329e+02 ;     F - C1    - F2
    7     2     8     1   1.0736e+02    5.9329e+02 ;    F1 - C1    - F2
    9     3    10     1   1.0736e+02    5.9329e+02 ;    F3 - C2    - F4
    9     3    11     1   1.0736e+02    5.9329e+02 ;    F3 - C2    - F5
   10     3    11     1   1.0736e+02    5.9329e+02 ;    F4 - C2    - F5
   12     5    13     1   1.0736e+02    5.9329e+02 ;    F6 - C3    - F7
   12     5    14     1   1.0736e+02    5.9329e+02 ;    F6 - C3    - F8
   13     5    14     1   1.0736e+02    5.9329e+02 ;    F7 - C3    - F8

[ dihedrals ] ; propers
```

```
; treated as RBs in GROMACS to use combine multiple AMBER torsions per quartet
;  i   j   k   l  func  C0     C1     C2     C3     C4     C5
   2   1   3   9   3  0.65084  1.95253  0.00000  -2.60338  0.00000  0.00000 ;   C1-   C-   C2-
F3
   2   1   3  10   3  0.65084  1.95253  0.00000  -2.60338  0.00000  0.00000 ;   C1-   C-
C2-  F4
   2   1   3  11   3  0.65084  1.95253  0.00000  -2.60338  0.00000  0.00000 ;   C1-   C-
C2-  F5
   2   1   4  15   3  1.71544  0.96232  0.00000  -2.67776  0.00000  0.00000 ;   C1-   C-   O-
H
   2   1   5  12   3  0.65084  1.95253  0.00000  -2.60338  0.00000  0.00000 ;   C1-   C-
C3-  F6
   2   1   5  13   3  0.65084  1.95253  0.00000  -2.60338  0.00000  0.00000 ;   C1-   C-
C3-  F7
   2   1   5  14   3  0.65084  1.95253  0.00000  -2.60338  0.00000  0.00000 ;   C1-   C-
C3-  F8
   3   1   2   6   3  0.65084  1.95253  0.00000  -2.60338  0.00000  0.00000 ;   C2-   C-   C1-
F
   3   1   2   7   3  0.65084  1.95253  0.00000  -2.60338  0.00000  0.00000 ;   C2-   C-   C1-
F1
   3   1   2   8   3  0.65084  1.95253  0.00000  -2.60338  0.00000  0.00000 ;   C2-   C-   C1-
F2
   3   1   4  15   3  1.71544  0.96232  0.00000  -2.67776  0.00000  0.00000 ;   C2-   C-   O-
H
   3   1   5  12   3  0.65084  1.95253  0.00000  -2.60338  0.00000  0.00000 ;   C2-   C-
C3-  F6
   3   1   5  13   3  0.65084  1.95253  0.00000  -2.60338  0.00000  0.00000 ;   C2-   C-
C3-  F7
   3   1   5  14   3  0.65084  1.95253  0.00000  -2.60338  0.00000  0.00000 ;   C2-   C-
C3-  F8
   4   1   2   6   3  0.65084  1.95253  0.00000  -2.60338  0.00000  0.00000 ;   O-   C-   C1-
F
   4   1   2   7   3  0.65084  1.95253  0.00000  -2.60338  0.00000  0.00000 ;   O-   C-   C1-
F1
   4   1   2   8   3  0.65084  1.95253  0.00000  -2.60338  0.00000  0.00000 ;   O-   C-   C1-
F2
   4   1   3   9   3  0.65084  1.95253  0.00000  -2.60338  0.00000  0.00000 ;   O-   C-   C2-
F3
   4   1   3  10   3  0.65084  1.95253  0.00000  -2.60338  0.00000  0.00000 ;   O-   C-   C2-
F4
   4   1   3  11   3  0.65084  1.95253  0.00000  -2.60338  0.00000  0.00000 ;   O-   C-   C2-
F5
   4   1   5  12   3  0.65084  1.95253  0.00000  -2.60338  0.00000  0.00000 ;   O-   C-   C3-
F6
   4   1   5  13   3  0.65084  1.95253  0.00000  -2.60338  0.00000  0.00000 ;   O-   C-   C3-
F7
   4   1   5  14   3  0.65084  1.95253  0.00000  -2.60338  0.00000  0.00000 ;   O-   C-   C3-
F8
   5   1   2   6   3  0.65084  1.95253  0.00000  -2.60338  0.00000  0.00000 ;   C3-   C-   C1-
F
   5   1   2   7   3  0.65084  1.95253  0.00000  -2.60338  0.00000  0.00000 ;   C3-   C-   C1-
F1
```

5 1 2 8 3 0.65084 1.95253 0.00000 -2.60338 0.00000 0.00000 ; C3- C- C1-
F2
5 1 3 9 3 0.65084 1.95253 0.00000 -2.60338 0.00000 0.00000 ; C3- C- C2-
F3
5 1 3 10 3 0.65084 1.95253 0.00000 -2.60338 0.00000 0.00000 ; C3- C-
C2- F4
5 1 3 11 3 0.65084 1.95253 0.00000 -2.60338 0.00000 0.00000 ; C3- C-
C2- F5
5 1 4 15 3 1.71544 0.96232 0.00000 -2.67776 0.00000 0.00000 ; C3- C- O-
H

```
Py.itp
[ molecule type ]
;name          nrexcl
 KAT           3

[ atoms ]
    1   ca    1   KAT    C    1    -0.005220    12.01000 ; qtot 0.000
    2   ca    1   KAT    C1   2    -0.097410    12.01000 ; qtot 0.000
    3   ca    1   KAT    C2   3     0.012648    12.01000 ; qtot 0.000
    4   ca    1   KAT    C3   4    -0.095690    12.01000 ; qtot 0.000
    5   ca    1   KAT    C4   5    -0.006692    12.01000 ; qtot 0.000
    6   nb    1   KAT    N    6     0.173897    14.01000 ; qtot 0.000
    7   h4    1   KAT    H    7     0.224185     1.00800 ; qtot 0.000
    8   ha    1   KAT    H1   8     0.197455     1.00800 ; qtot 0.000
    9   ha    1   KAT    H2   9     0.175383     1.00800 ; qtot 0.000
   10   ha    1   KAT    H3   10    0.196707     1.00800 ; qtot 0.000
   11   h4    1   KAT    H4   11    0.224737     1.00800 ; qtot 0.000


[ bonds ]
;   ai    aj funct   r           k
    1     2   1   1.3984e-01   3.8585e+05 ;     C - C1
    1     6   1   1.3390e-01   4.0836e+05 ;     C - N
    1     7   1   1.0890e-01   2.8577e+05 ;     C - H
    2     3   1   1.3984e-01   3.8585e+05 ;    C1 - C2
    2     8   1   1.0860e-01   2.8937e+05 ;    C1 - H1
    3     4   1   1.3984e-01   3.8585e+05 ;    C2 - C3
    3     9   1   1.0860e-01   2.8937e+05 ;    C2 - H2
    4     5   1   1.3984e-01   3.8585e+05 ;    C3 - C4
    4    10   1   1.0860e-01   2.8937e+05 ;    C3 - H3
    5     6   1   1.3390e-01   4.0836e+05 ;    C4 - N
    5    11   1   1.0890e-01   2.8577e+05 ;    C4 - H4

[ pairs ]
;   ai    aj    funct
    1     4     1 ;     C - C3
    1     9     1 ;     C - H2
    1    11     1 ;     C - H4
    2     5     1 ;    C1 - C4
    2    10     1 ;    C1 - H3
    3     6     1 ;    C2 - N
    3    11     1 ;    C2 - H4
    4     8     1 ;    C3 - H1
    5     9     1 ;    C4 - H2
    6     8     1 ;     N - H1
    6    10     1 ;     N - H3
    7     3     1 ;     H - C2
    7     5     1 ;     H - C4
    7     8     1 ;     H - H1
    8     9     1 ;    H1 - H2
    9    10     1 ;    H2 - H3
   10    11     1 ;    H3 - H4
```

```
[ angles ]
;  ai    aj    ak   funct   theta        cth
   1     2     3     1   1.2002e+02   5.5731e+02 ;     C - C1    - C2
   1     2     8     1   1.1988e+02   4.0334e+02 ;     C - C1    - H1
   1     6     5     1   1.1722e+02   5.7153e+02 ;     C - N     - C4
   2     1     6     1   1.2294e+02   5.7572e+02 ;    C1 - C     - N
   2     1     7     1   1.2034e+02   4.0250e+02 ;    C1 - C     - H
   2     3     4     1   1.2002e+02   5.5731e+02 ;    C1 - C2    - C3
   2     3     9     1   1.1988e+02   4.0334e+02 ;    C1 - C2    - H2
   3     2     8     1   1.1988e+02   4.0334e+02 ;    C2 - C1    - H1
   3     4     5     1   1.2002e+02   5.5731e+02 ;    C2 - C3    - C4
   3     4    10     1   1.1988e+02   4.0334e+02 ;    C2 - C3    - H3
   4     3     9     1   1.1988e+02   4.0334e+02 ;    C3 - C2    - H2
   4     5     6     1   1.2294e+02   5.7572e+02 ;    C3 - C4    - N
   4     5    11     1   1.2034e+02   4.0250e+02 ;    C3 - C4    - H4
   5     4    10     1   1.1988e+02   4.0334e+02 ;    C4 - C3    - H3
   6     1     7     1   1.1603e+02   4.3430e+02 ;     N - C     - H
   6     5    11     1   1.1603e+02   4.3430e+02 ;     N - C4    - H4

[ dihedrals ] ; propers
; treated as RBs in GROMACS to use combine multiple AMBER torsions per quartet
;    i      j      k      l   func   C0        C1        C2        C3        C4        C5
   1     2     3     4     3   30.33400    0.00000  -30.33400    0.00000    0.00000    0.00000 ;     C-    C1-
C2-   C3
   1     2     3     9     3   30.33400    0.00000  -30.33400    0.00000    0.00000    0.00000 ;     C-    C1-
C2-   H2
   1     6     5     4     3   40.16640    0.00000  -40.16640    0.00000    0.00000    0.00000 ;     C-     N-
C4-   C3
   1     6     5    11     3   40.16640    0.00000  -40.16640    0.00000    0.00000    0.00000 ;     C-     N-
C4-   H4
   2     1     6     5     3   40.16640    0.00000  -40.16640    0.00000    0.00000    0.00000 ;    C1-     C-
N-   C4
   2     3     4     5     3   30.33400    0.00000  -30.33400    0.00000    0.00000    0.00000 ;    C1-    C2-
C3-   C4
   2     3     4    10     3   30.33400    0.00000  -30.33400    0.00000    0.00000    0.00000 ;    C1-    C2-
C3-   H3
   3     4     5     6     3   30.33400    0.00000  -30.33400    0.00000    0.00000    0.00000 ;    C2-    C3-
C4-    N
   3     4     5    11     3   30.33400    0.00000  -30.33400    0.00000    0.00000    0.00000 ;    C2-    C3-
C4-   H4
   4     3     2     8     3   30.33400    0.00000  -30.33400    0.00000    0.00000    0.00000 ;    C3-    C2-
C1-   H1
   5     4     3     9     3   30.33400    0.00000  -30.33400    0.00000    0.00000    0.00000 ;    C4-    C3-
C2-   H2
   6     1     2     3     3   30.33400    0.00000  -30.33400    0.00000    0.00000    0.00000 ;     N-     C-
C1-   C2
   6     1     2     8     3   30.33400    0.00000  -30.33400    0.00000    0.00000    0.00000 ;     N-     C-
C1-   H1
   6     5     4    10     3   30.33400    0.00000  -30.33400    0.00000    0.00000    0.00000 ;     N-    C4-
C3-   H3
   7     1     2     3     3   30.33400    0.00000  -30.33400    0.00000    0.00000    0.00000 ;     H-     C-
C1-   C2
```

```
    7      1      2      8      3   30.33400    0.00000  -30.33400    0.00000    0.00000    0.00000 ;     H-     C-
C1-    H1
    7     1     6     5     3   40.16640    0.00000  -40.16640    0.00000    0.00000    0.00000 ;     H-    C-    N-
C4
    8     2     3     9     3   30.33400    0.00000  -30.33400    0.00000    0.00000    0.00000 ;     H1-    C1-
C2-    H2
    9     3     4    10     3   30.33400    0.00000  -30.33400    0.00000    0.00000    0.00000 ;     H2-    C2-
C3-    H3
   10     4     5    11     3   30.33400    0.00000  -30.33400    0.00000    0.00000    0.00000 ;     H3-    C3-
C4-    H4

[ dihedrals ] ; impropers
; treated as propers in GROMACS to use correct AMBER analytical function
;    i      j      k      l   func   phase     kd      pn
    1     3     2     8     1   180.00   4.60240   2 ;     C-    C2-    C1-    H1
    2     4     3     9     1   180.00   4.60240   2 ;    C1-    C3-    C2-    H2
    3     5     4    10     1   180.00   4.60240   2 ;    C2-    C4-    C3-    H3
    4    11     5     6     1   180.00   4.60240   2 ;    C3-    H4-    C4-     N
    6     1     7     2     1   180.00   4.60240   2 ;     N-     C-     H-    C1
```

PYH.itp

```
[ moleculetype ]
;name            nrexcl
 KIH             3

[ atoms ]
    1   ca     1   KIH     C    1     0.025607     12.01000 ; qtot 0.000
    2   ca     1   KIH    C1    2     -0.108771     12.01000 ; qtot 0.000
    3   ca     1   KIH    C2    3     0.052674     12.01000 ; qtot 0.000
    4   ca     1   KIH    C3    4     -0.105709     12.01000 ; qtot 0.000
    5   ca     1   KIH    C4    5     0.021791     12.01000 ; qtot 0.000
    6   na     1   KIH     N    6     -0.080585     14.01000 ; qtot 0.000
    7   h4     1   KIH     H    7     0.178498      1.00800 ; qtot 0.000
    8   ha     1   KIH    H1    8     0.178860      1.00800 ; qtot 0.000
    9   ha     1   KIH    H2    9     0.156149      1.00800 ; qtot 0.000
   10   ha     1   KIH    H3   10     0.177700      1.00800 ; qtot 0.000
   11   h4     1   KIH    H4   11     0.180099      1.00800 ; qtot 0.000
   12   hn     1   KIH    H5   12     0.323687      1.00800 ; qtot 0.000
[ bonds ]
;   ai     aj funct   r             k
    1      2   1    1.3984e-01    3.8585e+05 ;      C - C1
    1      6   1    1.3840e-01    3.5187e+05 ;      C - N
    1      7   1    1.0890e-01    2.8577e+05 ;      C - H
    2      3   1    1.3984e-01    3.8585e+05 ;     C1 - C2
    2      8   1    1.0860e-01    2.8937e+05 ;     C1 - H1
    3      4   1    1.3984e-01    3.8585e+05 ;     C2 - C3
    3      9   1    1.0860e-01    2.8937e+05 ;     C2 - H2
    4      5   1    1.3984e-01    3.8585e+05 ;     C3 - C4
    4     10   1    1.0860e-01    2.8937e+05 ;     C3 - H3
    5      6   1    1.3840e-01    3.5187e+05 ;     C4 - N
    5     11   1    1.0890e-01    2.8577e+05 ;     C4 - H4
    6     12   1    1.0100e-01    3.4175e+05 ;      N - H5

[ pairs ]
;   ai     aj    funct
    1      4      1 ;      C - C3
    1      9      1 ;      C - H2
    1     11      1 ;      C - H4
    2      5      1 ;     C1 - C4
    2     10      1 ;     C1 - H3
    2     12      1 ;     C1 - H5
    3      6      1 ;     C2 - N
    3     11      1 ;     C2 - H4
    4      8      1 ;     C3 - H1
    4     12      1 ;     C3 - H5
    5      9      1 ;     C4 - H2
    6      8      1 ;      N - H1
    6     10      1 ;      N - H3
    7      3      1 ;      H - C2
    7      5      1 ;      H - C4
    7      8      1 ;      H - H1
    7     12      1 ;      H - H5
```

```
    8     9     1 ;     H1 - H2
    9    10     1 ;     H2 - H3
   10    11     1 ;     H3 - H4
   11    12     1 ;     H4 - H5

[ angles ]
;  ai    aj    ak    funct   theta         cth
    1     2     3     1    1.2002e+02    5.5731e+02 ;      C - C1     - C2
    1     2     8     1    1.1988e+02    4.0334e+02 ;      C - C1     - H1
    1     6     5     1    1.2005e+02    5.4643e+02 ;      C - N      - C4
    1     6    12     1    1.2554e+02    3.8995e+02 ;      C - N      - H5
    2     1     6     1    1.1834e+02    5.7823e+02 ;     C1 - C      - N
    2     1     7     1    1.2034e+02    4.0250e+02 ;     C1 - C      - H
    2     3     4     1    1.2002e+02    5.5731e+02 ;     C1 - C2     - C3
    2     3     9     1    1.1988e+02    4.0334e+02 ;     C1 - C2     - H2
    3     2     8     1    1.1988e+02    4.0334e+02 ;     C2 - C1     - H1
    3     4     5     1    1.2002e+02    5.5731e+02 ;     C2 - C3     - C4
    3     4    10     1    1.1988e+02    4.0334e+02 ;     C2 - C3     - H3
    4     3     9     1    1.1988e+02    4.0334e+02 ;     C3 - C2     - H2
    4     5     6     1    1.1834e+02    5.7823e+02 ;     C3 - C4     - N
    4     5    11     1    1.2034e+02    4.0250e+02 ;     C3 - C4     - H4
    5     4    10     1    1.1988e+02    4.0334e+02 ;     C4 - C3     - H3
    5     6    12     1    1.2554e+02    3.8995e+02 ;     C4 - N      - H5
    6     1     7     1    1.1632e+02    4.2258e+02 ;      N - C      - H
    6     5    11     1    1.1632e+02    4.2258e+02 ;      N - C4     - H4

[ dihedrals ] ; propers
; treated as RBs in GROMACS to use combine multiple AMBER torsions per quartet
;    i      j      k      l   func    C0         C1         C2         C3         C4         C5
    1      2      3      4      3   30.33400    0.00000  -30.33400    0.00000    0.00000    0.00000 ;      C-    C1-
C2-    C3
    1      2      3      9      3   30.33400    0.00000  -30.33400    0.00000    0.00000    0.00000 ;      C-    C1-
C2-    H2
    1      6      5      4      3    2.51040    0.00000   -2.51040    0.00000    0.00000    0.00000 ;      C-     N-    C4-
C3
    1      6      5     11      3    2.51040    0.00000   -2.51040    0.00000    0.00000    0.00000 ;      C-     N-    C4-
H4
    2      1      6      5      3    2.51040    0.00000   -2.51040    0.00000    0.00000    0.00000 ;     C1-     C-     N-
C4
    2      1      6     12      3    2.51040    0.00000   -2.51040    0.00000    0.00000    0.00000 ;     C1-     C-     N-
H5
    2      3      4      5      3   30.33400    0.00000  -30.33400    0.00000    0.00000    0.00000 ;     C1-    C2-
C3-    C4
    2      3      4     10      3   30.33400    0.00000  -30.33400    0.00000    0.00000    0.00000 ;     C1-    C2-
C3-    H3
    3      4      5      6      3   30.33400    0.00000  -30.33400    0.00000    0.00000    0.00000 ;     C2-    C3-
C4-     N
    3      4      5     11      3   30.33400    0.00000  -30.33400    0.00000    0.00000    0.00000 ;     C2-    C3-
C4-    H4
    4      3      2      8      3   30.33400    0.00000  -30.33400    0.00000    0.00000    0.00000 ;     C3-    C2-
C1-    H1
    4      5      6     12      3    2.51040    0.00000   -2.51040    0.00000    0.00000    0.00000 ;     C3-    C4-
N-    H5
```

5 4 3 9 3 30.33400 0.00000 -30.33400 0.00000 0.00000 0.00000 ; C4- C3-
C2- H2
6 1 2 3 3 30.33400 0.00000 -30.33400 0.00000 0.00000 0.00000 ; N- C-
C1- C2
6 1 2 8 3 30.33400 0.00000 -30.33400 0.00000 0.00000 0.00000 ; N- C-
C1- H1
6 5 4 10 3 30.33400 0.00000 -30.33400 0.00000 0.00000 0.00000 ; N- C4-
C3- H3
7 1 2 3 3 30.33400 0.00000 -30.33400 0.00000 0.00000 0.00000 ; H- C-
C1- C2
7 1 2 8 3 30.33400 0.00000 -30.33400 0.00000 0.00000 0.00000 ; H- C-
C1- H1
7 1 6 5 3 2.51040 0.00000 -2.51040 0.00000 0.00000 0.00000 ; H- C- N-
C4
7 1 6 12 3 2.51040 0.00000 -2.51040 0.00000 0.00000 0.00000 ; H- C- N-
H5
8 2 3 9 3 30.33400 0.00000 -30.33400 0.00000 0.00000 0.00000 ; H1- C1-
C2- H2
9 3 4 10 3 30.33400 0.00000 -30.33400 0.00000 0.00000 0.00000 ; H2- C2-
C3- H3
10 4 5 11 3 30.33400 0.00000 -30.33400 0.00000 0.00000 0.00000 ; H3- C3-
C4- H4
11 5 6 12 3 2.51040 0.00000 -2.51040 0.00000 0.00000 0.00000 ; H4- C4-
N- H5

[dihedrals] ; impropers
; treated as propers in GROMACS to use correct AMBER analytical function
; i j k l func phase kd pn
1 3 2 8 1 180.00 4.60240 2 ; C- C2- C1- H1
1 5 6 12 1 180.00 4.60240 2 ; C- C4- N- H5
2 4 3 9 1 180.00 4.60240 2 ; C1- C3- C2- H2
3 5 4 10 1 180.00 4.60240 2 ; C2- C4- C3- H3
4 11 5 6 1 180.00 4.60240 2 ; C3- H4- C4- N
6 1 7 2 1 180.00 4.60240 2 ; N- C- H- C1
